# Supplementary material for: Assessing the Immunomodulatory Effect of Size on the Uptake and Immunogenicity of Influenza- and Hepatitis B Subunit Vaccines In Vitro
Source: Pharmaceuticals (Basel). 2022 Jul 18;15(7):887. doi: 10.3390/ph15070887 (PMC9321264; doi:10.3390/ph15070887)
Supplement: Supplementary file 1 [file pharmaceuticals-15-00887-s001.zip › pharmaceuticals-1786424-supplementary.pdf]

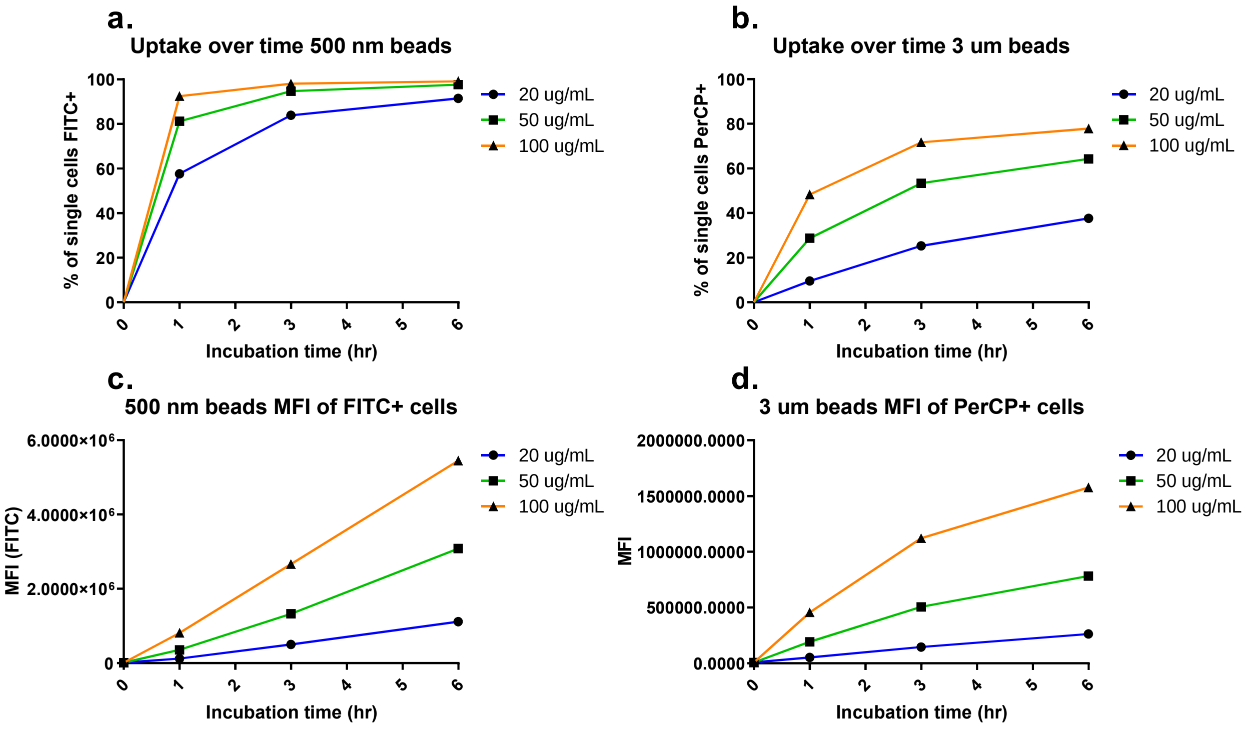

Supplementary Figure S1: Uptake of plain particles in THP-1 cells at different concentrations. (**a**) uptake over time of 500 nm fluorescent particles without antigen shows a clear pattern of uptake in 6 hours. (**b**) uptake over time of 3 µm particles. (**c**) MFI over time upon stimulation with 500 nm fluorescent particles. (**d**) MFI over time upon stimulation with 3 µm particles. As the FITC-signal for the 3 µm particles (**b,d**) was too high, samples were measured in the PerCP-channel instead of the FITC-channel.

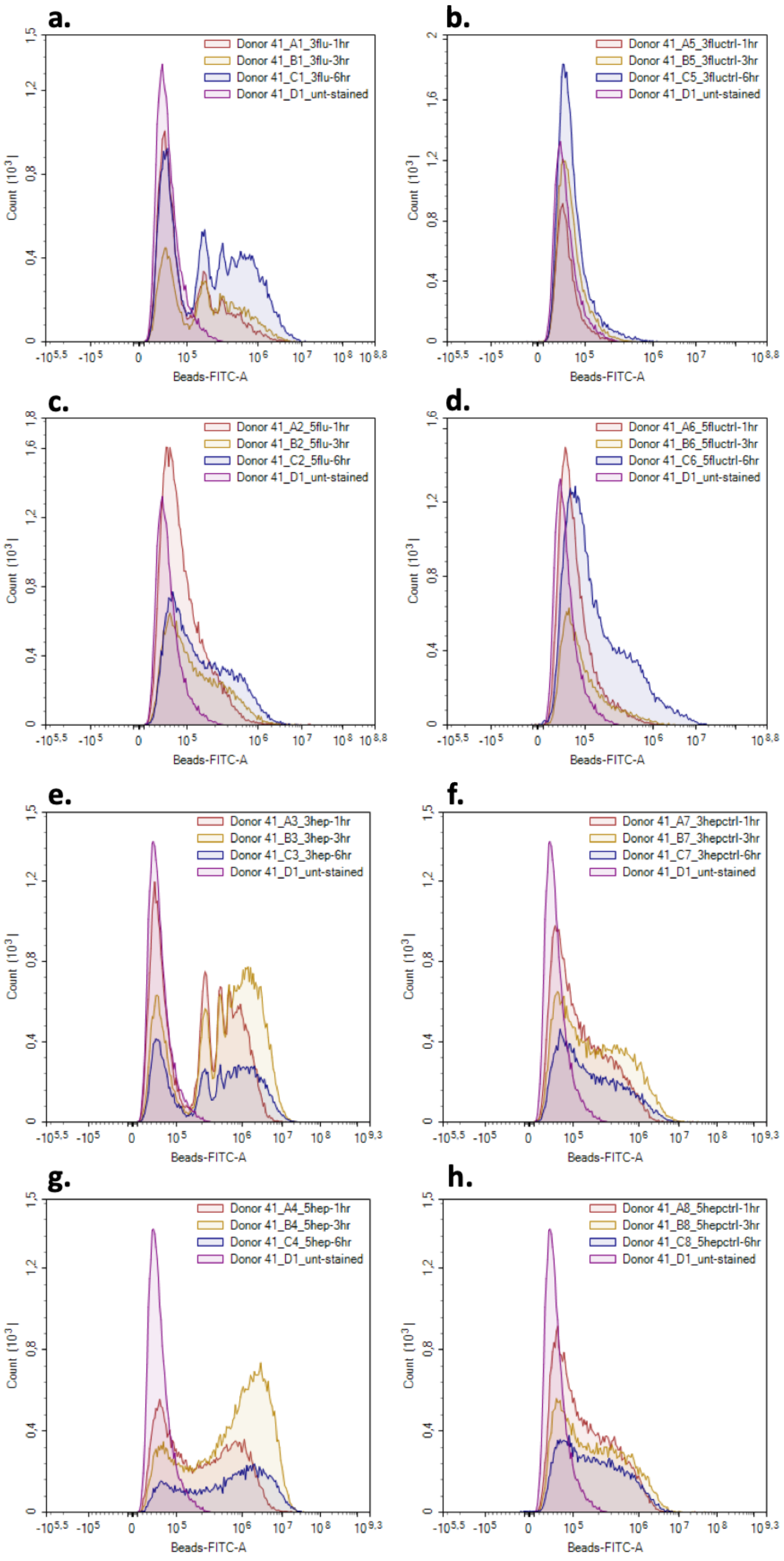
a.
Donor 41_A1_3flu-1hr
Donor 41_B1_3flu-3hr
Donor 41_C1_3flu-6hr
Donor 41_D1_unt-stained
Count [10^3]
Beads-FITC-A
b.
Donor 41_A5_3fluctrl-1hr
Donor 41_B5_3fluctrl-3hr
Donor 41_C5_3fluctrl-6hr
Donor 41_D1_unt-stained
Count [10^3]
Beads-FITC-A
c.
Donor 41_A2_5flu-1hr
Donor 41_B2_5flu-3hr
Donor 41_C2_5flu-6hr
Donor 41_D1_unt-stained
Count [10^3]
Beads-FITC-A
d.
Donor 41_A6_5fluctrl-1hr
Donor 41_B6_5fluctrl-3hr
Donor 41_C6_5fluctrl-6hr
Donor 41_D1_unt-stained
Count [10^3]
Beads-FITC-A
e.
Donor 41_A3_3hep-1hr
Donor 41_B3_3hep-3hr
Donor 41_C3_3hep-6hr
Donor 41_D1_unt-stained
Count [10^3]
Beads-FITC-A
f.
Donor 41_A7_3hepctrl-1hr
Donor 41_B7_3hepctrl-3hr
Donor 41_C7_3hepctrl-6hr
Donor 41_D1_unt-stained
Count [10^3]
Beads-FITC-A
g.
Donor 41_A4_5hep-1hr
Donor 41_B4_5hep-3hr
Donor 41_C4_5hep-6hr
Donor 41_D1_unt-stained
Count [10^3]
Beads-FITC-A
h.
Donor 41_A8_5hepctrl-1hr
Donor 41_B8_5hepctrl-3hr
Donor 41_C8_5hepctrl-6hr
Donor 41_D1_unt-stained
Count [10^3]
Beads-FITC-A

Supplementary Figure S2: Fluorescence distributions over time of one representative donor used in the uptake experiments. Different colours show different time-points as depicted in the figure key. The purple graph represents the untreated control condition and resembles the FITC-negative population. (**a**) Cells stimulated with 3 μm influenza subunit vaccine conjugates versus untreated cells. (**b**) Cells stimulated with the amount of unconjugated vaccine similar to the amount bound to the 3 μm influenza subunit vaccine conjugates versus untreated cells. (**c**) Cells stimulated with 500 nm influenza subunit vaccine conjugates versus untreated cells. (**d**) Cells stimulated with the amount of unconjugated vaccine similar to the amount bound to the 500 nm influenza subunit vaccine conjugates versus untreated cells. (**e**) Cells stimulated with 3 μm hepatitis B subunit vaccine conjugates versus untreated cells. (**f**) Cells stimulated with the amount of unconjugated vaccine similar to the amount bound to the 3 μm hepatitis B subunit vaccine conjugates versus untreated cells. (**g**) Cells stimulated with 500 nm hepatitis B subunit vaccine conjugates vesus untreated cells. (**h**) Cells stimulated with the amount of unconjugated vaccine similar to the amount bound to the 500 nm hepatitis B subunit vaccine conjugates versus untreated cells.
