# Supplementary material for: Assessing the Immunomodulatory Effect of Size on the Uptake and Immunogenicity of Influenza- and Hepatitis B Subunit Vaccines In Vitro
Source: Pharmaceuticals (Basel). 2022 Jul 18;15(7):887. doi: 10.3390/ph15070887 (PMC9321264; doi:10.3390/ph15070887)
Supplement: Supplementary file 1 [file pharmaceuticals-15-00887-s001.zip › Supplementary file S1.pdf]

Report of Donor 31

Specimen Name: Donor 31 | Run Time: 26-1-2022 10:53

Cytometer: NovoCyte Quanteon 621200611403 | Software: NovoExpress 1.5.6

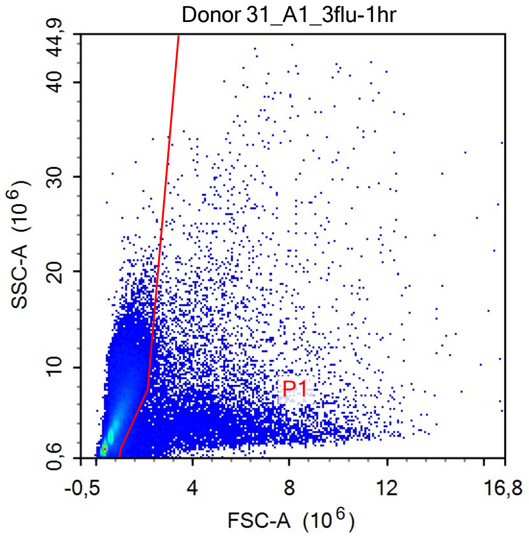

Gate	Count	% All
All	347.055	100,00%
P1	12.261	3,53%

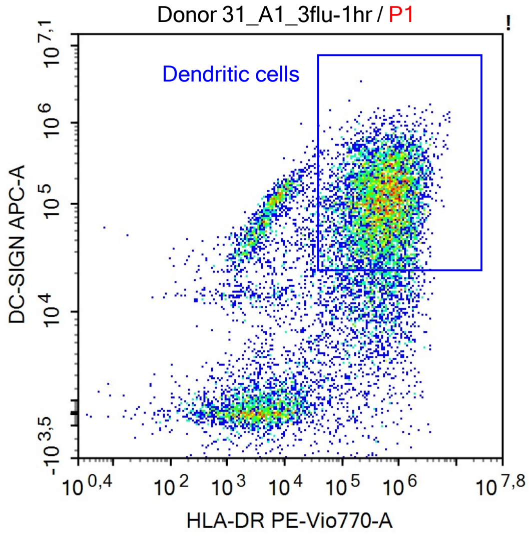

Gate	Count	% P1
P1	12.261	100,00%
Dendritic cells	6.237	50,87%

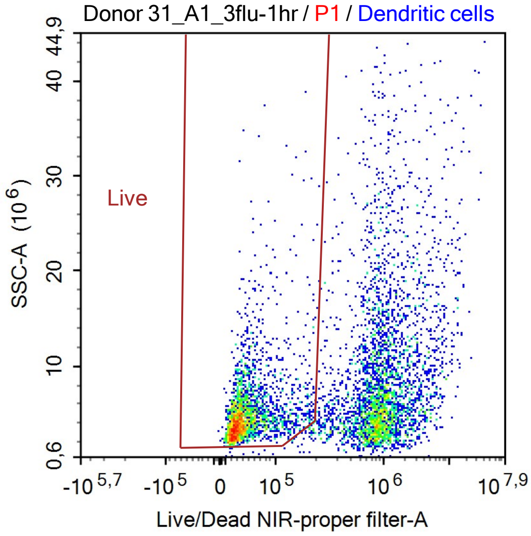

Gate	Count	% Dendritic cells
Dendritic cells	6.237	100,00%
Live	2.712	43,48%

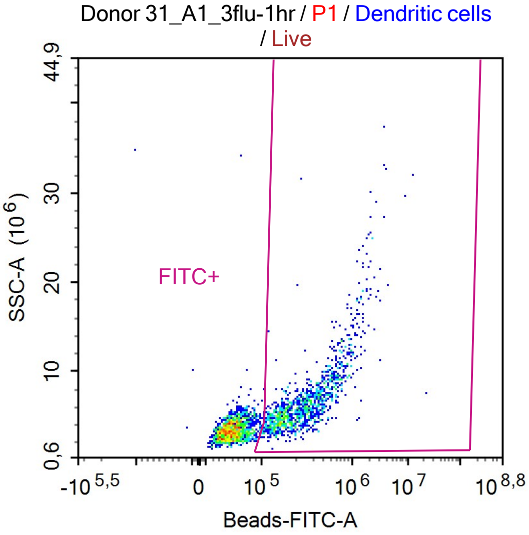

Gate	Count	% Live
Live	2.712	100,00%
FITC+	1.298	47,86%

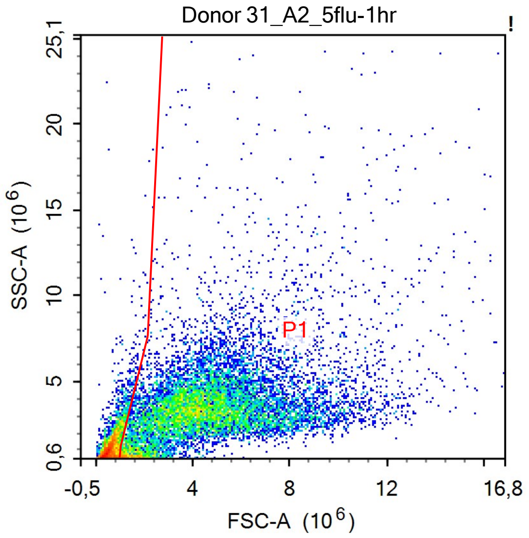

Gate	Count	% All
All	42.558	100,00%
P1	15.942	37,46%

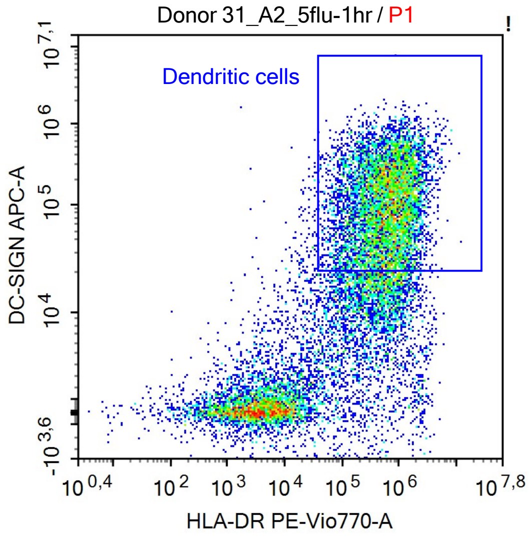

Gate	Count	% P1
P1	15.942	100,00%
Dendritic cells	7.228	45,34%

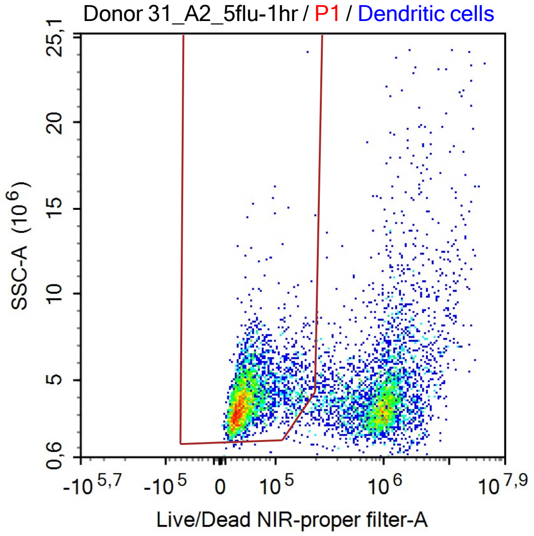

Gate	Count	% Dendritic cells
Dendritic cells	7.228	100,00%
Live	3.667	50,73%

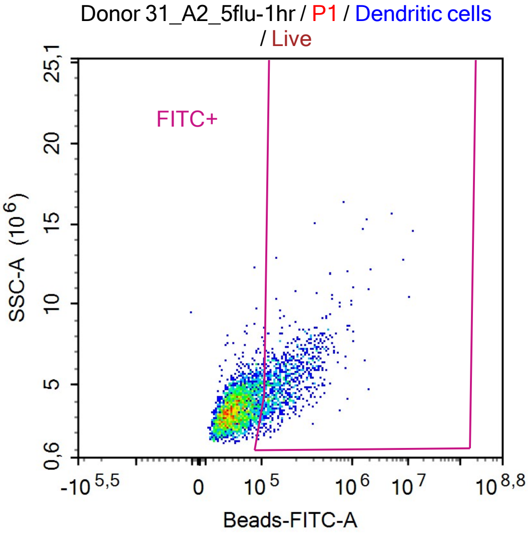

Gate	Count	% Live
Live	3.667	100,00%
FITC+	880	24,00%

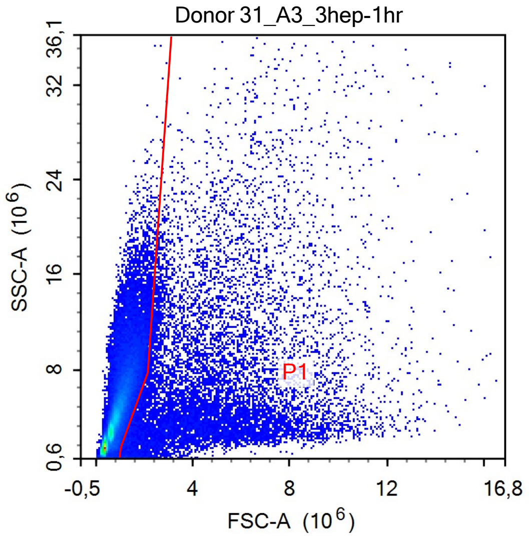

Gate	Count	% All
All	313.458	100,00%
P1	14.858	4,74%

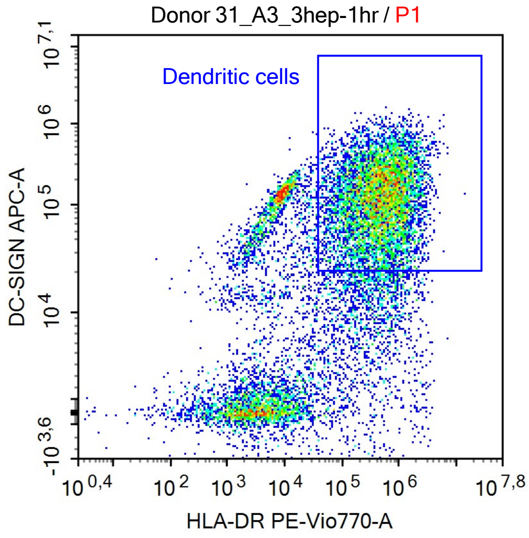

Gate	Count	% P1
P1	14.858	100,00%
Dendritic cells	7.441	50,08%

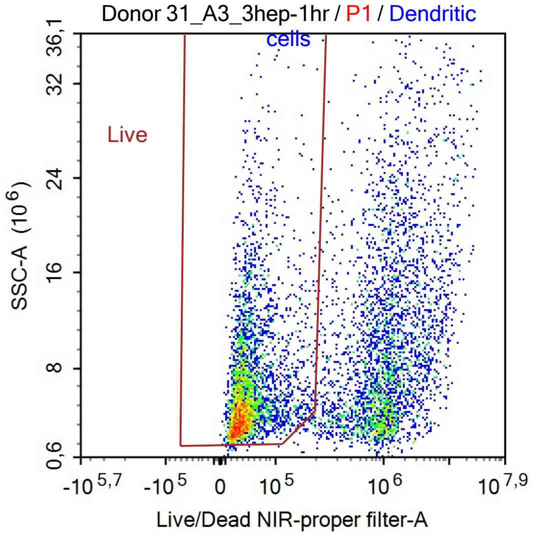

Gate	Count	% Dendritic cells
Dendritic cells	7.441	100,00%
Live	3.874	52,06%

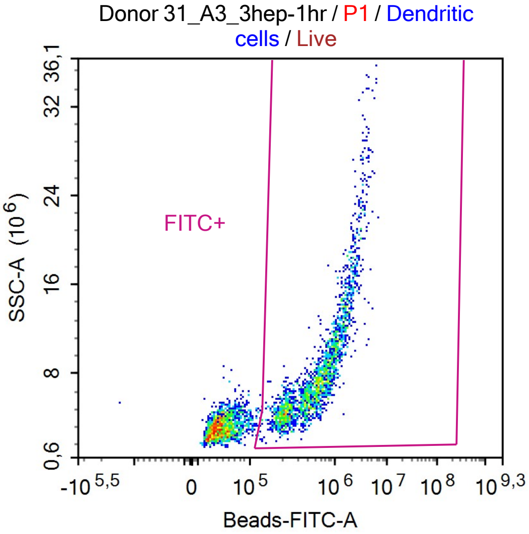

Gate	Count	% Live
Live	3.874	100,00%
FITC+	2.423	62,55%

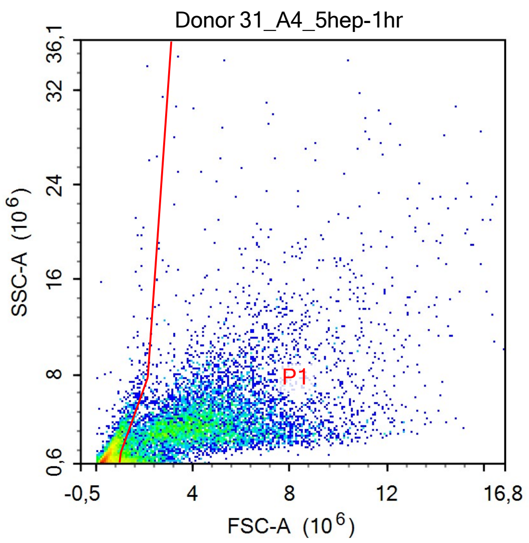

Gate	Count	% All
All	34.394	100,00%
P1	9.887	28,75%

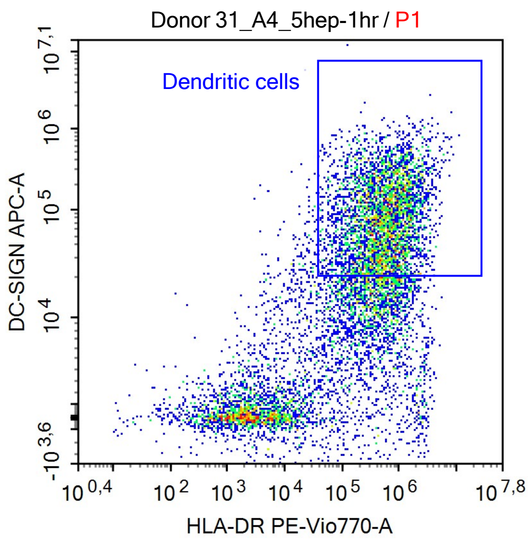

Gate	Count	% P1
P1	9.887	100,00%
Dendritic cells	4.230	42,78%

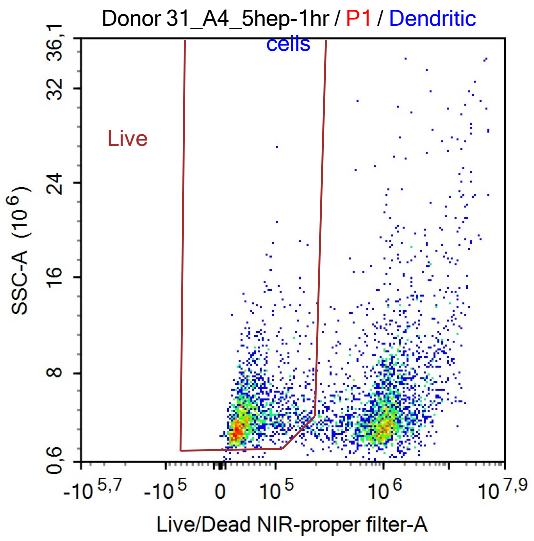

Gate	Count	% Dendritic cells
Dendritic cells	4.230	100,00%
Live	1.757	41,54%

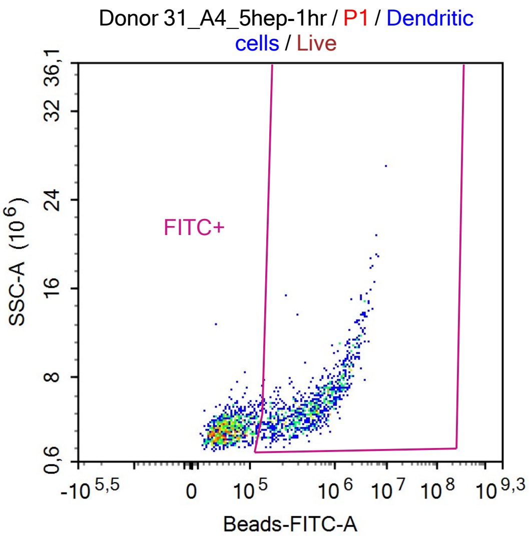

Gate	Count	% Live
Live	1.757	100,00%
FITC+	867	49,35%

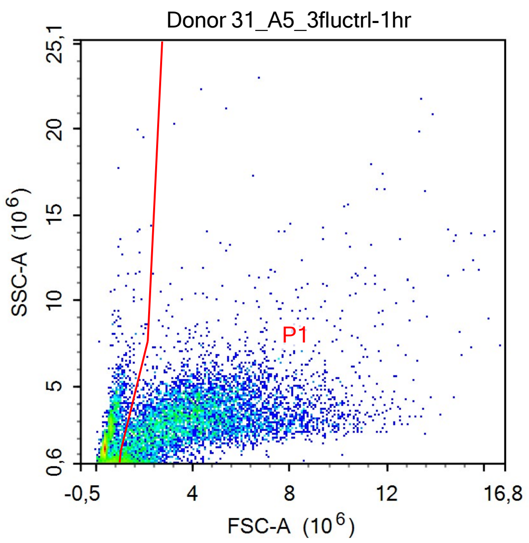

Gate	Count	% All
All	32.586	100,00%
P1	9.361	28,73%

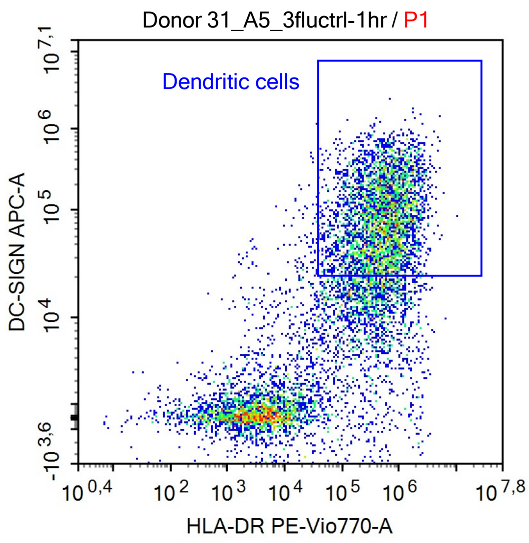

Gate	Count	% P1
P1	9.361	100,00%
Dendritic cells	3.881	41,46%

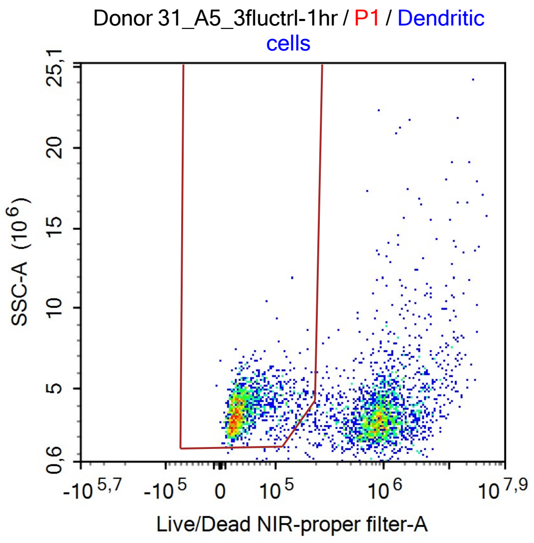

Gate	Count	% Dendritic cells
Dendritic cells	3.881	100,00%
Live	1.654	42,62%

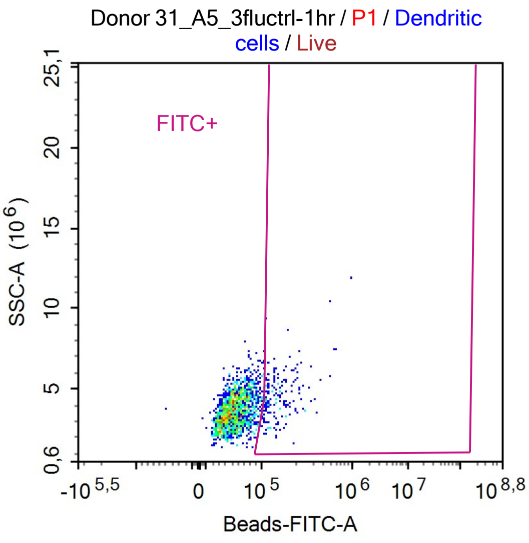

Gate	Count	% Live
Live	1.654	100,00%
FITC+	165	9,98%

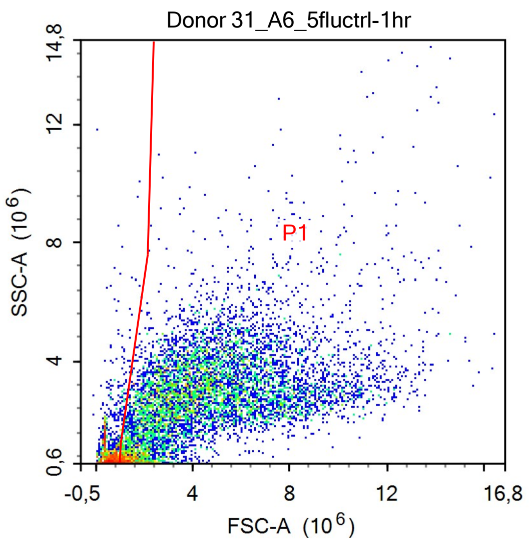

Gate	Count	% All
All	33.577	100,00%
P1	12.458	37,10%

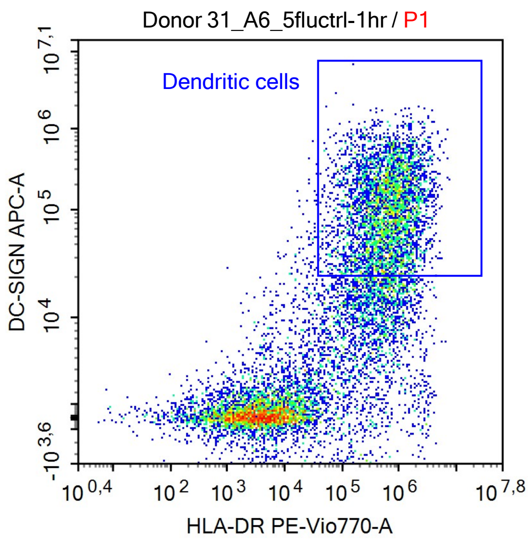

Gate	Count	% P1
P1	12.458	100,00%
Dendritic cells	4.133	33,18%

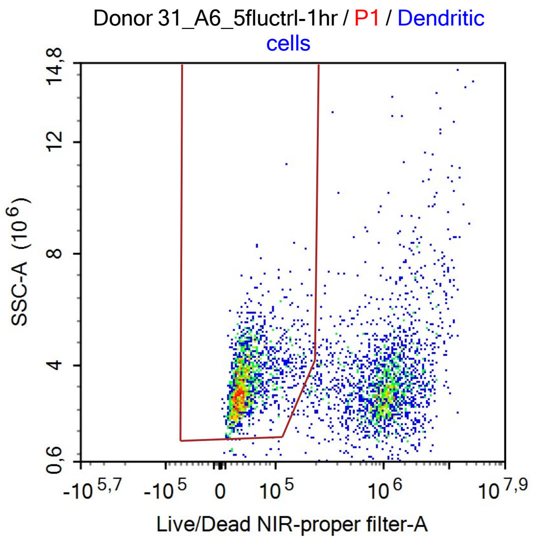

Gate	Count	% Dendritic cells
Dendritic cells	4.133	100,00%
Live	2.022	48,92%

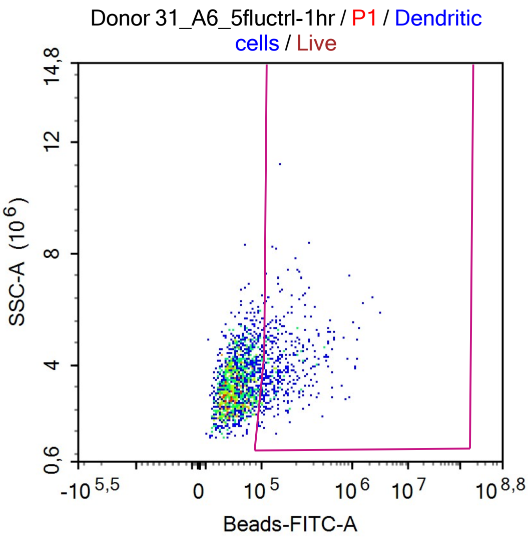

Gate	Count	% Live
Live	2.022	100,00%
FITC+	389	19,24%

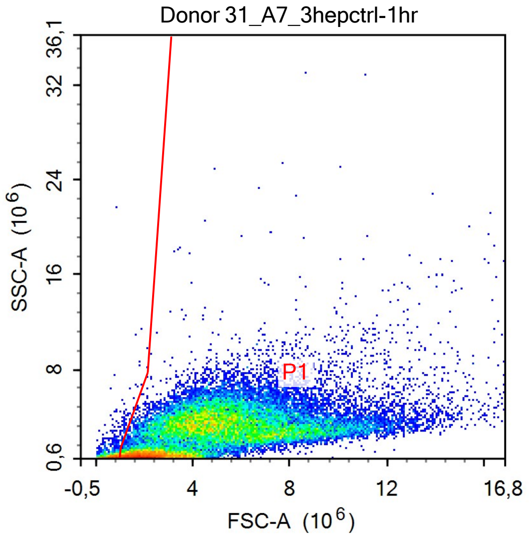

Gate	Count	% All
All	80.529	100,00%
P1	44.120	54,79%

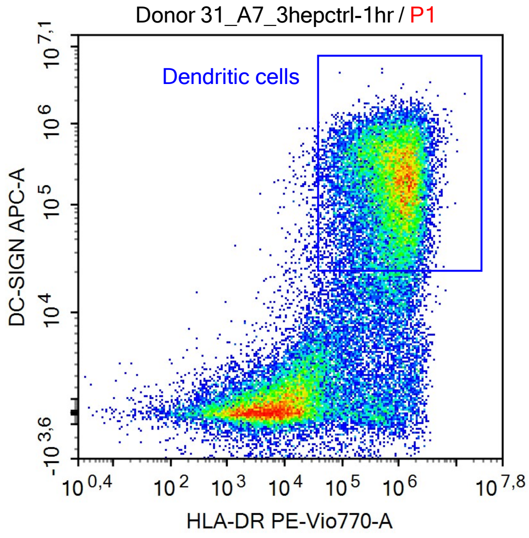

Gate	Count	% P1
P1	44.120	100,00%
Dendritic cells	18.451	41,82%

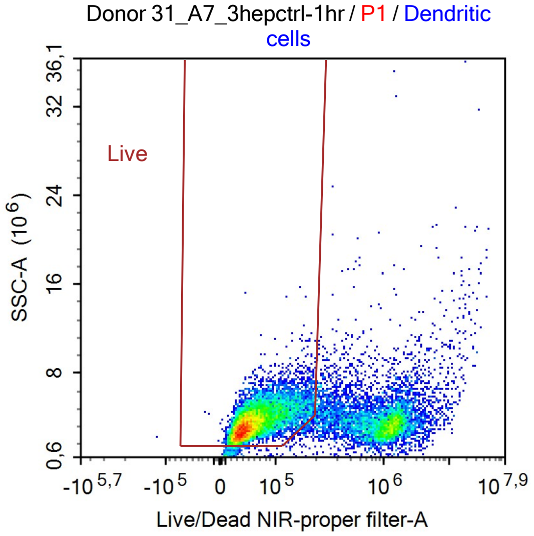

Gate	Count	% Dendritic cells
Dendritic cells	18.451	100,00%
Live	11.733	63,59%

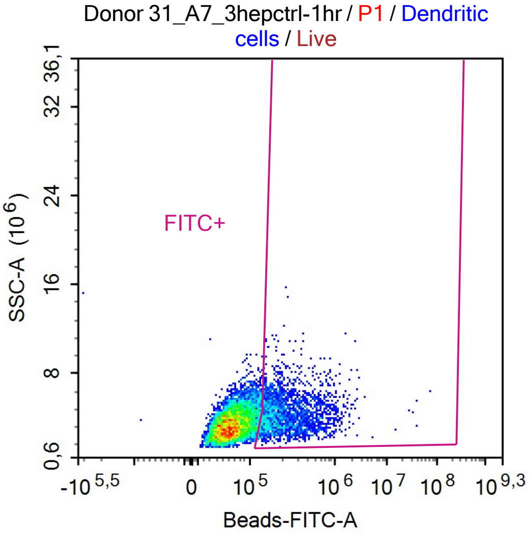

Gate	Count	% Live
Live	11.733	100,00%
FITC+	2.261	19,27%

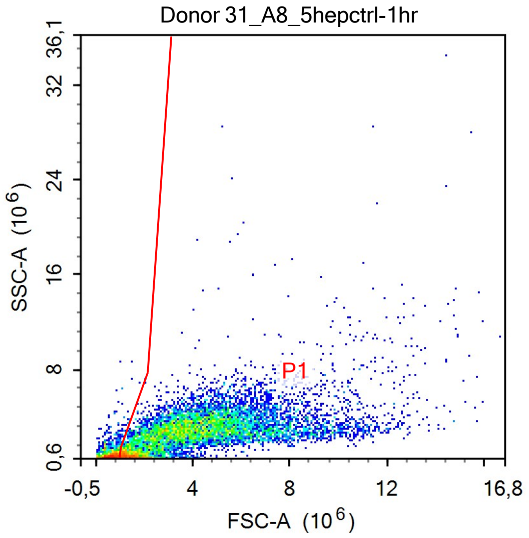

Gate	Count	% All
All	32.843	100,00%
P1	11.805	35,94%

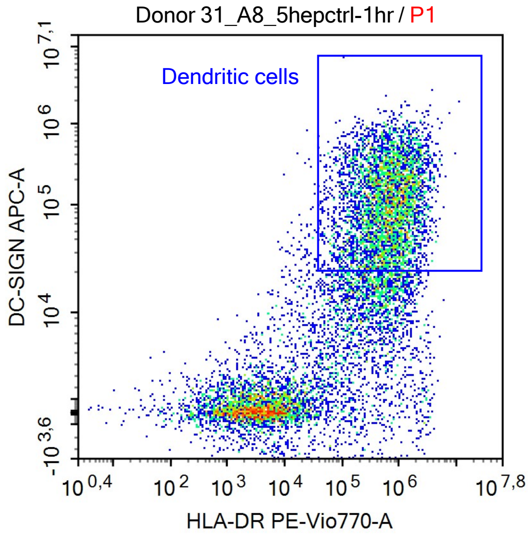

Gate	Count	% P1
P1	11.805	100,00%
Dendritic cells	4.566	38,68%

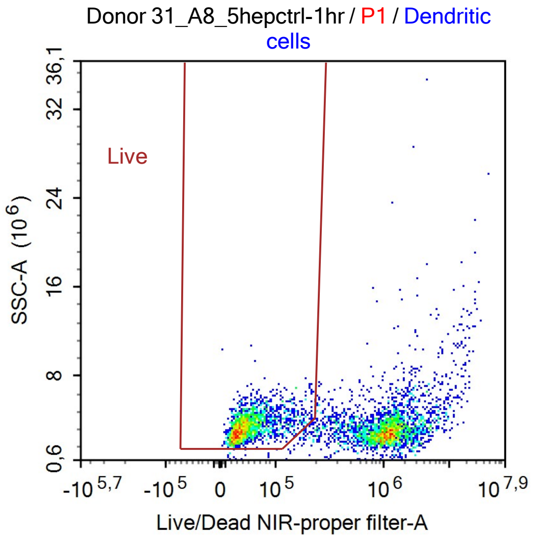

Gate	Count	% Dendritic cells
Dendritic cells	4.566	100,00%
Live	1.903	41,68%

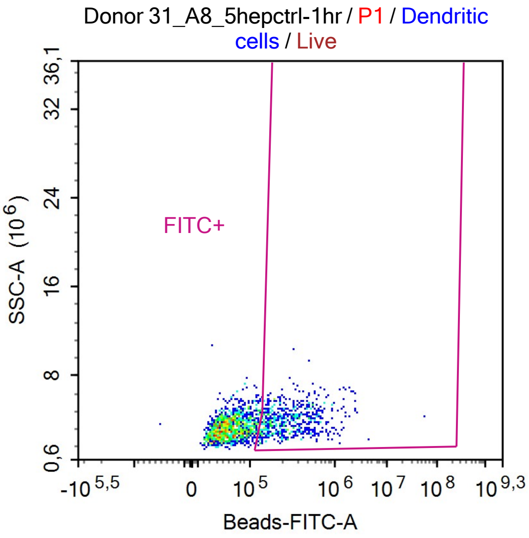

Gate	Count	% Live
Live	1.903	100,00%
FITC+	549	28,85%

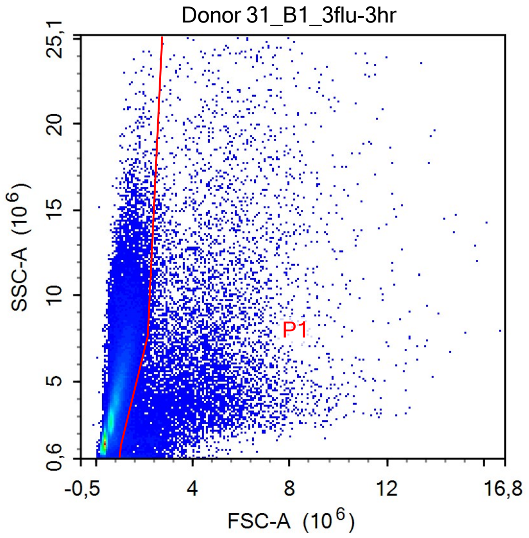

Gate	Count	% All
All	399.998	100,00%
P1	18.216	4,55%

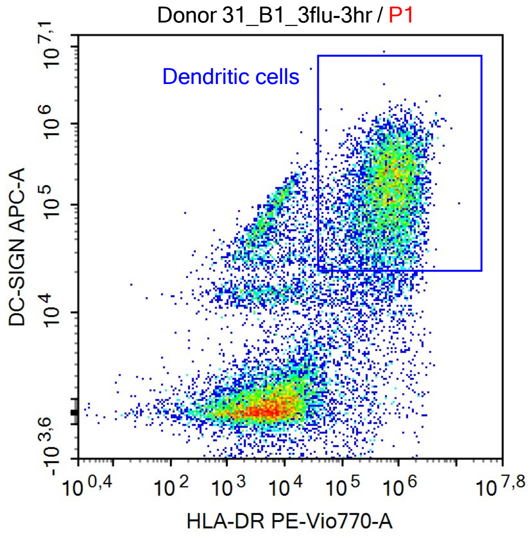

Gate	Count	% P1
P1	18.216	100,00%
Dendritic cells	6.116	33,57%

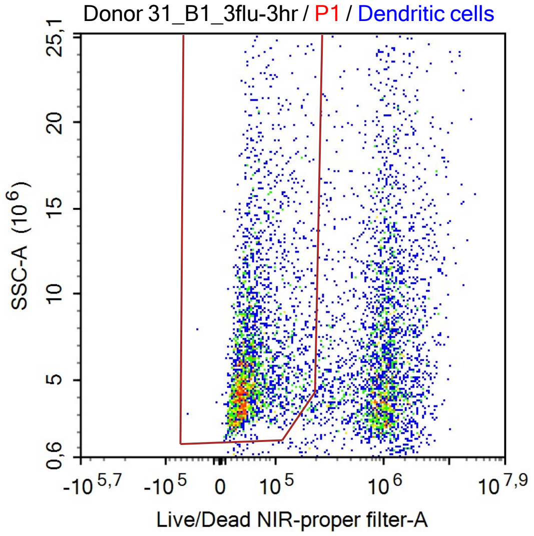

Gate	Count	% Dendritic cells
Dendritic cells	6.116	100,00%
Live	2.944	48,14%

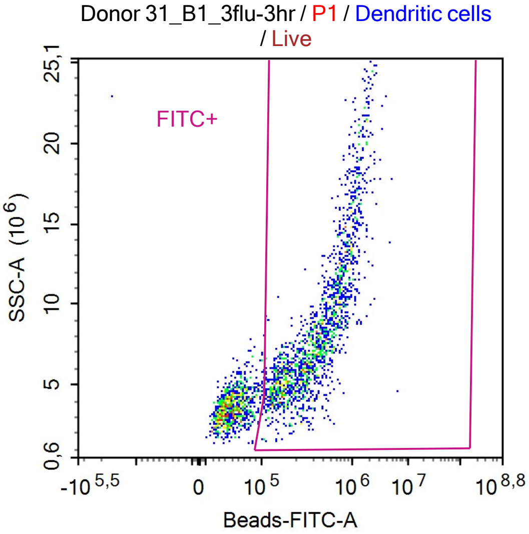

Gate	Count	% Live
Live	2.944	100,00%
FITC+	2.018	68,55%

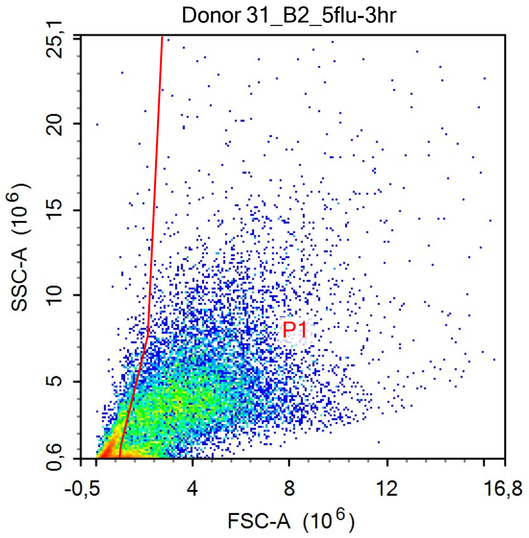

Gate	Count	% All
All	65.707	100,00%
P1	25.179	38,32%

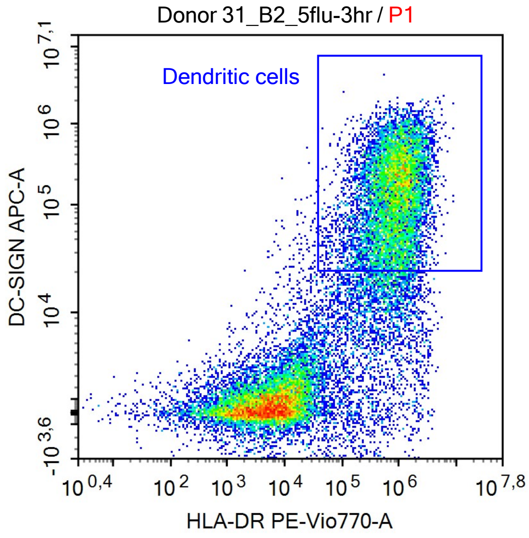

Gate	Count	% P1
P1	25.179	100,00%
Dendritic cells	9.342	37,10%

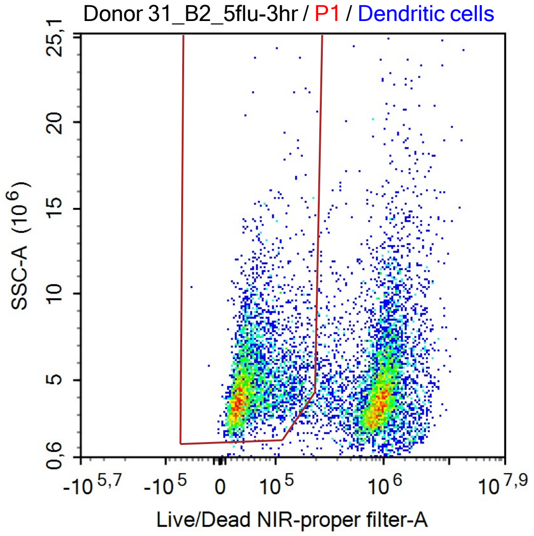

Gate	Count	% Dendritic cells
Dendritic cells	9.342	100,00%
Live	4.055	43,41%

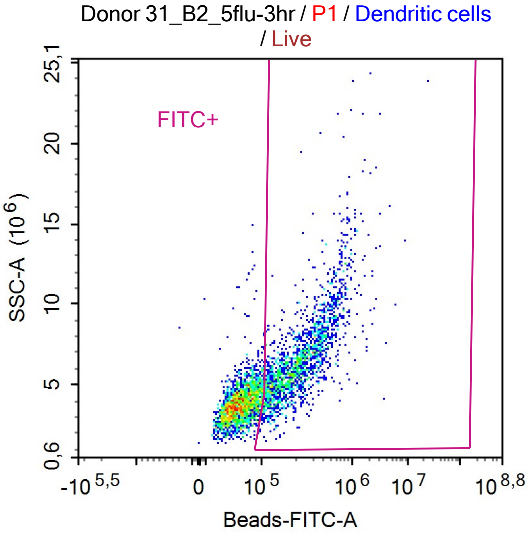

Gate	Count	% Live
Live	4.055	100,00%
FITC+	1.837	45,30%

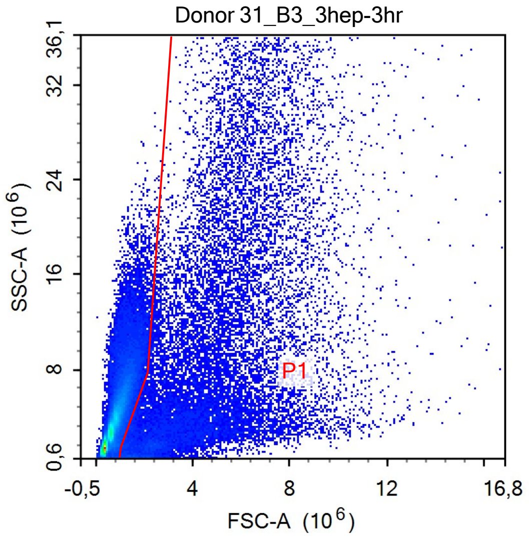

Gate	Count	% All
All	362.506	100,00%
P1	36.556	10,08%

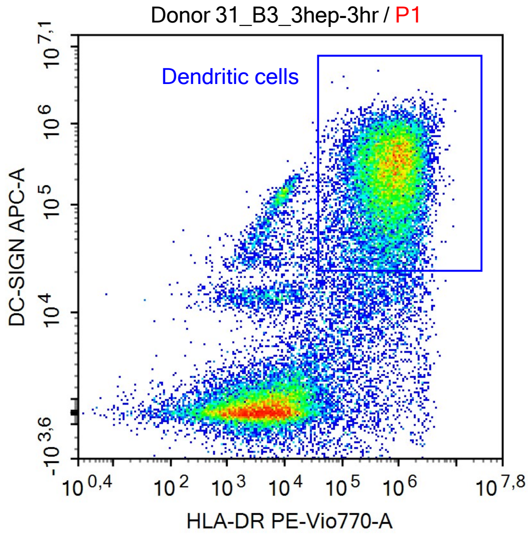

Gate	Count	% P1
P1	36.556	100,00%
Dendritic cells	15.553	42,55%

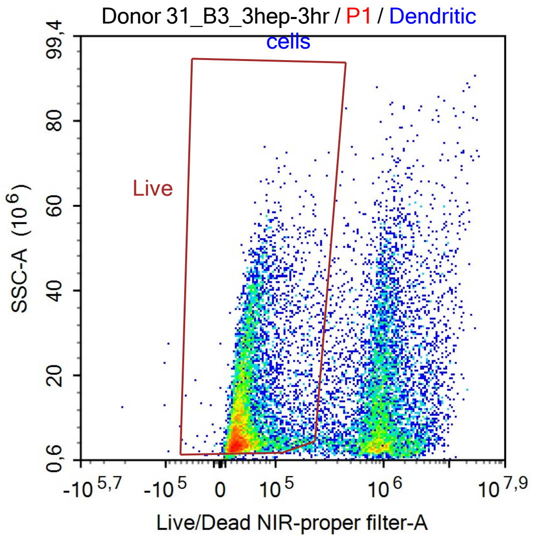

Gate	Count	% Dendritic cells
Dendritic cells	15.553	100,00%
Live	8.640	55,55%

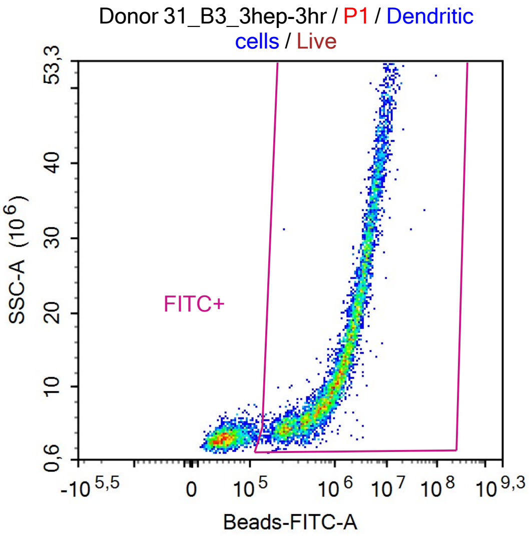

Gate	Count	% Live
Live	8.640	100,00%
FITC+	6.882	79,65%

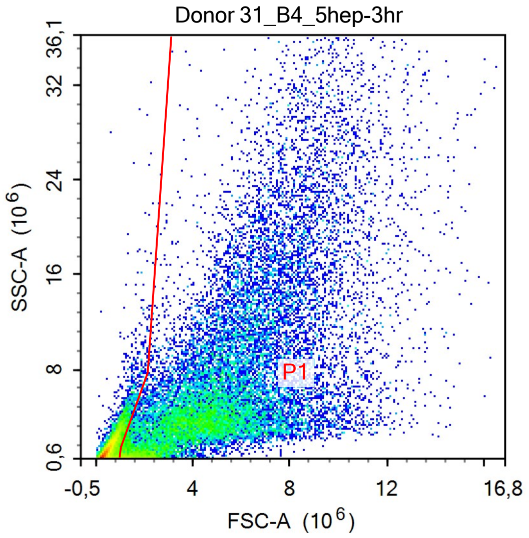

Gate	Count	% All
All	90.156	100,00%
P1	32.177	35,69%

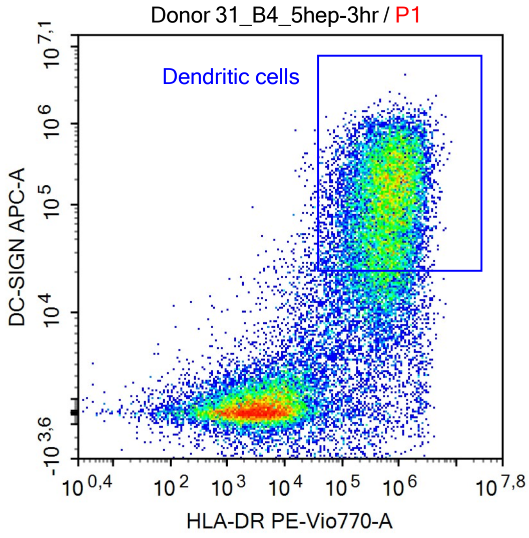

Gate	Count	% P1
P1	32.177	100,00%
Dendritic cells	12.870	40,00%

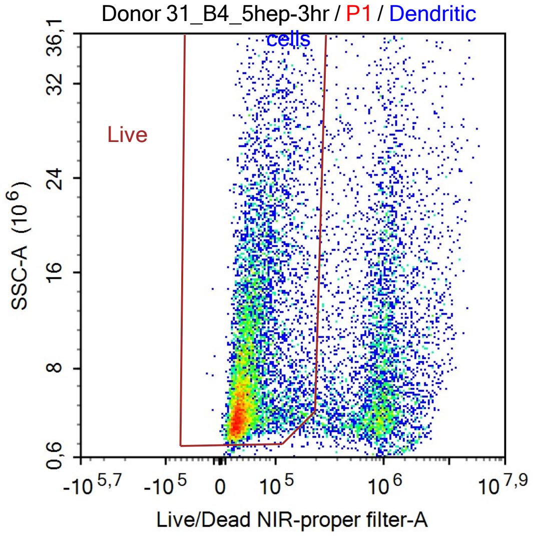

Gate	Count	% Dendritic cells
Dendritic cells	12.870	100,00%
Live	8.257	64,16%

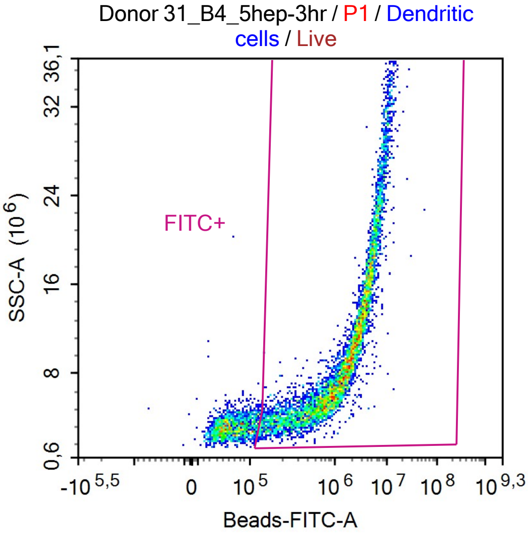

Gate	Count	% Live
Live	8.257	100,00%
FITC+	6.687	80,99%

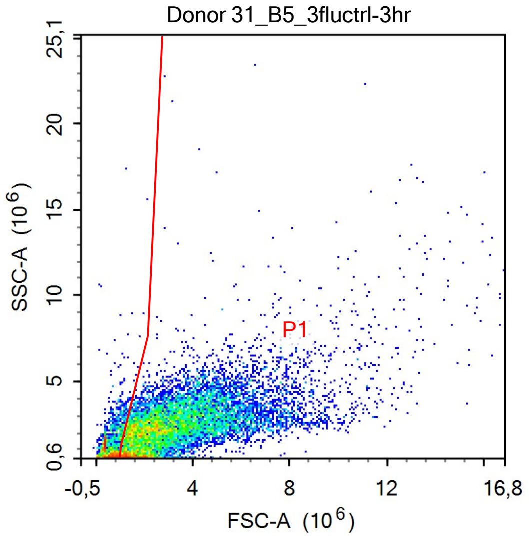

Gate	Count	% All
All	40.583	100,00%
P1	18.252	44,97%

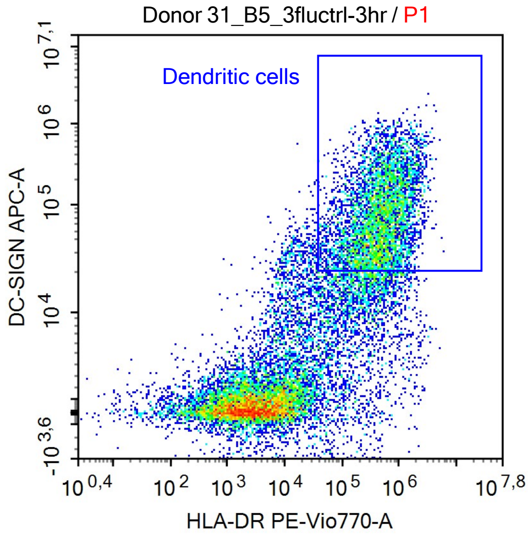

Gate	Count	% P1
P1	18.252	100,00%
Dendritic cells	5.268	28,86%

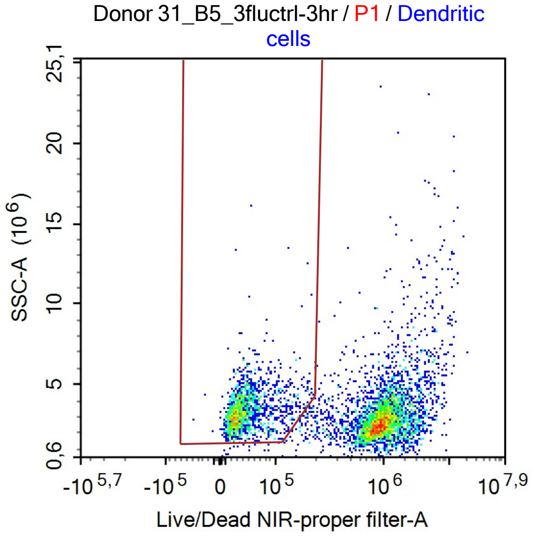

Gate	Count	% Dendritic cells
Dendritic cells	5.268	100,00%
Live	1.582	30,03%

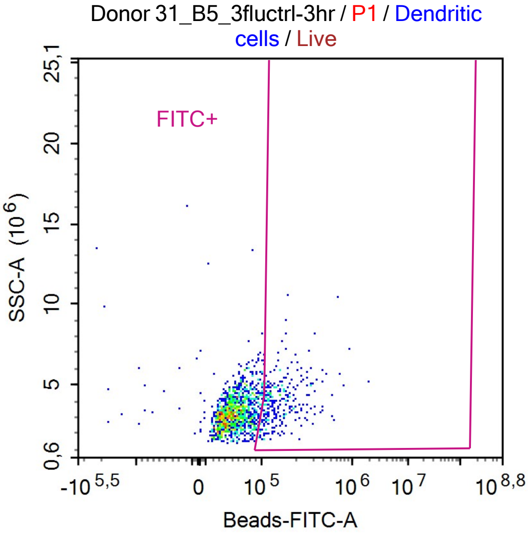

Gate	Count	% Live
Live	1.582	100,00%
FITC+	254	16,06%

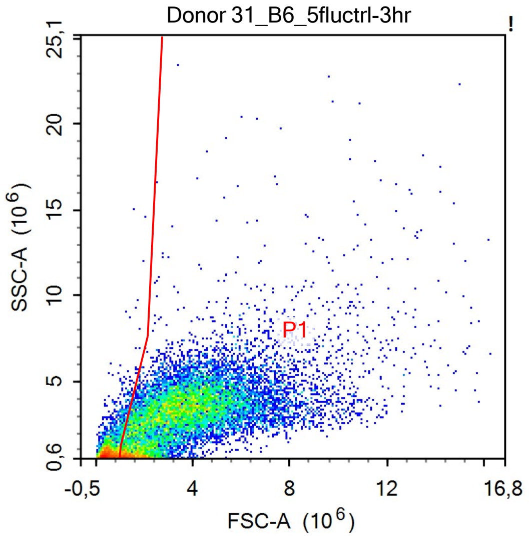

Gate	Count	% All
All	56.052	100,00%
P1	21.822	38,93%

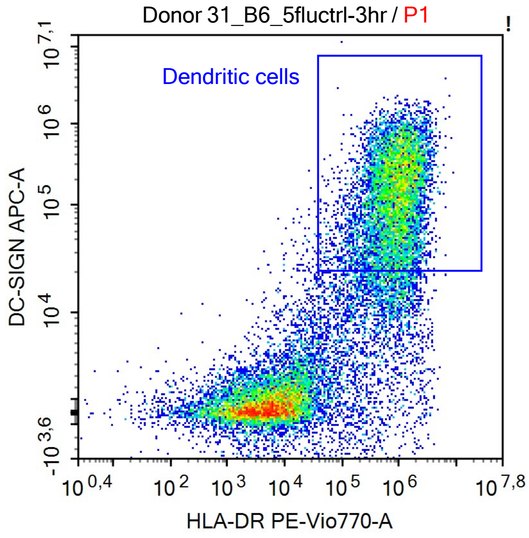

Gate	Count	% P1
P1	21.822	100,00%
Dendritic cells	8.150	37,35%

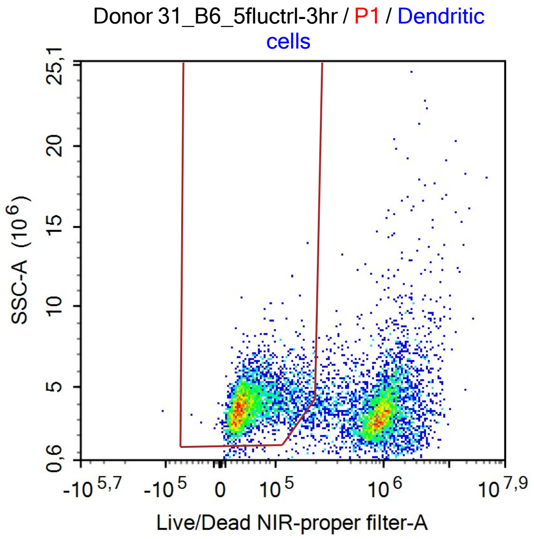

Gate	Count	% Dendritic cells
Dendritic cells	8.150	100,00%
Live	3.708	45,50%

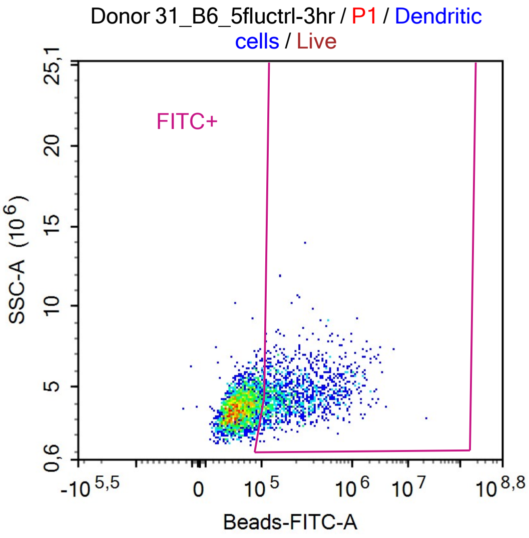

Gate	Count	% Live
Live	3.708	100,00%
FITC+	1.243	33,52%

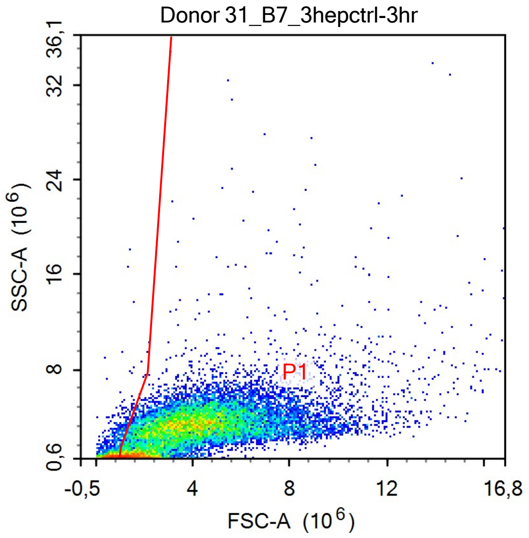

Gate	Count	% All
All	63.152	100,00%
P1	28.730	45,49%

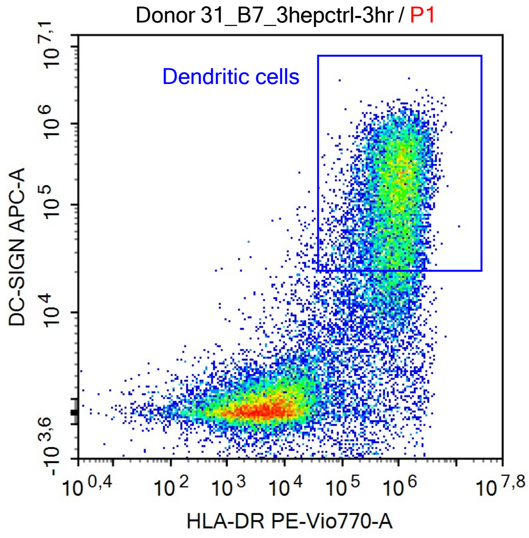

Gate	Count	% P1
P1	28.730	100,00%
Dendritic cells	9.664	33,64%

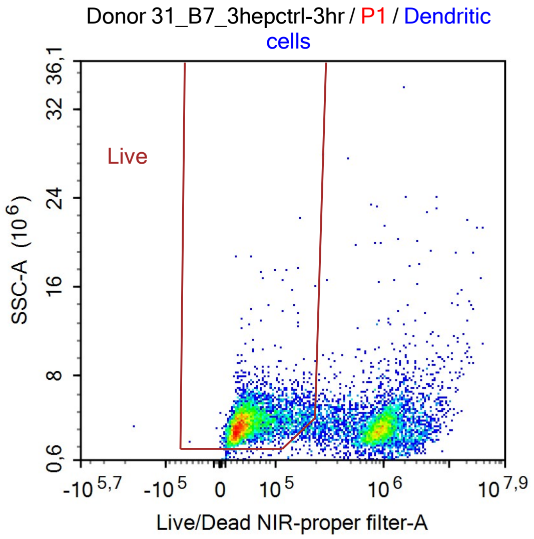

Gate	Count	% Dendritic cells
Dendritic cells	9.664	100,00%
Live	5.304	54,88%

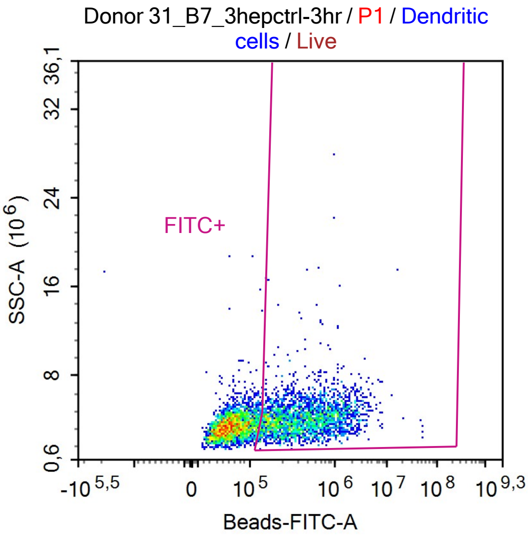

Gate	Count	% Live
Live	5.304	100,00%
FITC+	2.583	48,70%

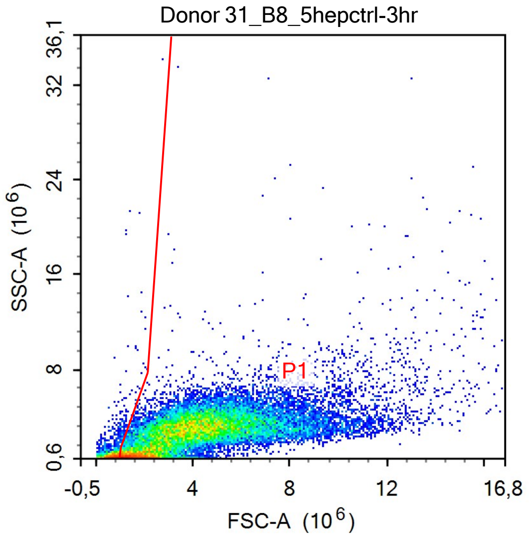

Gate	Count	% All
All	89.219	100,00%
P1	47.090	52,78%

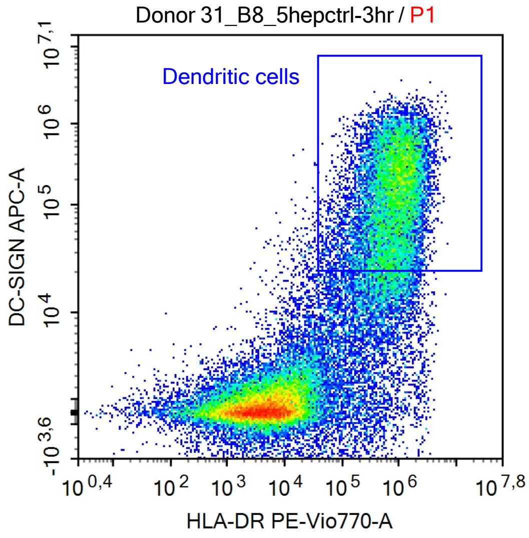

Gate	Count	% P1
P1	47.090	100,00%
Dendritic cells	13.406	28,47%

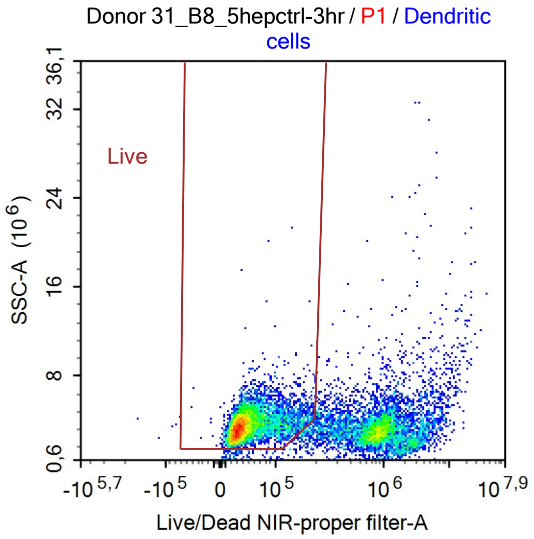

Gate	Count	% Dendritic cells
Dendritic cells	13.406	100,00%
Live	7.450	55,57%

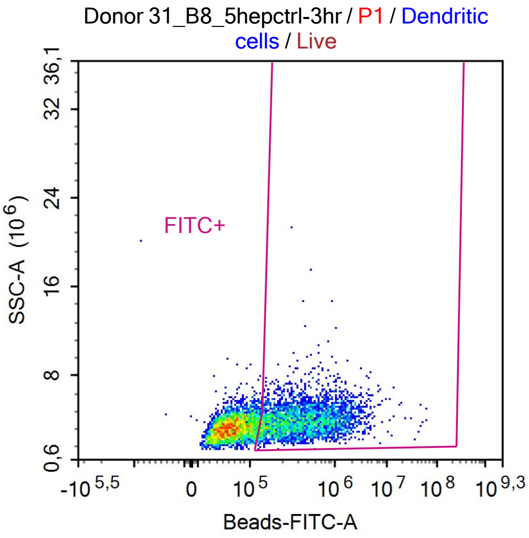

Gate	Count	% Live
Live	7.450	100,00%
FITC+	3.260	43,76%

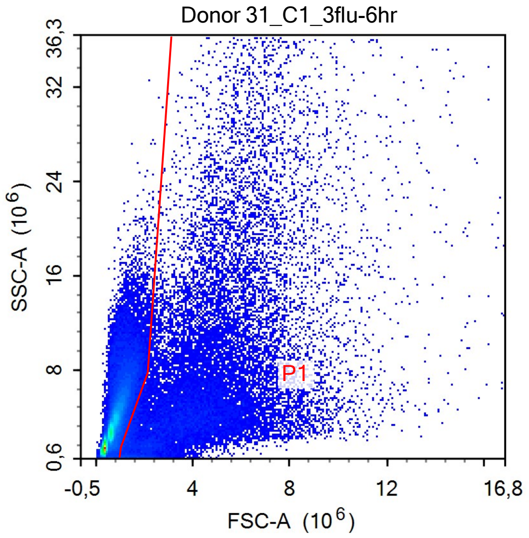

Gate	Count	% All
All	396.071	100,00%
P1	32.164	8,12%

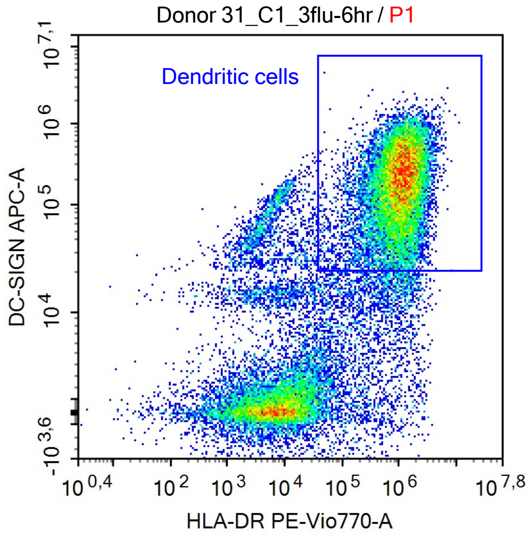

Gate	Count	% P1
P1	32.164	100,00%
Dendritic cells	16.006	49,76%

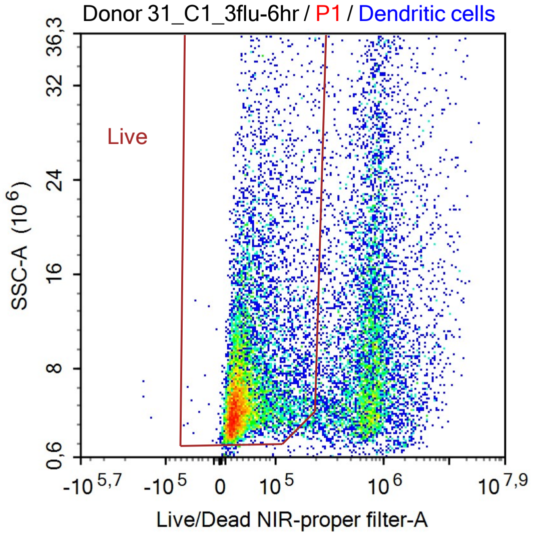

Gate	Count	% Dendritic cells
Dendritic cells	16.006	100,00%
Live	9.057	56,59%

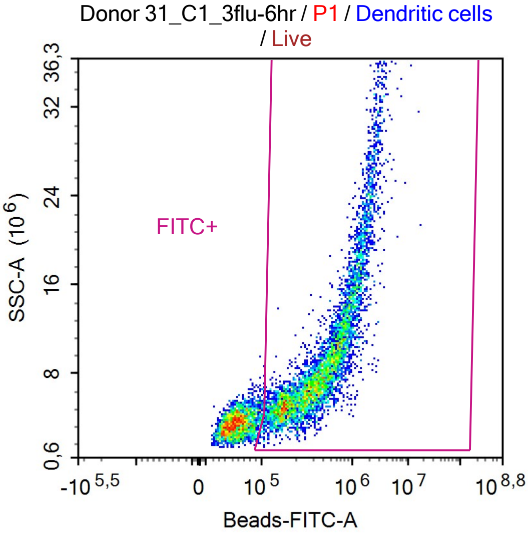

Gate	Count	% Live
Live	9.057	100,00%
FITC+	6.553	72,35%

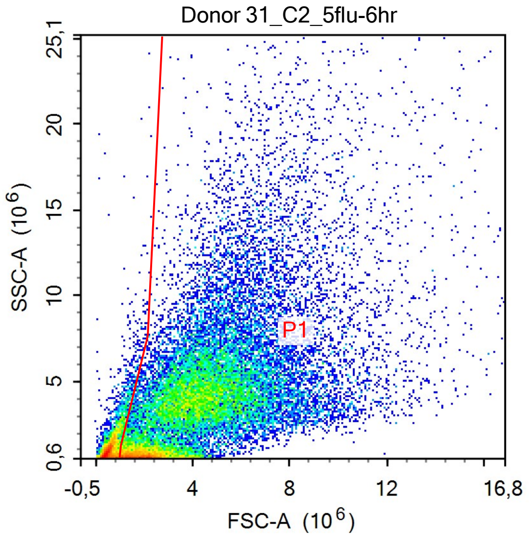

Gate	Count	% All
All	101.136	100,00%
P1	47.946	47,41%

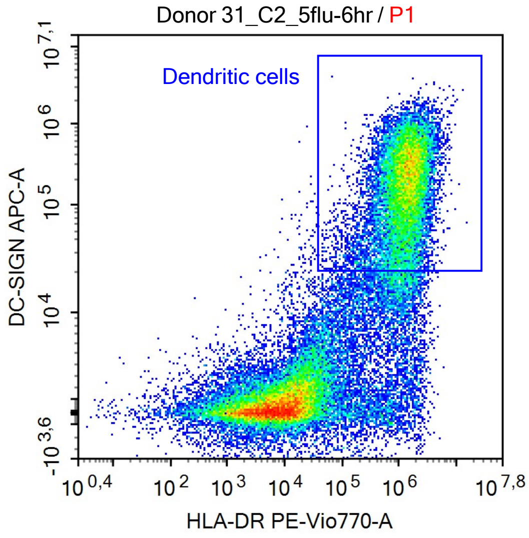

Gate	Count	% P1
P1	47.946	100,00%
Dendritic cells	15.823	33,00%

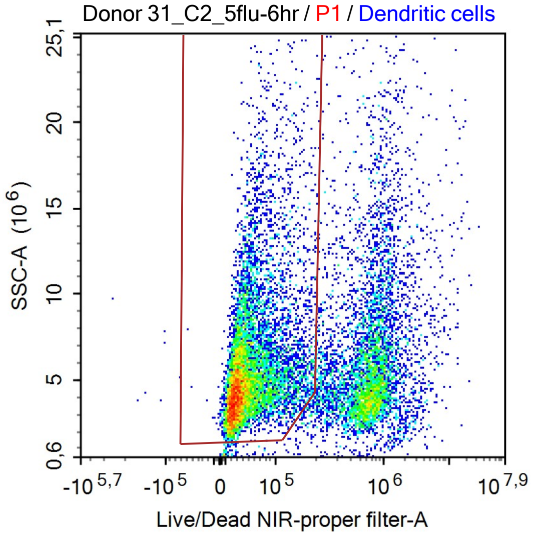

Gate	Count	% Dendritic cells
Dendritic cells	15.823	100,00%
Live	9.603	60,69%

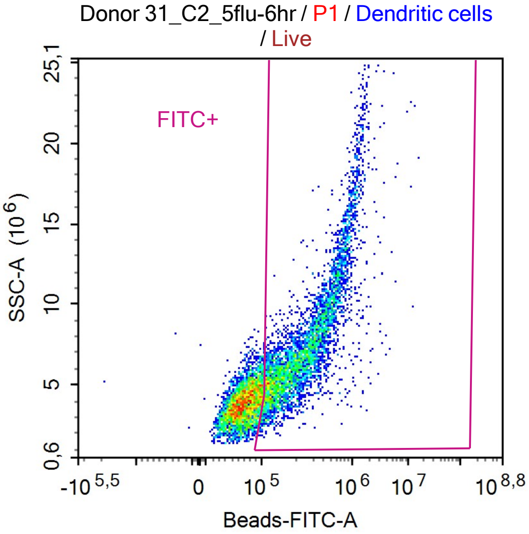

Gate	Count	% Live
Live	9.603	100,00%
FITC+	5.113	53,24%

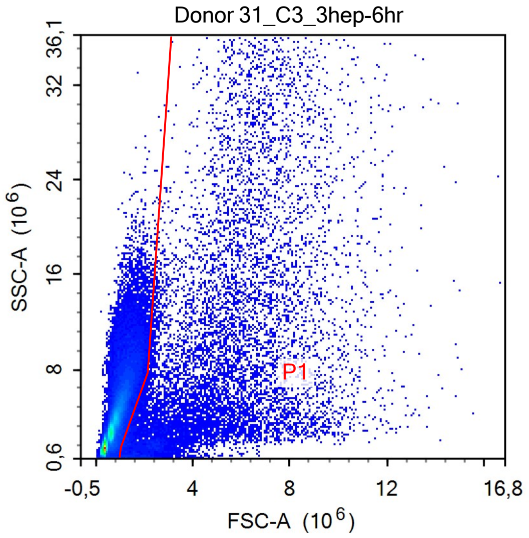

Gate	Count	% All
All	332.802	100,00%
P1	22.383	6,73%

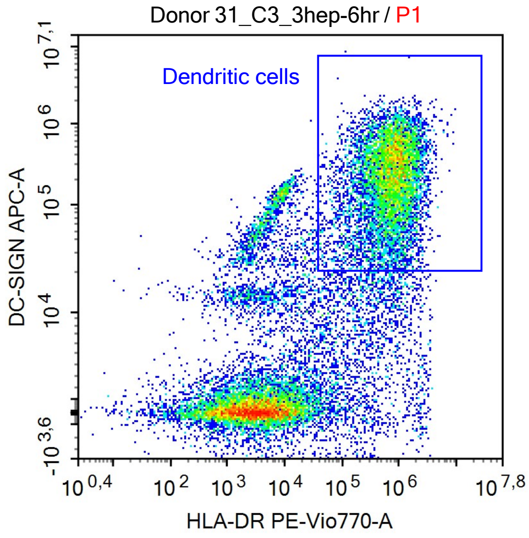

Gate	Count	% P1
P1	22.383	100,00%
Dendritic cells	7.925	35,41%

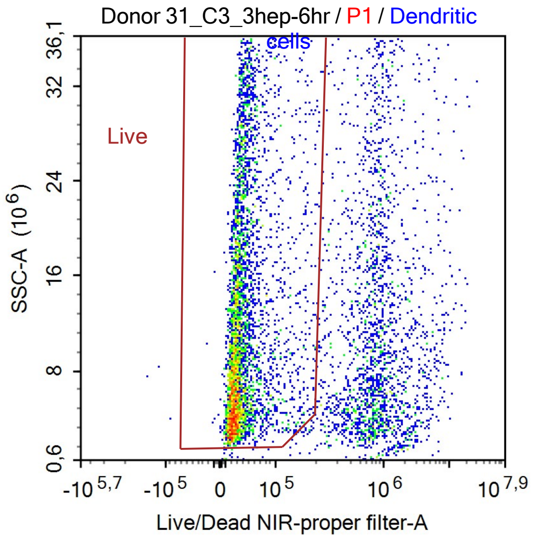

Gate	Count	% Dendritic cells
Dendritic cells	7.925	100,00%
Live	5.300	66,88%

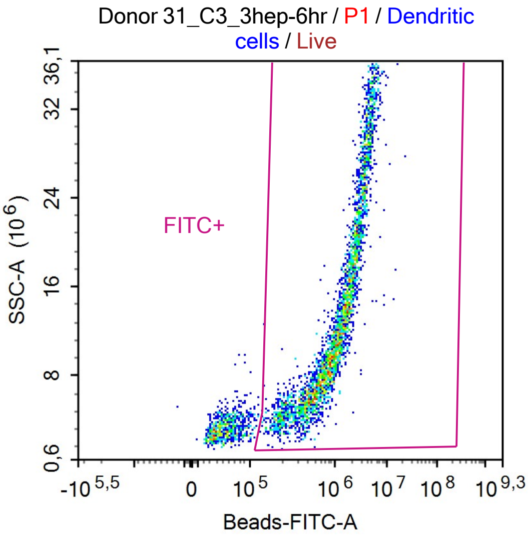

Gate	Count	% Live
Live	5.300	100,00%
FITC+	4.591	86,62%

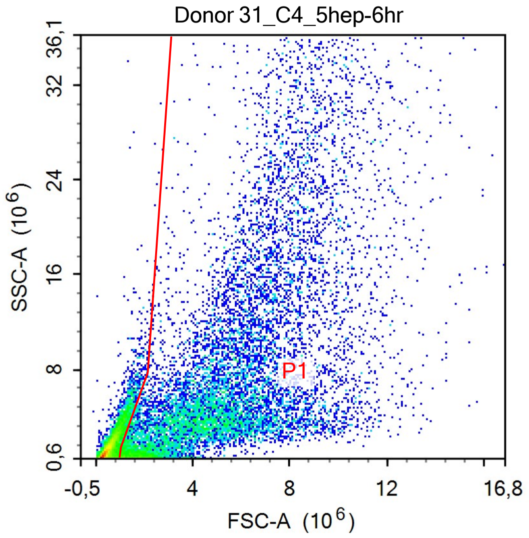

Gate	Count	% All
All	69.157	100,00%
P1	21.586	31,21%

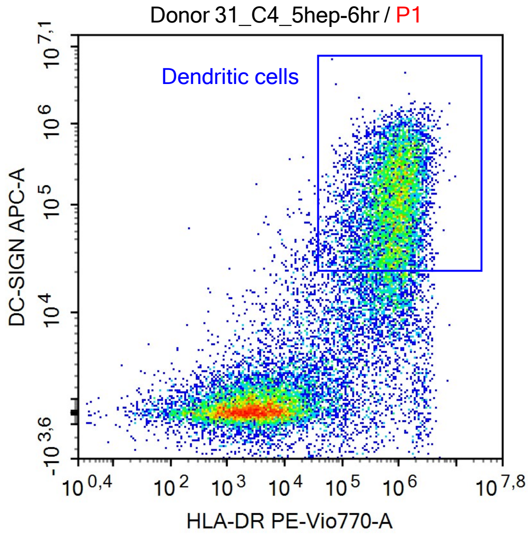

Gate	Count	% P1
P1	21.586	100,00%
Dendritic cells	7.317	33,90%

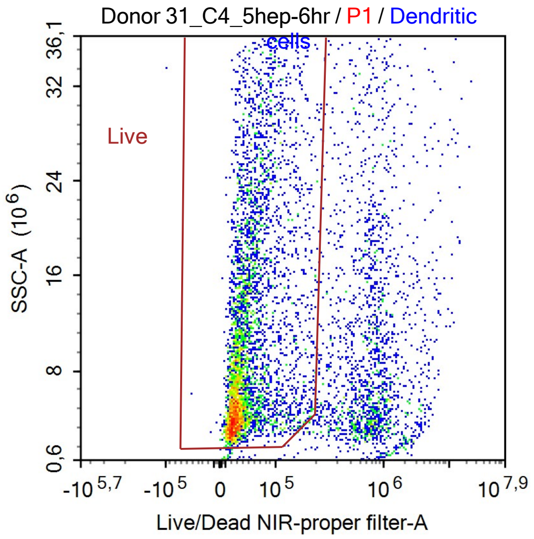

Gate	Count	% Dendritic cells
Dendritic cells	7.317	100,00%
Live	5.234	71,53%

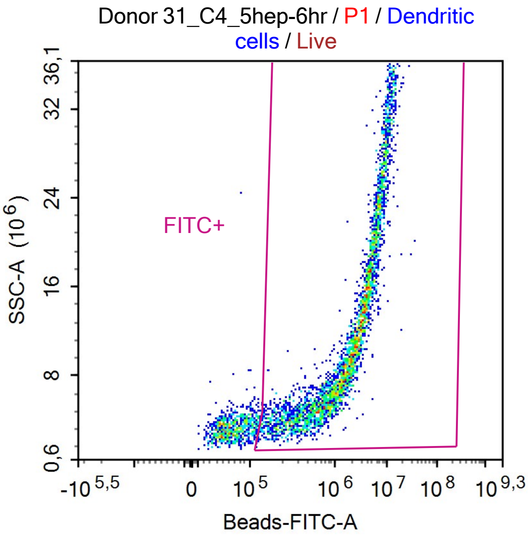

Gate	Count	% Live
Live	5.234	100,00%
FITC+	4.378	83,65%

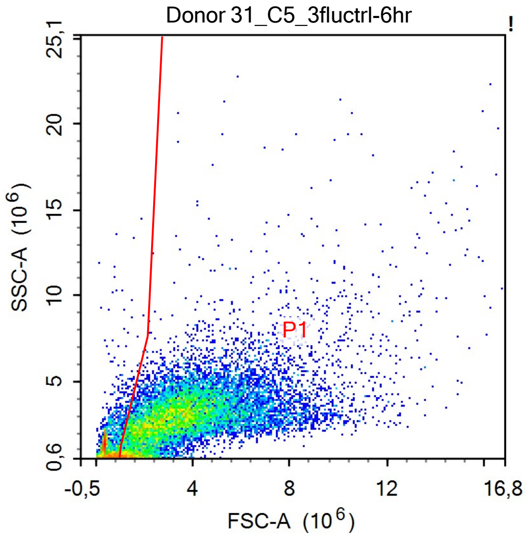

Gate	Count	% All
All	46.352	100,00%
P1	20.009	43,17%

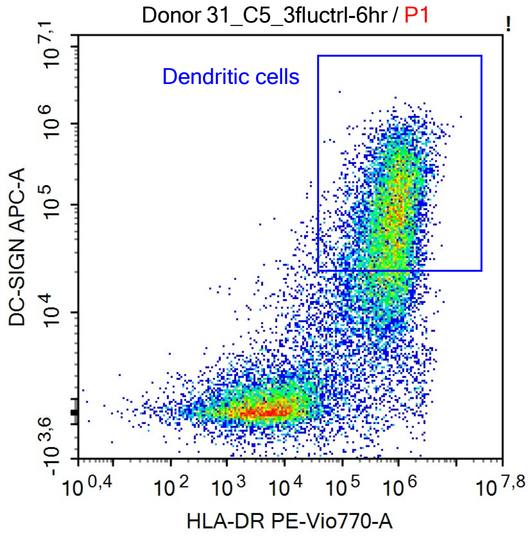

Gate	Count	% P1
P1	20.009	100,00%
Dendritic cells	7.930	39,63%

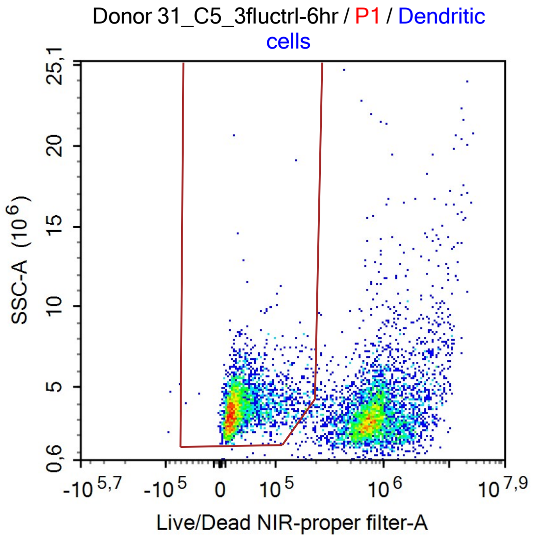

Gate	Count	% Dendritic cells
Dendritic cells	7.930	100,00%
Live	3.508	44,24%

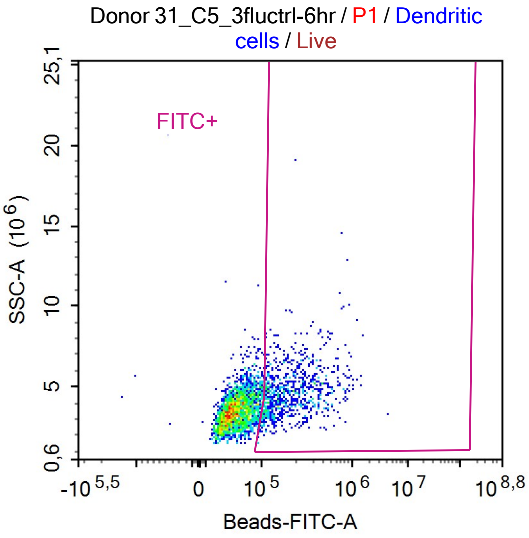

Gate	Count	% Live
Live	3.508	100,00%
FITC+	839	23,92%

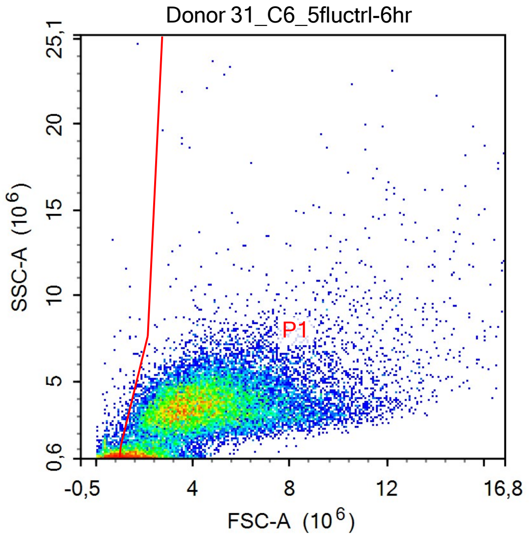

Gate	Count	% All
All	71.449	100,00%
P1	33.597	47,02%

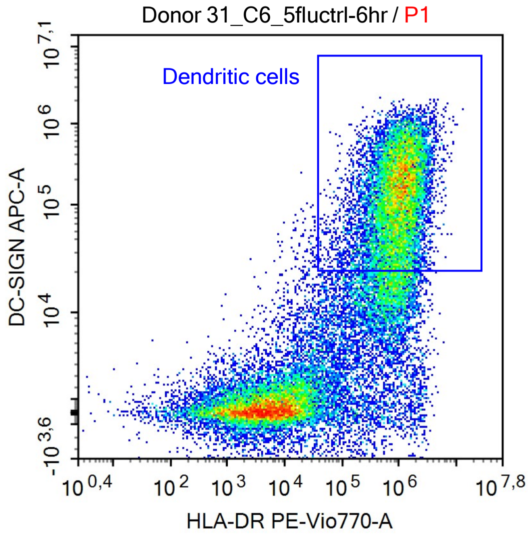

Gate	Count	% P1
P1	33.597	100,00%
Dendritic cells	12.416	36,96%

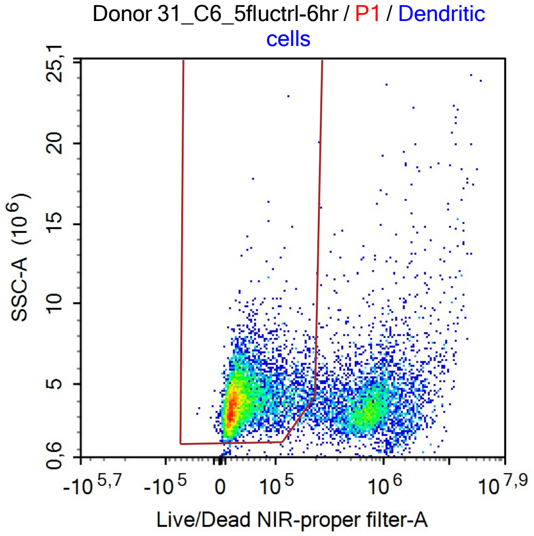

Gate	Count	% Dendritic cells
Dendritic cells	12.416	100,00%
Live	8.031	64,68%

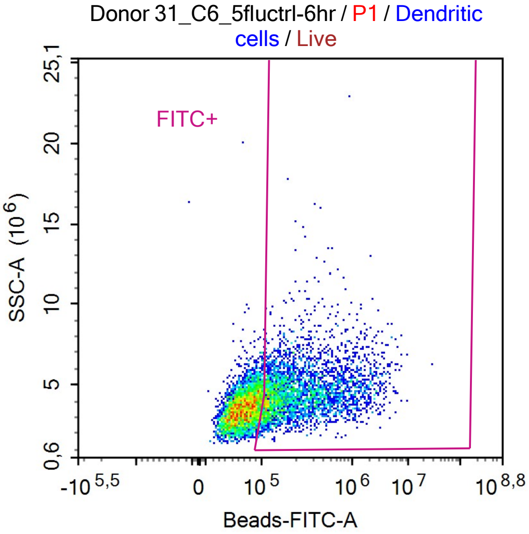

Gate	Count	% Live
Live	8.031	100,00%
FITC+	3.700	46,07%

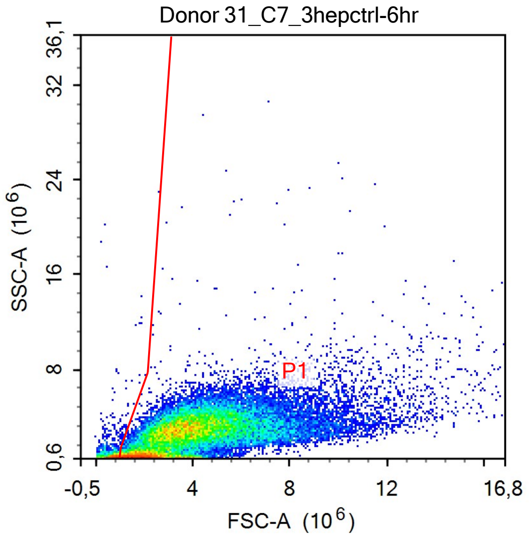

Gate	Count	% All
All	108.823	100,00%
P1	57.965	53,27%

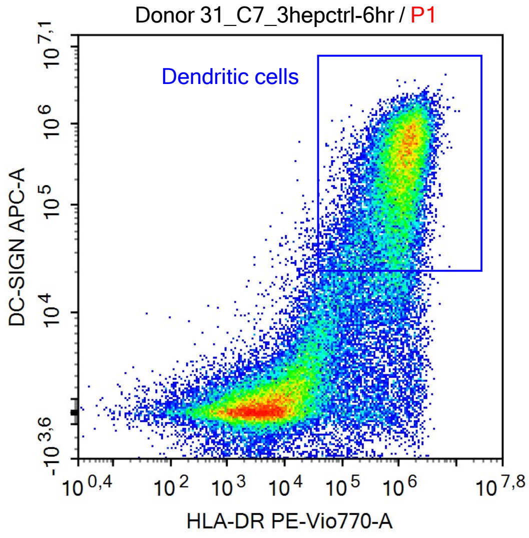

Gate	Count	% P1
P1	57.965	100,00%
Dendritic cells	19.504	33,65%

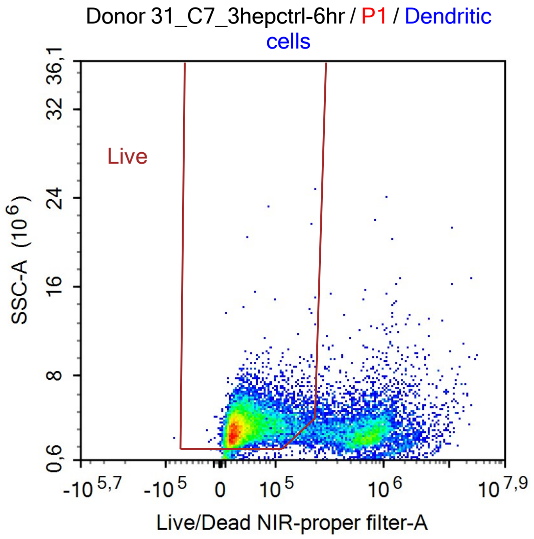

Gate	Count	% Dendritic cells
Dendritic cells	19.504	100,00%
Live	12.654	64,88%

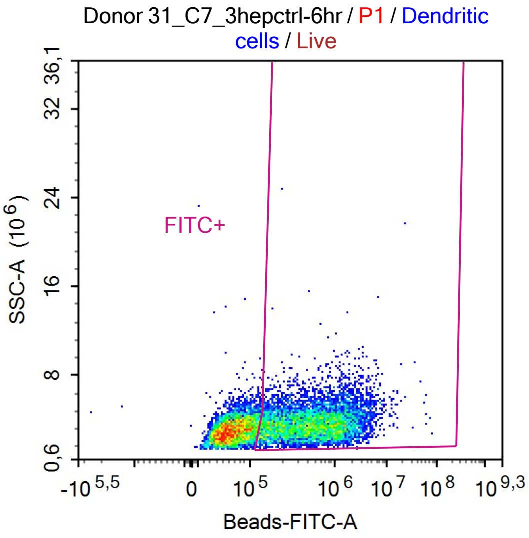

Gate	Count	% Live
Live	12.654	100,00%
FITC+	7.194	56,85%

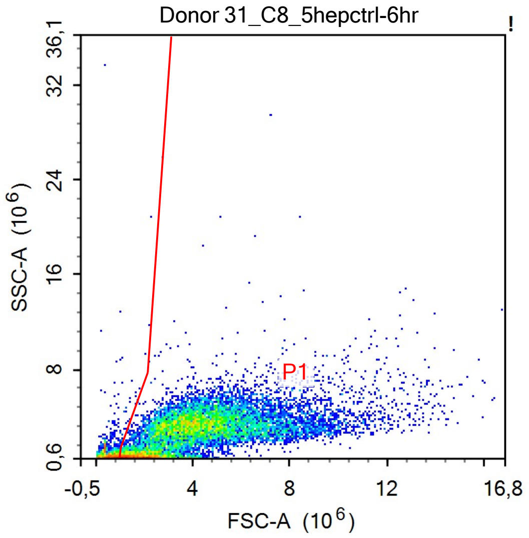

Gate	Count	% All
All	61.625	100,00%
P1	27.268	44,25%

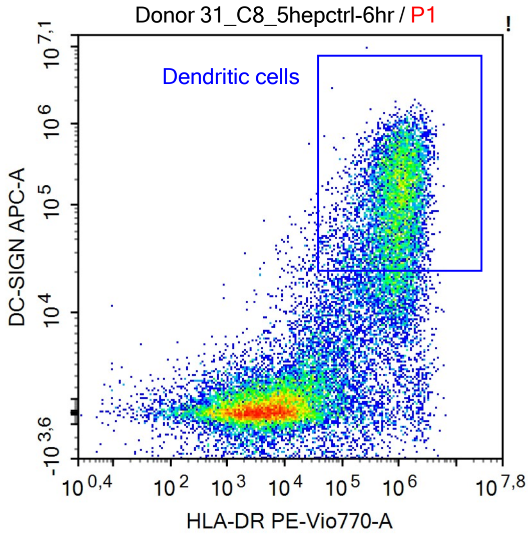

Gate	Count	% P1
P1	27.268	100,00%
Dendritic cells	6.674	24,48%

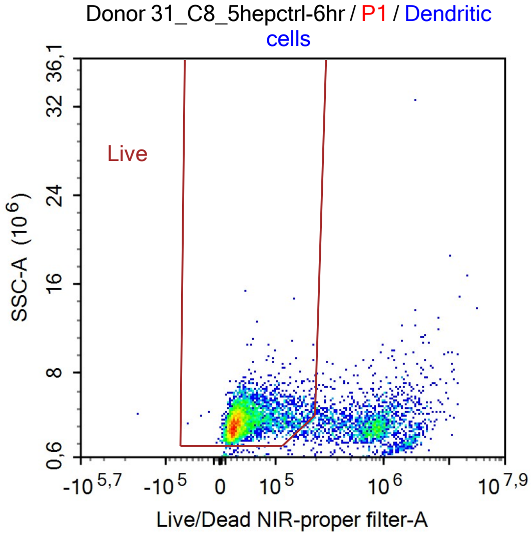

Gate	Count	% Dendritic cells
Dendritic cells	6.674	100,00%
Live	4.620	69,22%

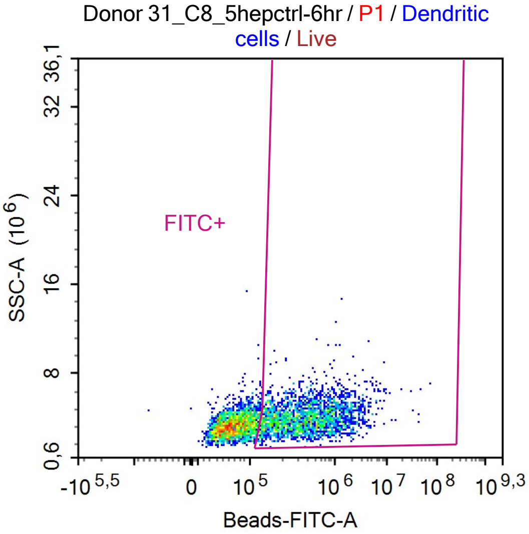

Gate	Count	% Live
Live	4.620	100,00%
FITC+	2.423	52,45%

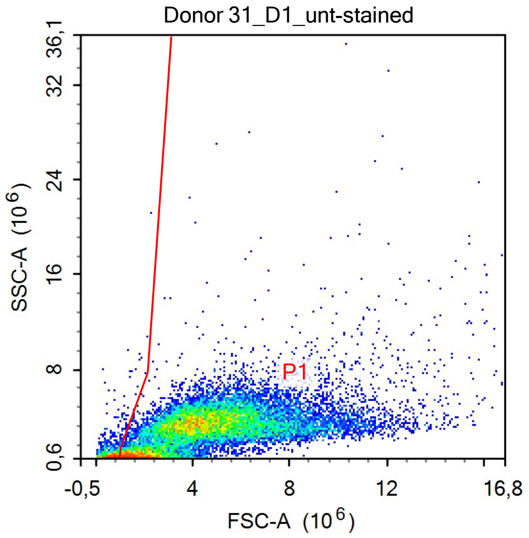

Gate	Count	% All
All	61.004	100,00%
P1	31.065	50,92%

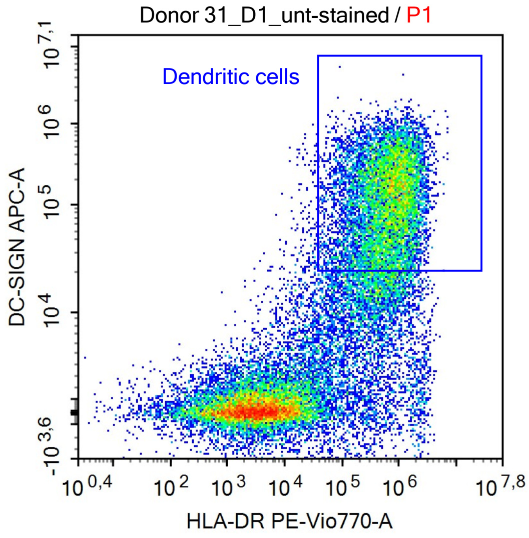

Gate	Count	% P1
P1	31.065	100,00%
Dendritic cells	10.290	33,12%

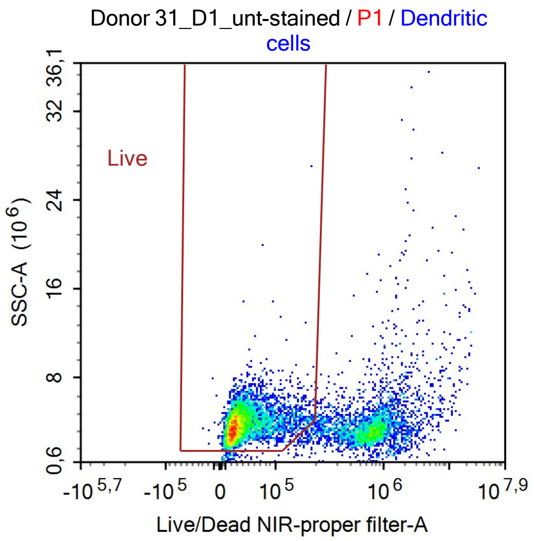

Gate	Count	% Dendritic cells
Dendritic cells	10.290	100,00%
Live	6.851	66,58%

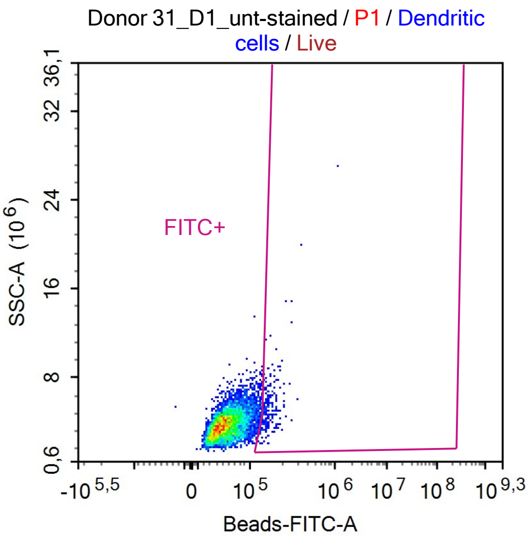

Gate	Count	% Live
Live	6.851	100,00%
FITC+	153	2,23%

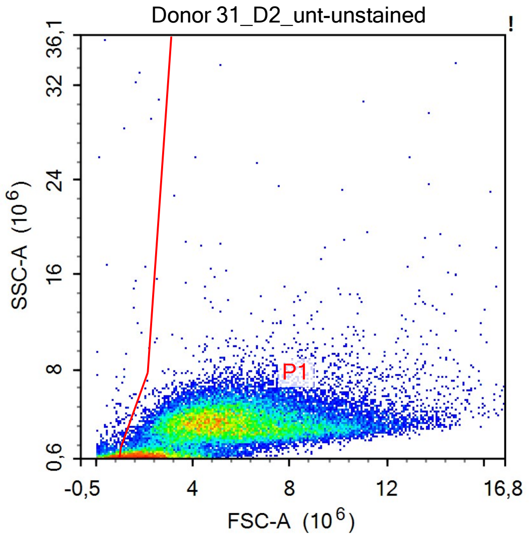

Gate	Count	% All
All	91.103	100,00%
P1	55.567	60,99%

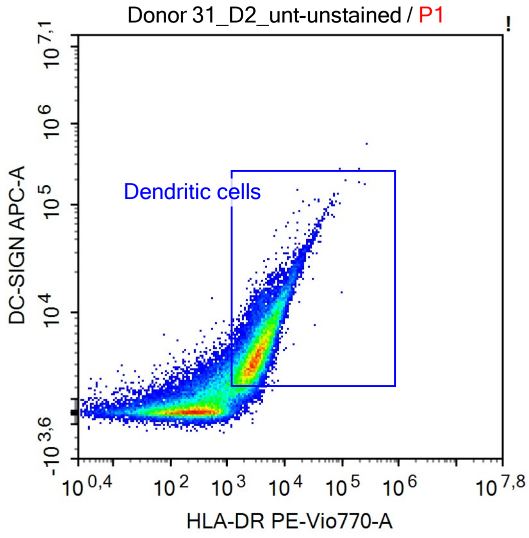

Gate	Count	% P1
P1	55.567	100,00%
Dendritic cells	22.313	40,16%

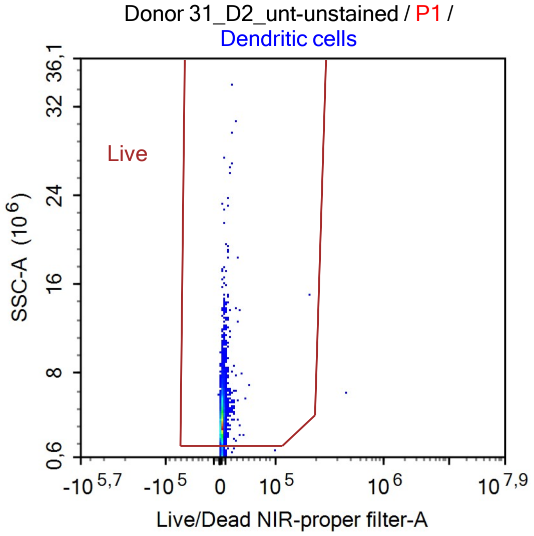

Gate	Count	% Dendritic cells
Dendritic cells	22.313	100,00%
Live	21.231	95,15%

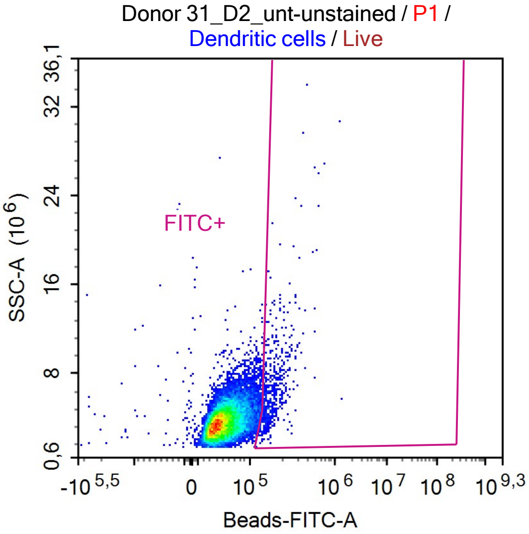

Gate	Count	% Live
Live	21.231	100,00%
FITC+	458	2,16%

Donor 31 FLU

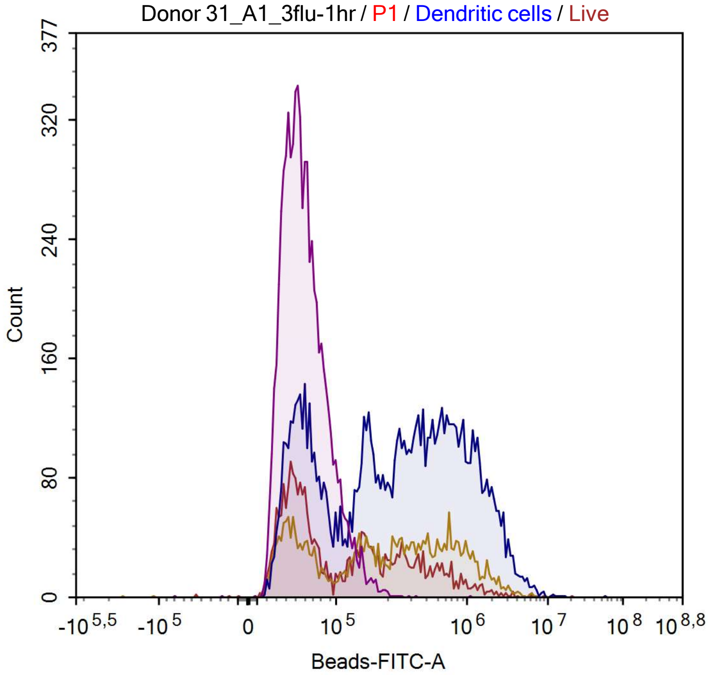

#	Sample	Gate	Count	Mean X	Median X
1	Donor 31_A1_3flu-1hr	Live	2.712	217.990	88.933
2	Donor 31_B1_3flu-3hr	Live*	2.944	426.077	201.176
3	Donor 31_C1_3flu-6hr	Live*	9.057	574.420	249.097
4	Donor 31_D1_unt-[illegible]	Live*	6.851	60.662	55.959

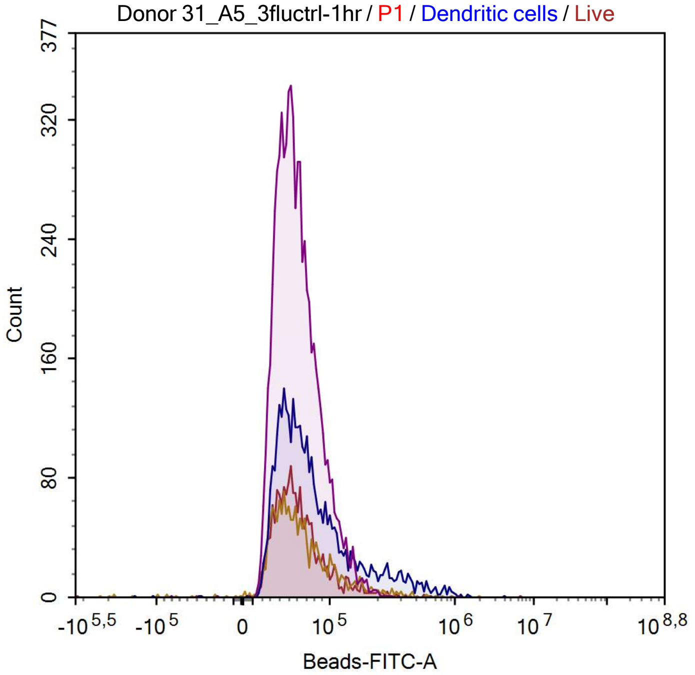

#	Sample	Gate	Count	Mean X	Median X
1	Donor 31_A5_3fluctrl-1hr	Live	1.654	69.758	56.205
2	Donor 31_B5_3fluctrl-3hr	Live*	1.582	70.459	57.122
3	Donor 31_C5_3fluctrl-6hr	Live*	3.508	102.194	66.283
4	Donor 31_D1_unt-[illegible]	Live*	6.851	60.662	55.959

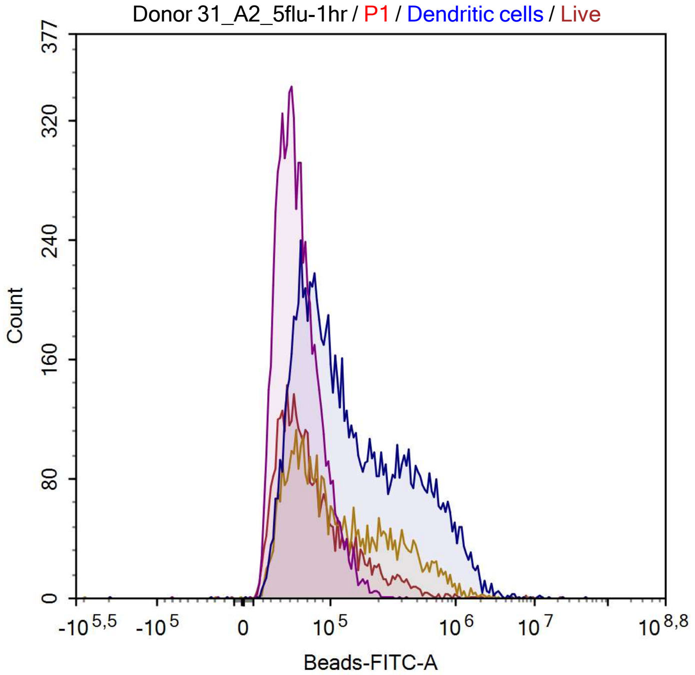

#	Sample	Gate	Count	Mean X	Median X
1	Donor 31_A2_5flu-1hr	Live	3.667	110.141	66.185
2	Donor 31_B2_5flu-3hr	Live*	4.055	186.698	95.298
3	Donor 31_C2_5flu-6hr	Live*	9.603	261.009	114.100
4	Donor 31_D1_unt-[illegible]	Live*	6.851	60.662	55.959

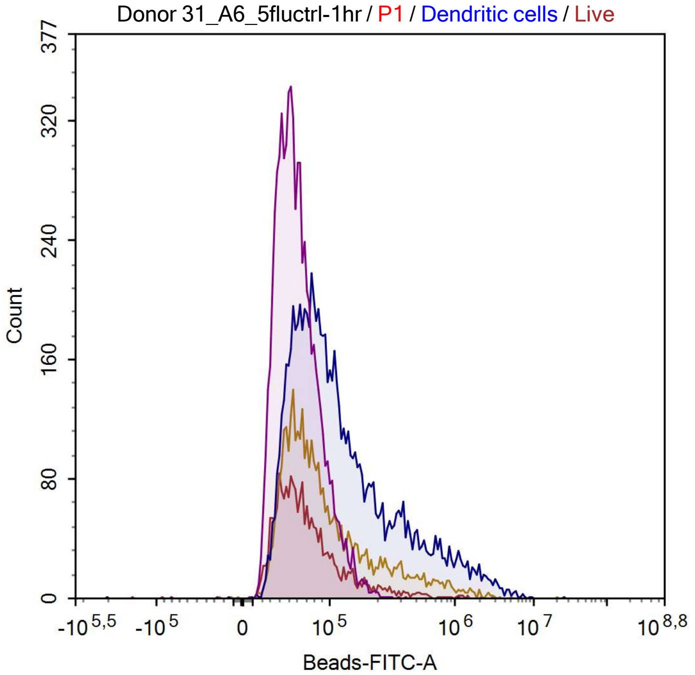

#	Sample	Gate	Count	Mean X	Median X
1	Donor 31_A6_5fluctrl-1hr	Live	2.022	89.928	63.391
2	Donor 31_B6_5fluctrl-3hr	Live*	3.708	158.557	78.868
3	Donor 31_C6_5fluctrl-6hr	Live*	8.031	247.583	98.258
4	Donor 31_D1_unt-[illegible]	Live*	6.851	60.662	55.959

Donor 31 HEP

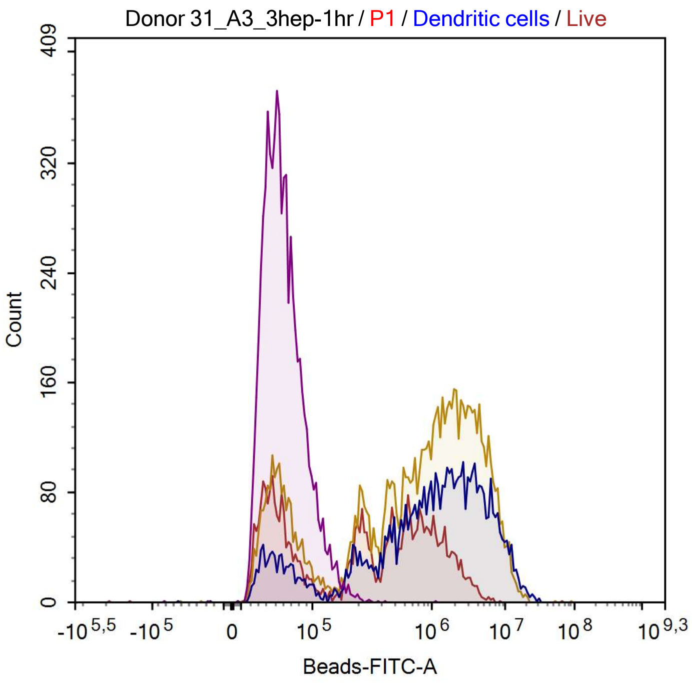

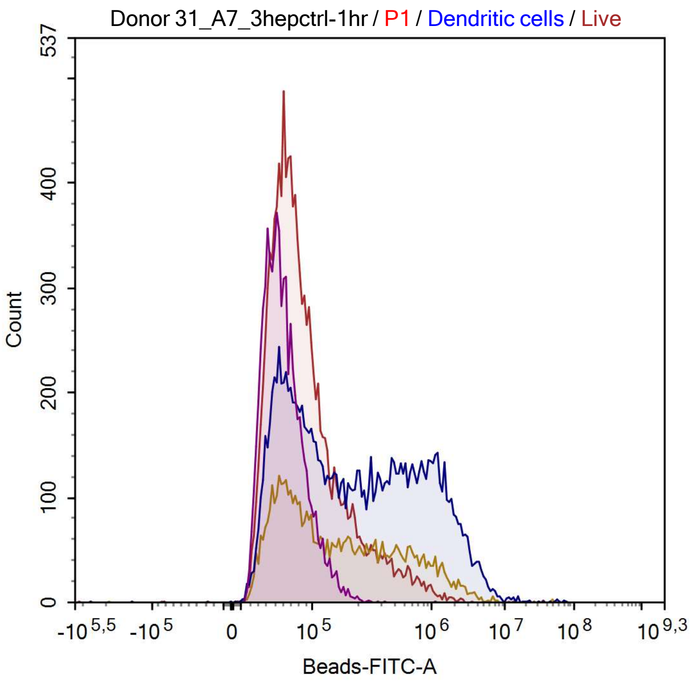

#	Sample	Gate	Count	Mean X	Median X
1	Donor 31_A3_3hep-1hr	Live	3.874	573.890	234.908
2	Donor 31_B3_3hep-3hr	Live*	8.640	1.917.822	871.598
3	Donor 31_C3_3hep-6hr	Live*	5.300	2.507.104	1.141.961
4	Donor 31_D1_unt-[illegible]	Live*	6.851	60.662	55.959

#	Sample	Gate	Count	Mean X	Median X
1	Donor 31_A7_3hepctrl-1hr	Live	11.733	135.383	75.331
2	Donor 31_B7_3hepctrl-3hr	Live*	5.304	388.358	124.624
3	Donor 31_C7_3hepctrl-6hr	Live*	12.654	607.275	168.234
4	Donor 31_D1_unt-[illegible]	Live*	6.851	60.662	55.959

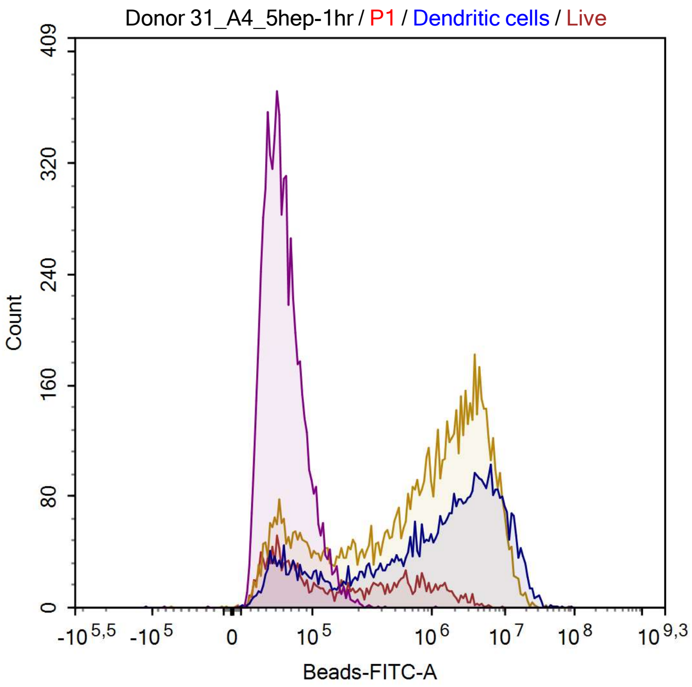

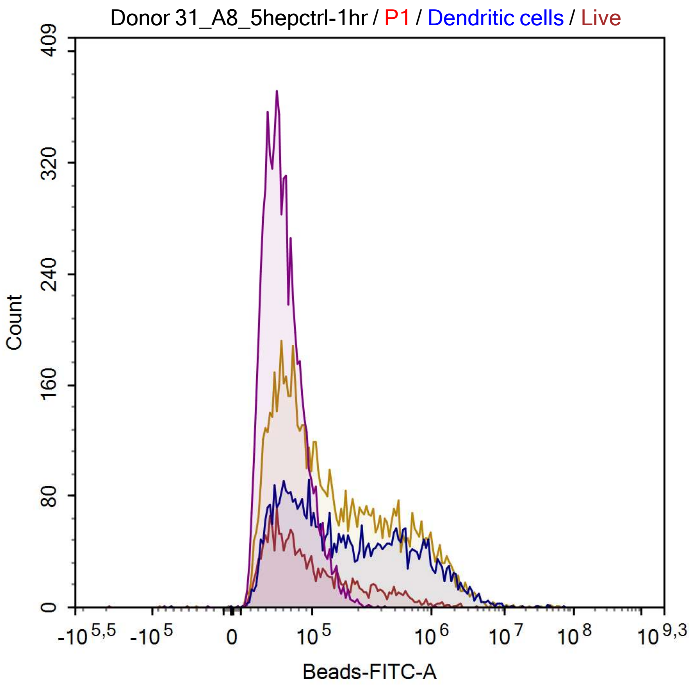

#	Sample	Gate	Count	Mean X	Median X
1	Donor 31_A4_5hep-1hr	Live	1.757	473.857	128.404
2	Donor 31_B4_5hep-3hr	Live*	8.257	2.319.345	1.055.847
3	Donor 31_C4_5hep-6hr	Live*	5.234	3.710.281	1.614.936
4	Donor 31_D1_unt-[illegible]	Live*	6.851	60.662	55.959

#	Sample	Gate	Count	Mean X	Median X
1	Donor 31_A8_5hepctrl-1hr	Live	1.903	177.205	79.521
2	Donor 31_B8_5hepctrl-3hr	Live*	7.450	371.409	108.475
3	Donor 31_C8_5hepctrl-6hr	Live*	4.620	477.545	140.718
4	Donor 31_D1_unt-[illegible]	Live*	6.851	60.662	55.959
