# Supplementary material for: Assessing the Immunomodulatory Effect of Size on the Uptake and Immunogenicity of Influenza- and Hepatitis B Subunit Vaccines In Vitro
Source: Pharmaceuticals (Basel). 2022 Jul 18;15(7):887. doi: 10.3390/ph15070887 (PMC9321264; doi:10.3390/ph15070887)
Supplement: Supplementary file 1 [file pharmaceuticals-15-00887-s001.zip › Supplementary file S2.pdf]

Report of Donor 41

Specimen Name: Donor 41 | Run Time: 26-1-2022 11:52

Cytometer: NovoCyte Quanteon 621200611403 | Software: NovoExpress 1.5.6

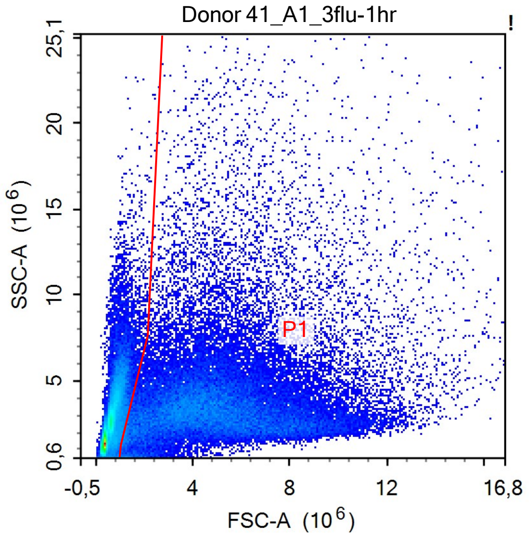

Gate	Count	% All
All	292.578	100,00%
P1	41.197	14,08%

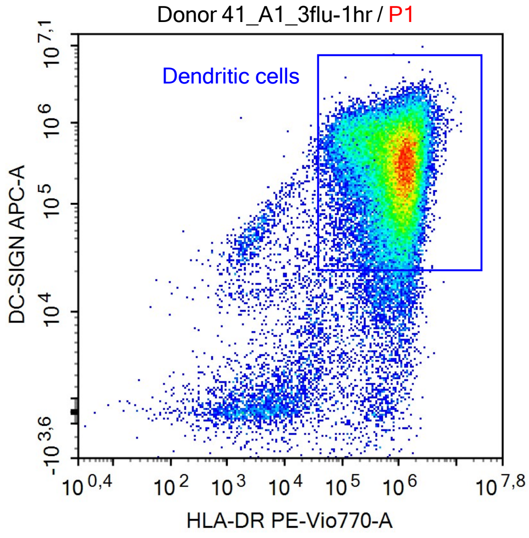

Gate	Count	% P1
P1	41.197	100,00%
Dendritic cells	34.738	84,32%

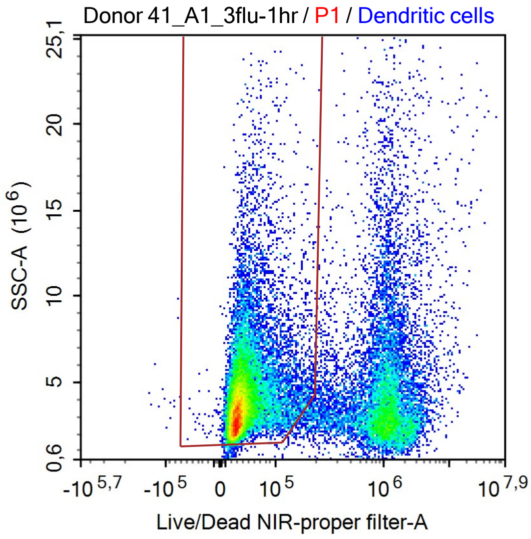

Gate	Count	% Dendritic cells
Dendritic cells	34.738	100,00%
Live	23.634	68,04%

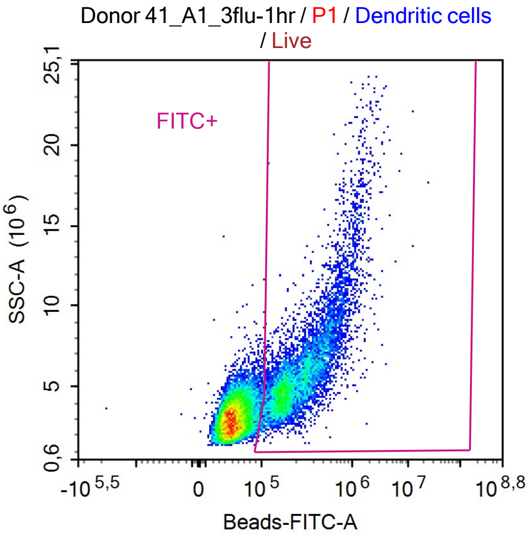

Gate	Count	% Live
Live	23.634	100,00%
FITC+	8.620	36,47%

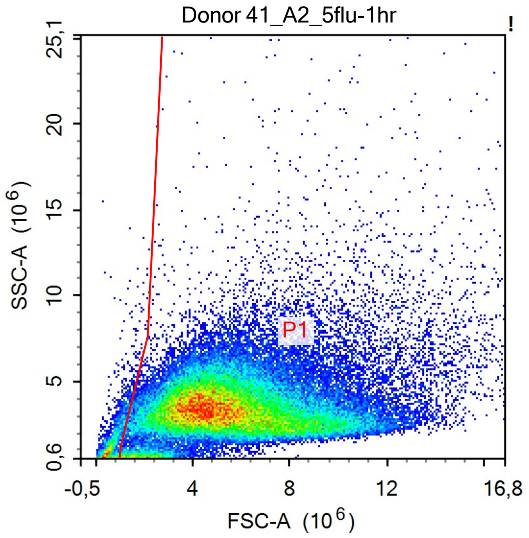

Gate	Count	% All
All	138.597	100,00%
P1	73.250	52,85%

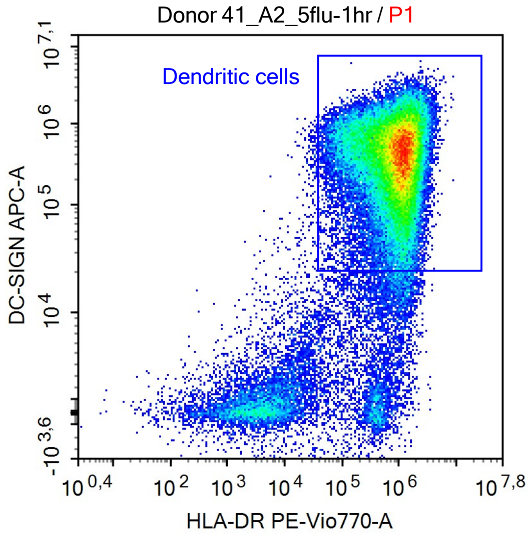

Gate	Count	% P1
P1	73.250	100,00%
Dendritic cells	60.209	82,20%

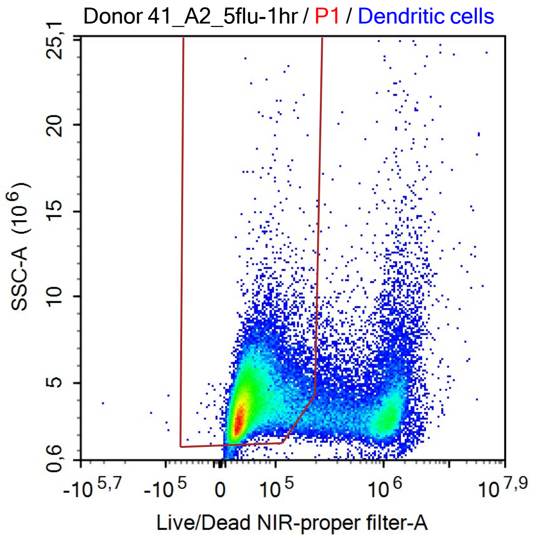

Gate	Count	% Dendritic cells
Dendritic cells	60.209	100,00%
Live	46.372	77,02%

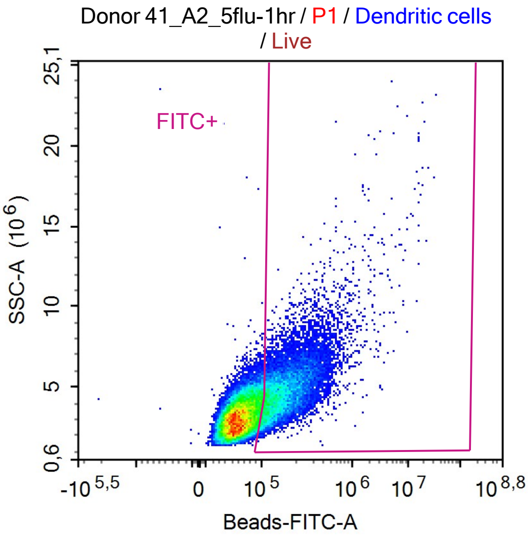

Gate	Count	% Live
Live	46.372	100,00%
FITC+	13.694	29,53%

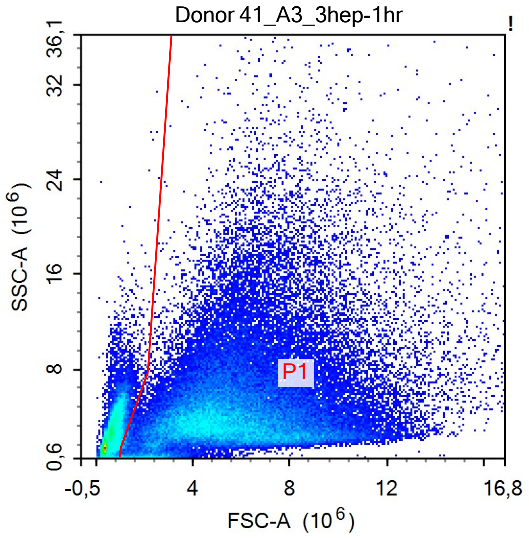

Gate	Count	% All
All	296.769	100,00%
P1	66.806	22,51%

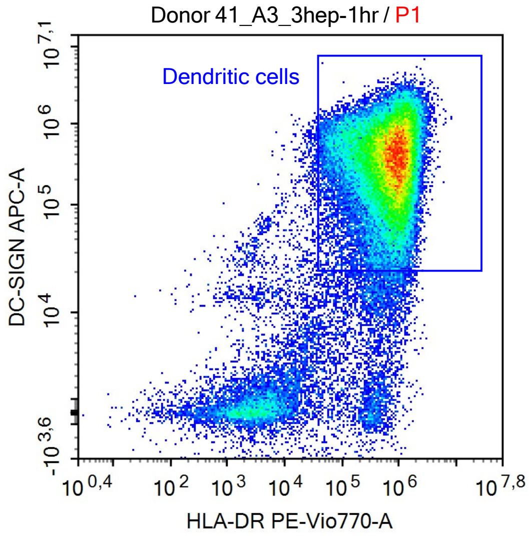

Gate	Count	% P1
P1	66.806	100,00%
Dendritic cells	54.259	81,22%

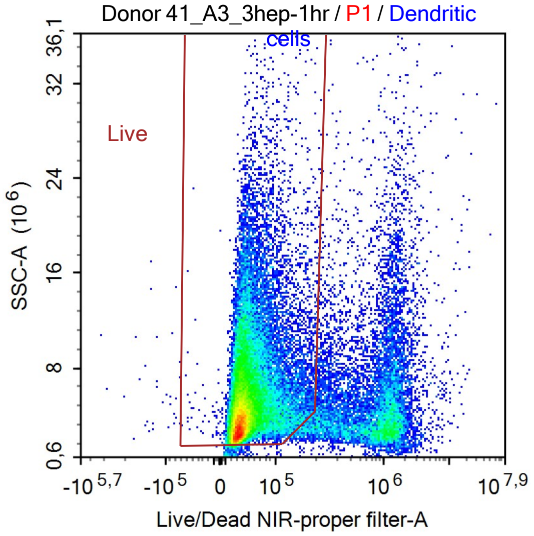

Gate	Count	% Dendritic cells
Dendritic cells	54.259	100,00%
Live	43.371	79,93%

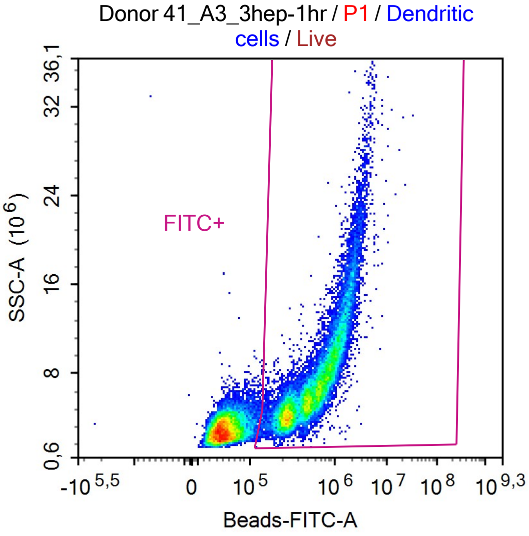

Gate	Count	% Live
Live	43.371	100,00%
FITC+	25.328	58,40%

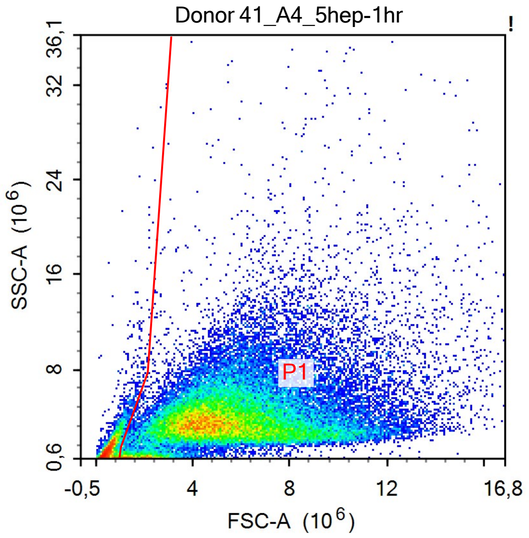

Gate	Count	% All
All	117.372	100,00%
P1	48.110	40,99%

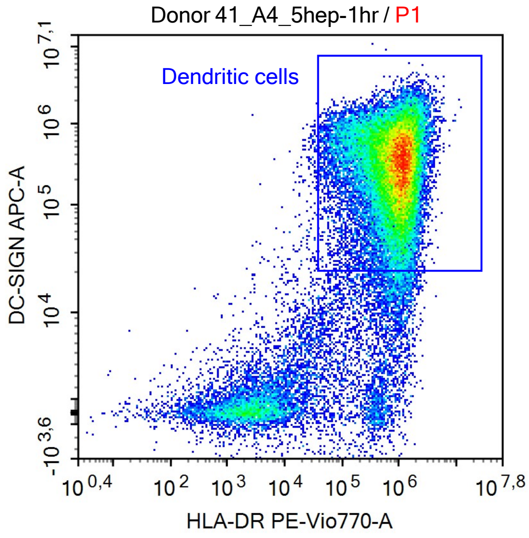

Gate	Count	% P1
P1	48.110	100,00%
Dendritic cells	37.139	77,20%

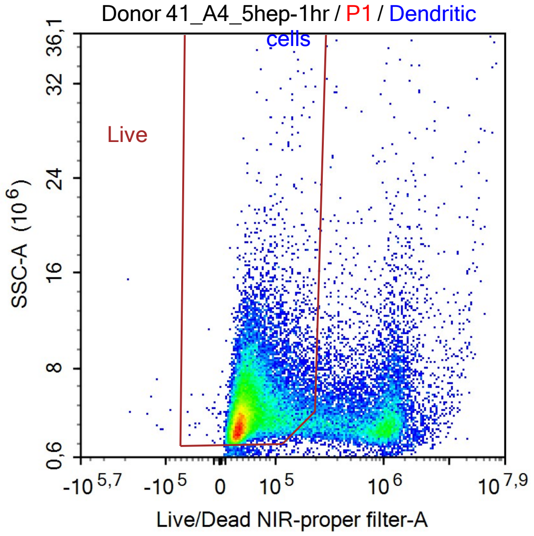

Gate	Count	% Dendritic cells
Dendritic cells	37.139	100,00%
Live	28.309	76,22%

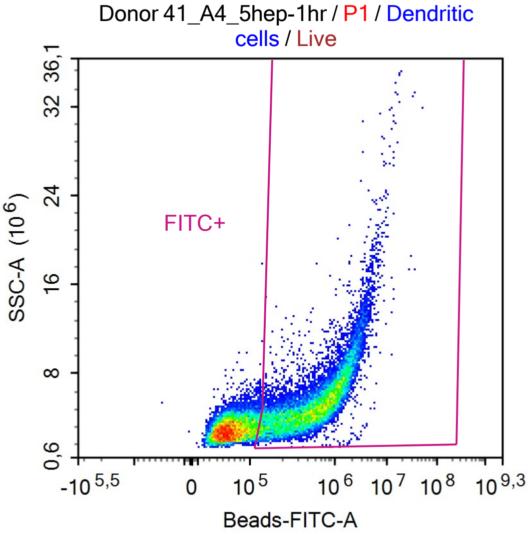

Gate	Count	% Live
Live	28.309	100,00%
FITC+	16.223	57,31%

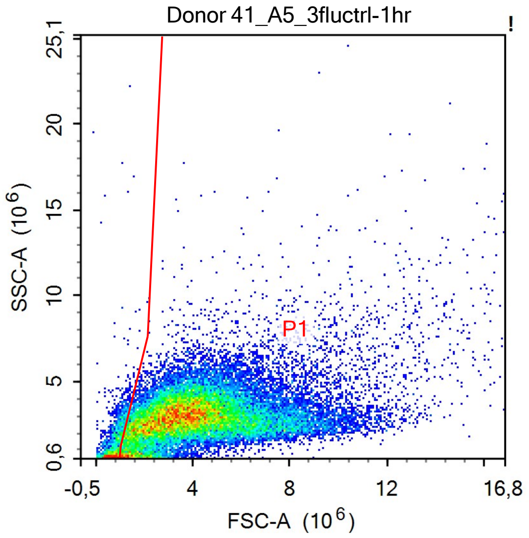

Gate	Count	% All
All	72.535	100,00%
P1	32.199	44,39%

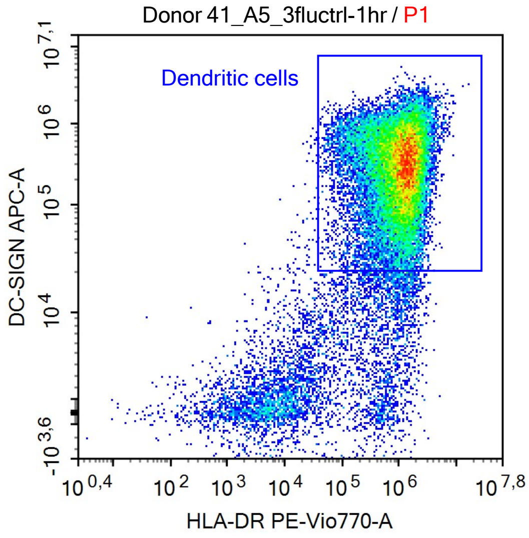

Gate	Count	% P1
P1	32.199	100,00%
Dendritic cells	26.063	80,94%

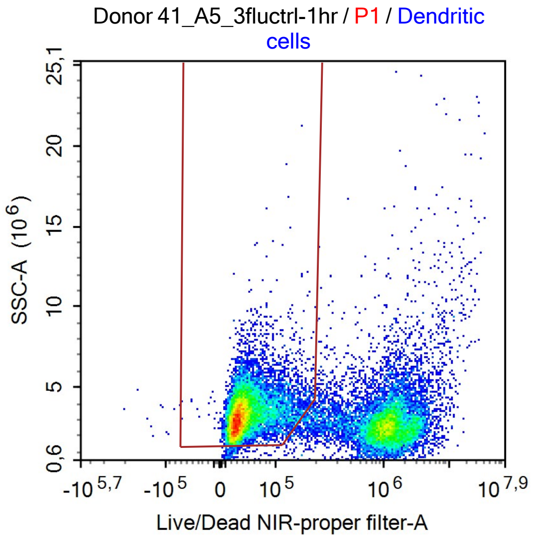

Gate	Count	% Dendritic cells
Dendritic cells	26.063	100,00%
Live	14.842	56,95%

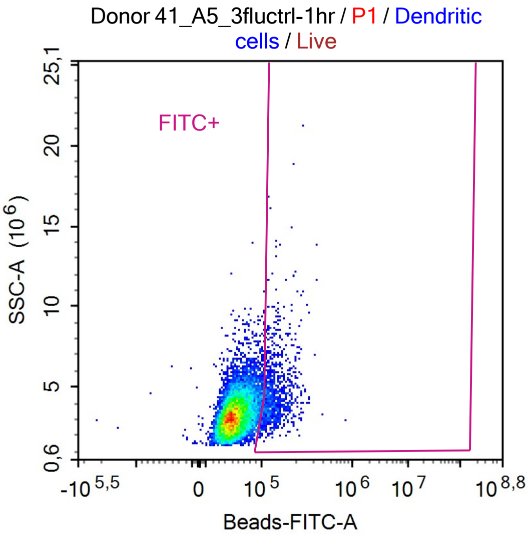

Gate	Count	% Live
Live	14.842	100,00%
FITC+	957	6,45%

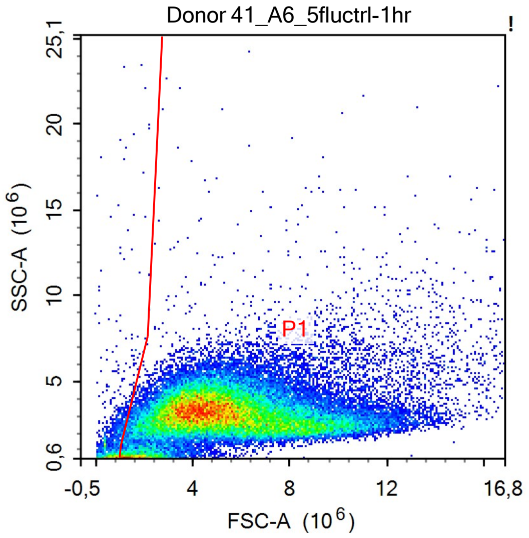

Gate	Count	% All
All	114.140	100,00%
P1	59.889	52,47%

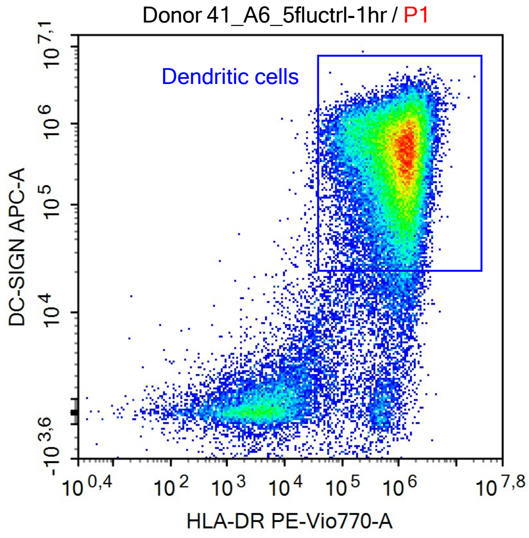

Gate	Count	% P1
P1	59.889	100,00%
Dendritic cells	46.088	76,96%

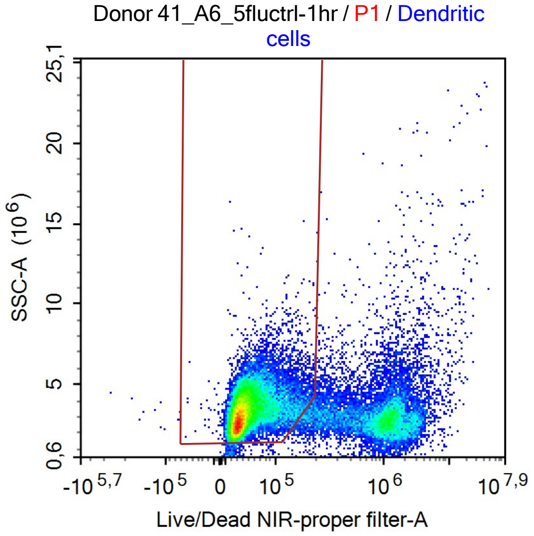

Gate	Count	% Dendritic cells
Dendritic cells	46.088	100,00%
Live	33.745	73,22%

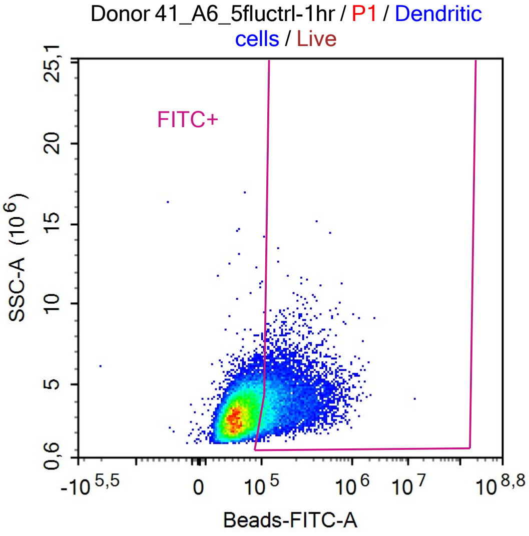

Gate	Count	% Live
Live	33.745	100,00%
FITC+	7.074	20,96%

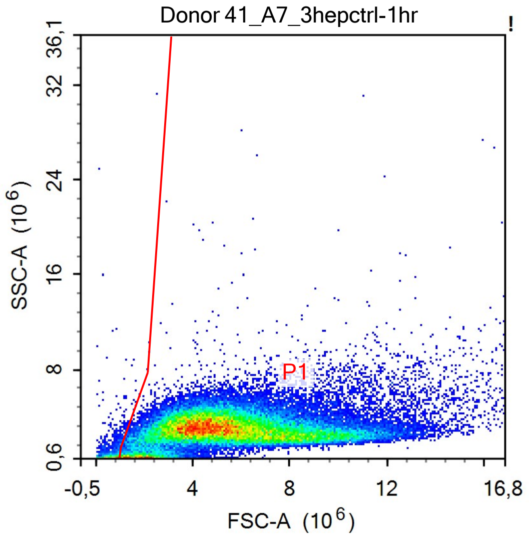

Gate	Count	% All
All	123.513	100,00%
P1	64.231	52,00%

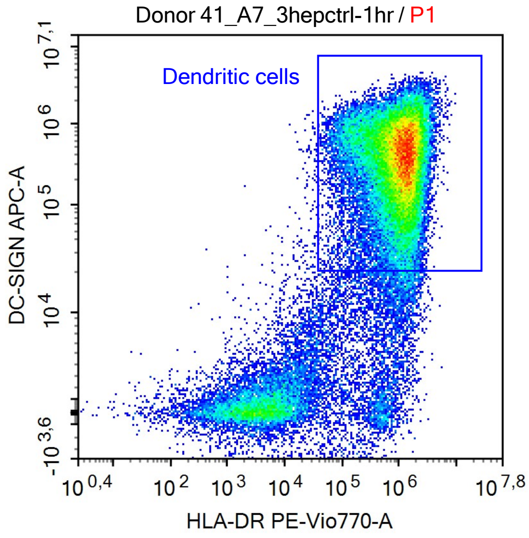

Gate	Count	% P1
P1	64.231	100,00%
Dendritic cells	47.267	73,59%

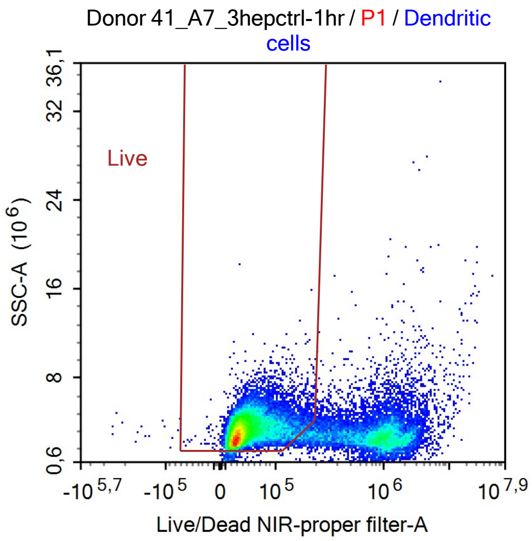

Gate	Count	% Dendritic cells
Dendritic cells	47.267	100,00%
Live	35.329	74,74%

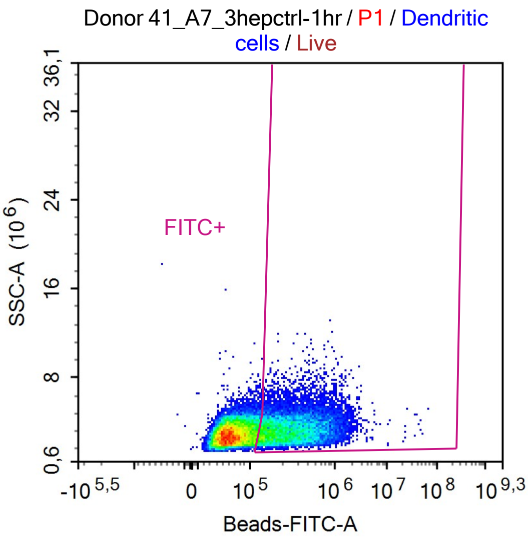

Gate	Count	% Live
Live	35.329	100,00%
FITC+	14.059	39,79%

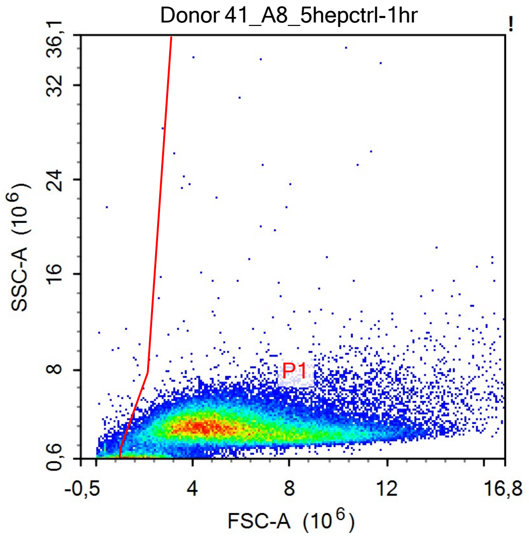

Gate	Count	% All
All	122.529	100,00%
P1	63.321	51,68%

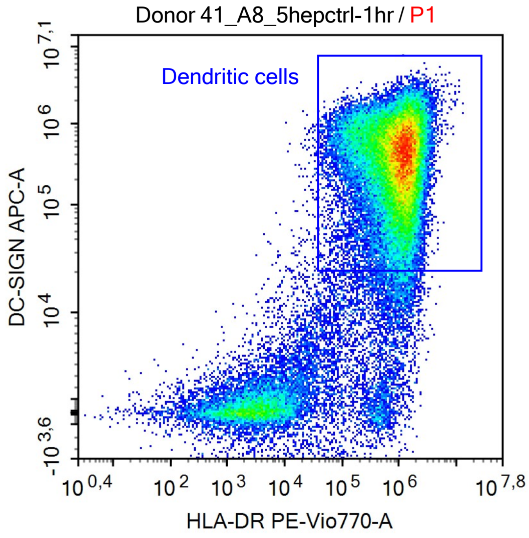

Gate	Count	% P1
P1	63.321	100,00%
Dendritic cells	46.562	73,53%

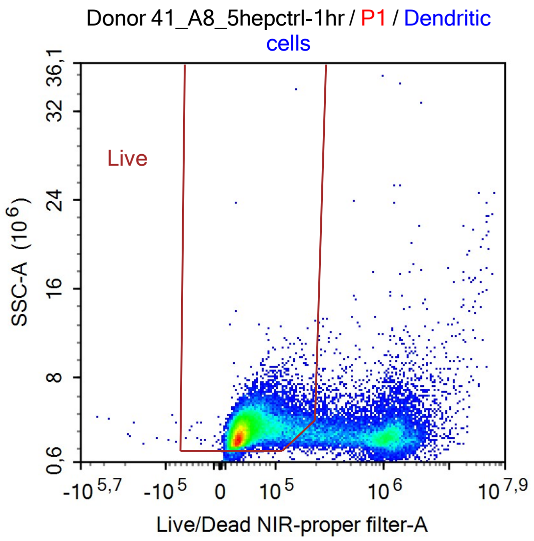

Gate	Count	% Dendritic cells
Dendritic cells	46.562	100,00%
Live	35.273	75,75%

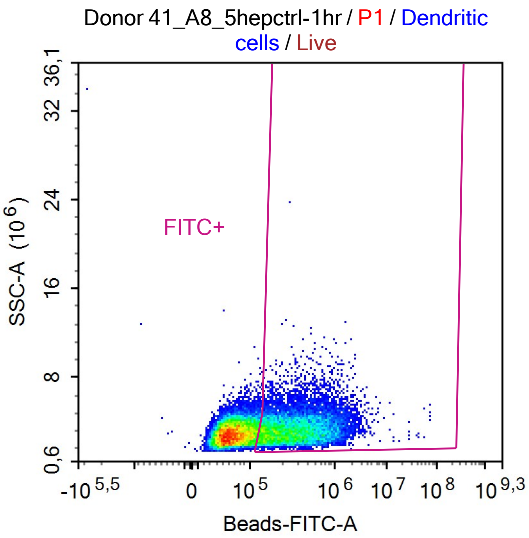

Gate	Count	% Live
Live	35.273	100,00%
FITC+	15.912	45,11%

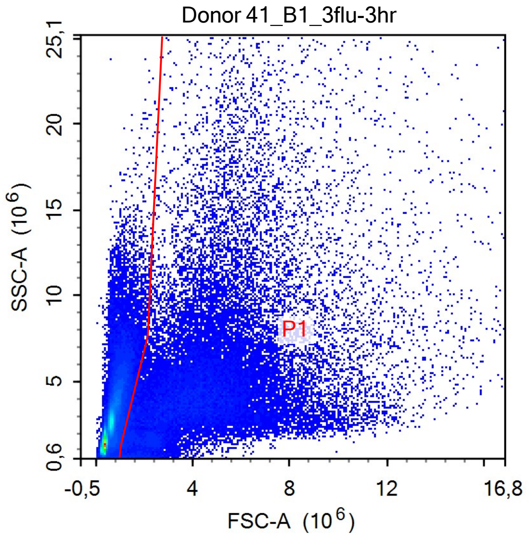

Gate	Count	% All
All	588.606	100,00%
P1	42.847	7,28%

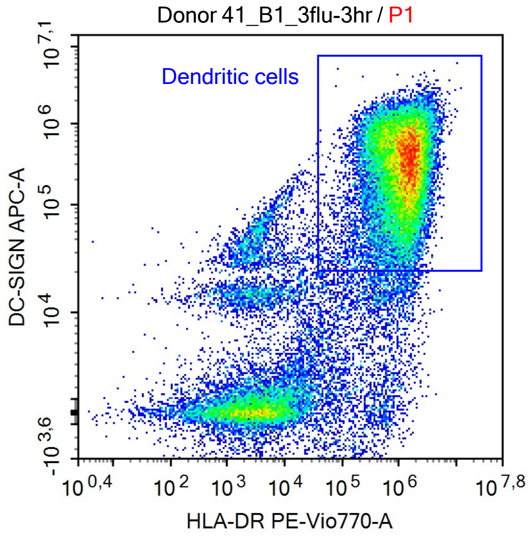

Gate	Count	% P1
P1	42.847	100,00%
Dendritic cells	25.472	59,45%

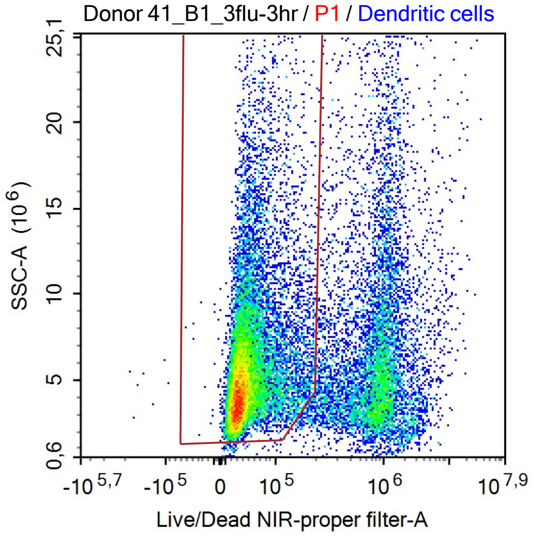

Gate	Count	% Dendritic cells
Dendritic cells	25.472	100,00%
Live	17.286	67,86%

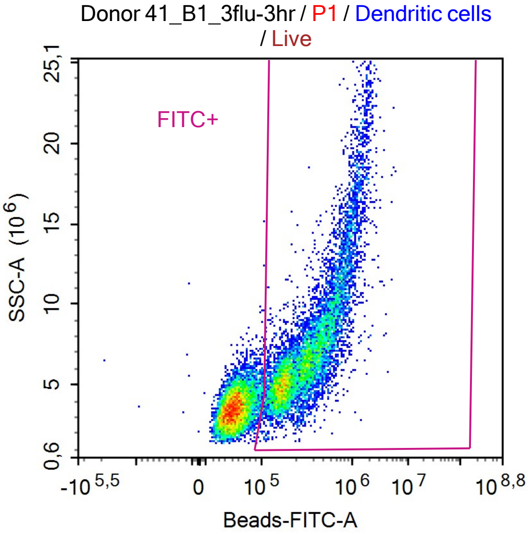

Gate	Count	% Live
Live	17.286	100,00%
FITC+	9.980	57,73%

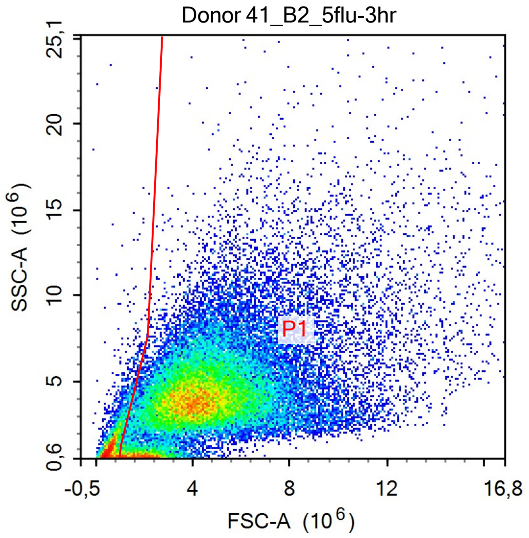

Gate	Count	% All
All	139.375	100,00%
P1	59.276	42,53%

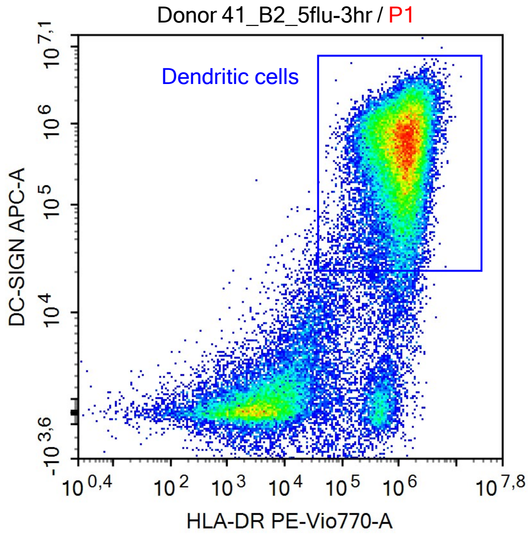

Gate	Count	% P1
P1	59.276	100,00%
Dendritic cells	36.766	62,03%

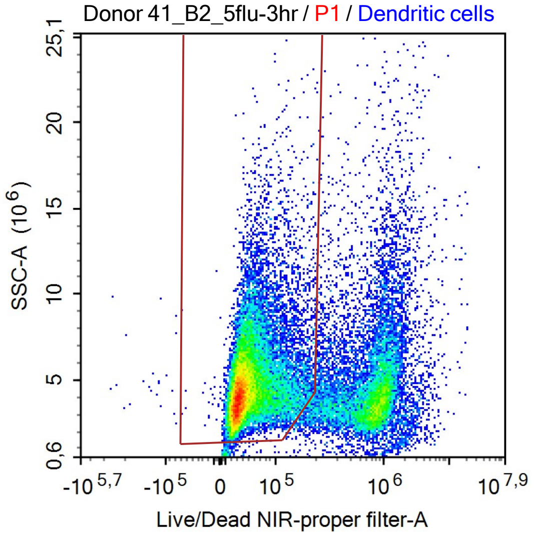

Gate	Count	% Dendritic cells
Dendritic cells	36.766	100,00%
Live	24.534	66,73%

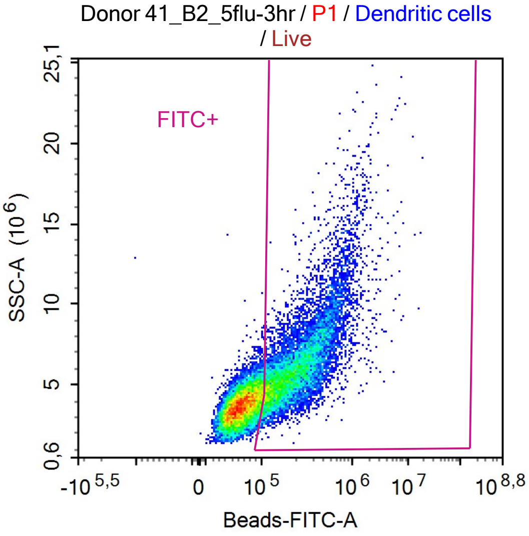

Gate	Count	% Live
Live	24.534	100,00%
FITC+	11.289	46,01%

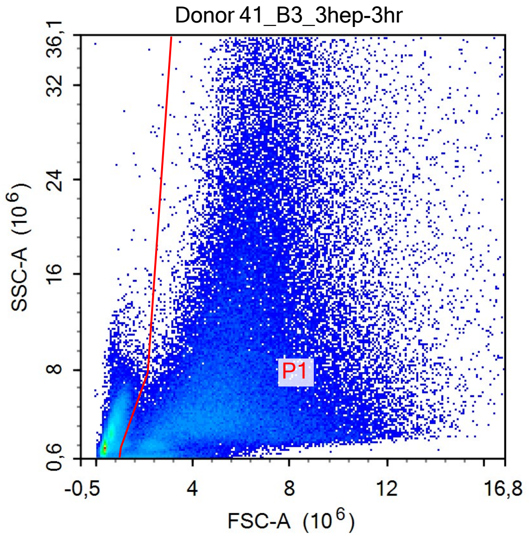

Gate	Count	% All
All	477.080	100,00%
P1	103.084	21,61%

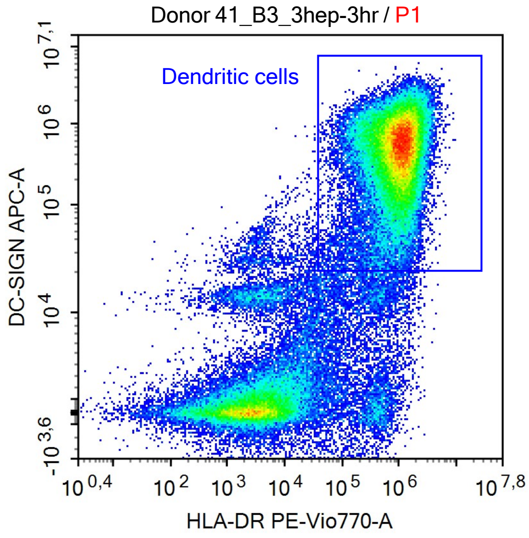

Gate	Count	% P1
P1	103.084	100,00%
Dendritic cells	59.833	58,04%

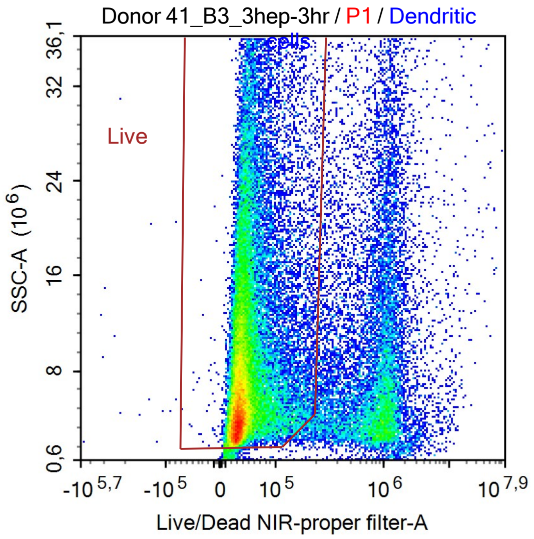

Gate	Count	% Dendritic cells
Dendritic cells	59.833	100,00%
Live	45.130	75,43%

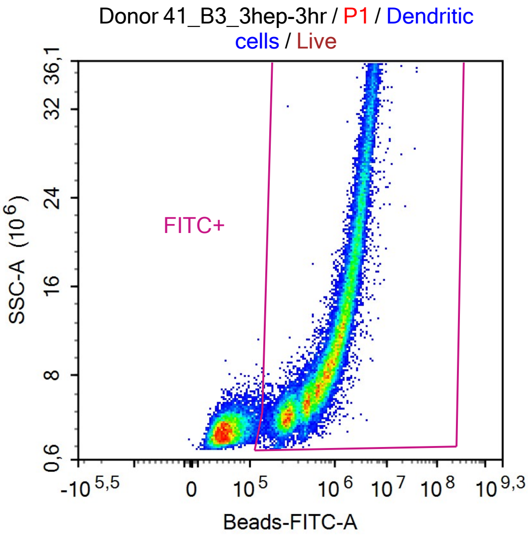

Gate	Count	% Live
Live	45.130	100,00%
FITC+	34.980	77,51%

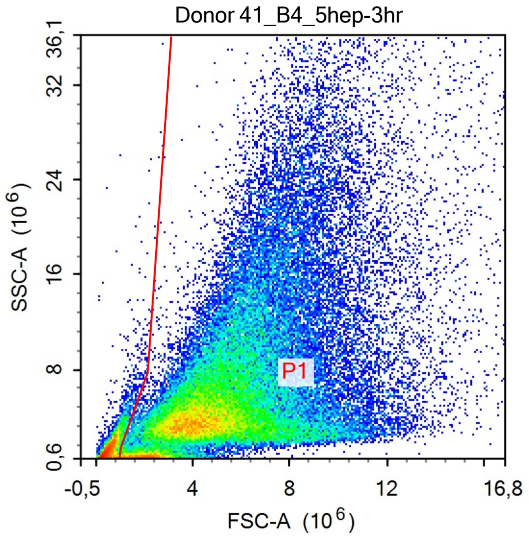

Gate	Count	% All
All	191.828	100,00%
P1	89.693	46,76%

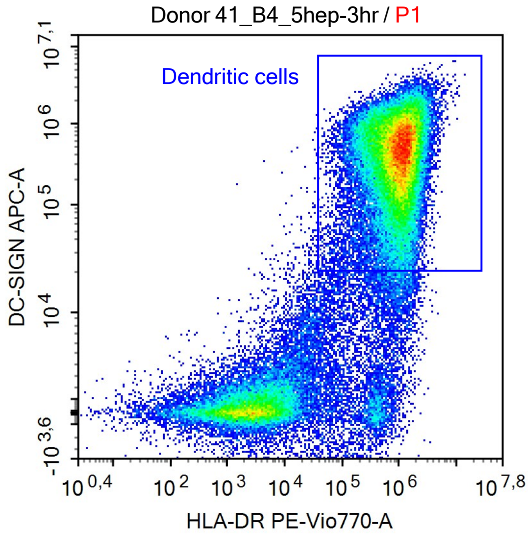

Gate	Count	% P1
P1	89.693	100,00%
Dendritic cells	56.714	63,23%

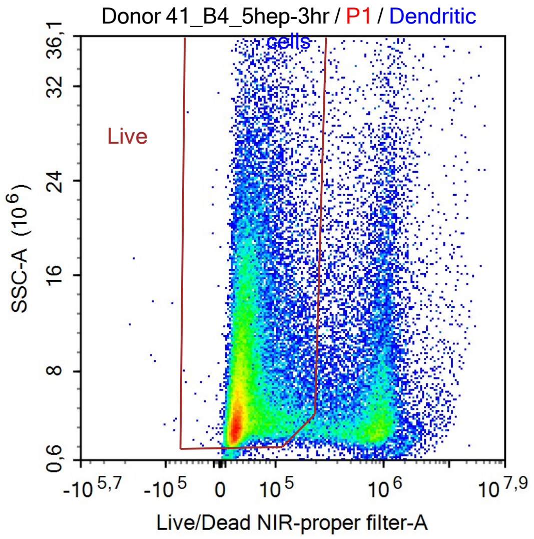

Gate	Count	% Dendritic cells
Dendritic cells	56.714	100,00%
Live	41.972	74,01%

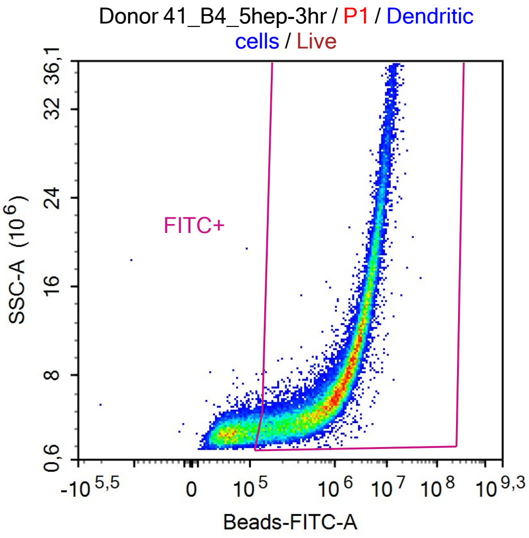

Gate	Count	% Live
Live	41.972	100,00%
FITC+	33.903	80,78%

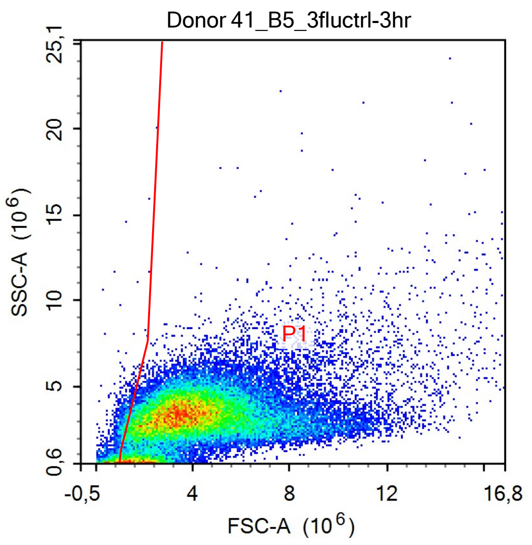

Gate	Count	% All
All	110.681	100,00%
P1	56.934	51,44%

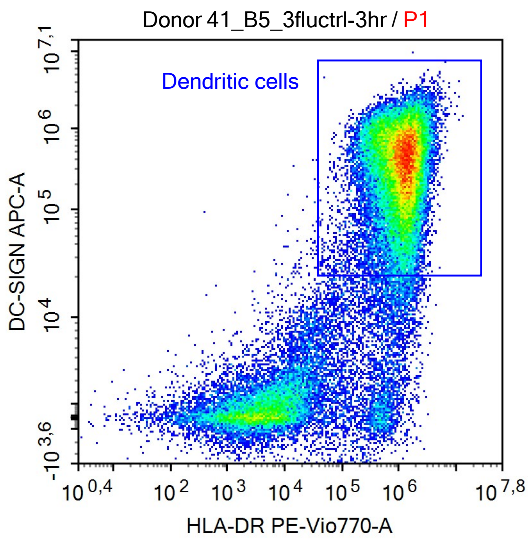

Gate	Count	% P1
P1	56.934	100,00%
Dendritic cells	36.783	64,61%

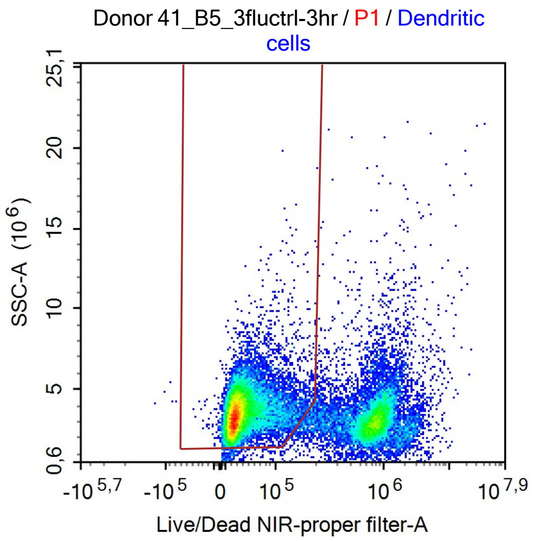

Gate	Count	% Dendritic cells
Dendritic cells	36.783	100,00%
Live	23.919	65,03%

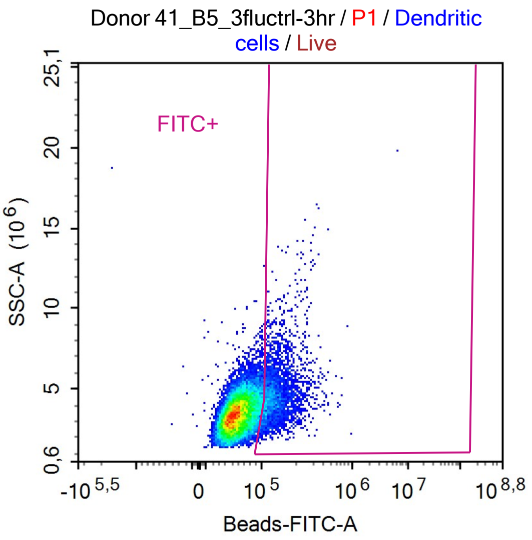

Gate	Count	% Live
Live	23.919	100,00%
FITC+	2.788	11,66%

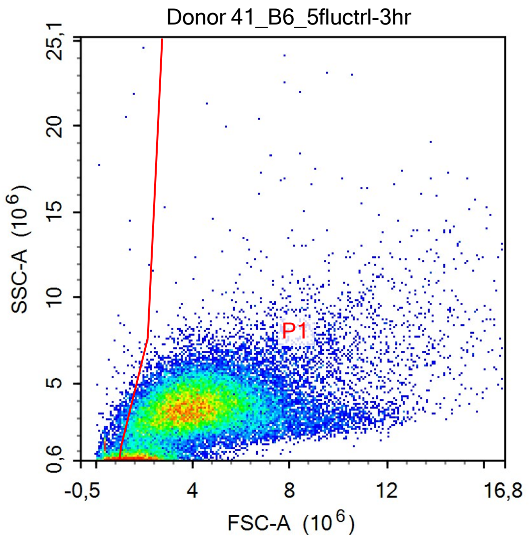

Gate	Count	% All
All	119.233	100,00%
P1	58.180	48,80%

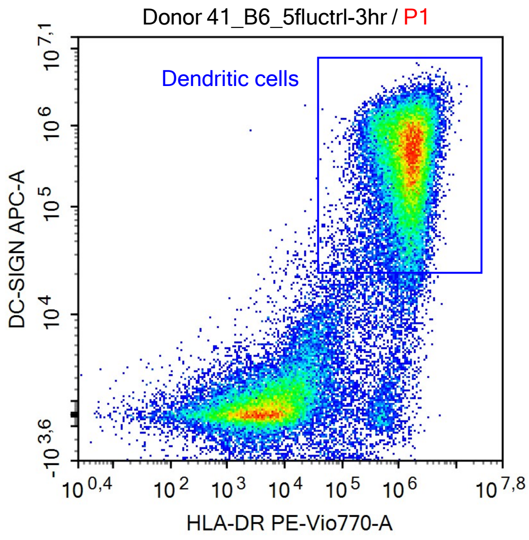

Gate	Count	% P1
P1	58.180	100,00%
Dendritic cells	28.470	48,93%

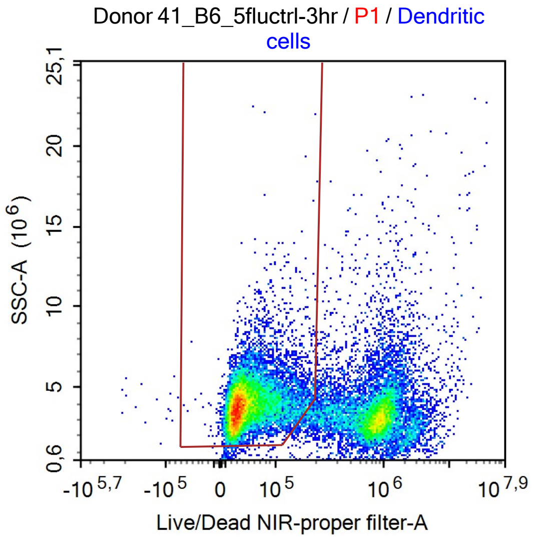

Gate	Count	% Dendritic cells
Dendritic cells	28.470	100,00%
Live	16.835	59,13%

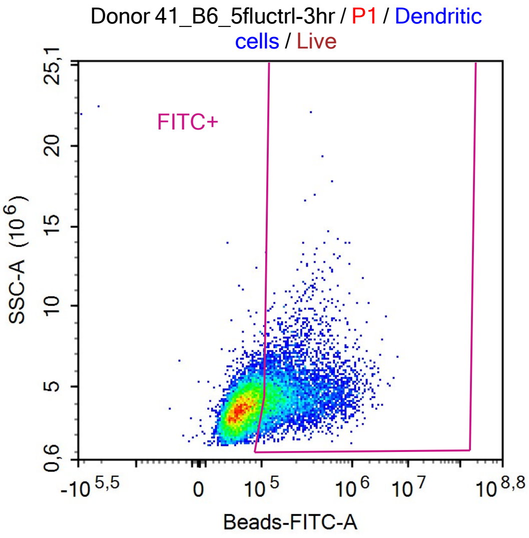

Gate	Count	% Live
Live	16.835	100,00%
FITC+	5.503	32,69%

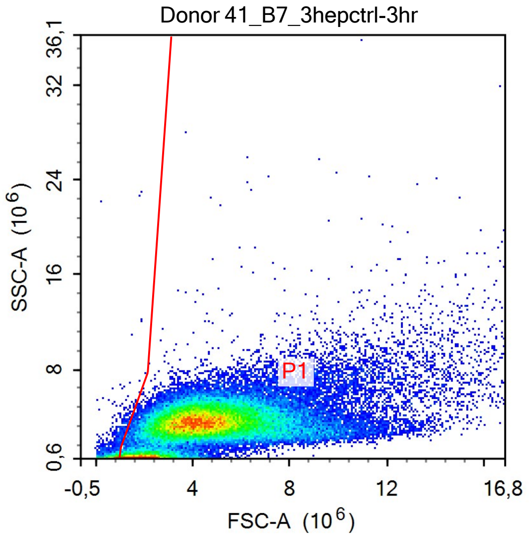

Gate	Count	% All
All	174.603	100,00%
P1	96.409	55,22%

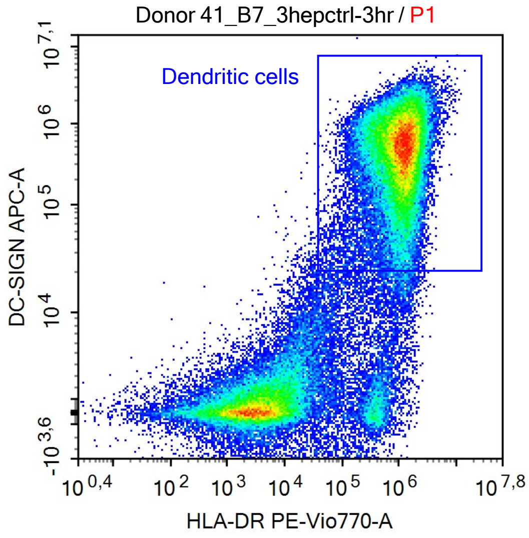

Gate	Count	% P1
P1	96.409	100,00%
Dendritic cells	49.817	51,67%

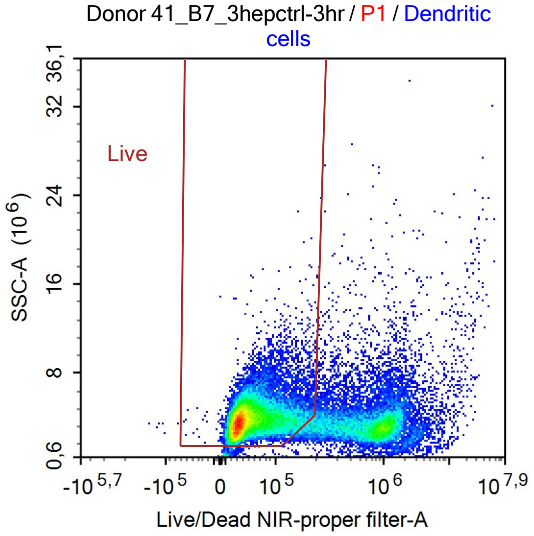

Gate	Count	% Dendritic cells
Dendritic cells	49.817	100,00%
Live	34.231	68,71%

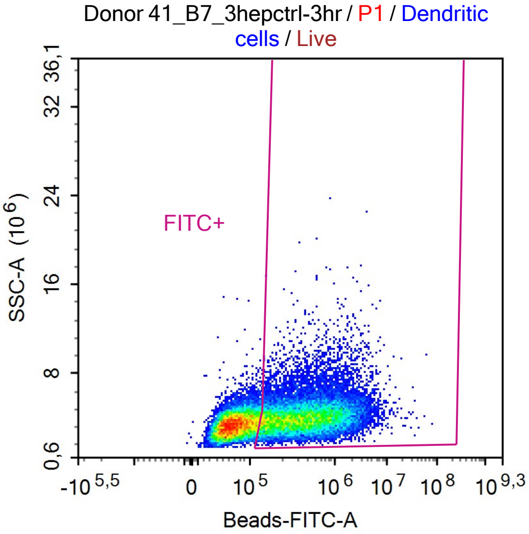

Gate	Count	% Live
Live	34.231	100,00%
FITC+	18.927	55,29%

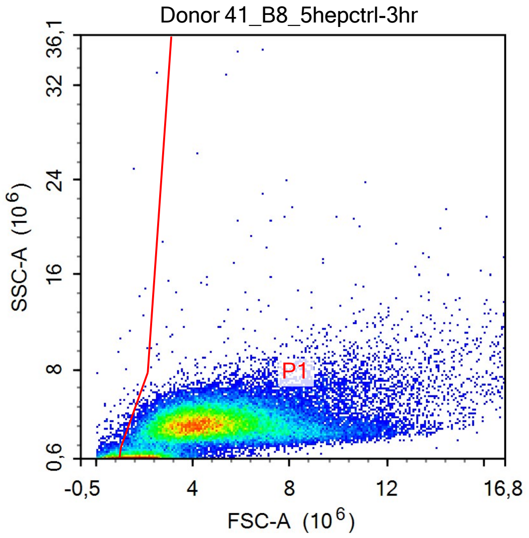

Gate	Count	% All
All	149.643	100,00%
P1	81.648	54,56%

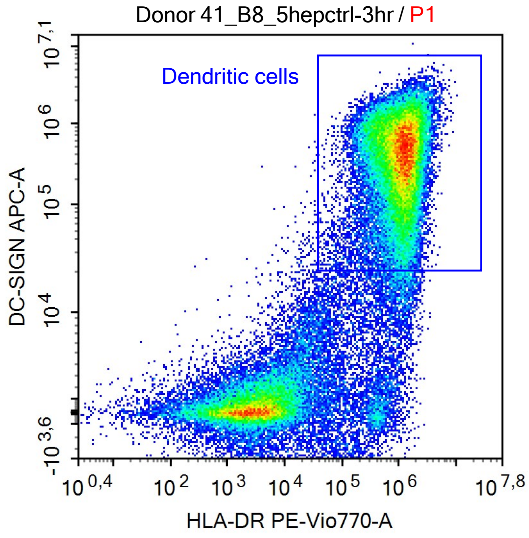

Gate	Count	% P1
P1	81.648	100,00%
Dendritic cells	42.364	51,89%

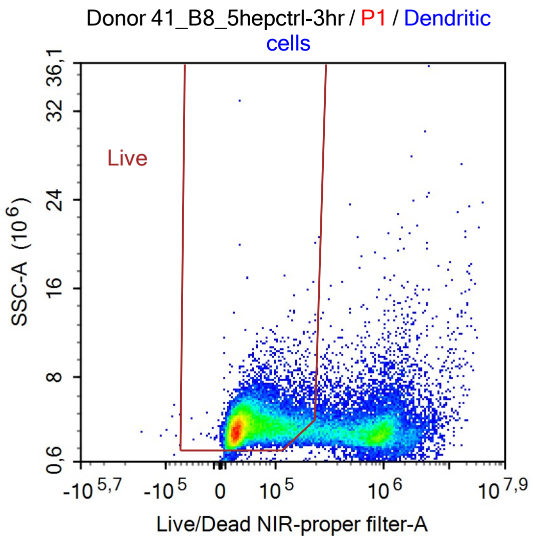

Gate	Count	% Dendritic cells
Dendritic cells	42.364	100,00%
Live	28.439	67,13%

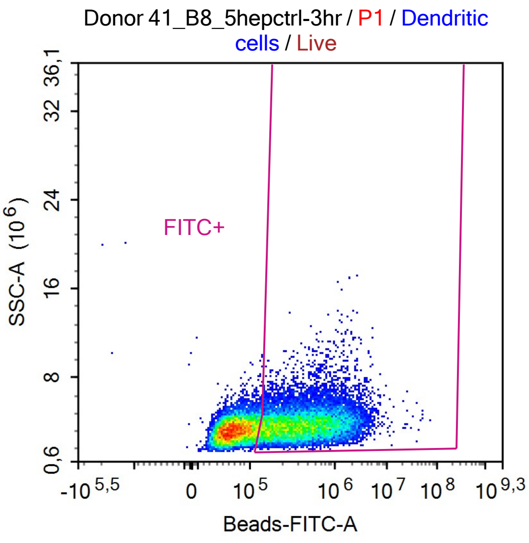

Gate	Count	% Live
Live	28.439	100,00%
FITC+	15.507	54,53%

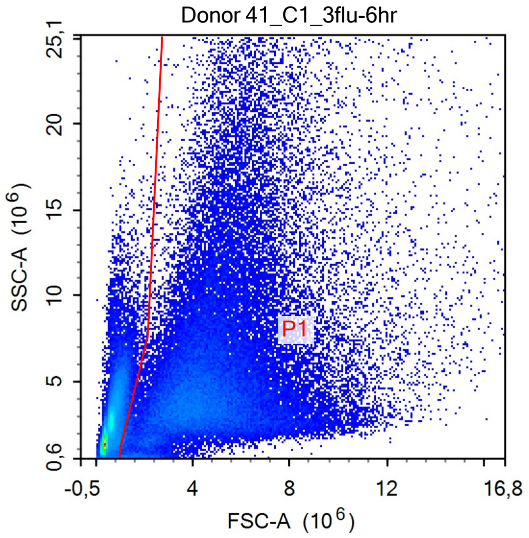

Gate	Count	% All
All	463.147	100,00%
P1	65.788	14,20%

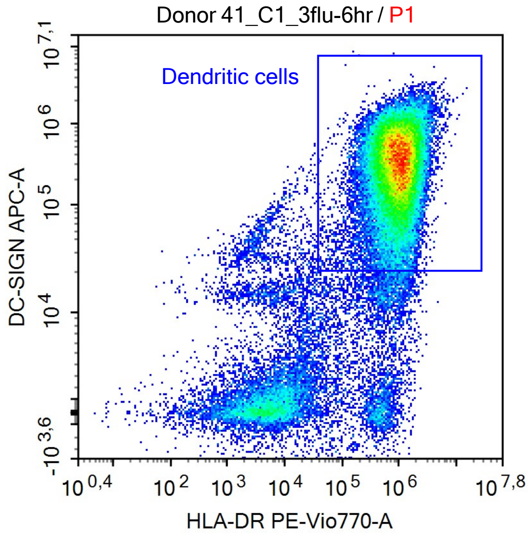

Gate	Count	% P1
P1	65.788	100,00%
Dendritic cells	50.573	76,87%

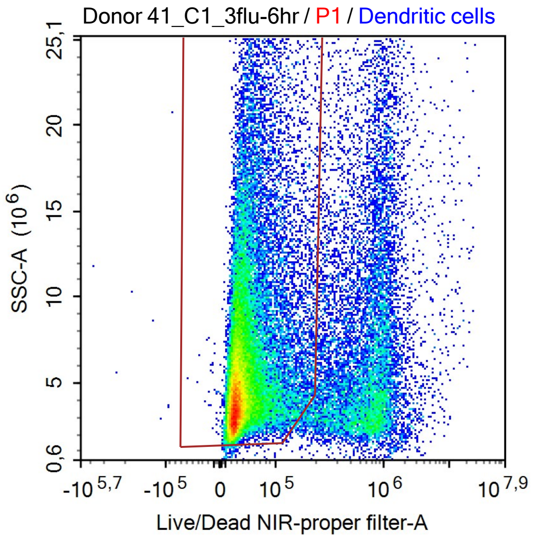

Gate	Count	% Dendritic cells
Dendritic cells	50.573	100,00%
Live	38.209	75,55%

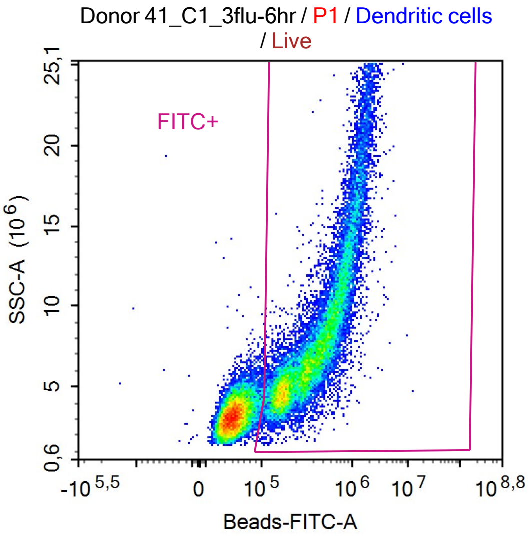

Gate	Count	% Live
Live	38.209	100,00%
FITC+	23.826	62,36%

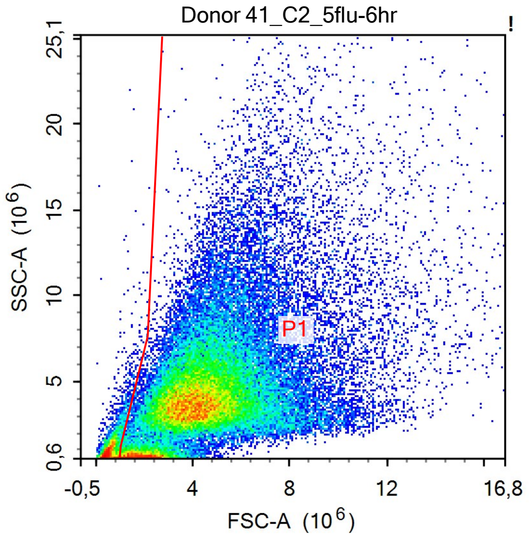

Gate	Count	% All
All	165.326	100,00%
P1	76.783	46,44%

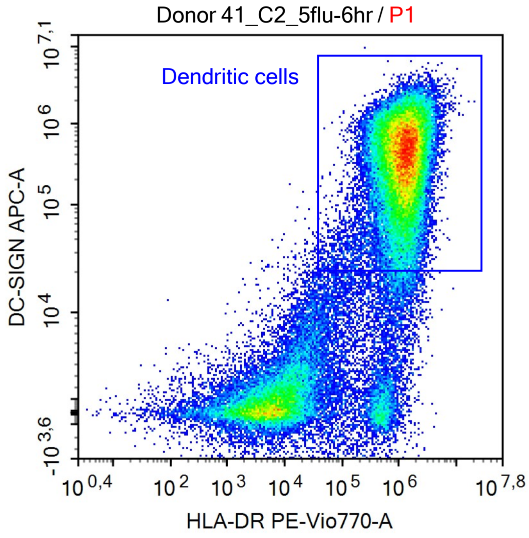

Gate	Count	% P1
P1	76.783	100,00%
Dendritic cells	47.611	62,01%

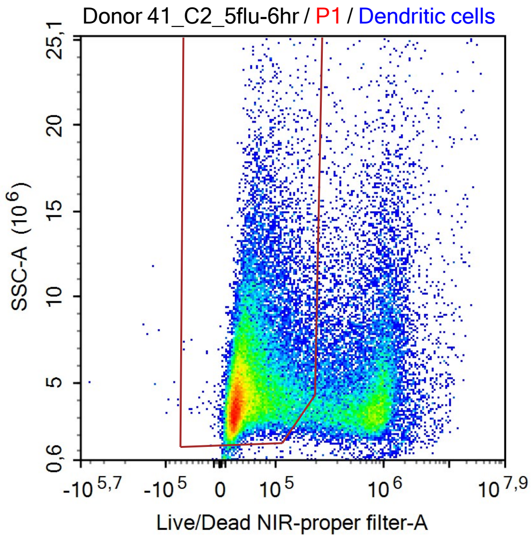

Gate	Count	% Dendritic cells
Dendritic cells	47.611	100,00%
Live	32.444	68,14%

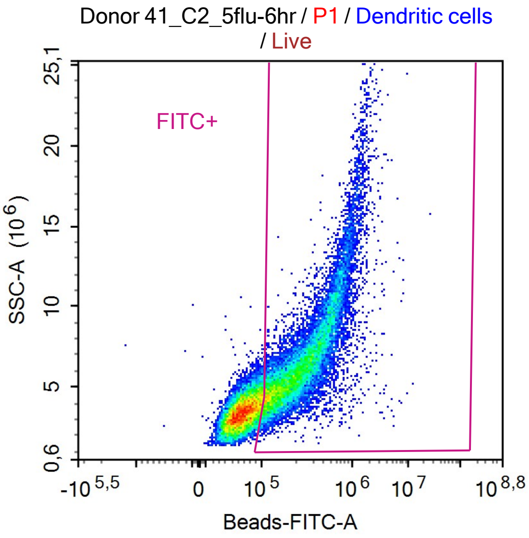

Gate	Count	% Live
Live	32.444	100,00%
FITC+	17.354	53,49%

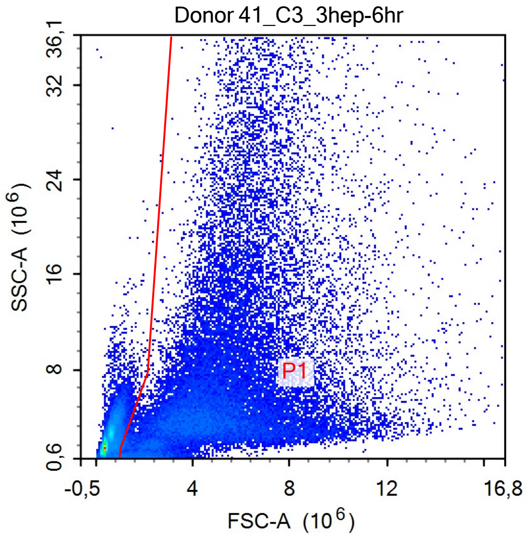

Gate	Count	% All
All	286.750	100,00%
P1	42.781	14,92%

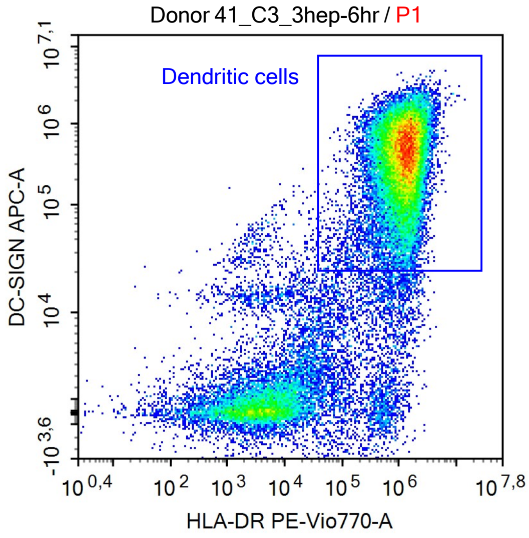

Gate	Count	% P1
P1	42.781	100,00%
Dendritic cells	27.362	63,96%

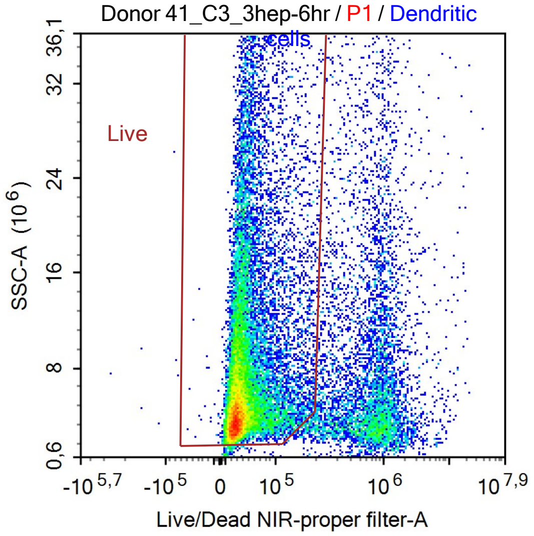

Gate	Count	% Dendritic cells
Dendritic cells	27.362	100,00%
Live	20.978	76,67%

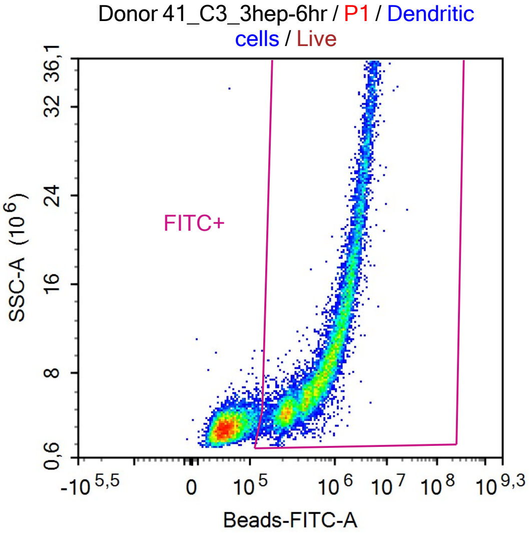

Gate	Count	% Live
Live	20.978	100,00%
FITC+	14.122	67,32%

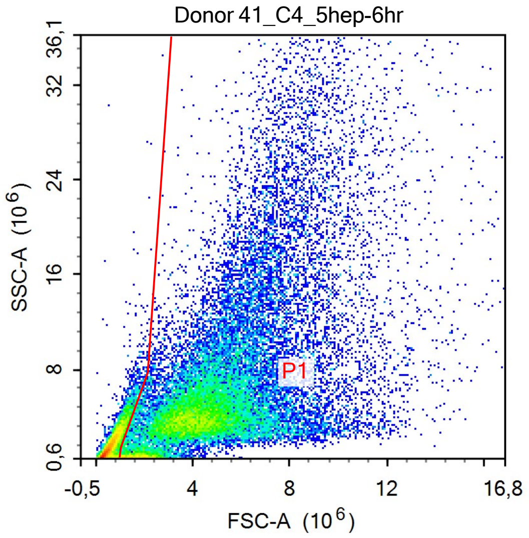

Gate	Count	% All
All	109.610	100,00%
P1	38.450	35,08%

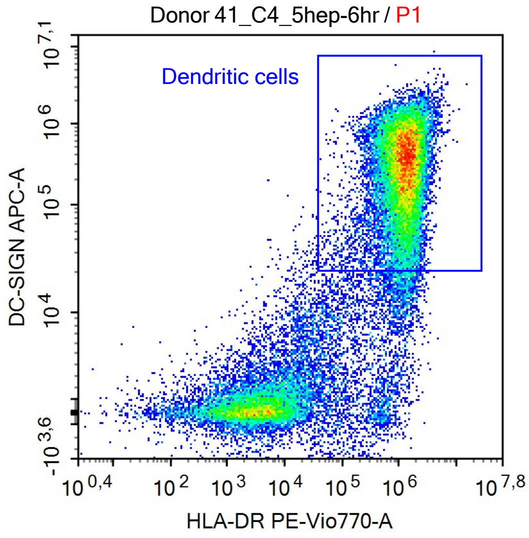

Gate	Count	% P1
P1	38.450	100,00%
Dendritic cells	22.233	57,82%

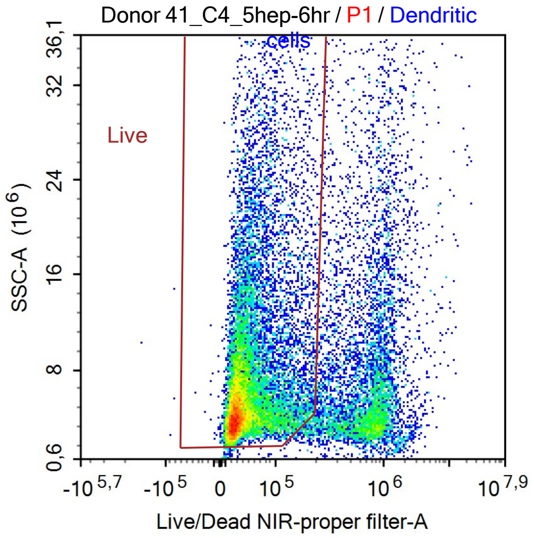

Gate	Count	% Dendritic cells
Dendritic cells	22.233	100,00%
Live	15.764	70,90%

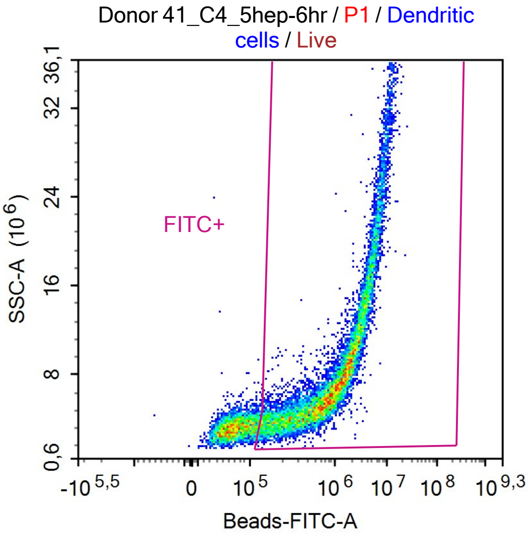

Gate	Count	% Live
Live	15.764	100,00%
FITC+	12.157	77,12%

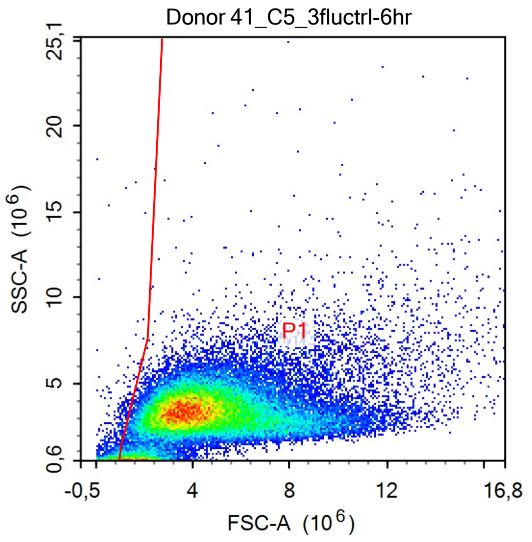

Gate	Count	% All
All	128.407	100,00%
P1	68.169	53,09%

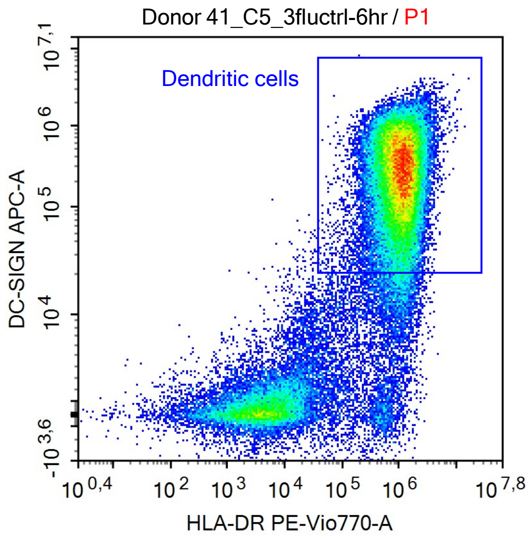

Gate	Count	% P1
P1	68.169	100,00%
Dendritic cells	45.622	66,92%

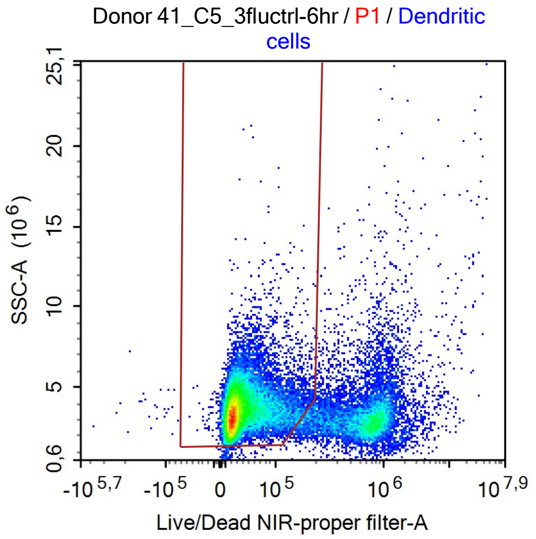

Gate	Count	% Dendritic cells
Dendritic cells	45.622	100,00%
Live	35.635	78,11%

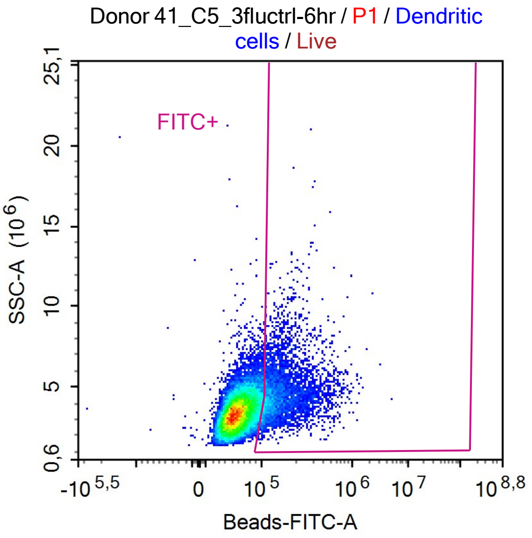

Gate	Count	% Live
Live	35.635	100,00%
FITC+	4.791	13,44%

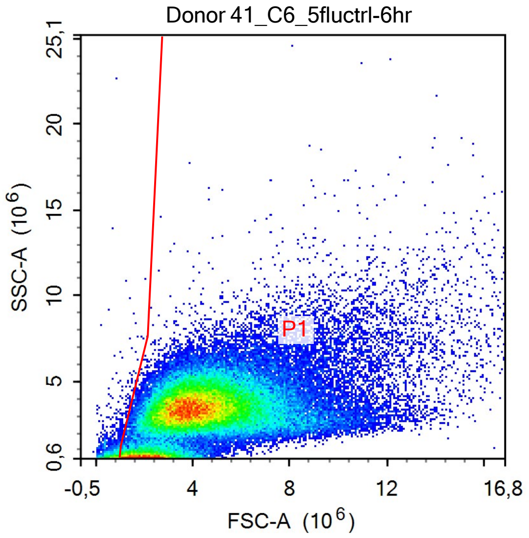

Gate	Count	% All
All	231.064	100,00%
P1	123.309	53,37%

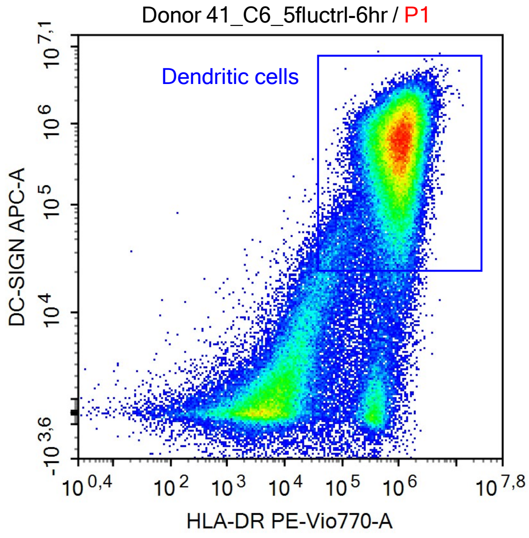

Gate	Count	% P1
P1	123.309	100,00%
Dendritic cells	72.491	58,79%

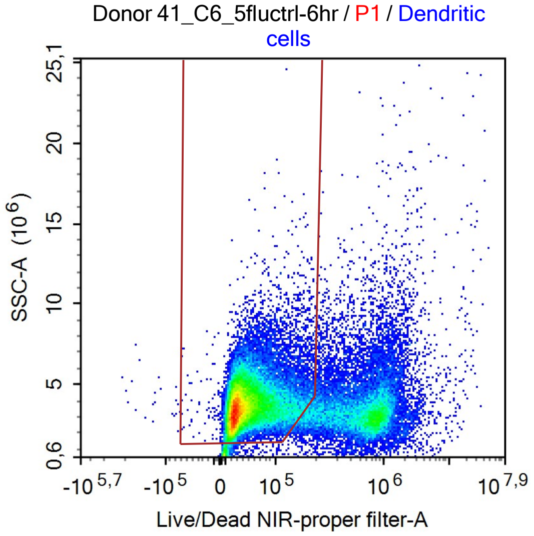

Gate	Count	% Dendritic cells
Dendritic cells	72.491	100,00%
Live	52.039	71,79%

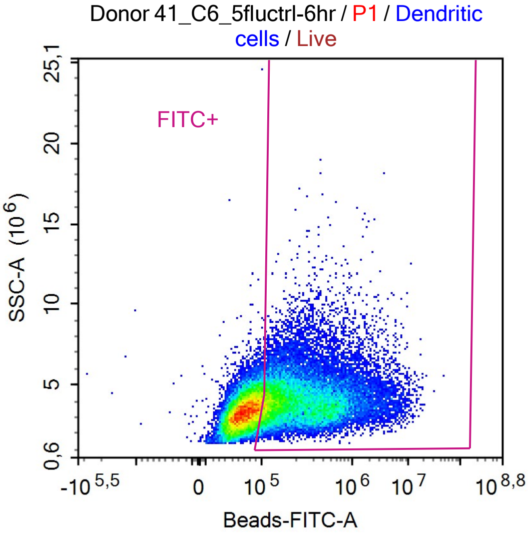

Gate	Count	% Live
Live	52.039	100,00%
FITC+	26.248	50,44%

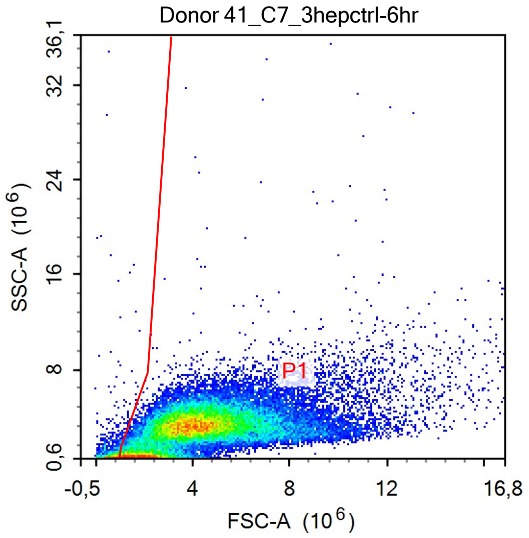

Gate	Count	% All
All	137.421	100,00%
P1	68.452	49,81%

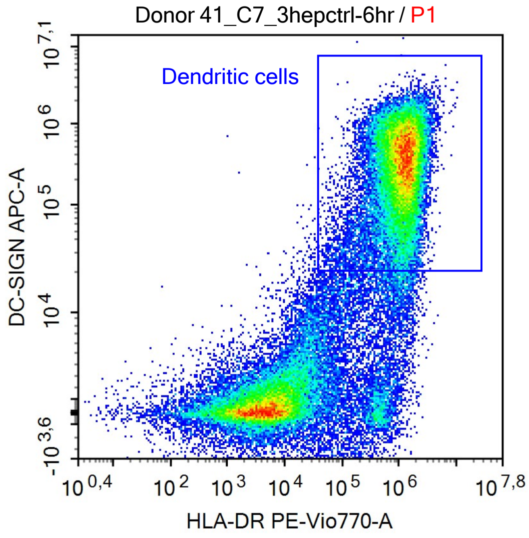

Gate	Count	% P1
P1	68.452	100,00%
Dendritic cells	31.129	45,48%

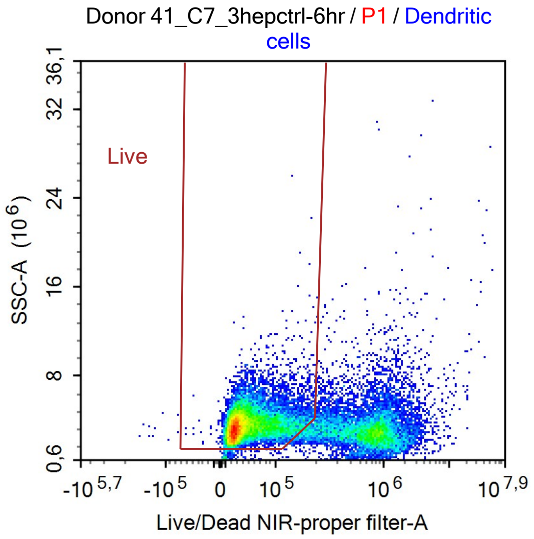

Gate	Count	% Dendritic cells
Dendritic cells	31.129	100,00%
Live	20.316	65,26%

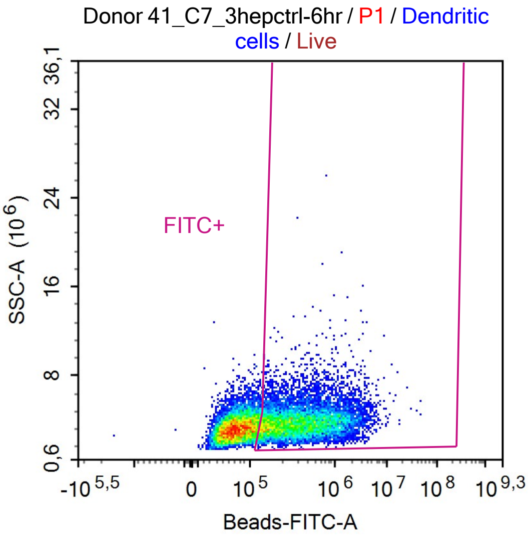

Gate	Count	% Live
Live	20.316	100,00%
FITC+	10.267	50,54%

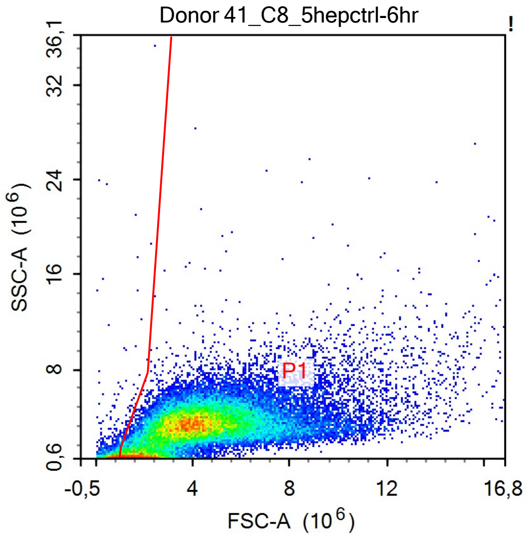

Gate	Count	% All
All	123.556	100,00%
P1	59.958	48,53%

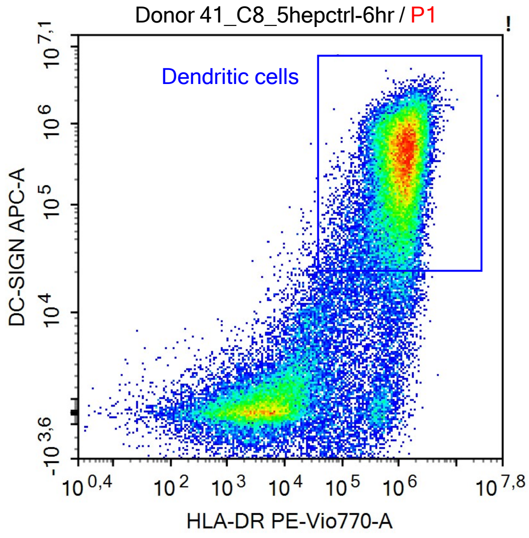

Gate	Count	% P1
P1	59.958	100,00%
Dendritic cells	30.856	51,46%

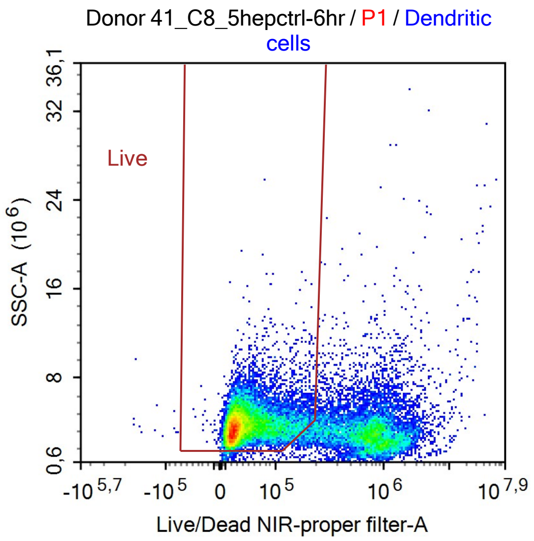

Gate	Count	% Dendritic cells
Dendritic cells	30.856	100,00%
Live	20.336	65,91%

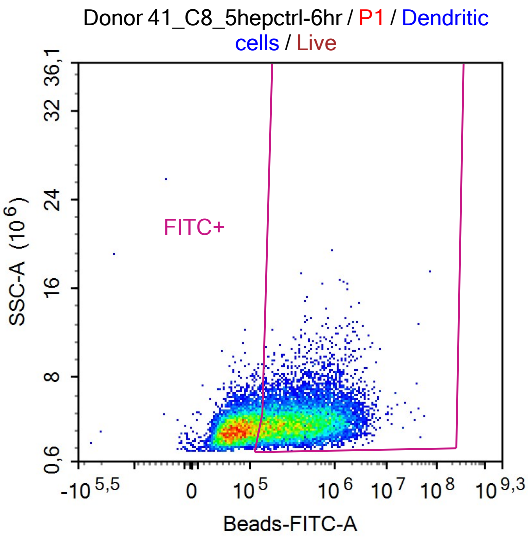

Gate	Count	% Live
Live	20.336	100,00%
FITC+	11.399	56,05%

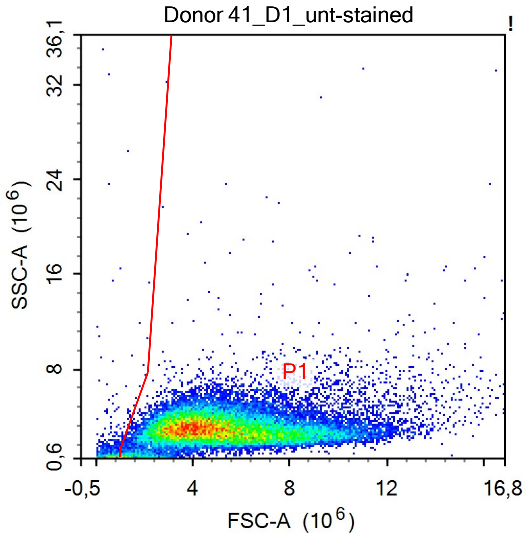

Gate	Count	% All
All	75.255	100,00%
P1	33.684	44,76%

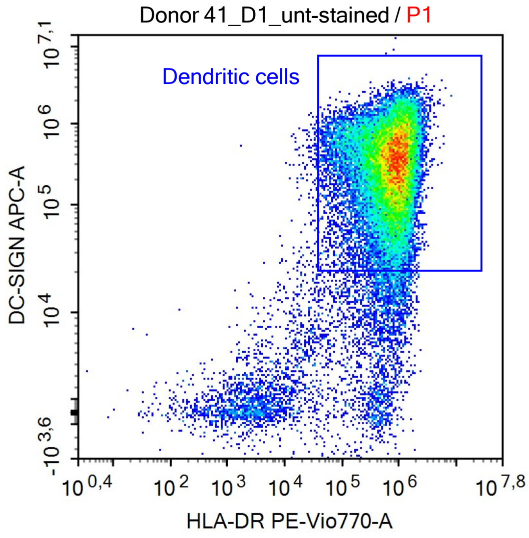

Gate	Count	% P1
P1	33.684	100,00%
Dendritic cells	28.541	84,73%

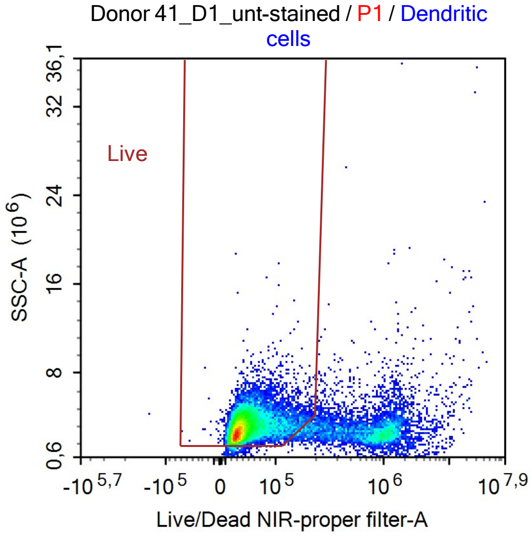

Gate	Count	% Dendritic cells
Dendritic cells	28.541	100,00%
Live	22.672	79,44%

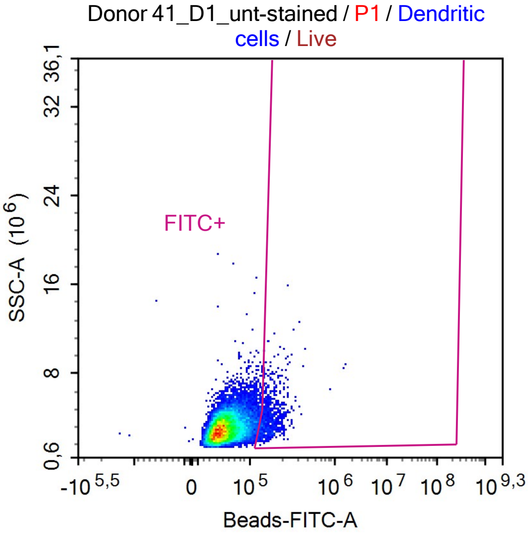

Gate	Count	% Live
Live	22.672	100,00%
FITC+	653	2,88%

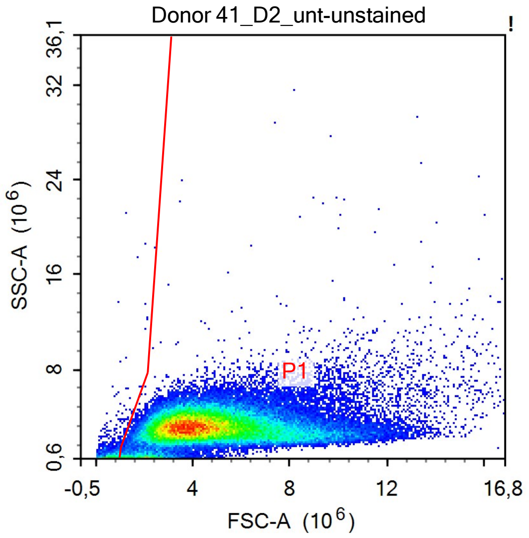

Gate	Count	% All
All	144.242	100,00%
P1	80.192	55,60%

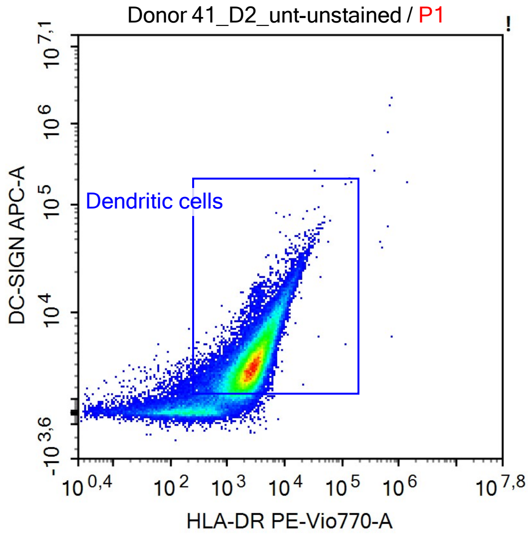

Gate	Count	% P1
P1	80.192	100,00%
Dendritic cells	63.025	78,59%

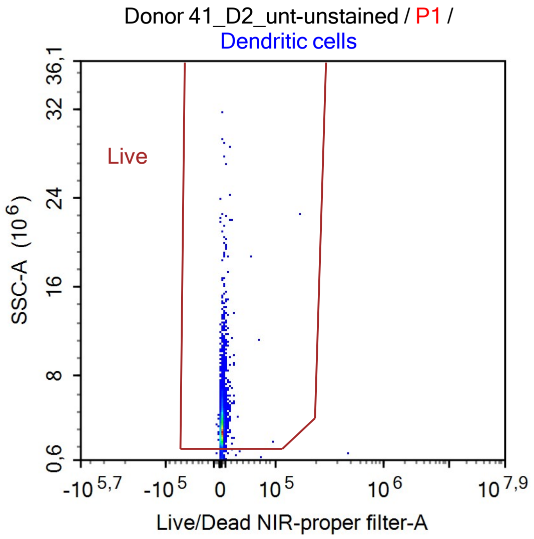

Gate	Count	% Dendritic cells
Dendritic cells	63.025	100,00%
Live	61.139	97,01%

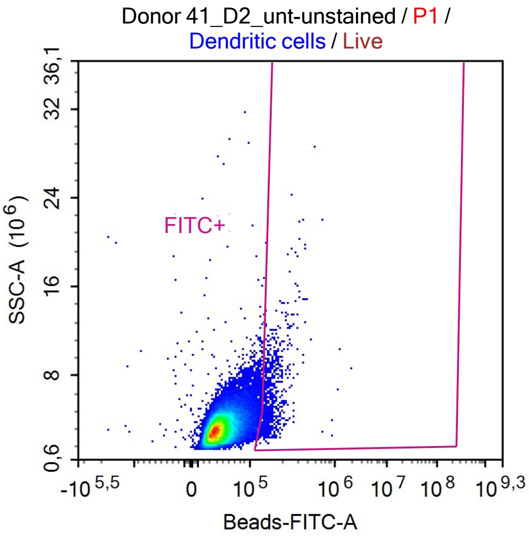

Gate	Count	% Live
Live	61.139	100,00%
FITC+	628	1,03%

Donor 41 FLU

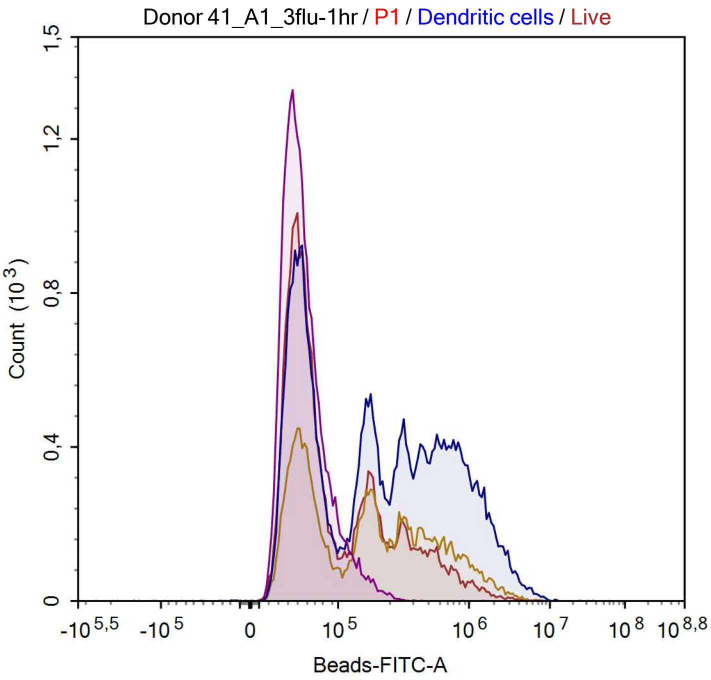

#	Sample	Gate	Count	Mean X	Median X
1	Donor 41_A1_3flu-1hr	Live	23.634	166.437	66.929
2	Donor 41_B1_3flu-3hr	Live*	17.286	305.275	141.863
3	Donor 41_C1_3flu-6hr	Live*	38.209	438.813	163.632
4	Donor 41_D1_unt-[illegible]	Live*	22.672	58.710	51.792

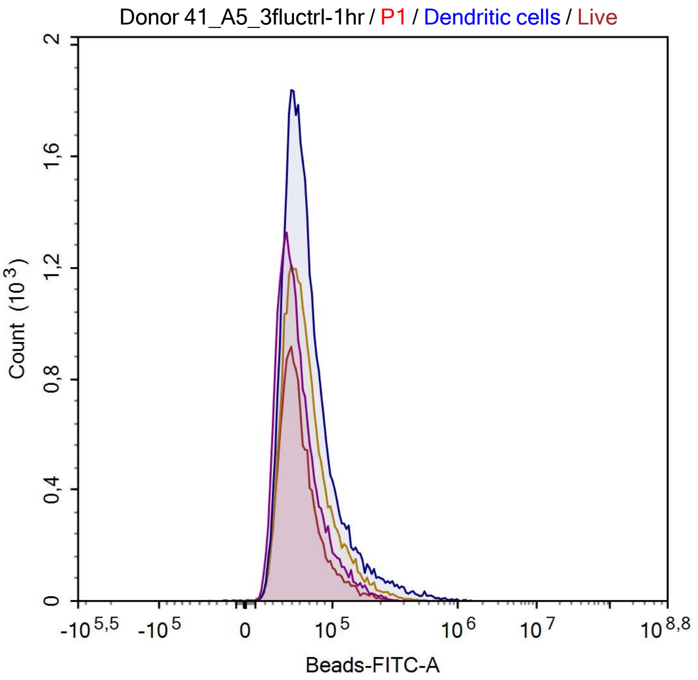

#	Sample	Gate	Count	Mean X	Median X
1	Donor 41_A5_3fluctrl-1hr	Live	14.842	60.712	54.550
2	Donor 41_B5_3fluctrl-3hr	Live*	23.919	70.551	61.224
3	Donor 41_C5_3fluctrl-6hr	Live*	35.635	76.946	61.943
4	Donor 41_D1_unt-[illegible]	Live*	22.672	58.710	51.792

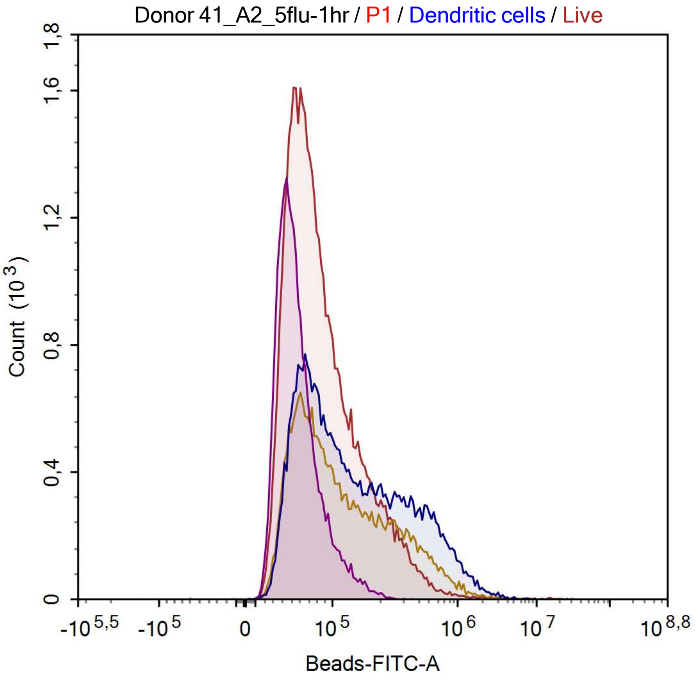

#	Sample	Gate	Count	Mean X	Median X
1	Donor 41_A2_5flu-1hr	Live	46.372	119.571	75.532
2	Donor 41_B2_5flu-3hr	Live*	24.534	184.427	98.399
3	Donor 41_C2_5flu-6hr	Live*	32.444	235.225	114.565
4	Donor 41_D1_unt-[illegible]	Live*	22.672	58.710	51.792

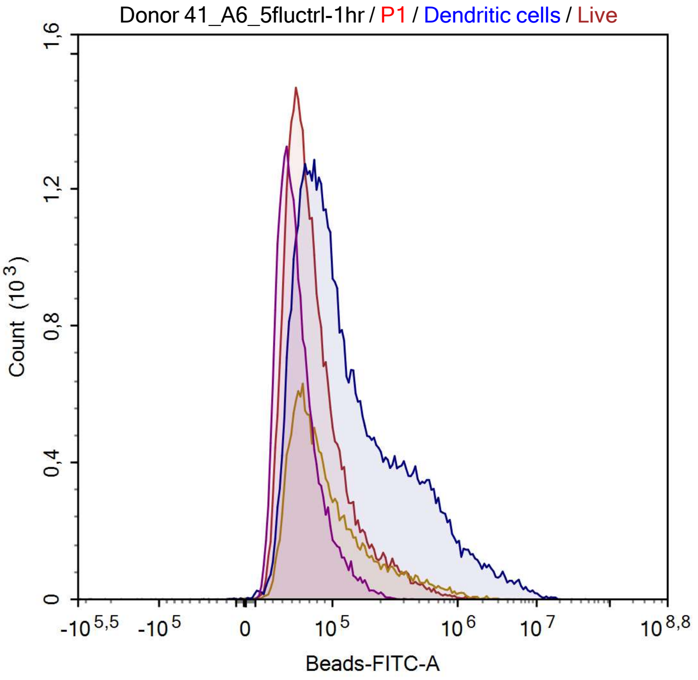

#	Sample	Gate	Count	Mean X	Median X
1	Donor 41_A6_5fluctrl-1hr	Live	33.745	89.161	67.704
2	Donor 41_B6_5fluctrl-3hr	Live*	16.835	130.223	80.472
3	Donor 41_C6_5fluctrl-6hr	Live*	52.039	317.900	105.272
4	Donor 41_D1_unt-[illegible]	Live*	22.672	58.710	51.792

Donor 41 HEP

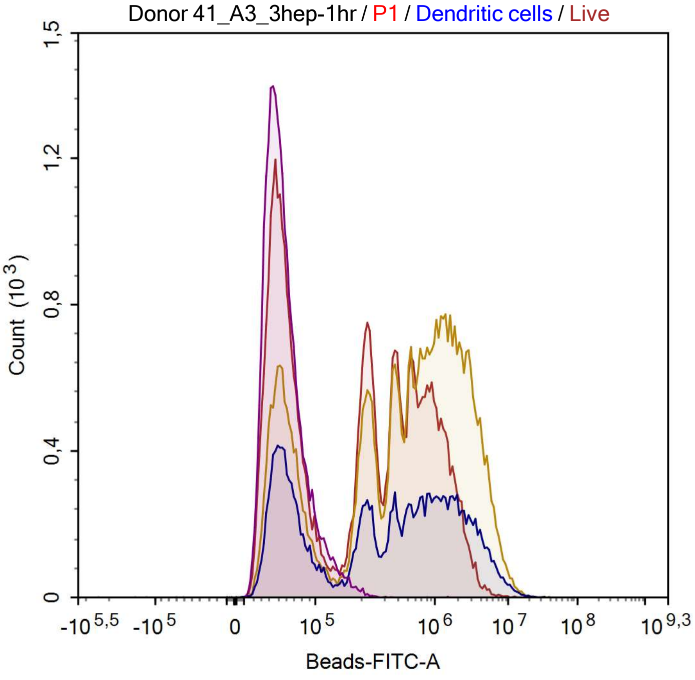

#	Sample	Gate	Count	Mean X	Median X
1	Donor 41_A3_3hep-1hr	Live	43.371	458.628	213.817
2	Donor 41_B3_3hep-3hr	Live*	45.130	1.241.238	564.413
3	Donor 41_C3_3hep-6hr	Live*	20.978	1.072.366	360.326
4	Donor 41_D1_unt-[illegible]	Live*	22.672	58.710	51.792

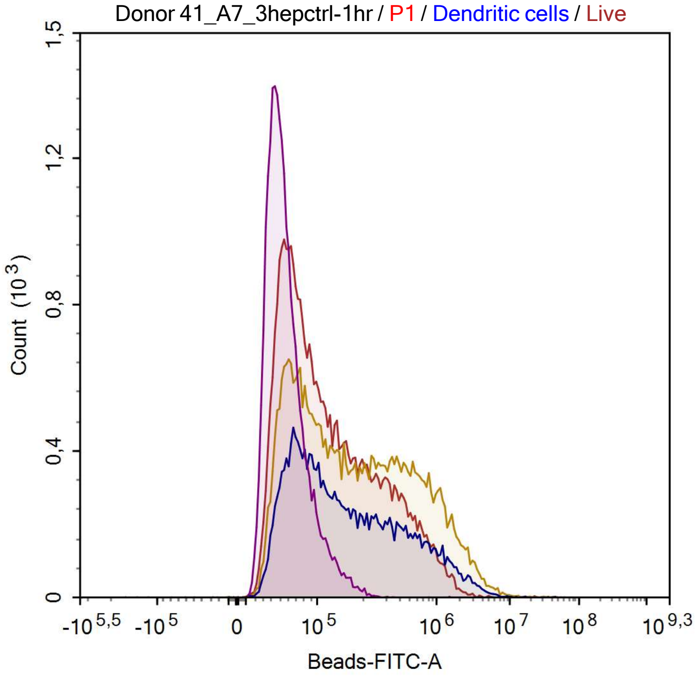

#	Sample	Gate	Count	Mean X	Median X
1	Donor 41_A7_3hepctrl-1hr	Live	35.329	207.910	99.927
2	Donor 41_B7_3hepctrl-3hr	Live*	34.231	445.877	153.892
3	Donor 41_C7_3hepctrl-6hr	Live*	20.316	357.293	131.236
4	Donor 41_D1_unt-[illegible]	Live*	22.672	58.710	51.792

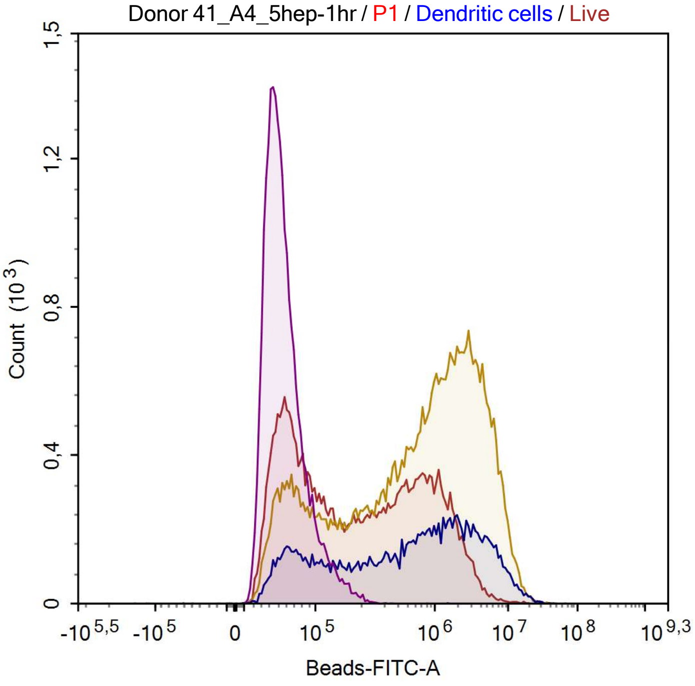

#	Sample	Gate	Count	Mean X	Median X
1	Donor 41_A4_5hep-1hr	Live	28.309	534.985	180.202
2	Donor 41_B4_5hep-3hr	Live*	41.972	1.888.229	818.344
3	Donor 41_C4_5hep-6hr	Live*	15.764	1.852.649	611.403
4	Donor 41_D1_unt-[illegible]	Live*	22.672	58.710	51.792

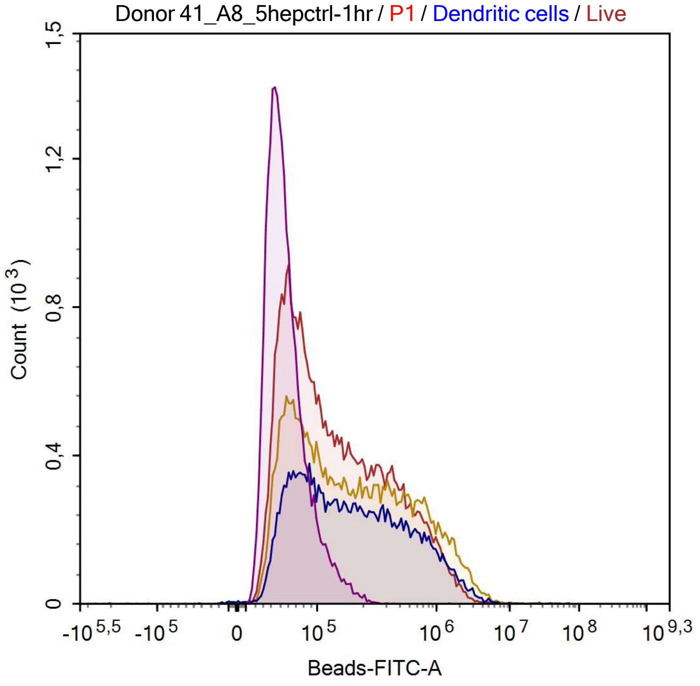

#	Sample	Gate	Count	Mean X	Median X
1	Donor 41_A8_5hepctrl-1hr	Live	35.273	250.852	111.908
2	Donor 41_B8_5hepctrl-3hr	Live*	28.439	404.303	148.545
3	Donor 41_C8_5hepctrl-6hr	Live*	20.336	363.463	152.320
4	Donor 41_D1_unt-[illegible]	Live*	22.672	58.710	51.792
