# Supplementary material for: Assessing the Immunomodulatory Effect of Size on the Uptake and Immunogenicity of Influenza- and Hepatitis B Subunit Vaccines In Vitro
Source: Pharmaceuticals (Basel). 2022 Jul 18;15(7):887. doi: 10.3390/ph15070887 (PMC9321264; doi:10.3390/ph15070887)
Supplement: Supplementary file 1 [file pharmaceuticals-15-00887-s001.zip › Supplementary file S3.pdf]

Report of Donor 45

Specimen Name: Donor 45 | Run Time: 28-1-2022 10:32

Cytometer: NovoCyte Quanteon 621200611403 | Software: NovoExpress 1.5.6

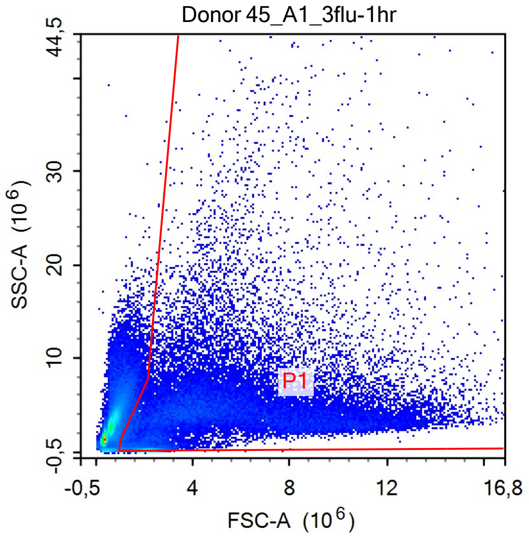

Gate	Count	% All
All	281.446	100,00%
P1	34.976	12,43%

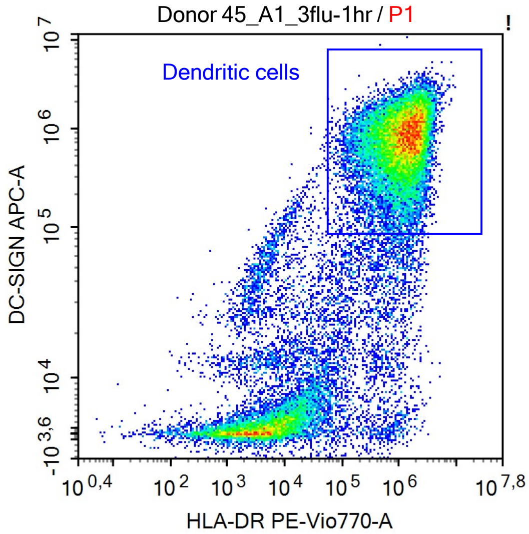

Gate	Count	% P1
P1	34.976	100,00%
Dendritic cells	20.266	57,94%

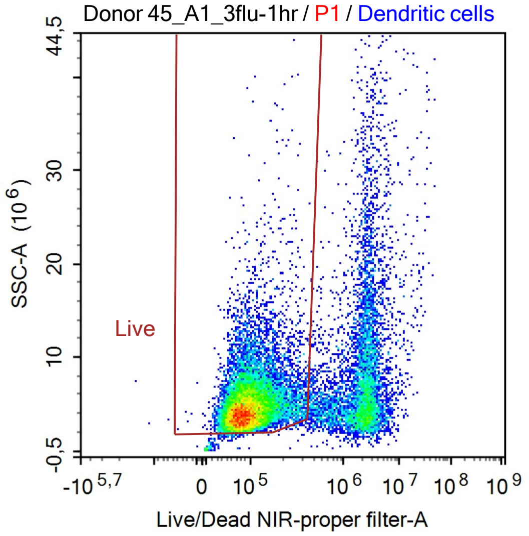

Gate	Count	% Dendritic cells
Dendritic cells	20.266	100,00%
Live	14.332	70,72%

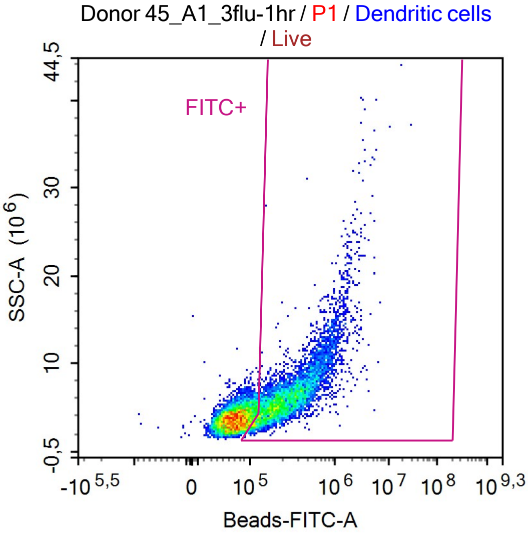

Gate	Count	% Live
Live	14.332	100,00%
FITC+	6.710	46,82%

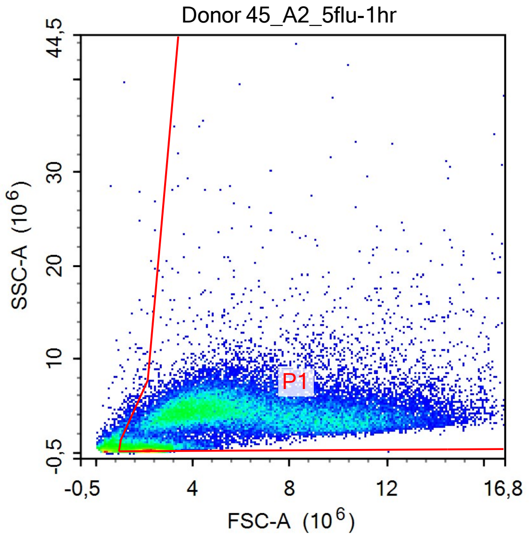

Gate	Count	% All
All	63.900	100,00%
P1	50.451	78,95%

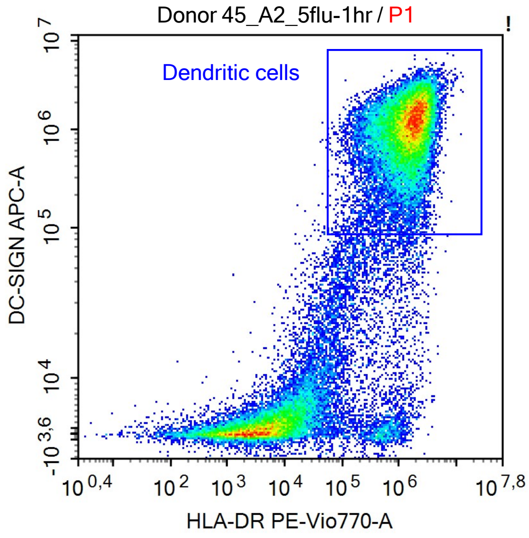

Gate	Count	% P1
P1	50.451	100,00%
Dendritic cells	26.998	53,51%

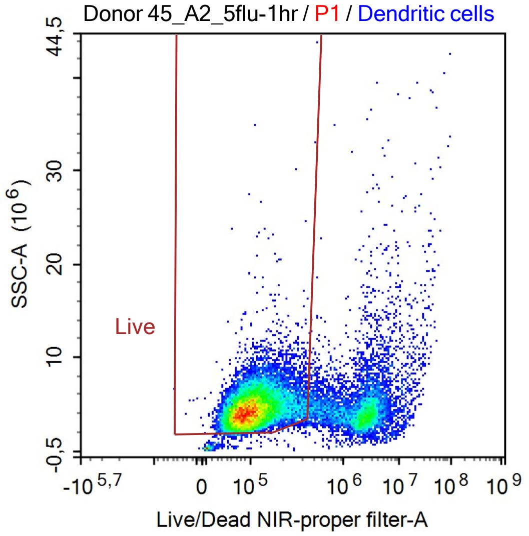

Gate	Count	% Dendritic cells
Dendritic cells	26.998	100,00%
Live	20.481	75,86%

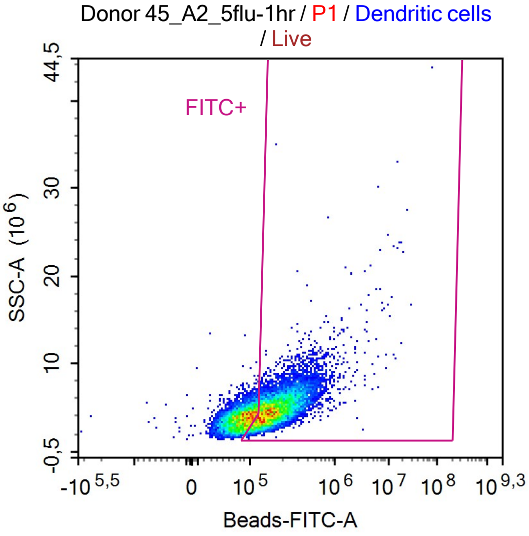

Gate	Count	% Live
Live	20.481	100,00%
FITC+	11.605	56,66%

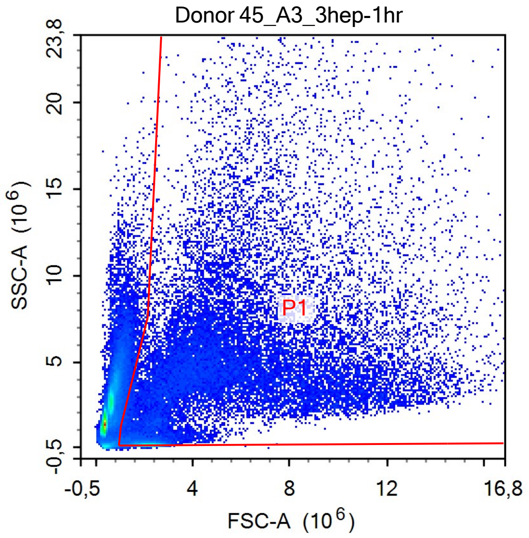

Gate	Count	% All
All	255.755	100,00%
P1	36.758	14,37%

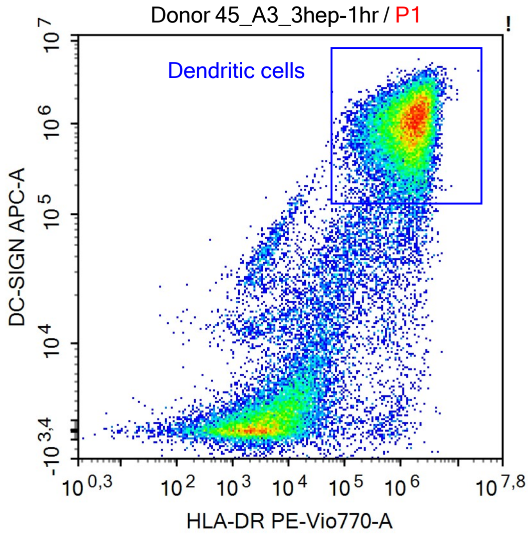

Gate	Count	% P1
P1	36.758	100,00%
Dendritic cells	17.695	48,14%

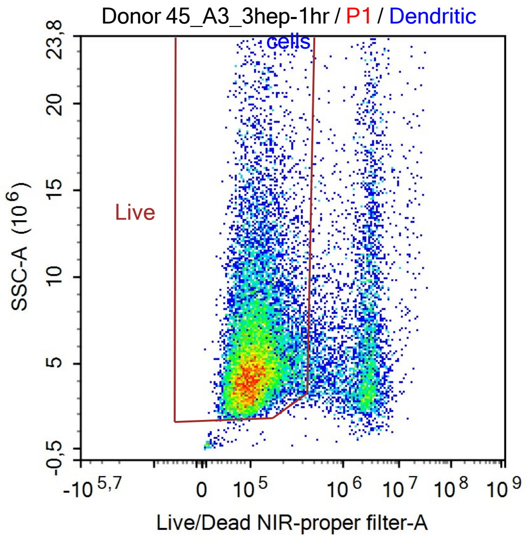

Gate	Count	% Dendritic cells
Dendritic cells	17.695	100,00%
Live	13.541	76,52%

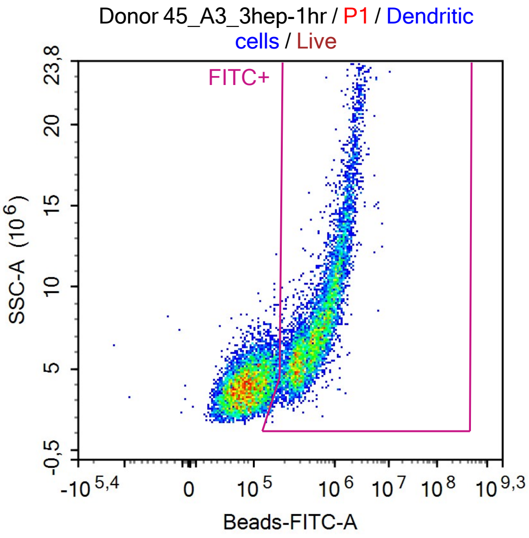

Gate	Count	% Live
Live	13.541	100,00%
FITC+	7.169	52,94%

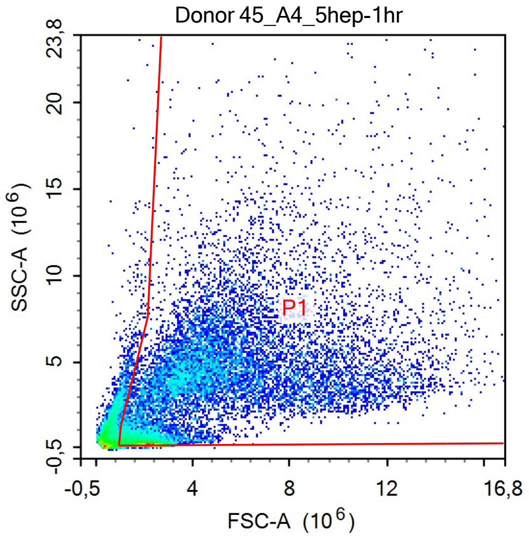

Gate	Count	% All
All	38.761	100,00%
P1	25.881	66,77%

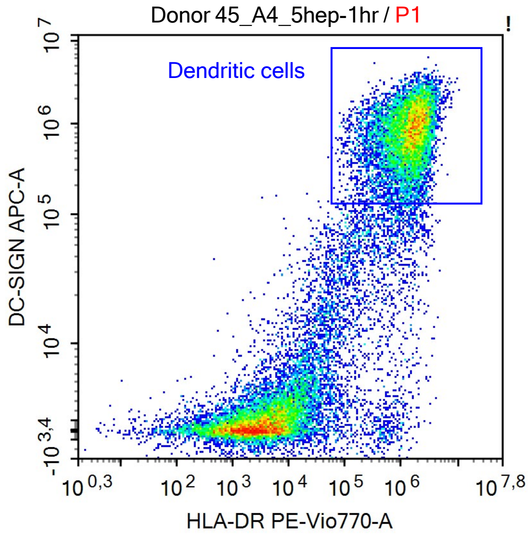

Gate	Count	% P1
P1	25.881	100,00%
Dendritic cells	9.117	35,23%

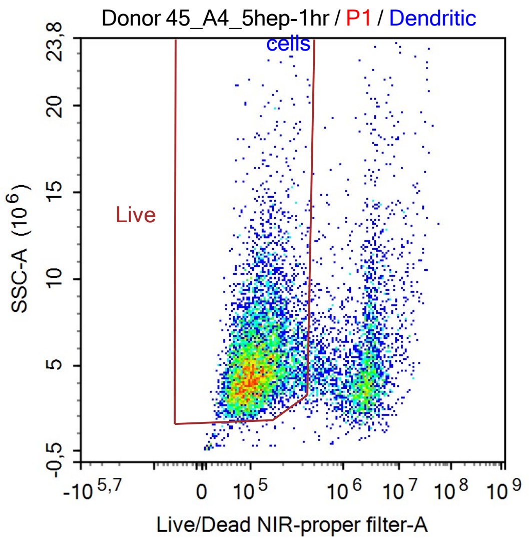

Gate	Count	% Dendritic cells
Dendritic cells	9.117	100,00%
Live	6.356	69,72%

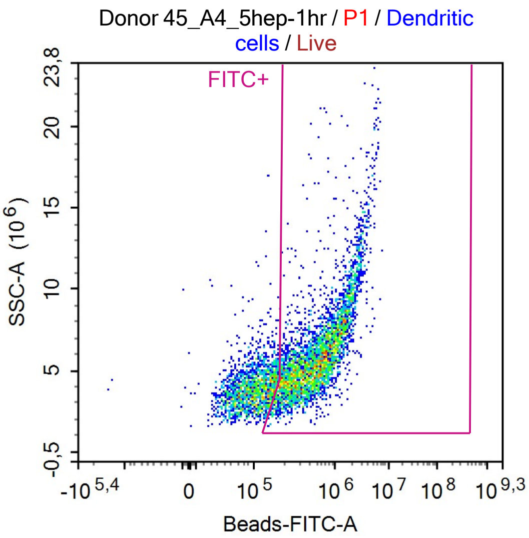

Gate	Count	% Live
Live	6.356	100,00%
FITC+	4.214	66,30%

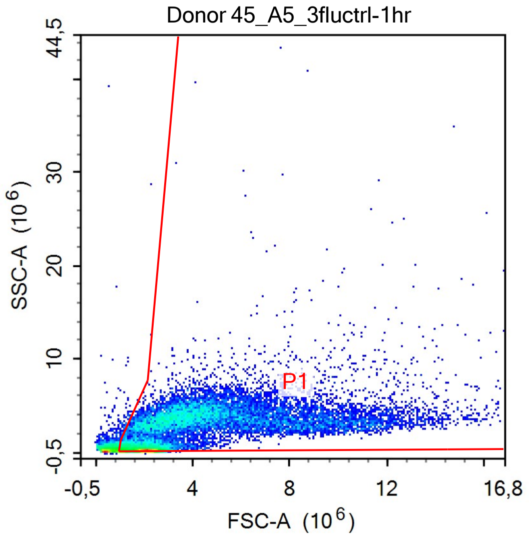

Gate	Count	% All
All	33.043	100,00%
P1	23.100	69,91%

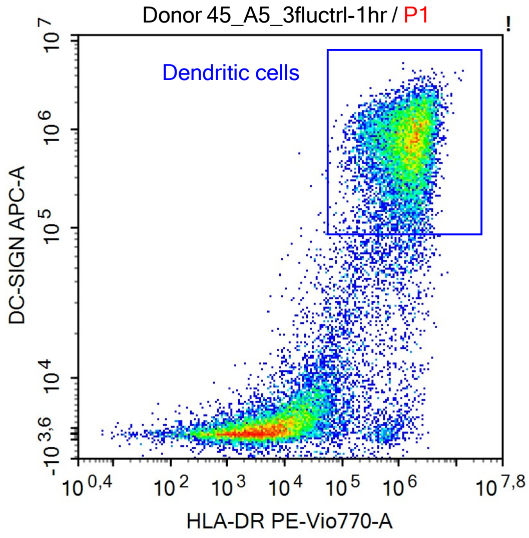

Gate	Count	% P1
P1	23.100	100,00%
Dendritic cells	9.429	40,82%

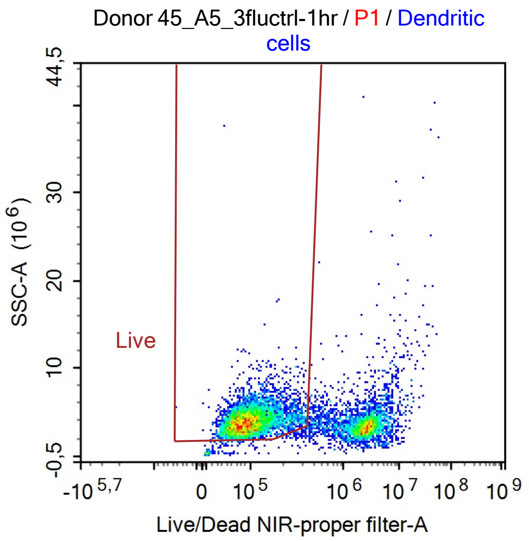

Gate	Count	% Dendritic cells
Dendritic cells	9.429	100,00%
Live	6.119	64,90%

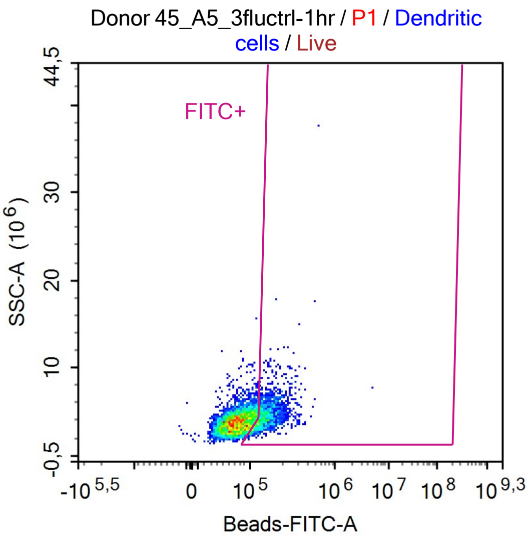

Gate	Count	% Live
Live	6.119	100,00%
FITC+	1.292	21,11%

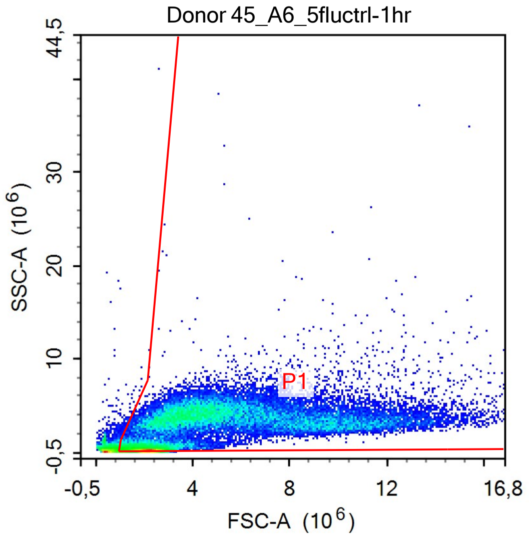

Gate	Count	% All
All	55.318	100,00%
P1	42.654	77,11%

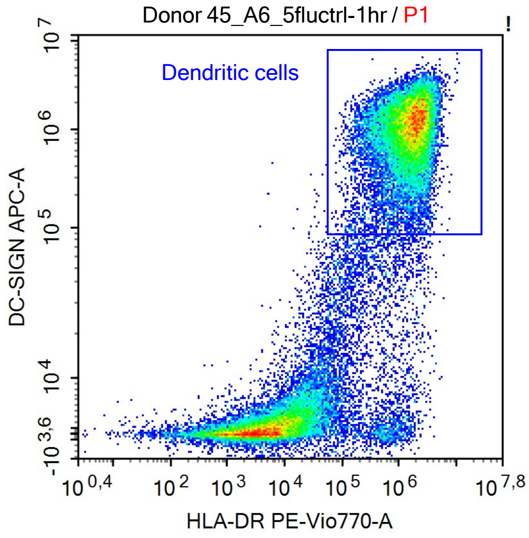

Gate	Count	% P1
P1	42.654	100,00%
Dendritic cells	20.355	47,72%

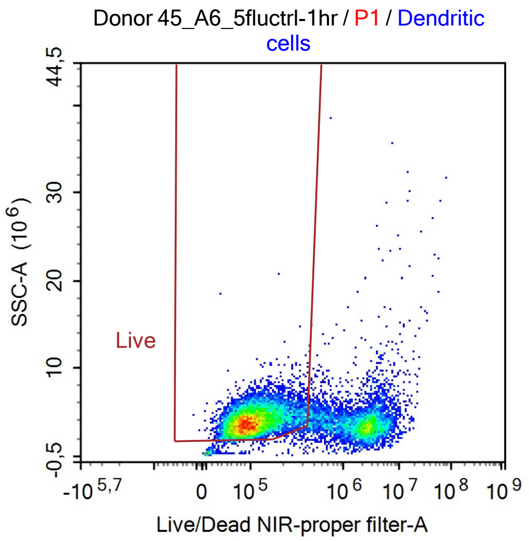

Gate	Count	% Dendritic cells
Dendritic cells	20.355	100,00%
Live	14.129	69,41%

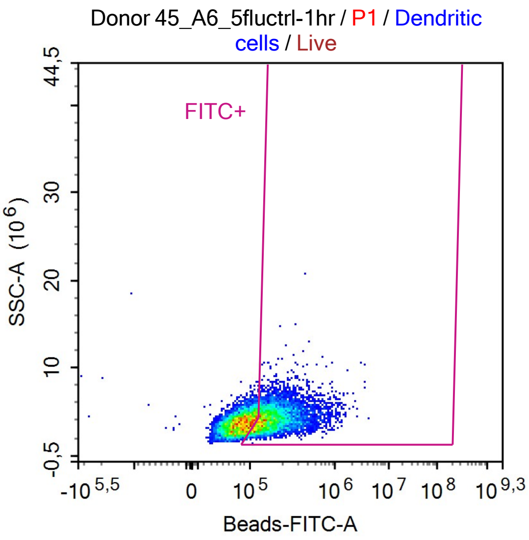

Gate	Count	% Live
Live	14.129	100,00%
FITC+	6.309	44,65%

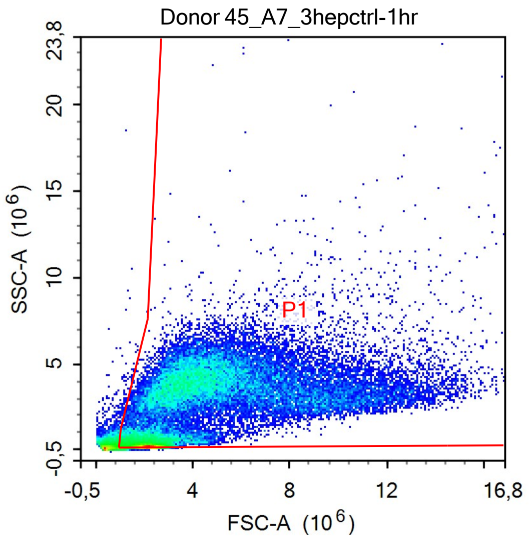

Gate	Count	% All
All	67.630	100,00%
P1	53.241	78,72%

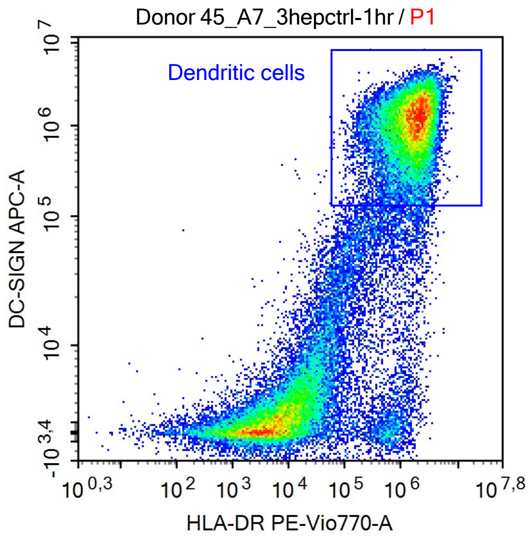

Gate	Count	% P1
P1	53.241	100,00%
Dendritic cells	22.047	41,41%

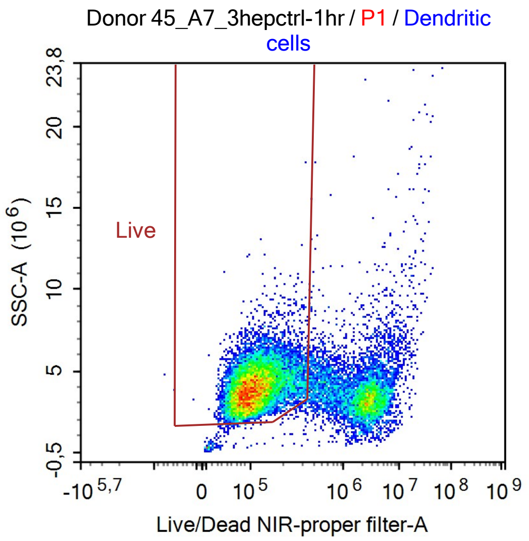

Gate	Count	% Dendritic cells
Dendritic cells	22.047	100,00%
Live	14.856	67,38%

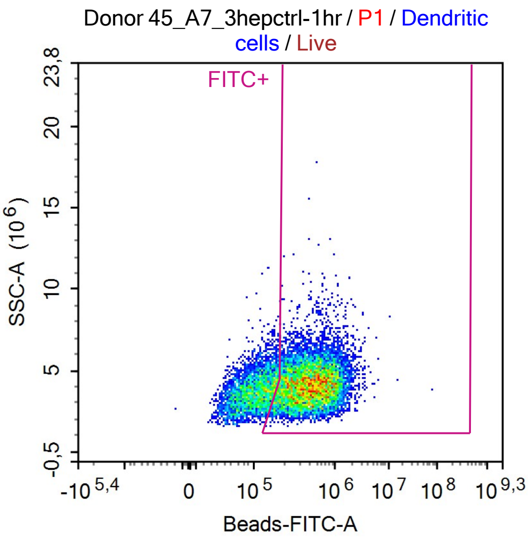

Gate	Count	% Live
Live	14.856	100,00%
FITC+	10.125	68,15%

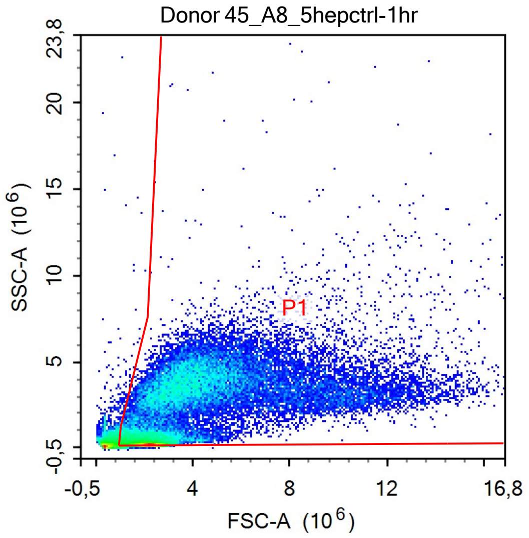

Gate	Count	% All
All	65.869	100,00%
P1	48.764	74,03%

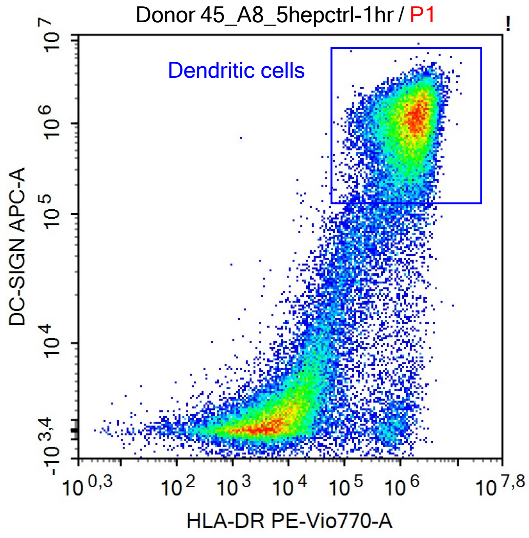

Gate	Count	% P1
P1	48.764	100,00%
Dendritic cells	18.714	38,38%

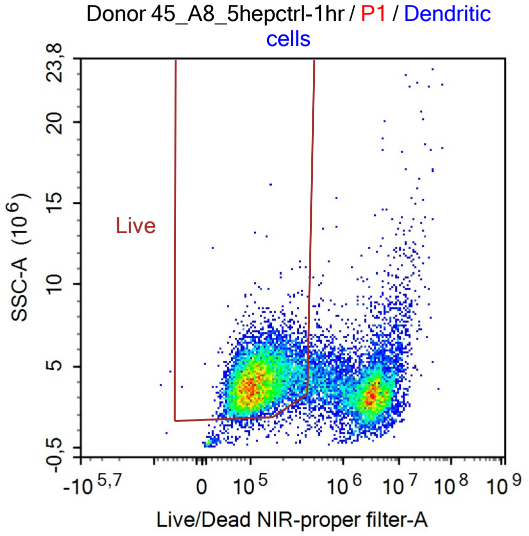

Gate	Count	% Dendritic cells
Dendritic cells	18.714	100,00%
Live	10.744	57,41%

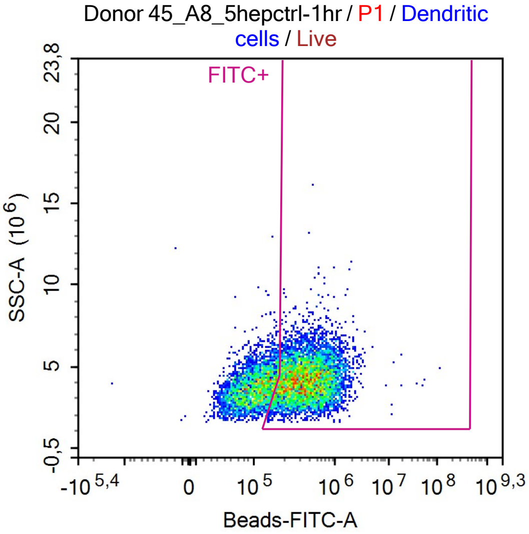

Gate	Count	% Live
Live	10.744	100,00%
FITC+	6.749	62,82%

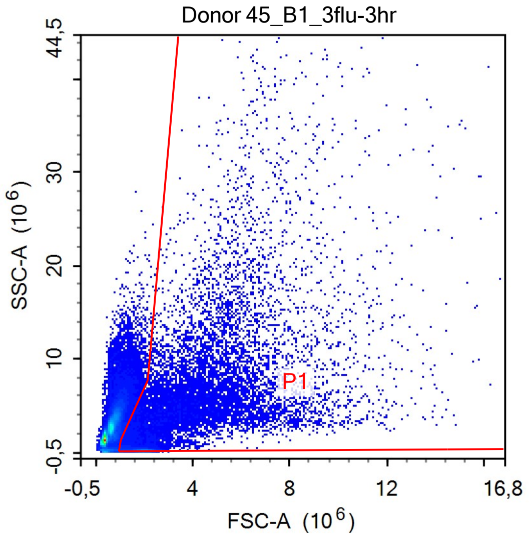

Gate	Count	% All
All	314.426	100,00%
P1	16.694	5,31%

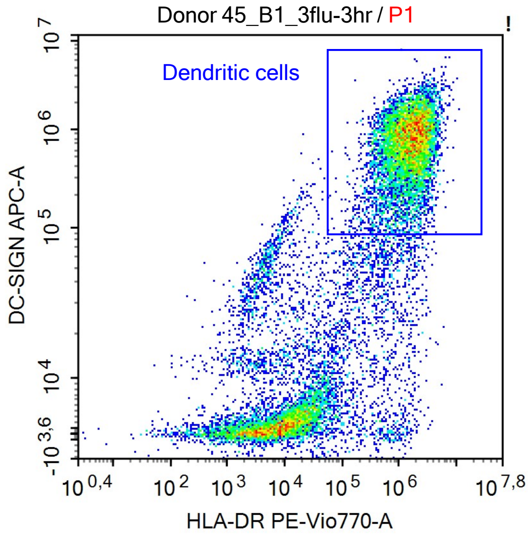

Gate	Count	% P1
P1	16.694	100,00%
Dendritic cells	7.790	46,66%

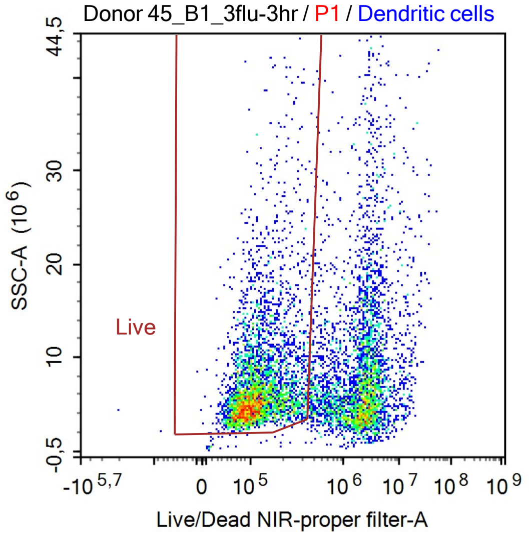

Gate	Count	% Dendritic cells
Dendritic cells	7.790	100,00%
Live	4.099	52,62%

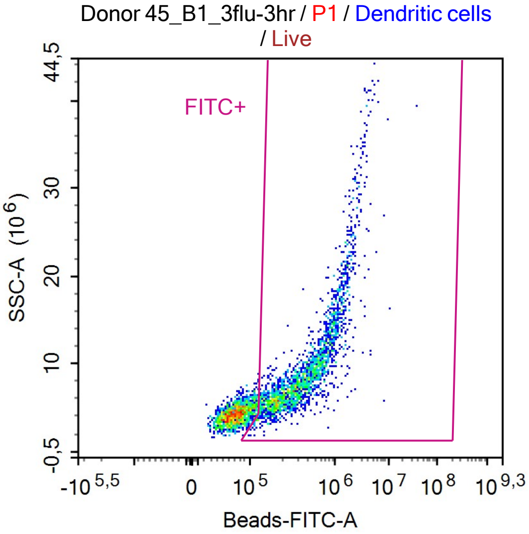

Gate	Count	% Live
Live	4.099	100,00%
FITC+	2.377	57,99%

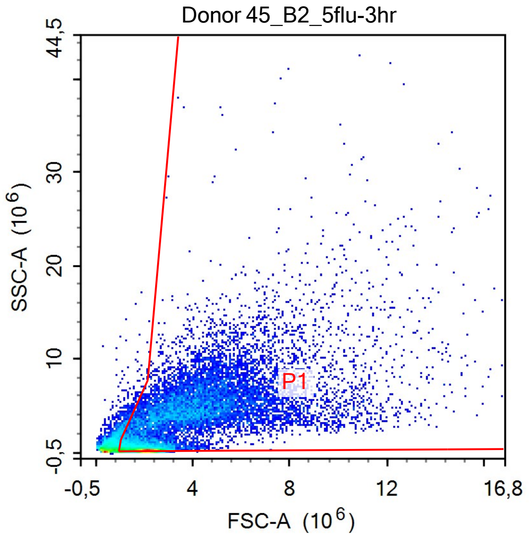

Gate	Count	% All
All	45.411	100,00%
P1	30.479	67,12%

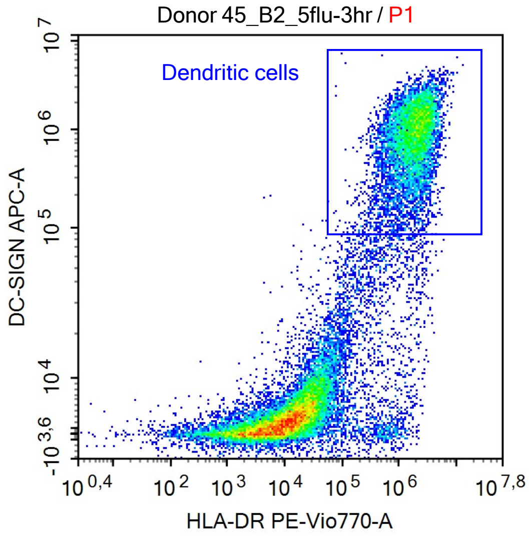

Gate	Count	% P1
P1	30.479	100,00%
Dendritic cells	8.129	26,67%

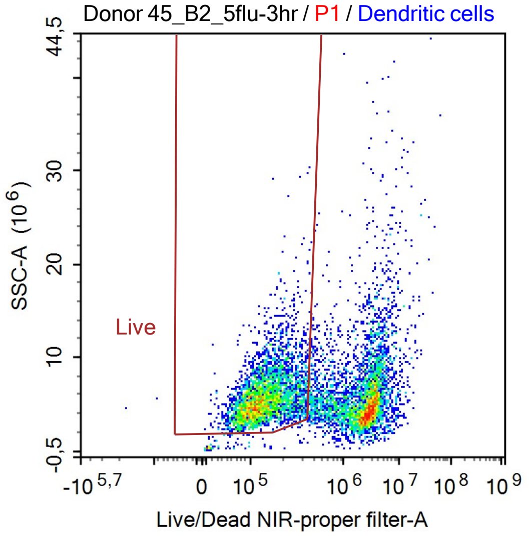

Gate	Count	% Dendritic cells
Dendritic cells	8.129	100,00%
Live	3.915	48,16%

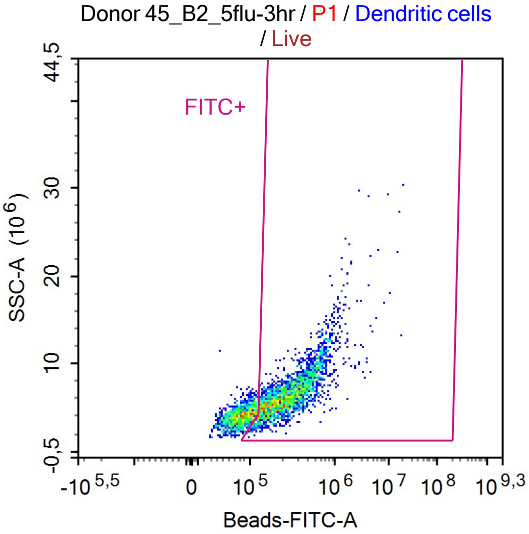

Gate	Count	% Live
Live	3.915	100,00%
FITC+	2.610	66,67%

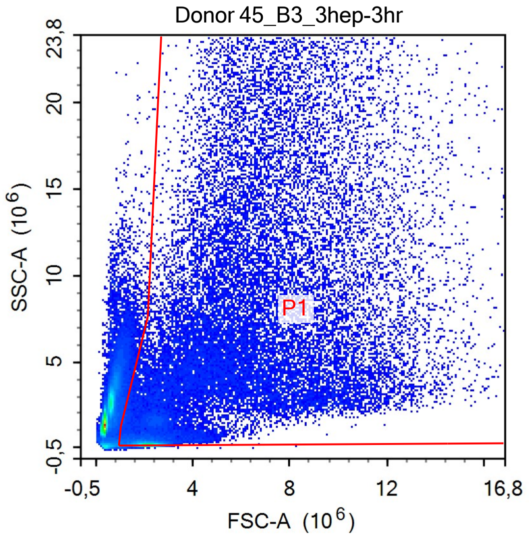

Gate	Count	% All
All	348.419	100,00%
P1	65.222	18,72%

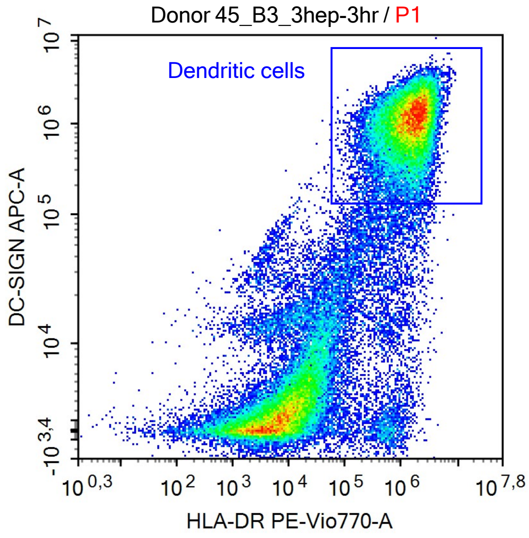

Gate	Count	% P1
P1	65.222	100,00%
Dendritic cells	25.798	39,55%

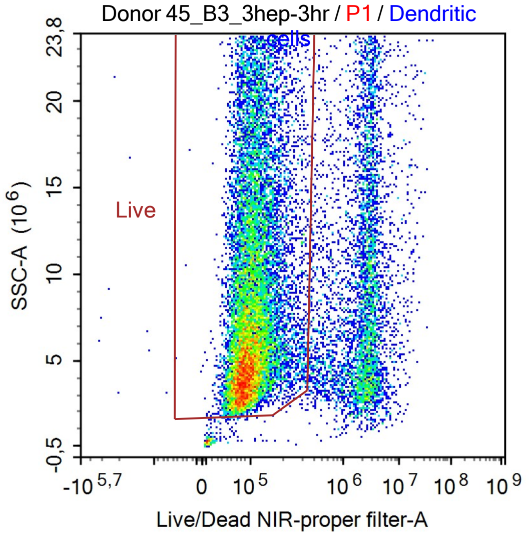

Gate	Count	% Dendritic cells
Dendritic cells	25.798	100,00%
Live	18.854	73,08%

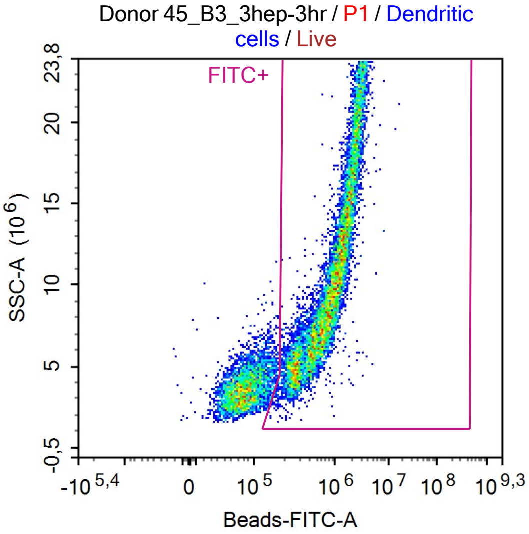

Gate	Count	% Live
Live	18.854	100,00%
FITC+	14.750	78,23%

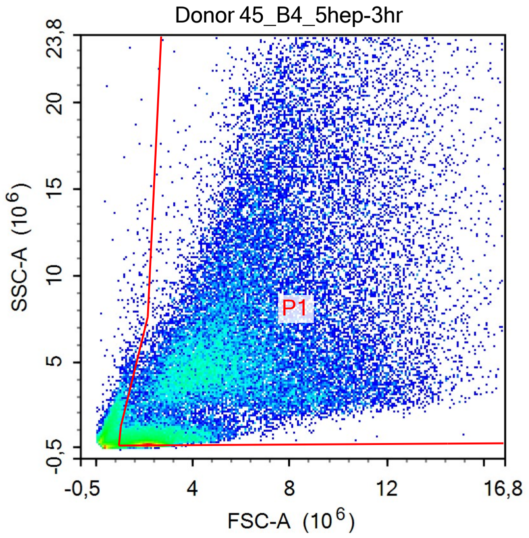

Gate	Count	% All
All	89.918	100,00%
P1	68.731	76,44%

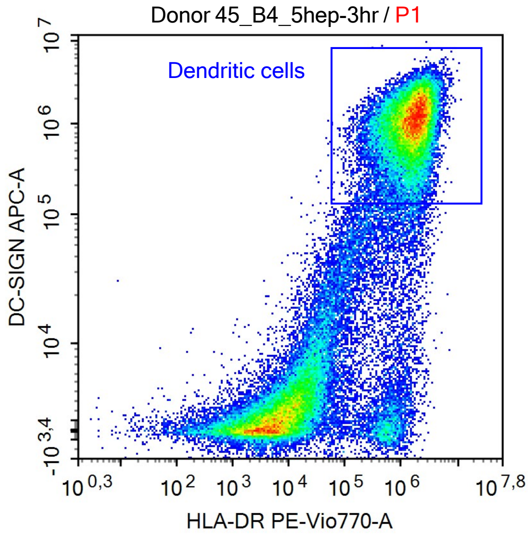

Gate	Count	% P1
P1	68.731	100,00%
Dendritic cells	29.267	42,58%

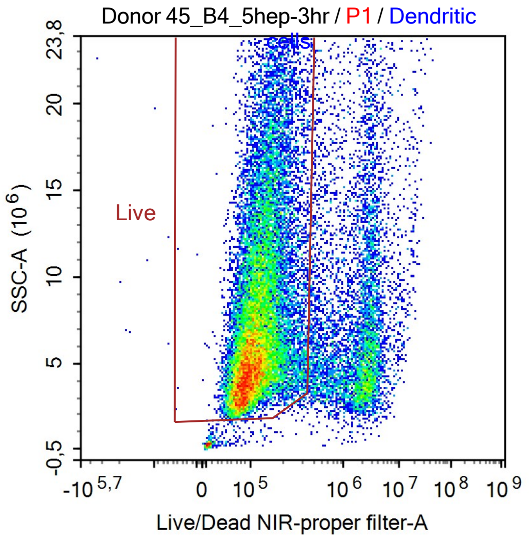

Gate	Count	% Dendritic cells
Dendritic cells	29.267	100,00%
Live	22.091	75,48%

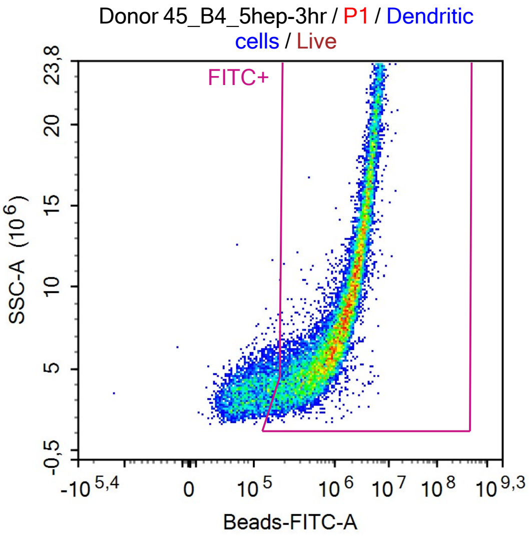

Gate	Count	% Live
Live	22.091	100,00%
FITC+	18.467	83,60%

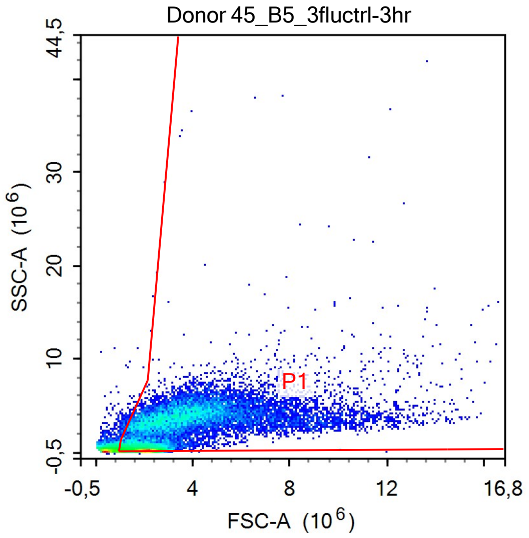

Gate	Count	% All
All	34.181	100,00%
P1	23.479	68,69%

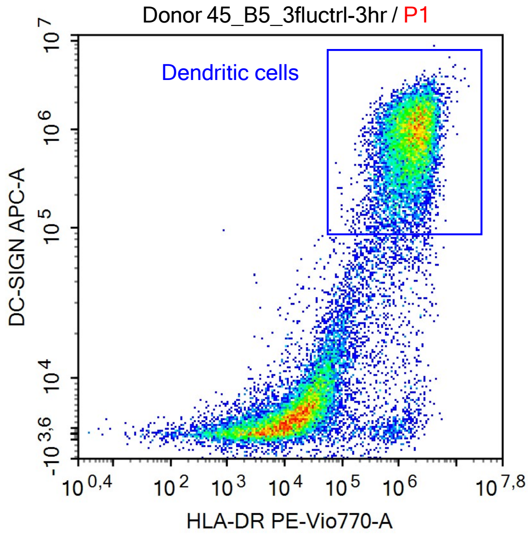

Gate	Count	% P1
P1	23.479	100,00%
Dendritic cells	9.465	40,31%

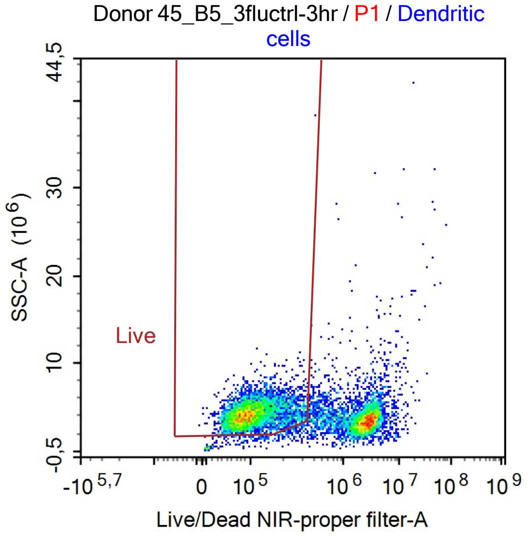

Gate	Count	% Dendritic cells
Dendritic cells	9.465	100,00%
Live	5.183	54,76%

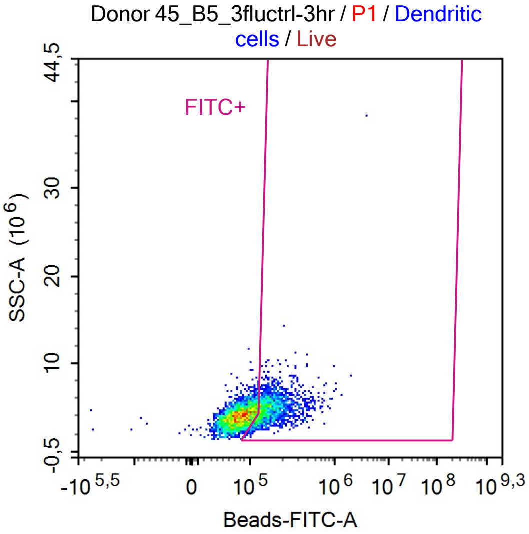

Gate	Count	% Live
Live	5.183	100,00%
FITC+	1.507	29,08%

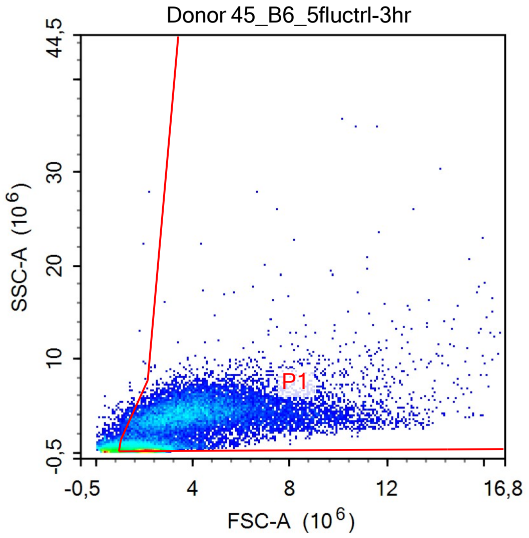

Gate	Count	% All
All	54.659	100,00%
P1	39.417	72,11%

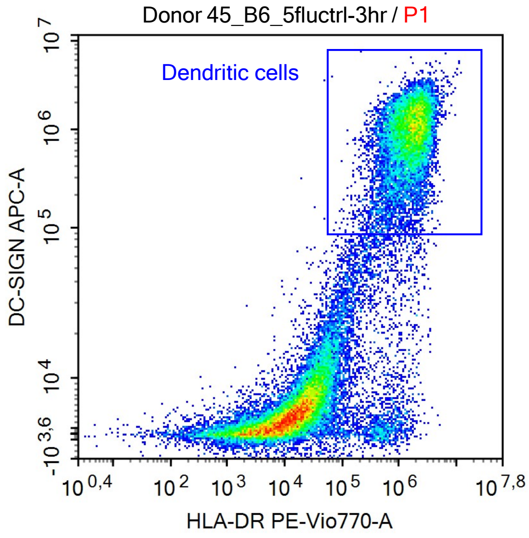

Gate	Count	% P1
P1	39.417	100,00%
Dendritic cells	11.879	30,14%

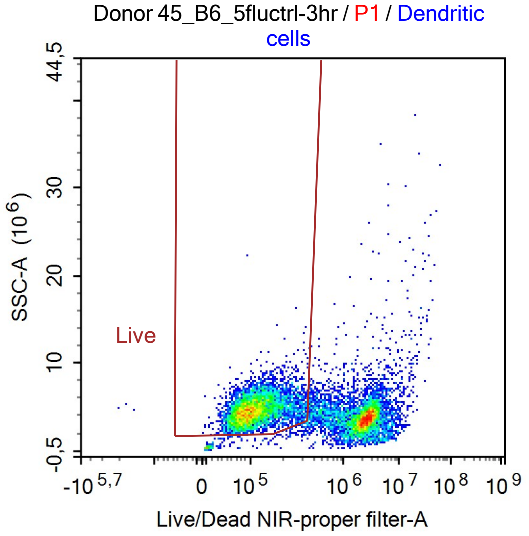

Gate	Count	% Dendritic cells
Dendritic cells	11.879	100,00%
Live	6.087	51,24%

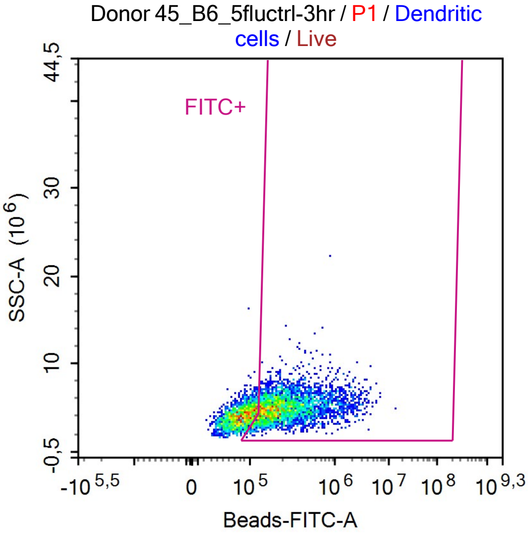

Gate	Count	% Live
Live	6.087	100,00%
FITC+	3.681	60,47%

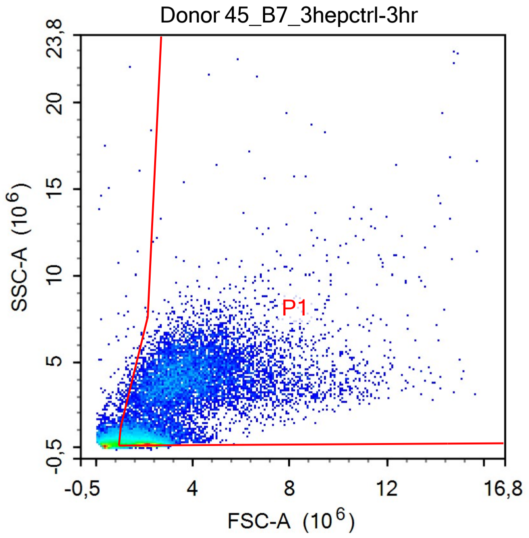

Gate	Count	% All
All	41.946	100,00%
P1	28.192	67,21%

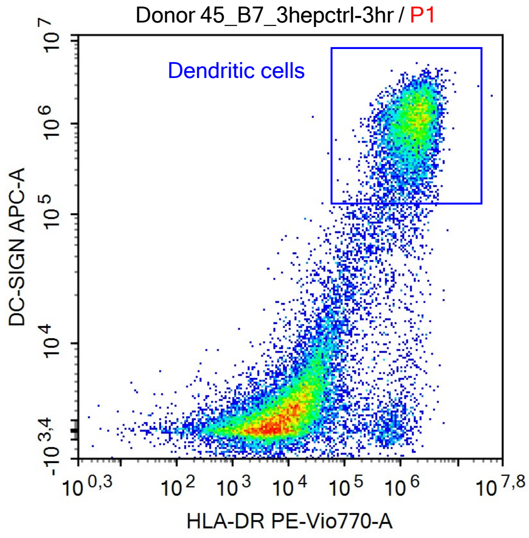

Gate	Count	% P1
P1	28.192	100,00%
Dendritic cells	6.570	23,30%

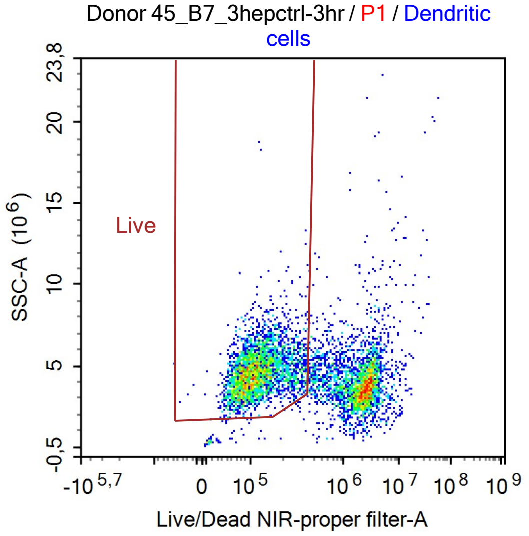

Gate	Count	% Dendritic cells
Dendritic cells	6.570	100,00%
Live	3.481	52,98%

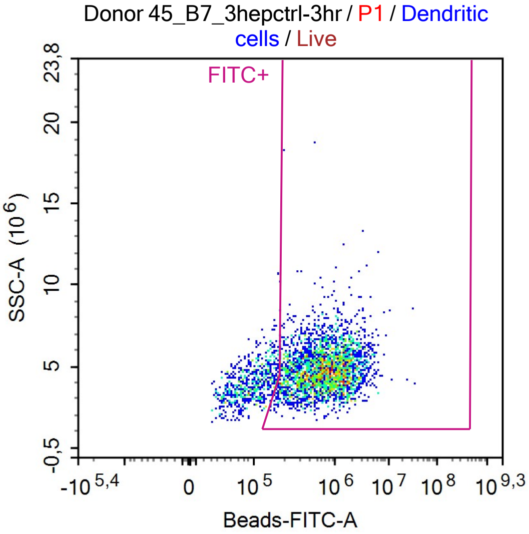

Gate	Count	% Live
Live	3.481	100,00%
FITC+	2.762	79,35%

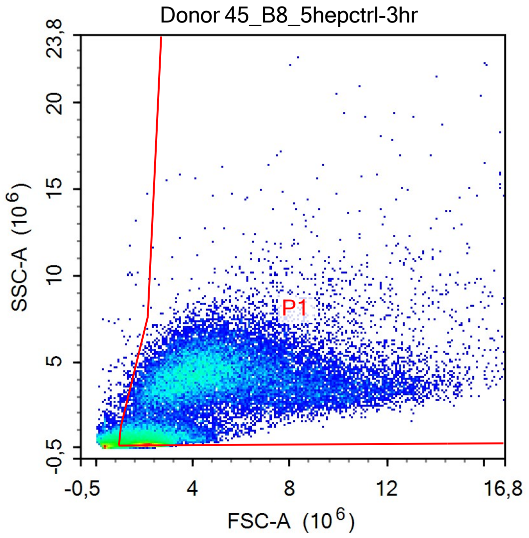

Gate	Count	% All
All	71.063	100,00%
P1	54.349	76,48%

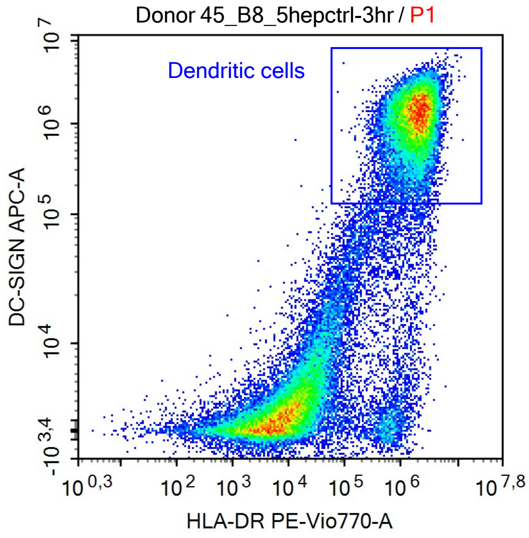

Gate	Count	% P1
P1	54.349	100,00%
Dendritic cells	19.920	36,65%

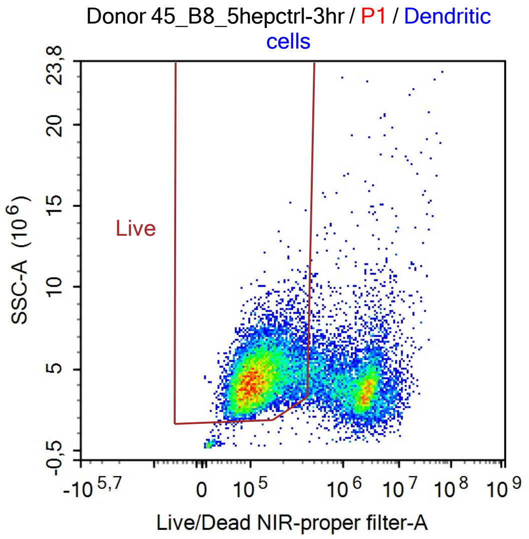

Gate	Count	% Dendritic cells
Dendritic cells	19.920	100,00%
Live	12.569	63,10%

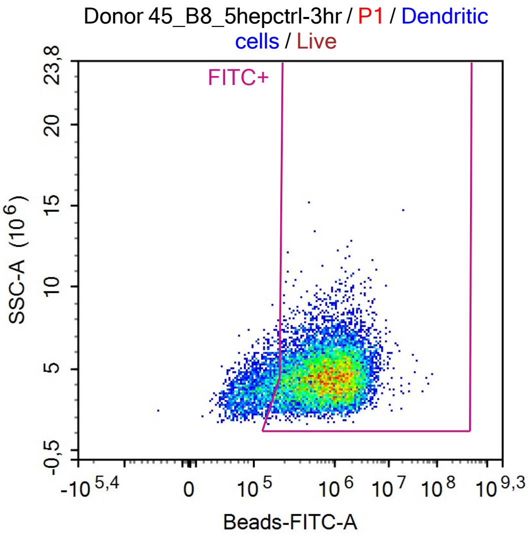

Gate	Count	% Live
Live	12.569	100,00%
FITC+	10.696	85,10%

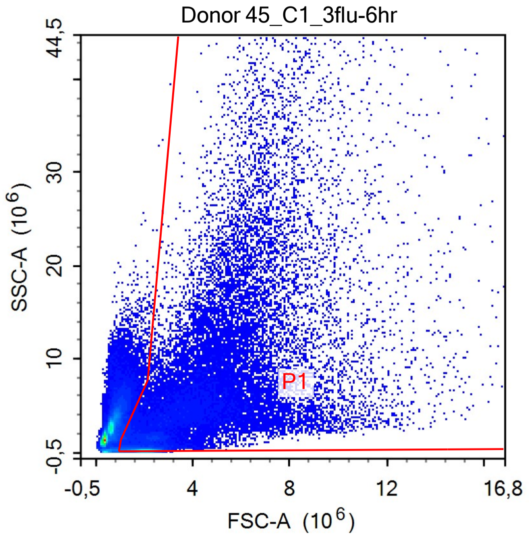

Gate	Count	% All
All	461.055	100,00%
P1	58.808	12,76%

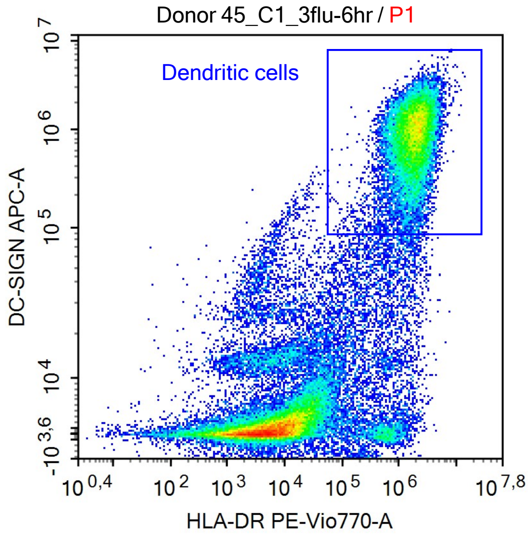

Gate	Count	% P1
P1	58.808	100,00%
Dendritic cells	18.077	30,74%

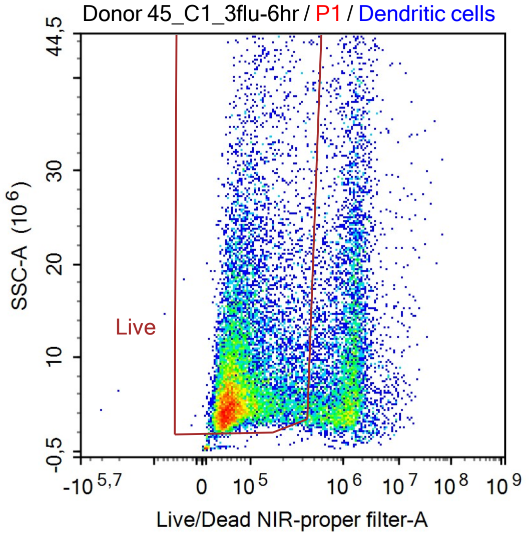

Gate	Count	% Dendritic cells
Dendritic cells	18.077	100,00%
Live	12.152	67,22%

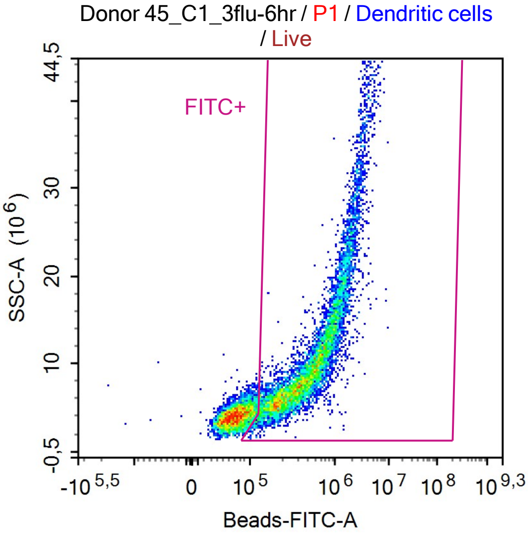

Gate	Count	% Live
Live	12.152	100,00%
FITC+	8.371	68,89%

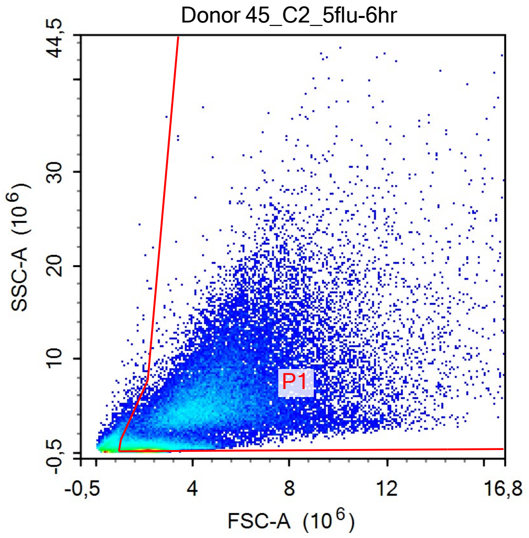

Gate	Count	% All
All	119.545	100,00%
P1	89.176	74,60%

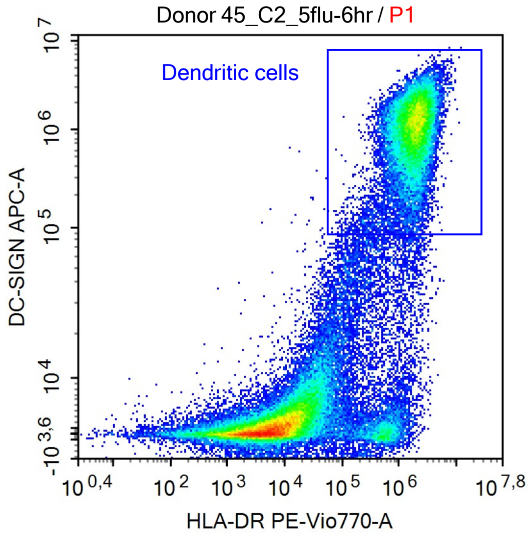

Gate	Count	% P1
P1	89.176	100,00%
Dendritic cells	25.987	29,14%

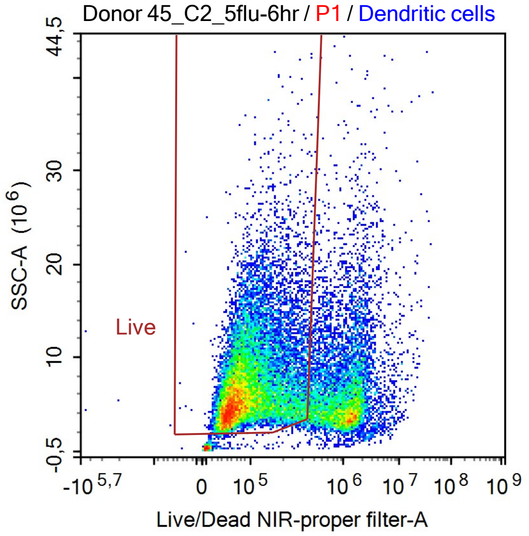

Gate	Count	% Dendritic cells
Dendritic cells	25.987	100,00%
Live	17.672	68,00%

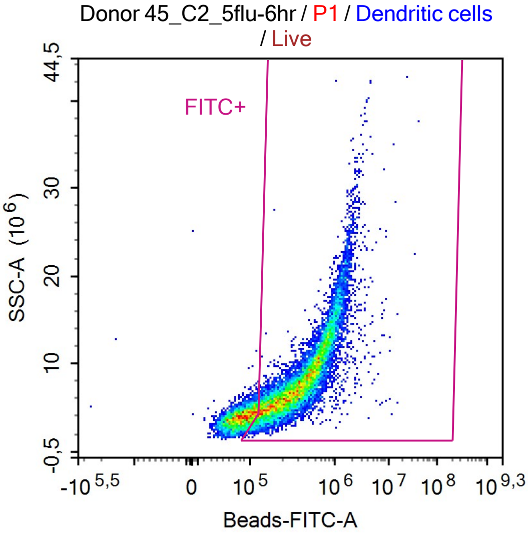

Gate	Count	% Live
Live	17.672	100,00%
FITC+	13.170	74,52%

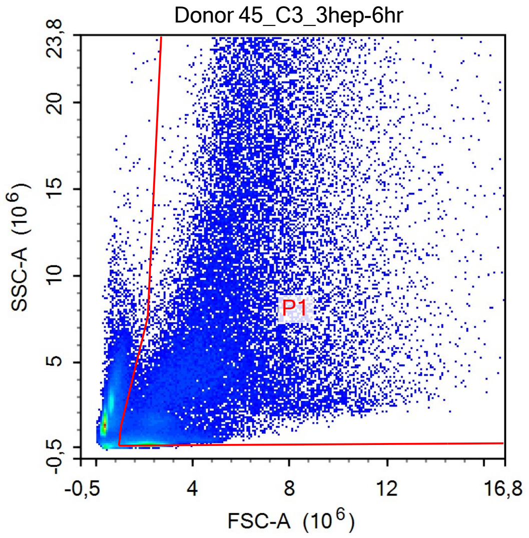

Gate	Count	% All
All	343.838	100,00%
P1	100.836	29,33%

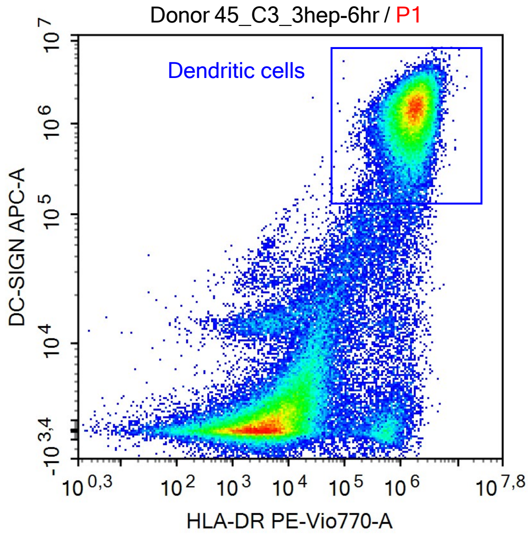

Gate	Count	% P1
P1	100.836	100,00%
Dendritic cells	31.867	31,60%

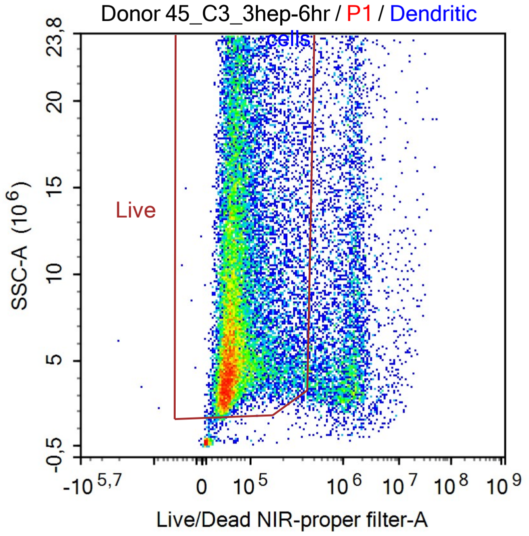

Gate	Count	% Dendritic cells
Dendritic cells	31.867	100,00%
Live	25.633	80,44%

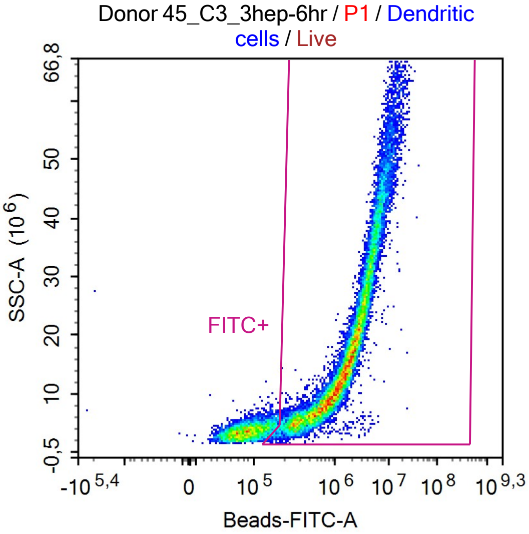

Gate	Count	% Live
Live	25.633	100,00%
FITC+	20.873	81,43%

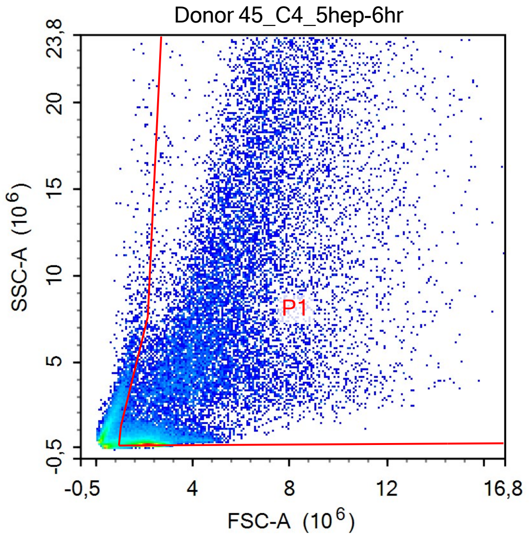

Gate	Count	% All
All	91.153	100,00%
P1	61.937	67,95%

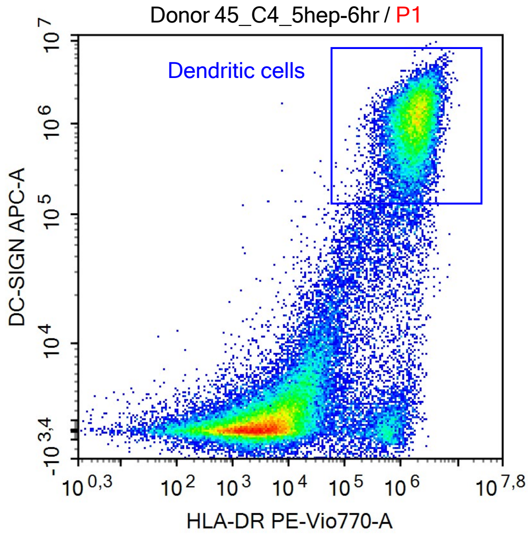

Gate	Count	% P1
P1	61.937	100,00%
Dendritic cells	14.810	23,91%

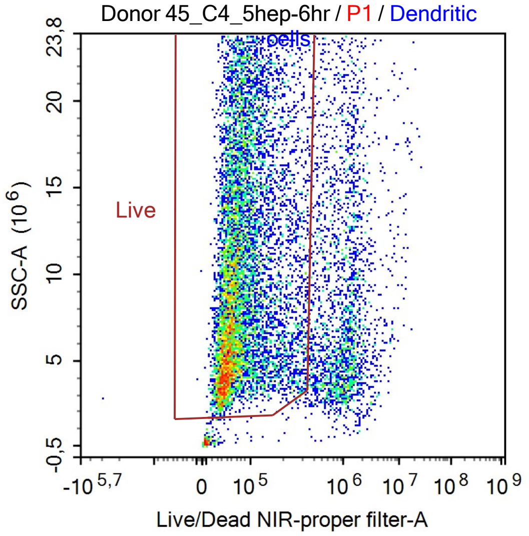

Gate	Count	% Dendritic cells
Dendritic cells	14.810	100,00%
Live	11.807	79,72%

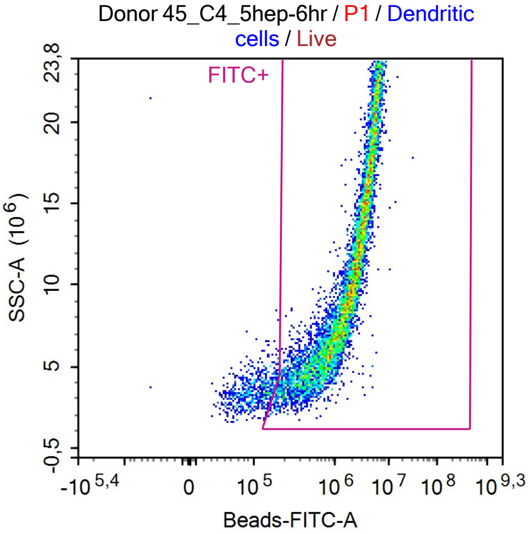

Gate	Count	% Live
Live	11.807	100,00%
FITC+	11.068	93,74%

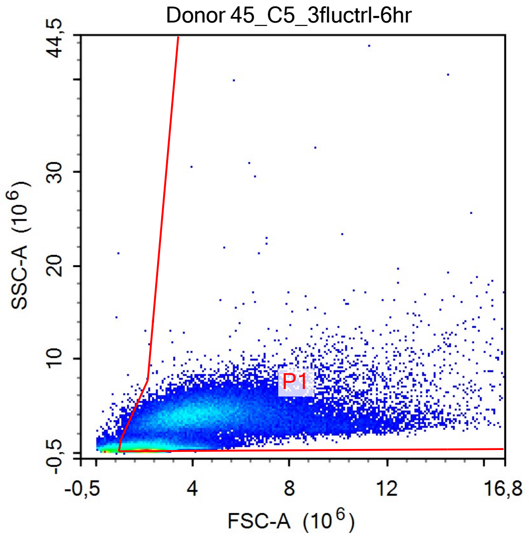

Gate	Count	% All
All	125.277	100,00%
P1	98.794	78,86%

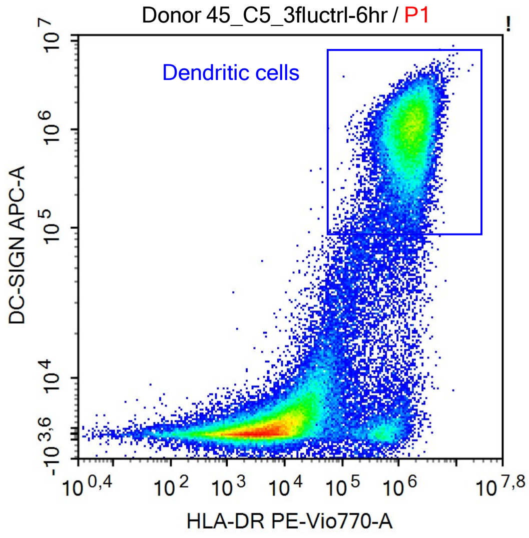

Gate	Count	% P1
P1	98.794	100,00%
Dendritic cells	27.648	27,99%

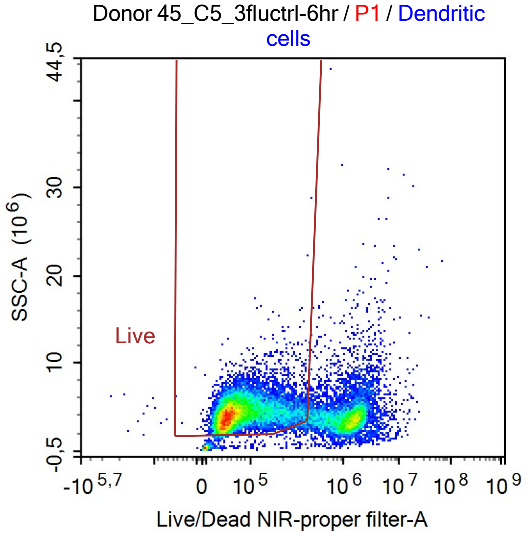

Gate	Count	% Dendritic cells
Dendritic cells	27.648	100,00%
Live	18.815	68,05%

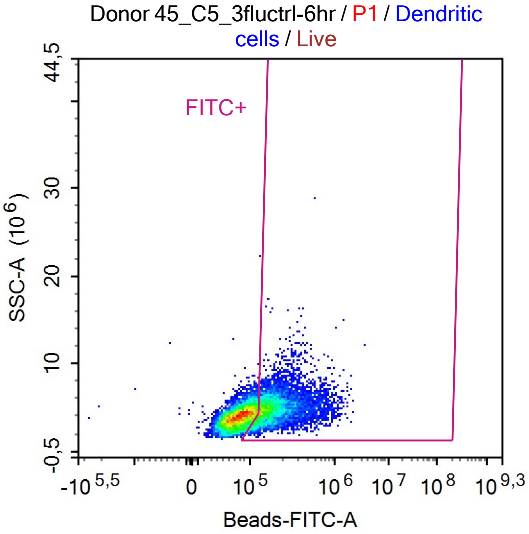

Gate	Count	% Live
Live	18.815	100,00%
FITC+	6.847	36,39%

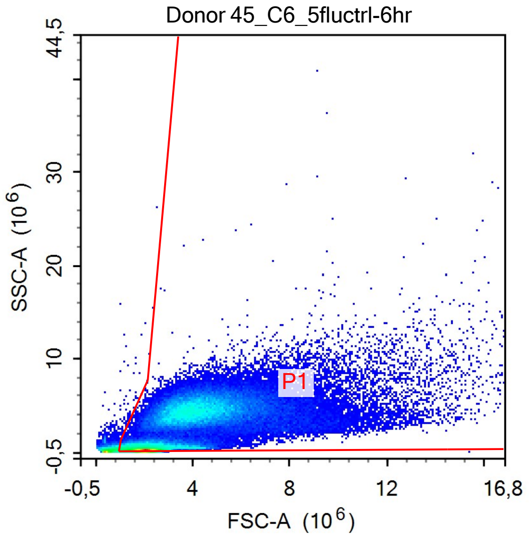

Gate	Count	% All
All	183.402	100,00%
P1	155.454	84,76%

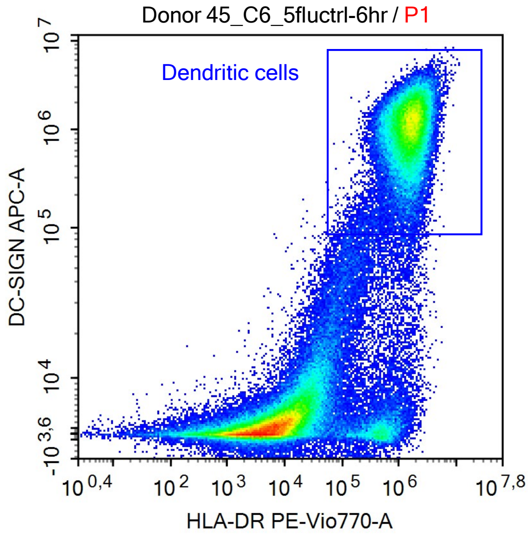

Gate	Count	% P1
P1	155.454	100,00%
Dendritic cells	48.181	30,99%

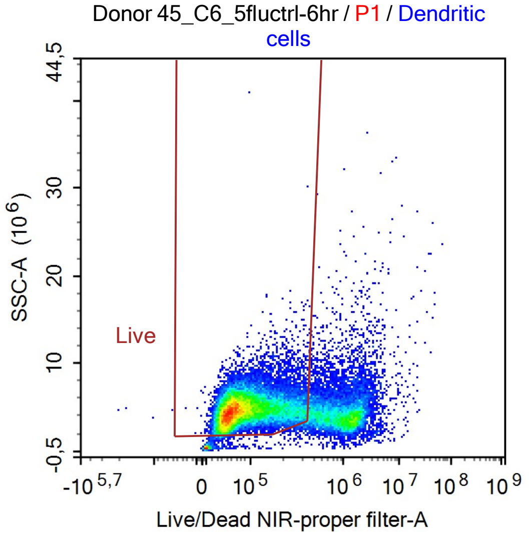

Gate	Count	% Dendritic cells
Dendritic cells	48.181	100,00%
Live	35.744	74,19%

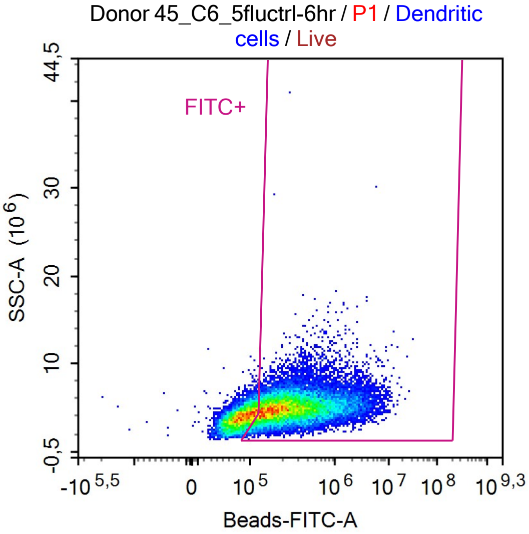

Gate	Count	% Live
Live	35.744	100,00%
FITC+	24.773	69,31%

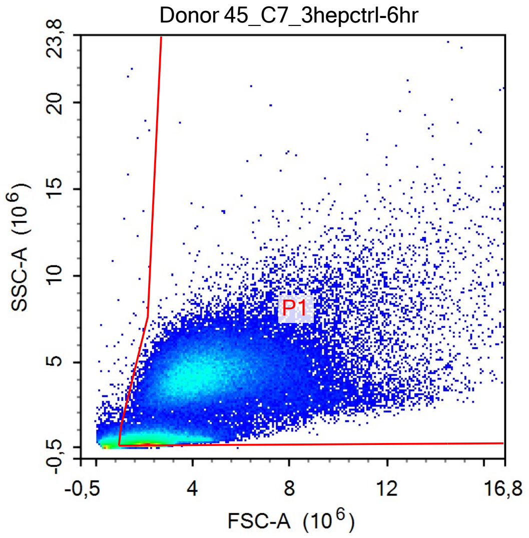

Gate	Count	% All
All	201.753	100,00%
P1	165.124	81,84%

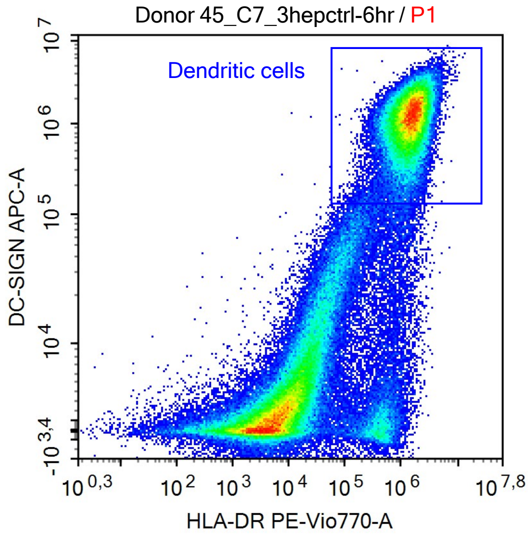

Gate	Count	% P1
P1	165.124	100,00%
Dendritic cells	51.255	31,04%

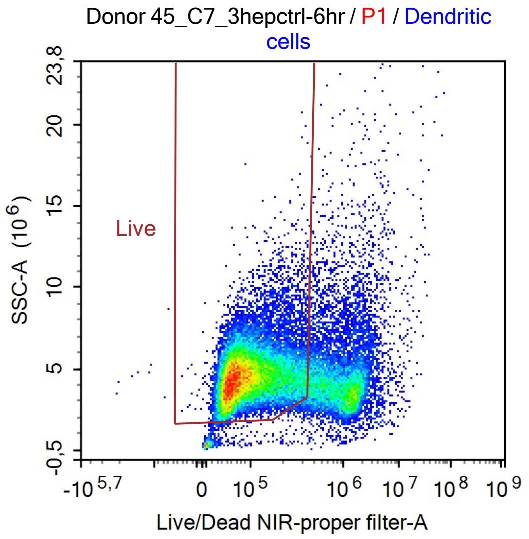

Gate	Count	% Dendritic cells
Dendritic cells	51.255	100,00%
Live	37.711	73,58%

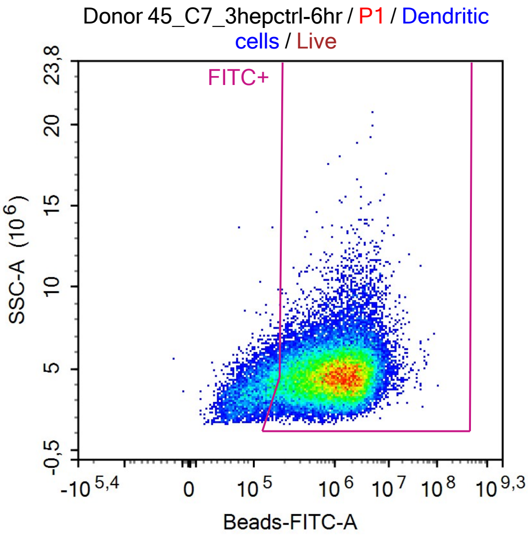

Gate	Count	% Live
Live	37.711	100,00%
FITC+	33.023	87,57%

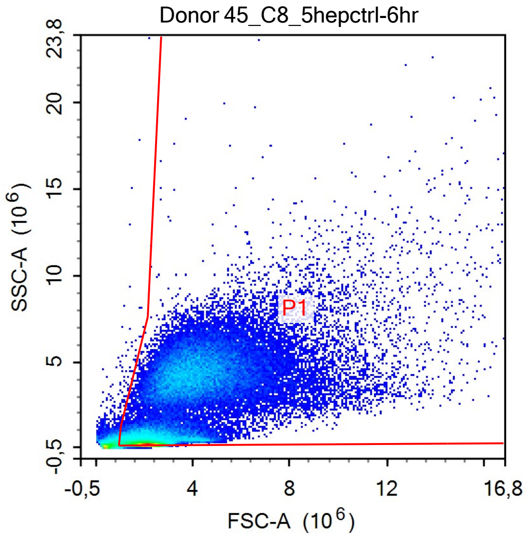

Gate	Count	% All
All	142.112	100,00%
P1	114.231	80,38%

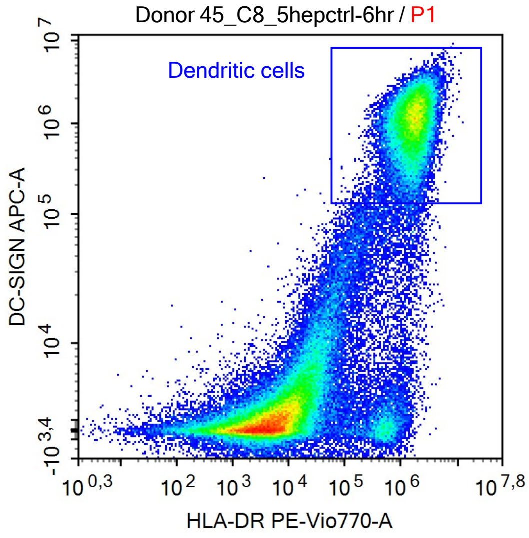

Gate	Count	% P1
P1	114.231	100,00%
Dendritic cells	26.579	23,27%

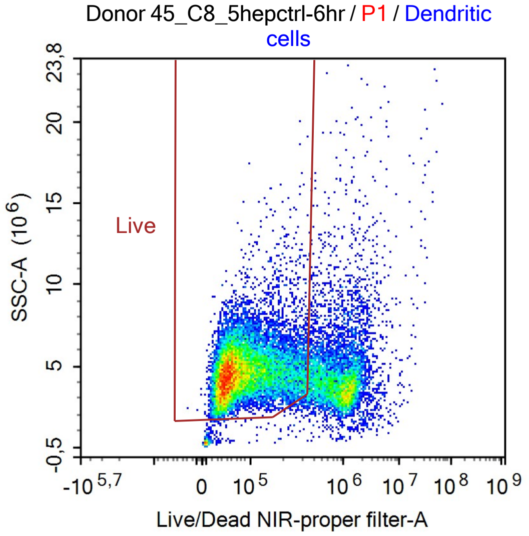

Gate	Count	% Dendritic cells
Dendritic cells	26.579	100,00%
Live	18.985	71,43%

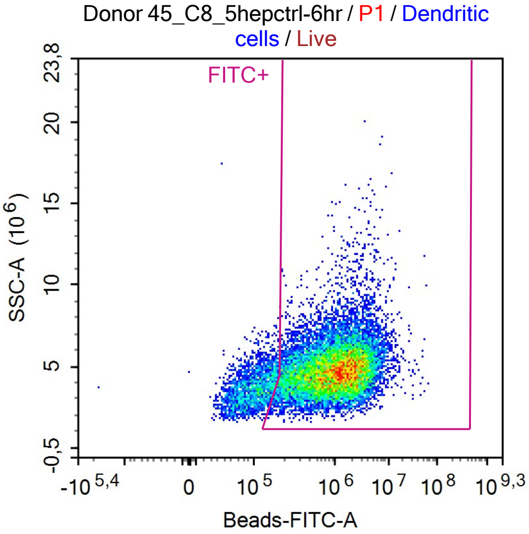

Gate	Count	% Live
Live	18.985	100,00%
FITC+	16.368	86,22%

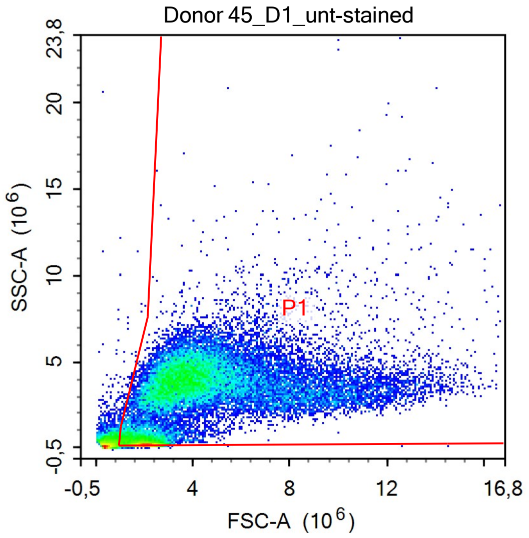

Gate	Count	% All
All	42.856	100,00%
P1	32.674	76,24%

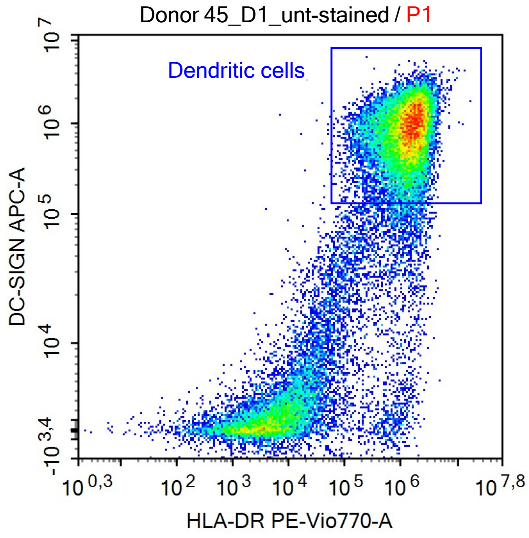

Gate	Count	% P1
P1	32.674	100,00%
Dendritic cells	18.440	56,44%

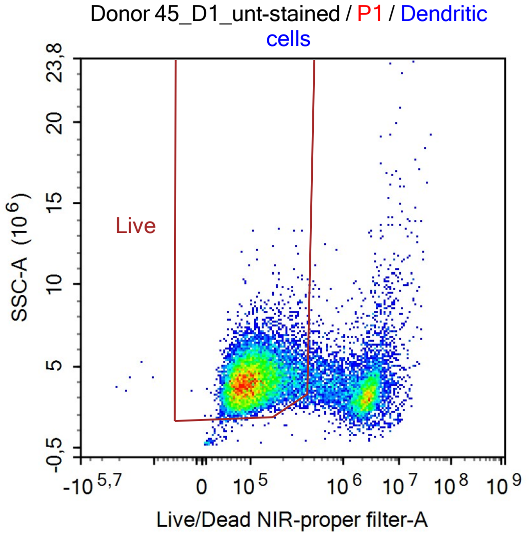

Gate	Count	% Dendritic cells
Dendritic cells	18.440	100,00%
Live	13.060	70,82%

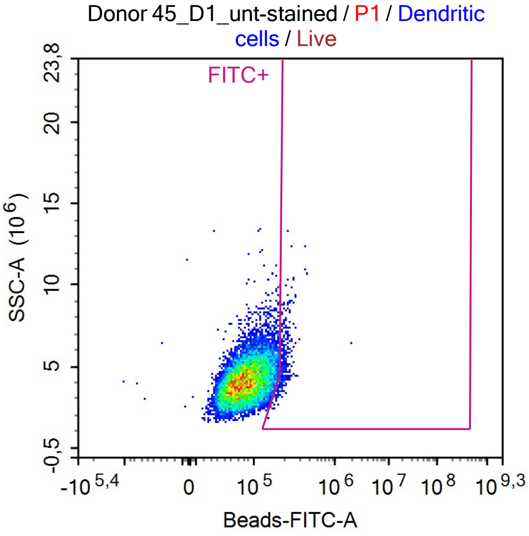

Gate	Count	% Live
Live	13.060	100,00%
FITC+	248	1,90%

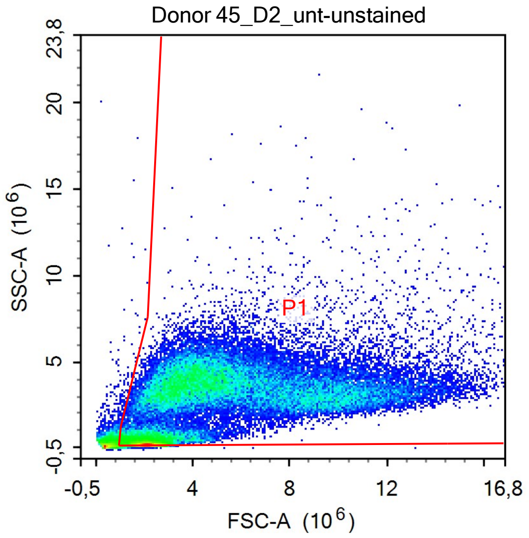

Gate	Count	% All
All	81.005	100,00%
P1	65.904	81,36%

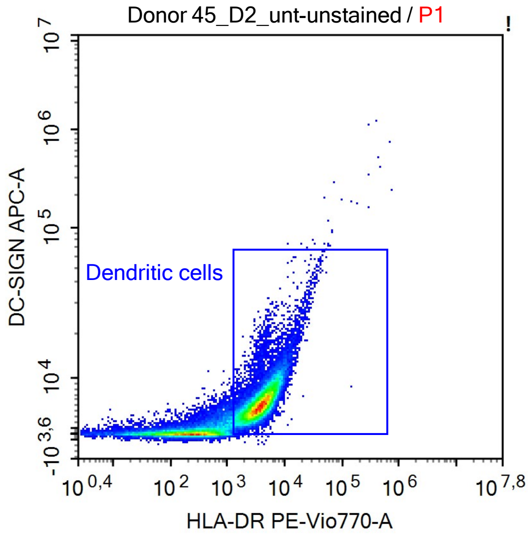

Gate	Count	% P1
P1	65.904	100,00%
Dendritic cells	32.896	49,92%

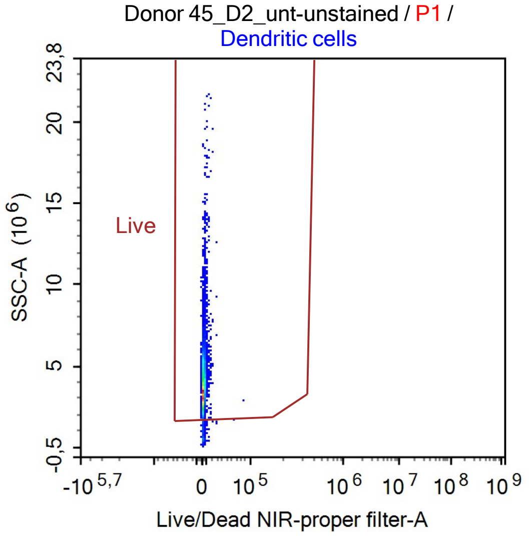

Gate	Count	% Dendritic cells
Dendritic cells	32.896	100,00%
Live	31.771	96,58%

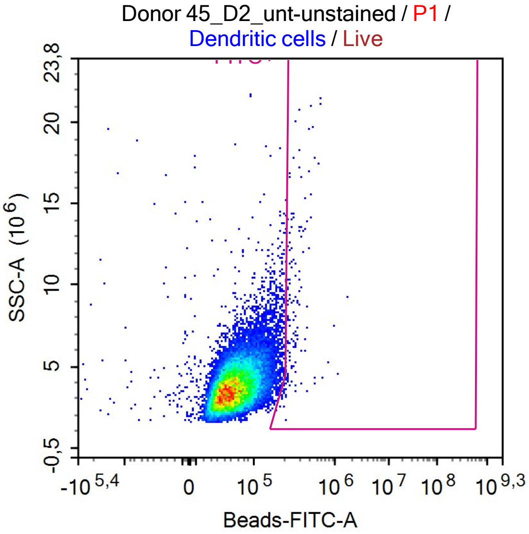

Gate	Count	% Live
Live	31.771	100,00%
FITC+	126	0,40%

Donor 45 FLU

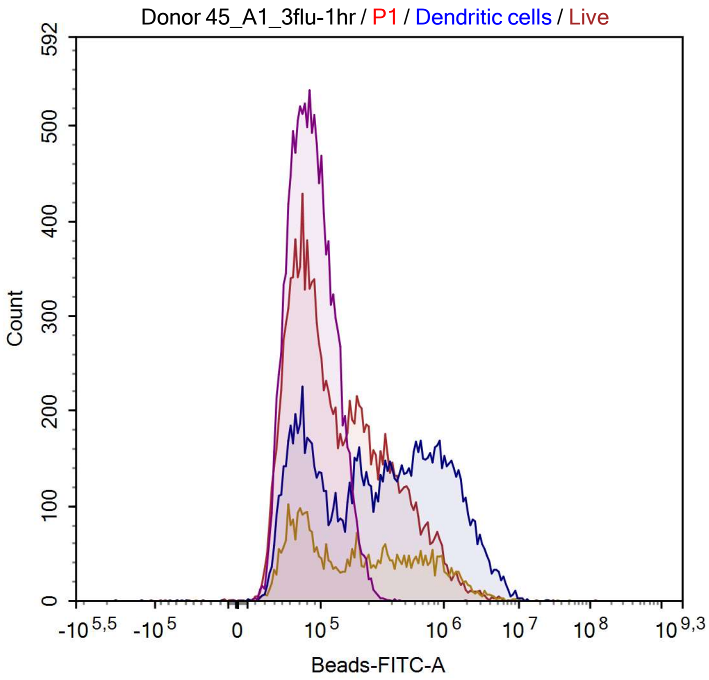

#	Sample	Gate	Count	Mean X	Median X
1	Donor 45_A1_3flu-1hr	Live	14.332	227.982	110.745
2	Donor 45_B1_3flu-3hr	Live*	4.099	437.355	165.173
3	Donor 45_C1_3flu-6hr	Live*	12.152	647.736	250.656
4	Donor 45_D1_unt-[illegible]	Live*	13.060	89.496	84.376

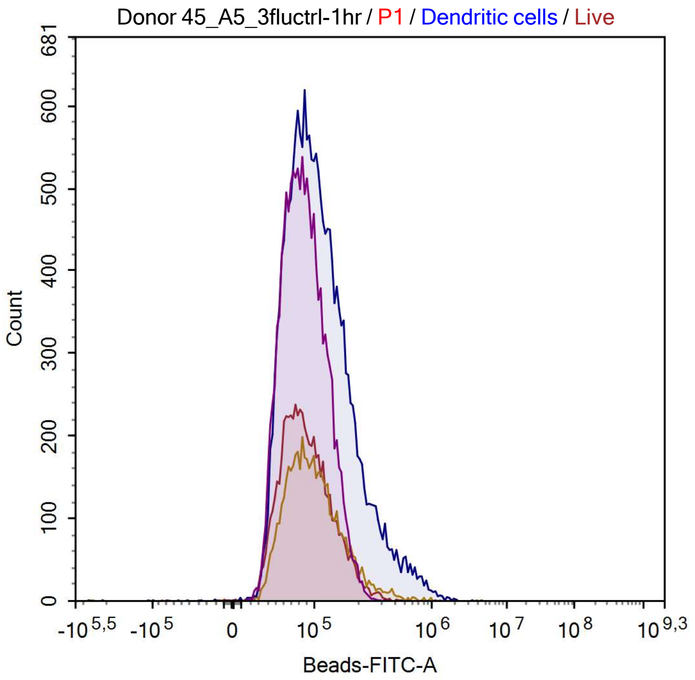

#	Sample	Gate	Count	Mean X	Median X
1	Donor 45_A5_3fluctrl-1hr	Live	6.119	94.978	85.092
2	Donor 45_B5_3fluctrl-3hr	Live*	5.183	111.817	95.205
3	Donor 45_C5_3fluctrl-6hr	Live*	18.815	132.241	101.177
4	Donor 45_D1_unt-[illegible]	Live*	13.060	89.496	84.376

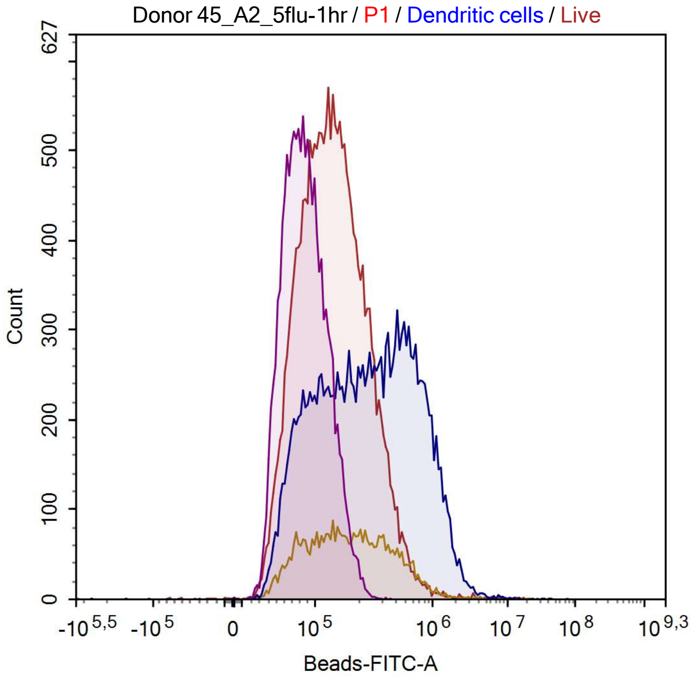

#	Sample	Gate	Count	Mean X	Median X
1	Donor 45_A2_5flu-1hr	Live	20.481	180.201	129.106
2	Donor 45_B2_5flu-3hr	Live*	3.915	281.150	163.458
3	Donor 45_C2_5flu-6hr	Live*	17.672	414.182	230.279
4	Donor 45_D1_unt-[illegible]	Live*	13.060	89.496	84.376

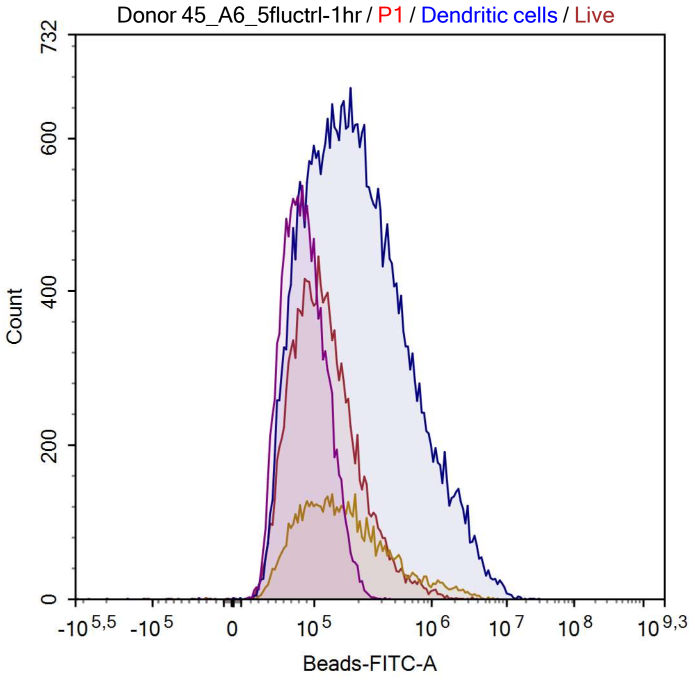

#	Sample	Gate	Count	Mean X	Median X
1	Donor 45_A6_5fluctrl-1hr	Live	14.129	143.638	111.042
2	Donor 45_B6_5fluctrl-3hr	Live*	6.087	278.058	143.781
3	Donor 45_C6_5fluctrl-6hr	Live*	35.744	428.219	176.819
4	Donor 45_D1_unt-[illegible]	Live*	13.060	89.496	84.376

Donor 45 HEP

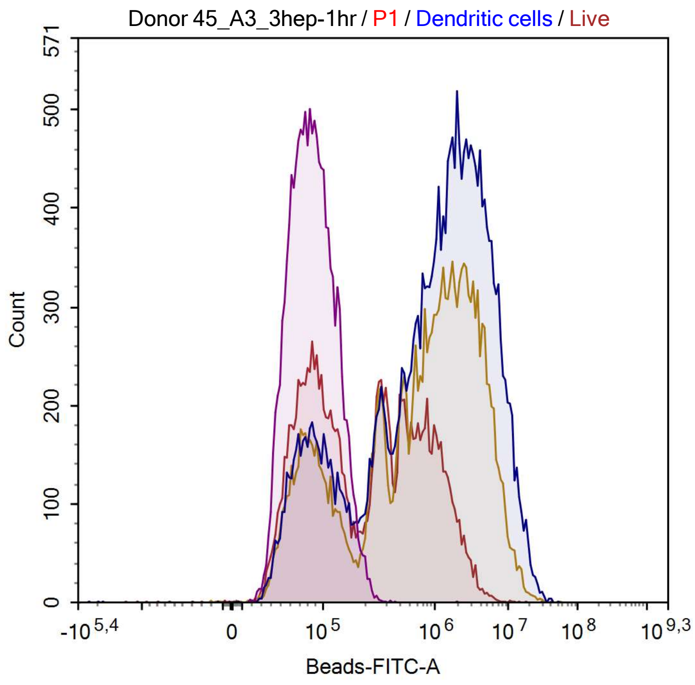

#	Sample	Gate	Count	Mean X	Median X
1	Donor 45_A3_3hep-1hr	Live	13.541	469.726	215.377
2	Donor 45_B3_3hep-3hr	Live*	18.854	1.790.932	838.967
3	Donor 45_C3_3hep-6hr	Live*	25.633	2.508.999	1.172.151
4	Donor 45_D1_unt-[illegible]	Live*	13.060	89.496	84.376

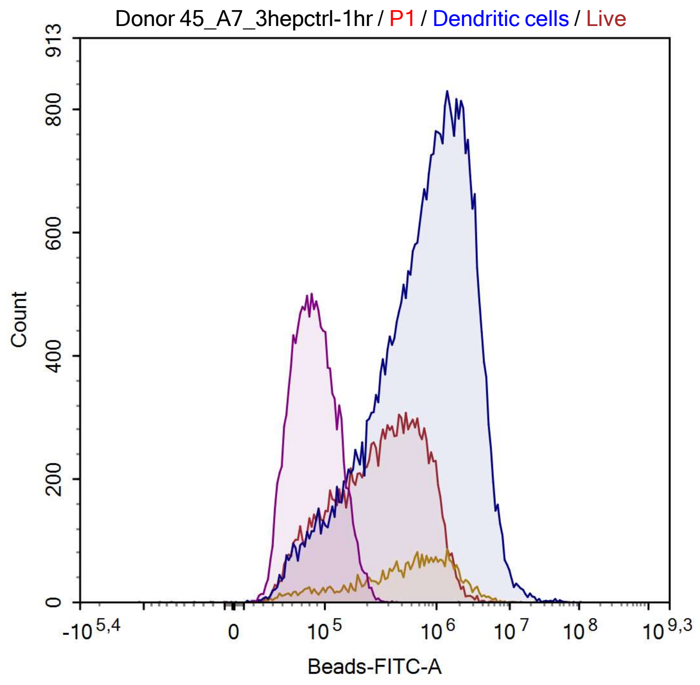

#	Sample	Gate	Count	Mean X	Median X
1	Donor 45_A7_3hepctrl-1hr	Live	14.856	409.829	270.835
2	Donor 45_B7_3hepctrl-3hr	Live*	3.481	849.589	503.419
3	Donor 45_C7_3hepctrl-6hr	Live*	37.711	1.454.734	835.261
4	Donor 45_D1_unt-[illegible]	Live*	13.060	89.496	84.376

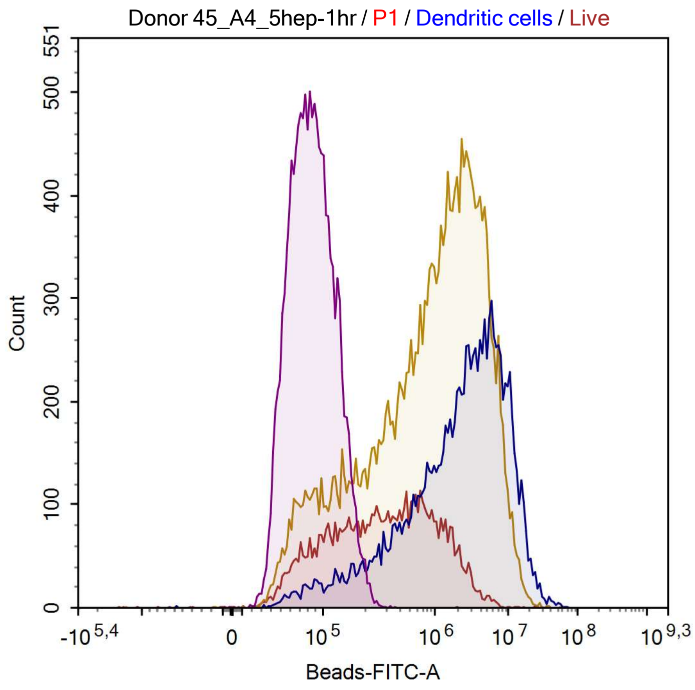

#	Sample	Gate	Count	Mean X	Median X
1	Donor 45_A4_5hep-1hr	Live	6.356	626.773	288.020
2	Donor 45_B4_5hep-3hr	Live*	22.091	2.040.434	1.059.001
3	Donor 45_C4_5hep-6hr	Live*	11.807	4.327.917	2.545.932
4	Donor 45_D1_unt-[illegible]	Live*	13.060	89.496	84.376

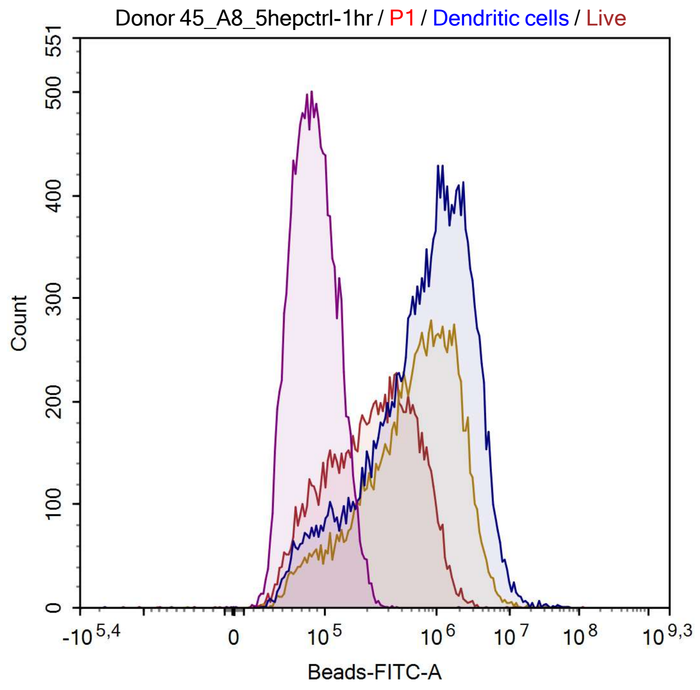

#	Sample	Gate	Count	Mean X	Median X
1	Donor 45_A8_5hepctrl-1hr	Live	10.744	368.840	228.412
2	Donor 45_B8_5hepctrl-3hr	Live*	12.569	1.008.711	601.683
3	Donor 45_C8_5hepctrl-6hr	Live*	18.985	1.458.732	829.042
4	Donor 45_D1_unt-[illegible]	Live*	13.060	89.496	84.376
