# Supplementary material for: Assessing the Immunomodulatory Effect of Size on the Uptake and Immunogenicity of Influenza- and Hepatitis B Subunit Vaccines In Vitro
Source: Pharmaceuticals (Basel). 2022 Jul 18;15(7):887. doi: 10.3390/ph15070887 (PMC9321264; doi:10.3390/ph15070887)
Supplement: Supplementary file 1 [file pharmaceuticals-15-00887-s001.zip › Supplementary file S4.pdf]

Report of Donor 48

Specimen Name: Donor 48 | Run Time: 11-12-2021 12:22

Cytometer: NovoCyte Quanteon 621200611403 | Software: NovoExpress 1.5.6

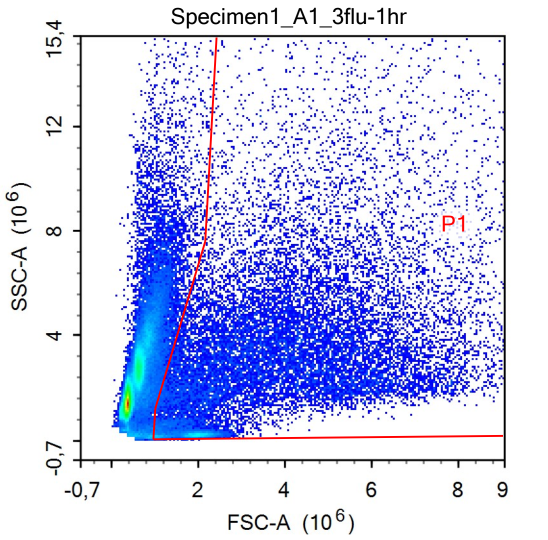

Gate	Count	% All
All	208.957	100,00%
P1	23.718	11,35%

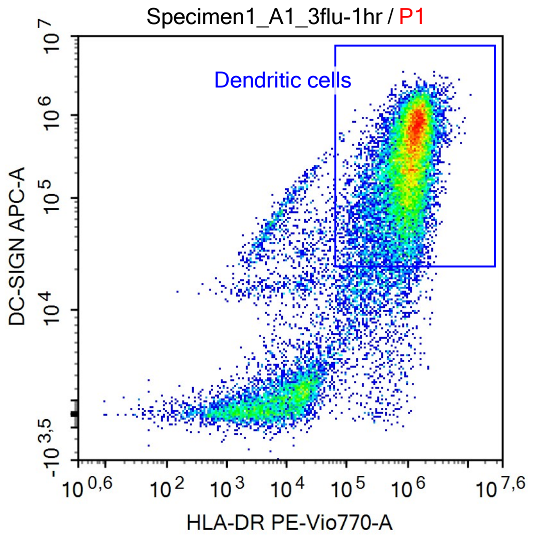

Gate	Count	% P1
P1	23.718	100,00%
Dendritic cells	16.396	69,13%

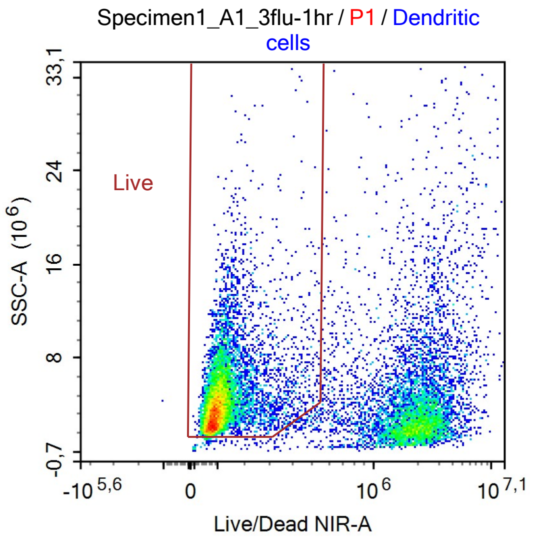

Gate	Count	% Dendritic cells
Dendritic cells	16.396	100,00%
Live	10.059	61,35%

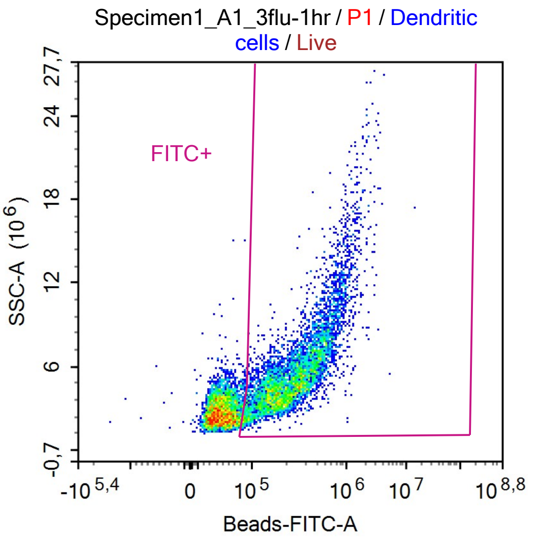

Gate	Count	% Live
Live	10.059	100,00%
FITC+	6.101	60,65%

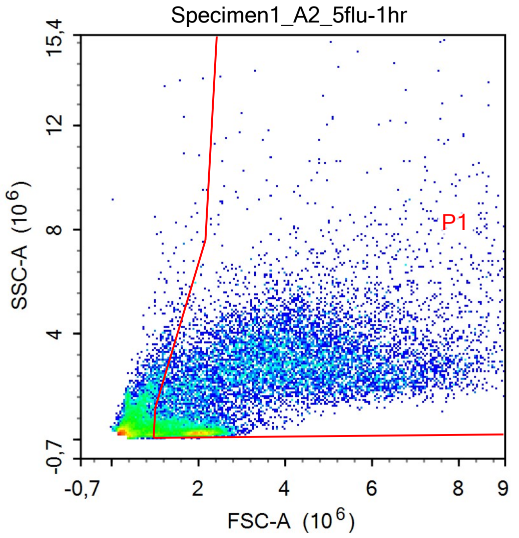

Gate	Count	% All
All	27.577	100,00%
P1	15.778	57,21%

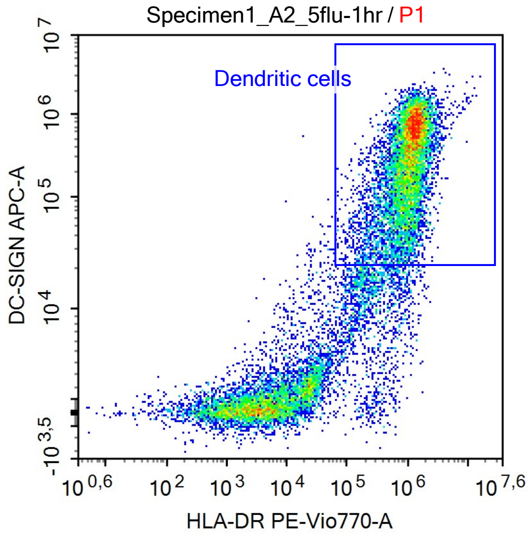

Gate	Count	% P1
P1	15.778	100,00%
Dendritic cells	8.420	53,37%

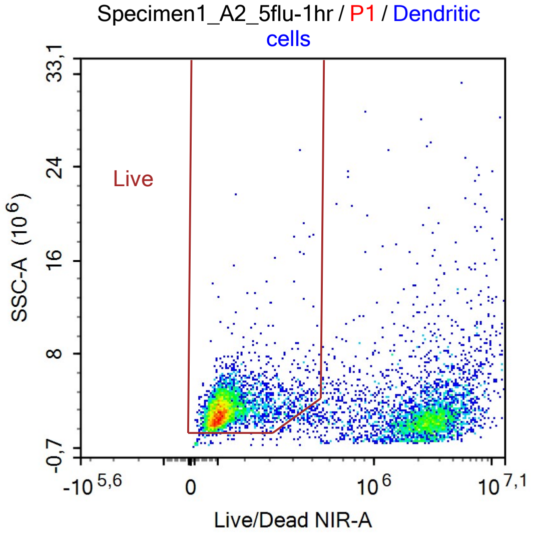

Gate	Count	% Dendritic cells
Dendritic cells	8.420	100,00%
Live	4.809	57,11%

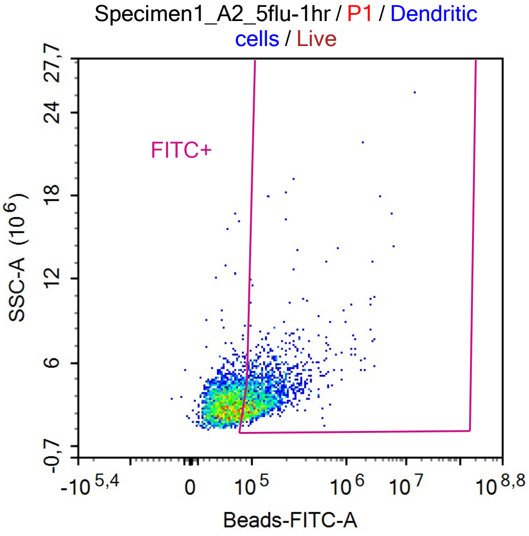

Gate	Count	% Live
Live	4.809	100,00%
FITC+	1.846	38,39%

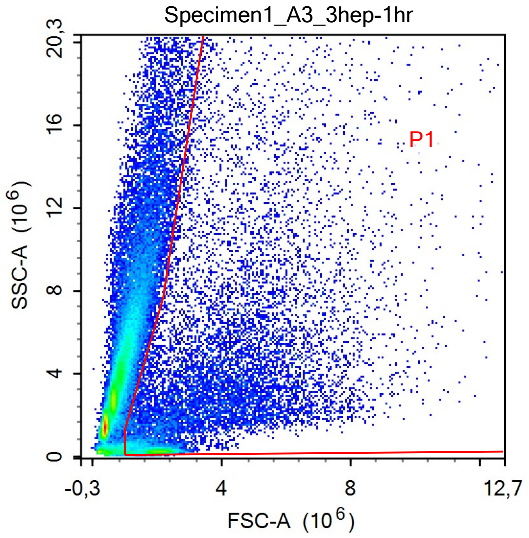

Gate	Count	% All
All	104.934	100,00%
P1	18.811	17,93%

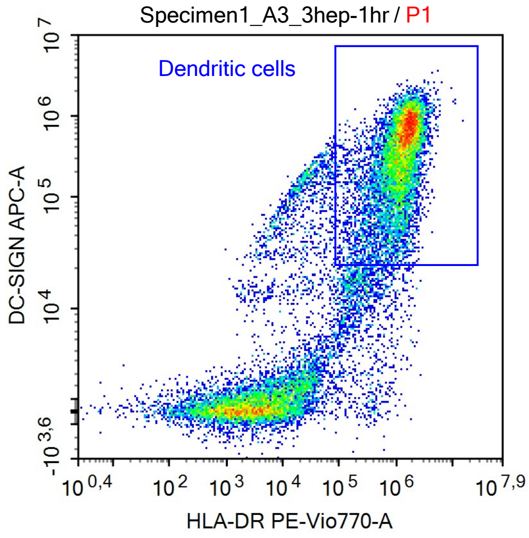

Gate	Count	% P1
P1	18.811	100,00%
Dendritic cells	9.035	48,03%

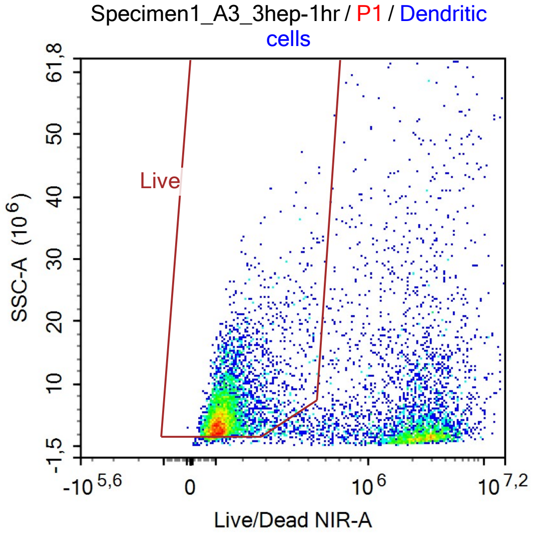

Gate	Count	% Dendritic cells
Dendritic cells	9.035	100,00%
Live	5.294	58,59%

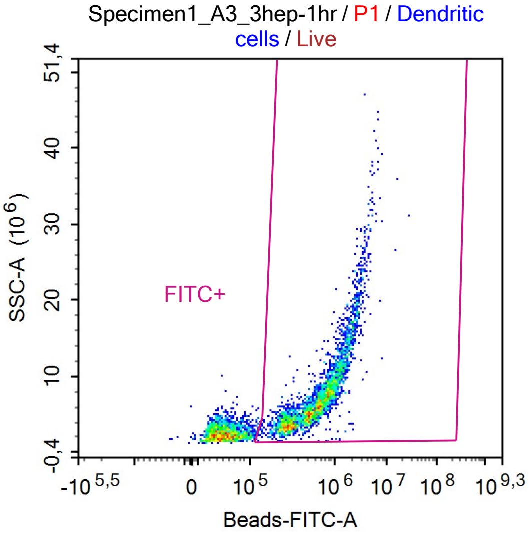

Gate	Count	% Live
Live	5.294	100,00%
FITC+	3.537	66,81%

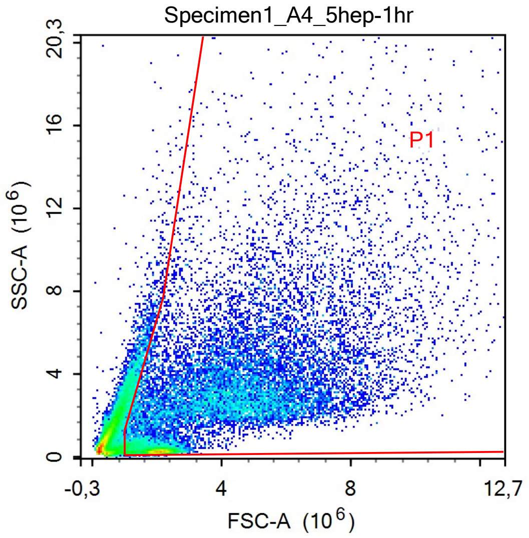

Gate	Count	% All
All	42.683	100,00%
P1	20.348	47,67%

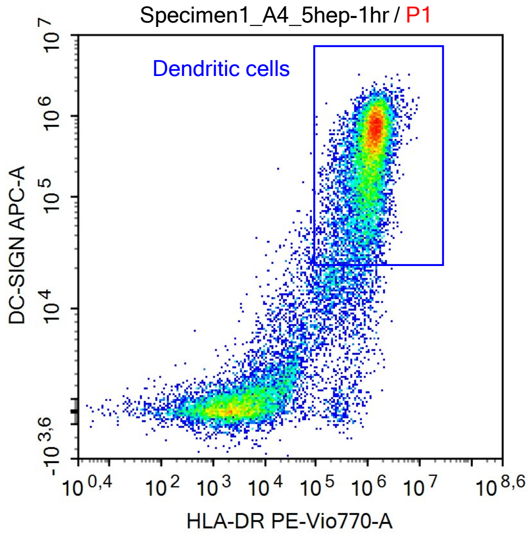

Gate	Count	% P1
P1	20.348	100,00%
Dendritic cells	11.331	55,69%

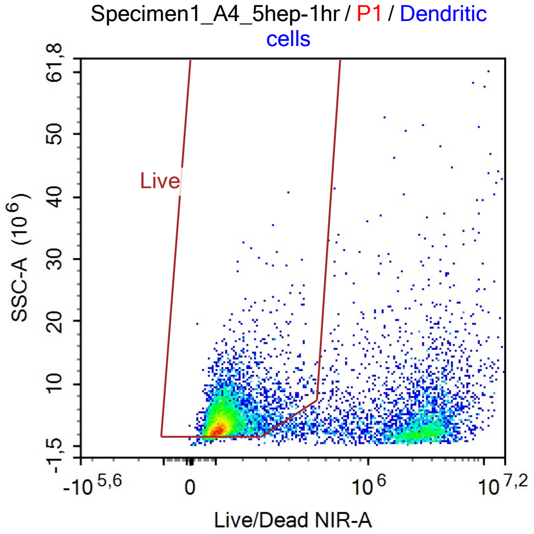

Gate	Count	% Dendritic cells
Dendritic cells	11.331	100,00%
Live	7.802	68,86%

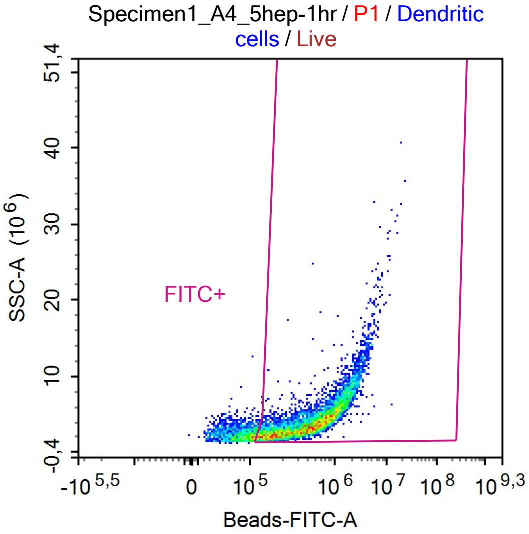

Gate	Count	% Live
Live	7.802	100,00%
FITC+	6.211	79,61%

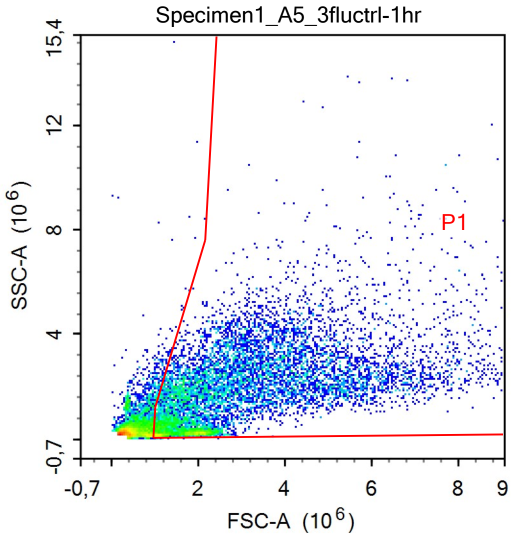

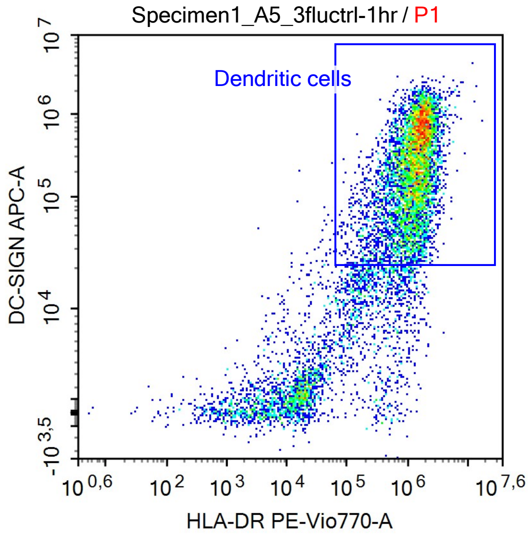

Gate	Count	% All
All	16.558	100,00%
P1	9.244	55,83%

Gate	Count	% P1
P1	9.244	100,00%
Dendritic cells	6.242	67,52%

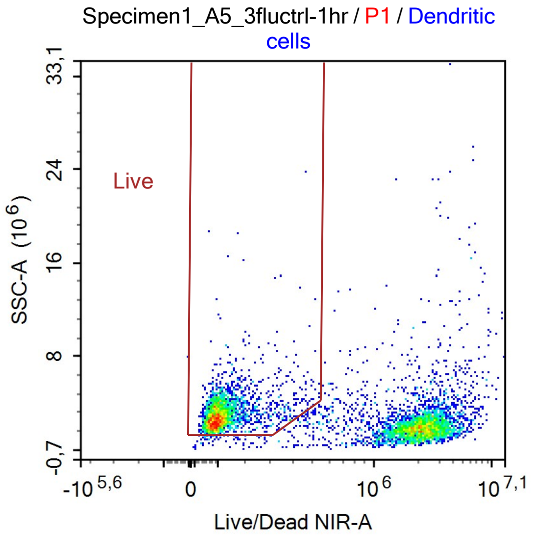

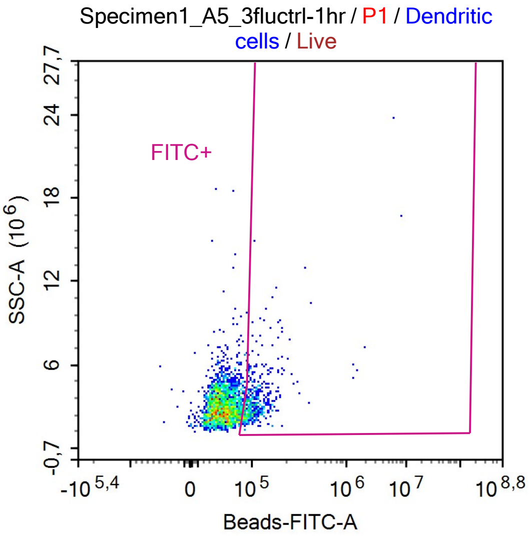

Gate	Count	% Dendritic cells
Dendritic cells	6.242	100,00%
Live	2.855	45,74%

Gate	Count	% Live
Live	2.855	100,00%
FITC+	474	16,60%

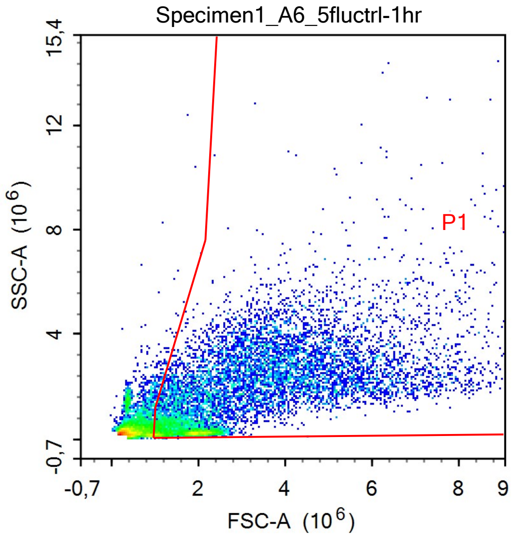

Gate	Count	% All
All	19.099	100,00%
P1	10.374	54,32%

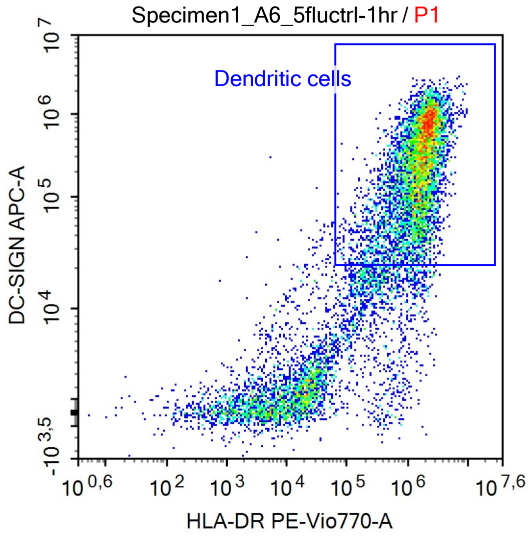

Gate	Count	% P1
P1	10.374	100,00%
Dendritic cells	6.325	60,97%

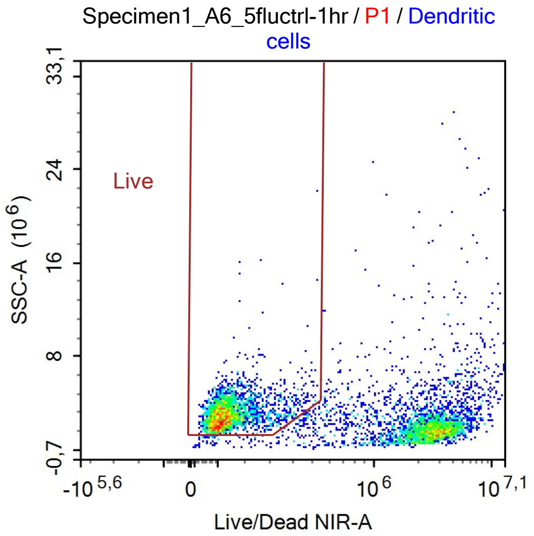

Gate	Count	% Dendritic cells
Dendritic cells	6.325	100,00%
Live	3.129	49,47%

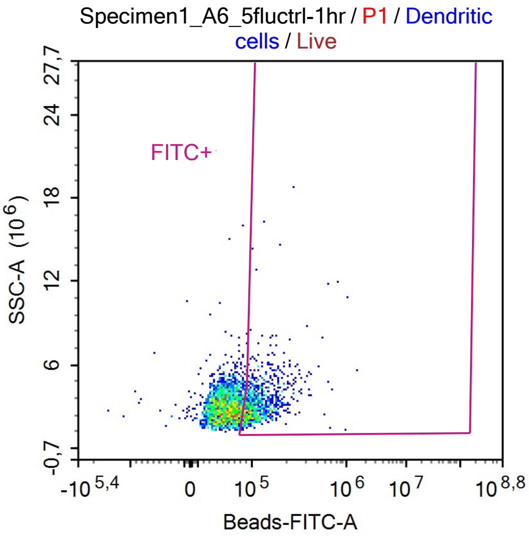

Gate	Count	% Live
Live	3.129	100,00%
FITC+	938	29,98%

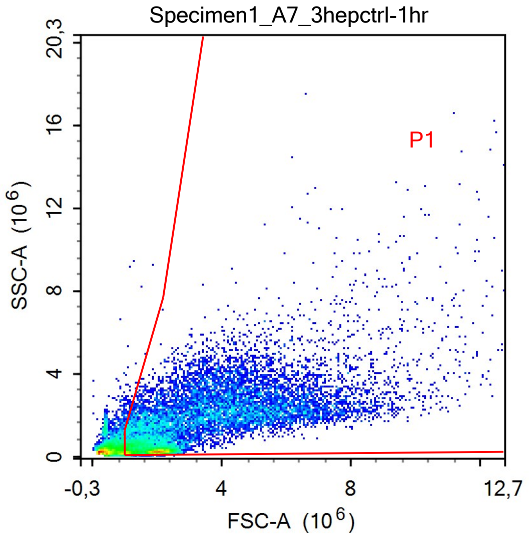

Gate	Count	% All
All	28.668	100,00%
P1	17.344	60,50%

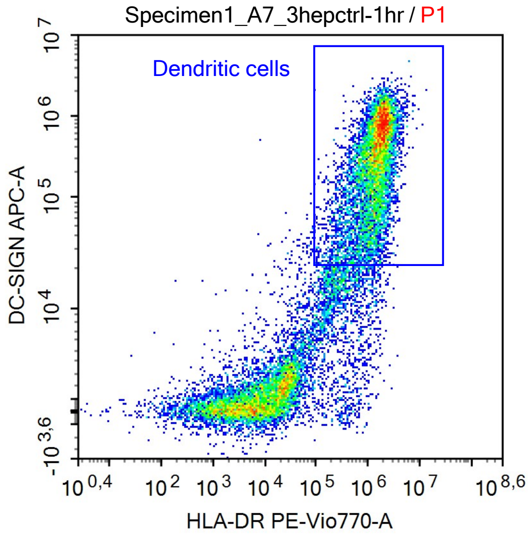

Gate	Count	% P1
P1	17.344	100,00%
Dendritic cells	8.240	47,51%

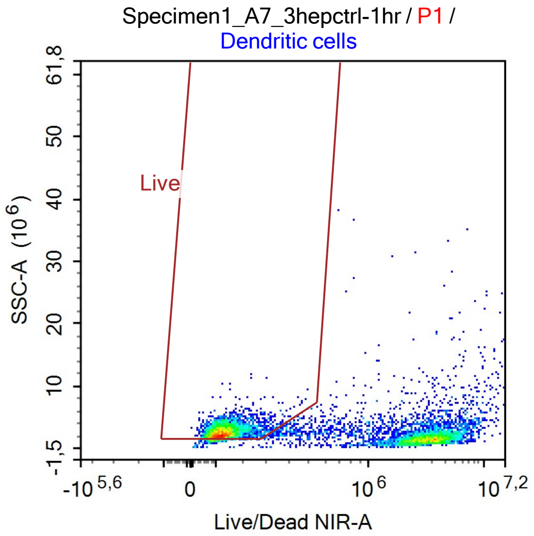

Gate	Count	% Dendritic cells
Dendritic cells	8.240	100,00%
Live	3.975	48,24%

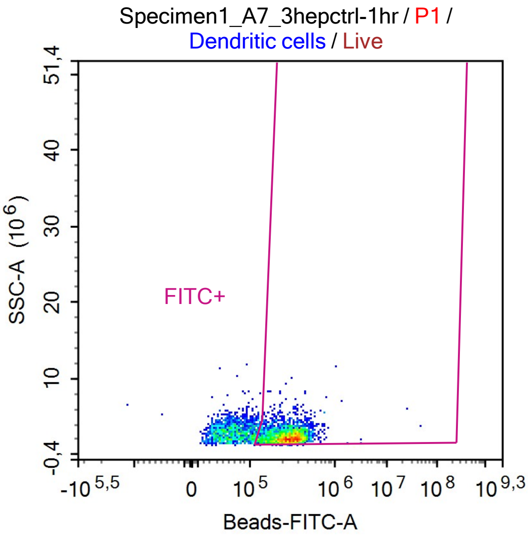

Gate	Count	% Live
Live	3.975	100,00%
FITC+	2.546	64,05%

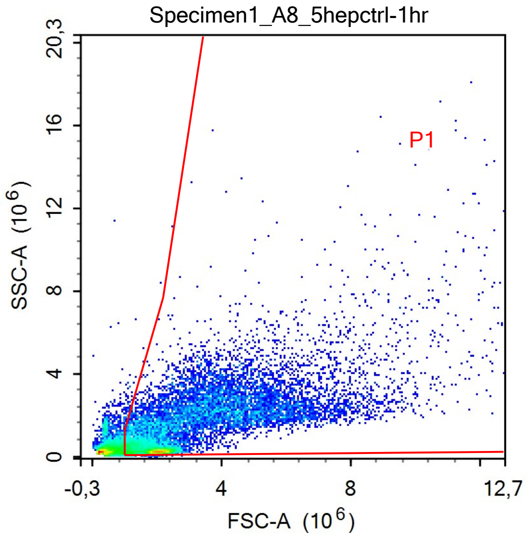

Gate	Count	% All
All	29.180	100,00%
P1	17.635	60,44%

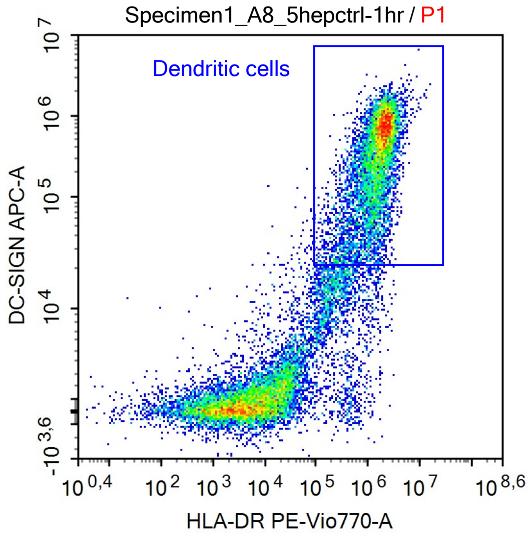

Gate	Count	% P1
P1	17.635	100,00%
Dendritic cells	7.200	40,83%

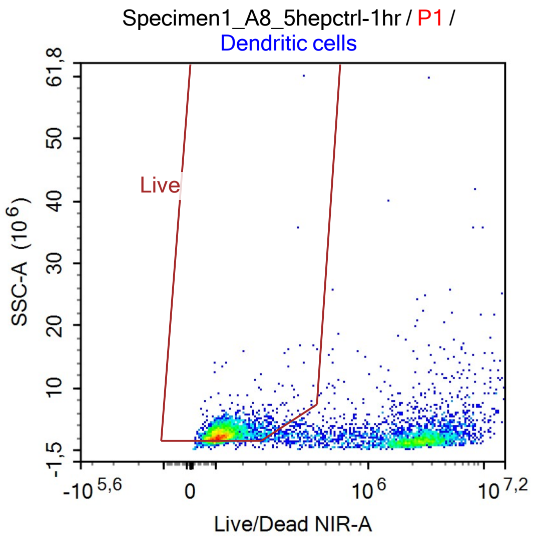

Gate	Count	% Dendritic cells
Dendritic cells	7.200	100,00%
Live	3.927	54,54%

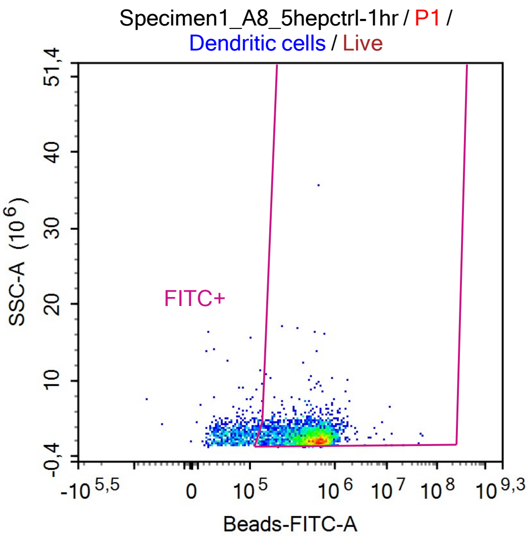

Gate	Count	% Live
Live	3.927	100,00%
FITC+	3.249	82,73%

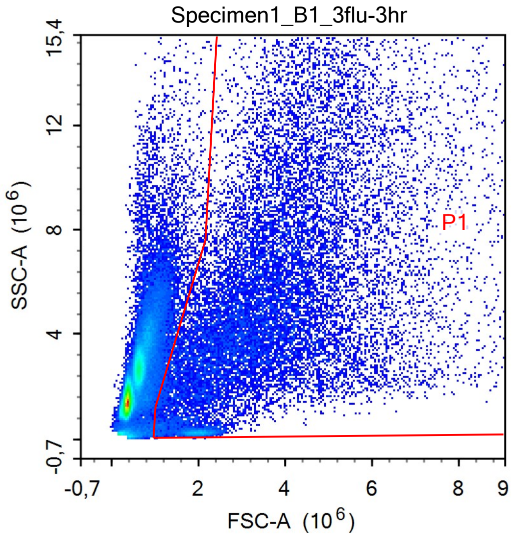

Gate	Count	% All
All	219.310	100,00%
P1	24.144	11,01%

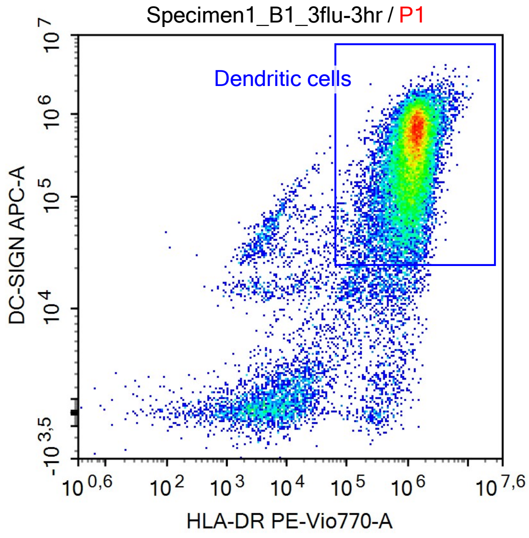

Gate	Count	% P1
P1	24.144	100,00%
Dendritic cells	19.132	79,24%

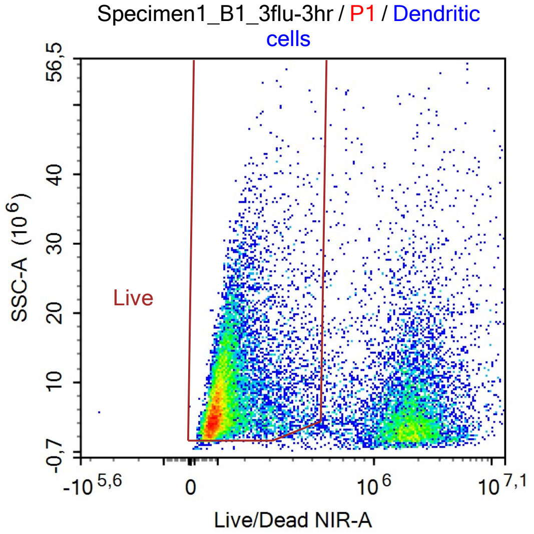

Gate	Count	% Dendritic cells
Dendritic cells	19.132	100,00%
Live	12.288	64,23%

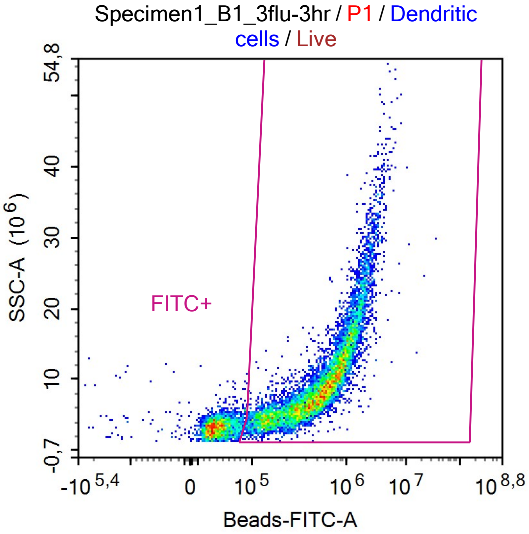

Gate	Count	% Live
Live	12.288	100,00%
FITC+	9.880	80,40%

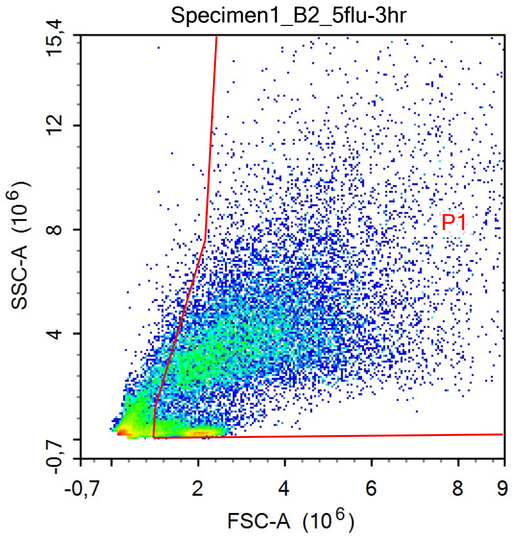

Gate	Count	% All
All	31.682	100,00%
P1	20.105	63,46%

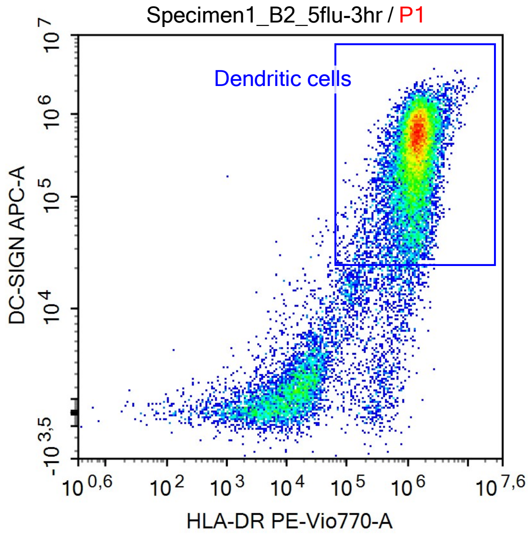

Gate	Count	% P1
P1	20.105	100,00%
Dendritic cells	14.147	70,37%

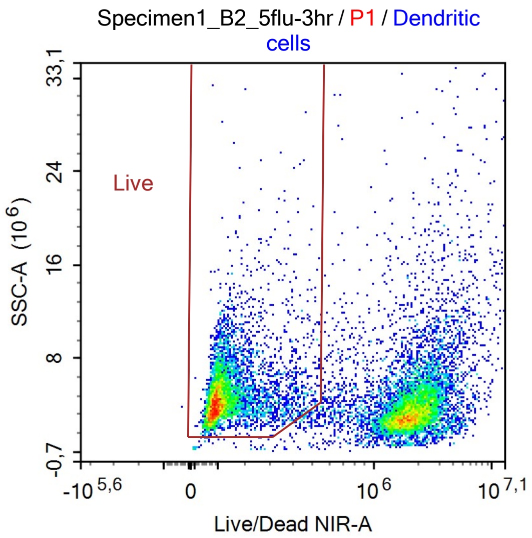

Gate	Count	% Dendritic cells
Dendritic cells	14.147	100,00%
Live	6.231	44,04%

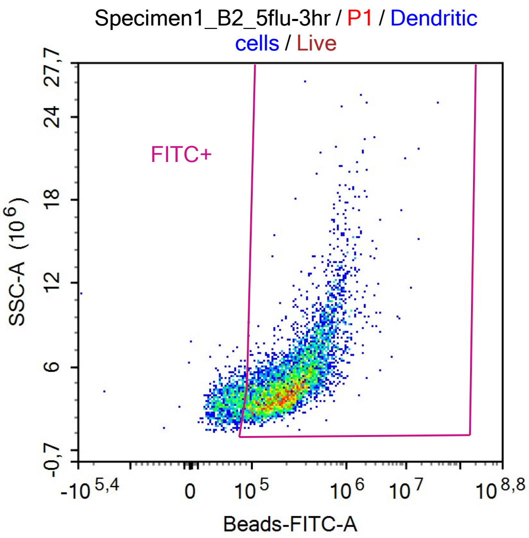

Gate	Count	% Live
Live	6.231	100,00%
FITC+	5.225	83,85%

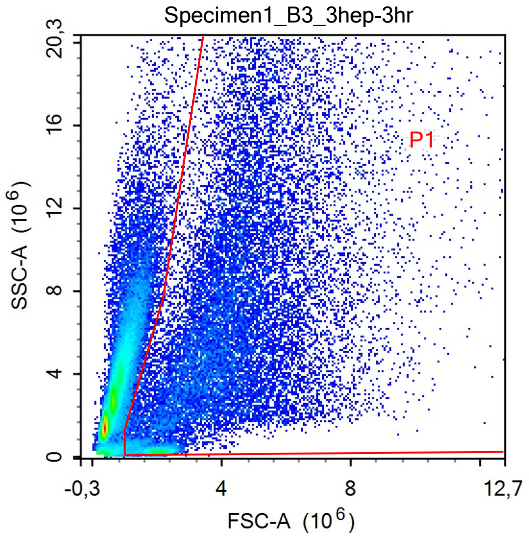

Gate	Count	% All
All	146.170	100,00%
P1	30.405	20,80%

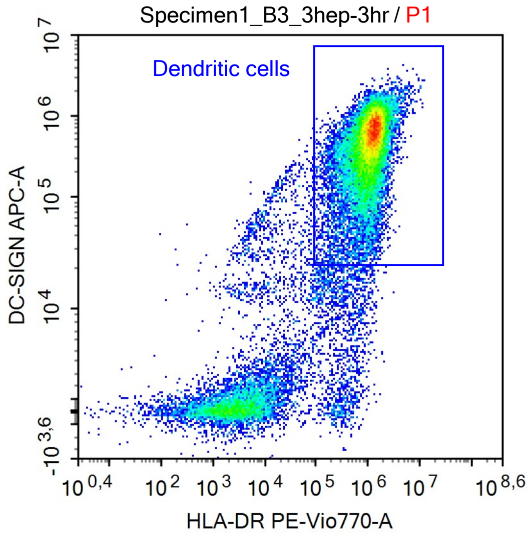

Gate	Count	% P1
P1	30.405	100,00%
Dendritic cells	21.842	71,84%

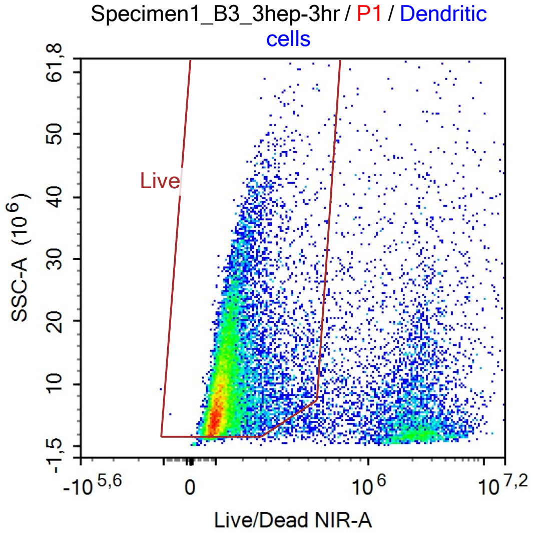

Gate	Count	% Dendritic cells
Dendritic cells	21.842	100,00%
Live	16.998	77,82%

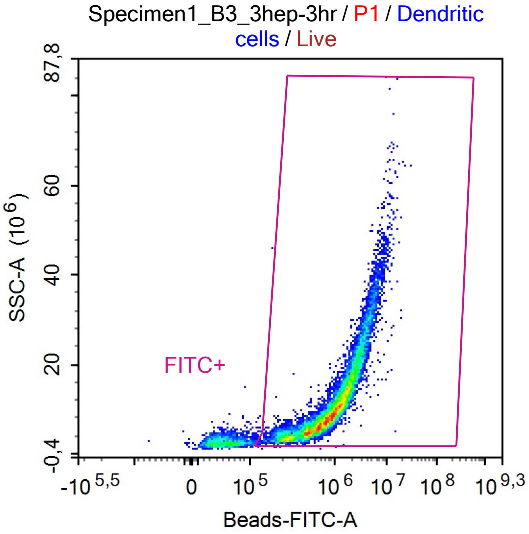

Gate	Count	% Live
Live	16.998	100,00%
FITC+	14.975	88,10%

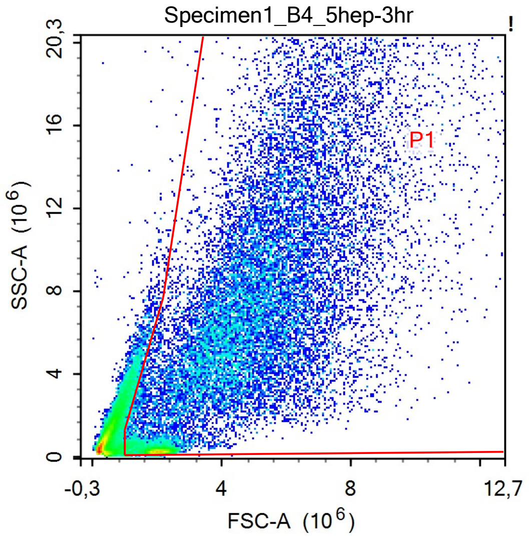

Gate	Count	% All
All	55.074	100,00%
P1	28.120	51,06%

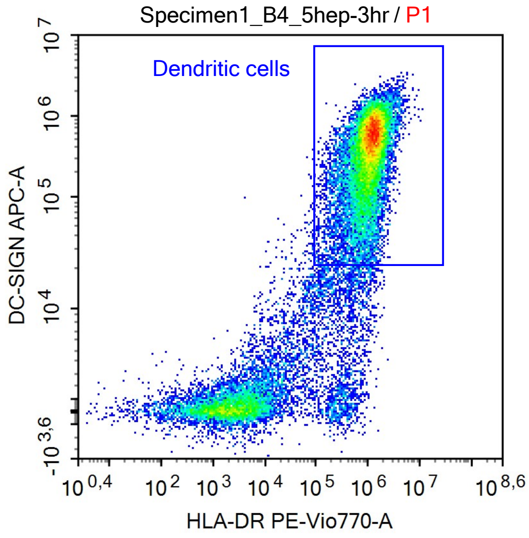

Gate	Count	% P1
P1	28.120	100,00%
Dendritic cells	18.394	65,41%

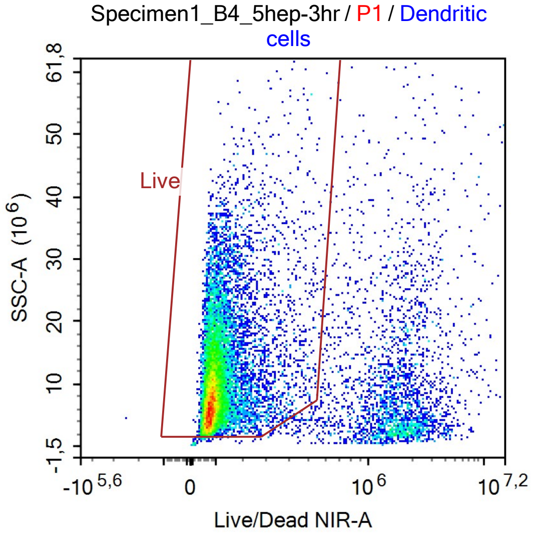

Gate	Count	% Dendritic cells
Dendritic cells	18.394	100,00%
Live	15.583	84,72%

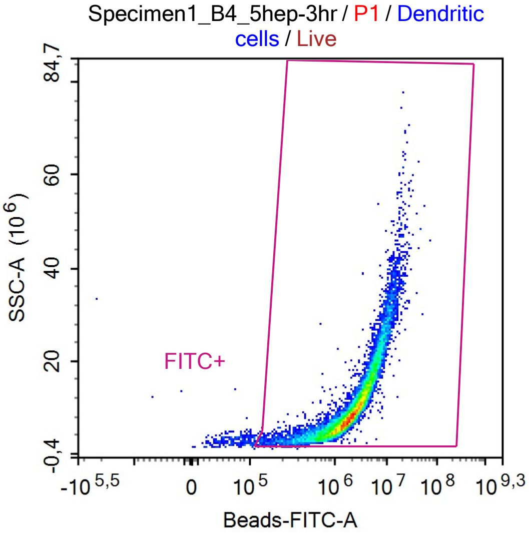

Gate	Count	% Live
Live	15.583	100,00%
FITC+	15.243	97,82%

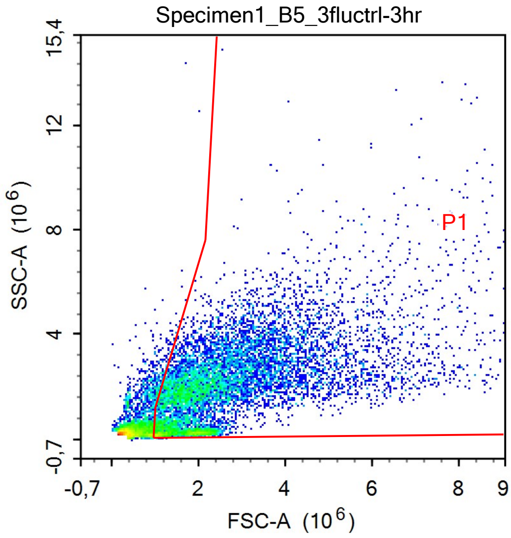

Gate	Count	% All
All	17.763	100,00%
P1	9.689	54,55%

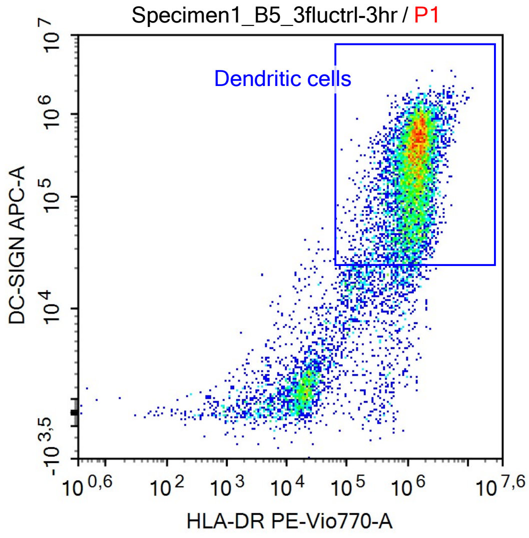

Gate	Count	% P1
P1	9.689	100,00%
Dendritic cells	6.945	71,68%

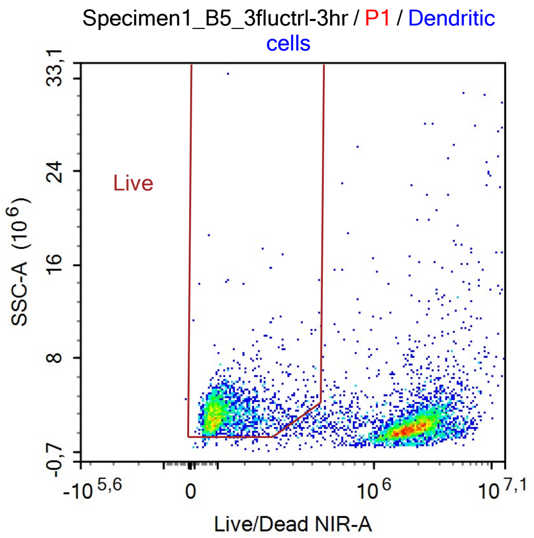

Gate	Count	% Dendritic cells
Dendritic cells	6.945	100,00%
Live	2.564	36,92%

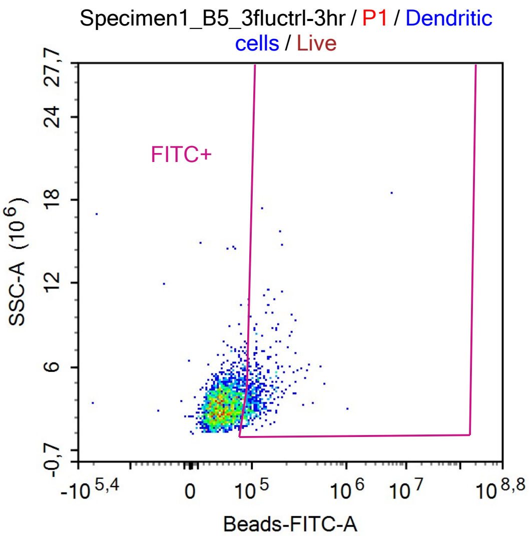

Gate	Count	% Live
Live	2.564	100,00%
FITC+	436	17,00%

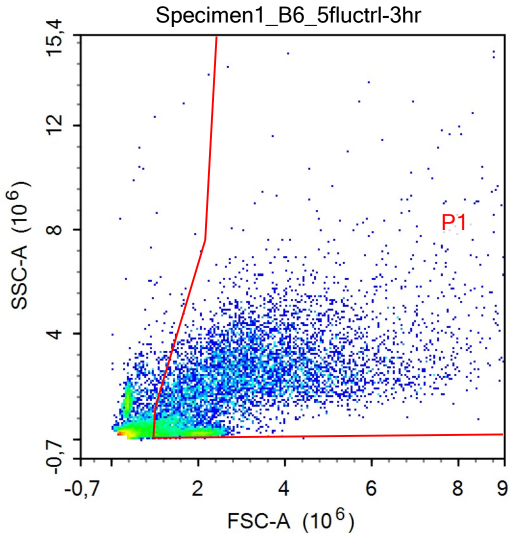

Gate	Count	% All
All	18.028	100,00%
P1	8.427	46,74%

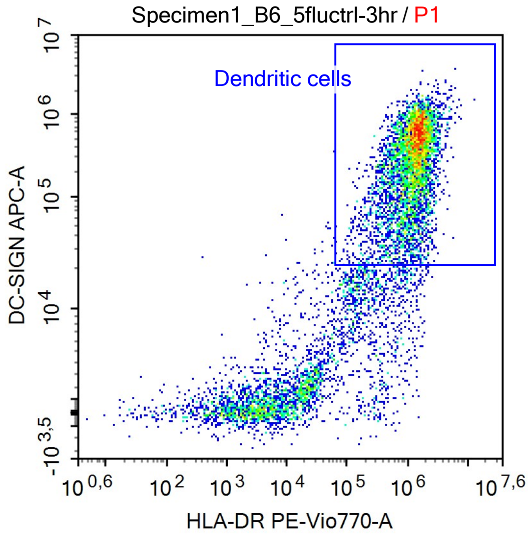

Gate	Count	% P1
P1	8.427	100,00%
Dendritic cells	5.159	61,22%

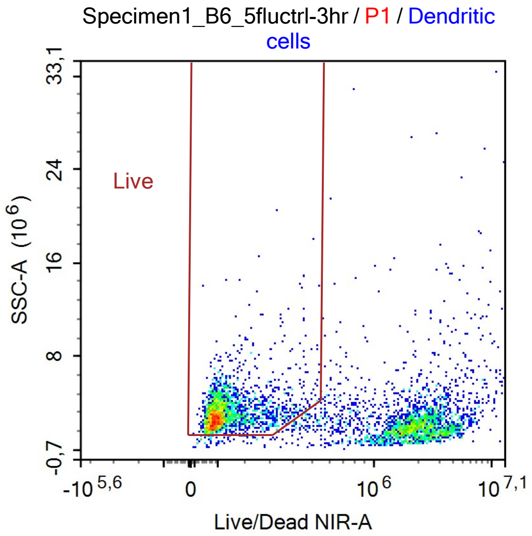

Gate	Count	% Dendritic cells
Dendritic cells	5.159	100,00%
Live	2.670	51,75%

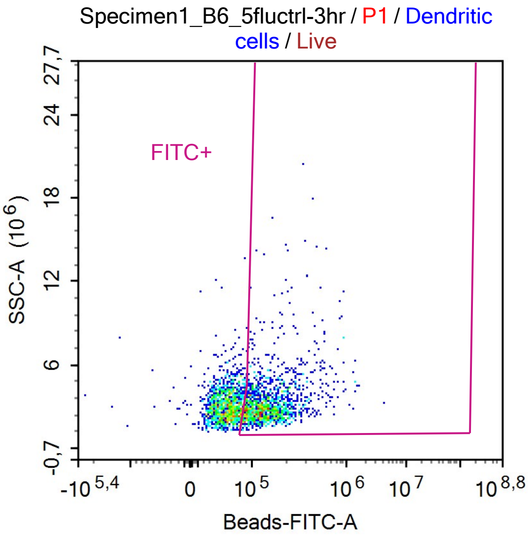

Gate	Count	% Live
Live	2.670	100,00%
FITC+	1.362	51,01%

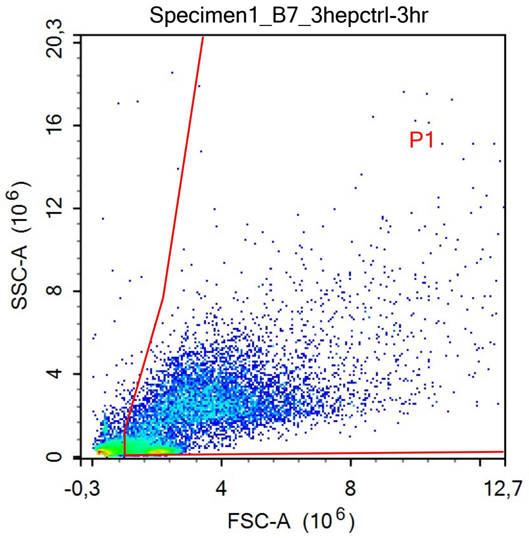

Gate	Count	% All
All	21.606	100,00%
P1	12.670	58,64%

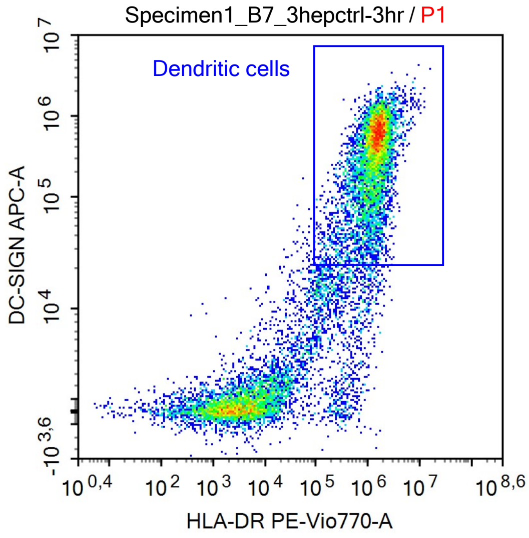

Gate	Count	% P1
P1	12.670	100,00%
Dendritic cells	6.380	50,36%

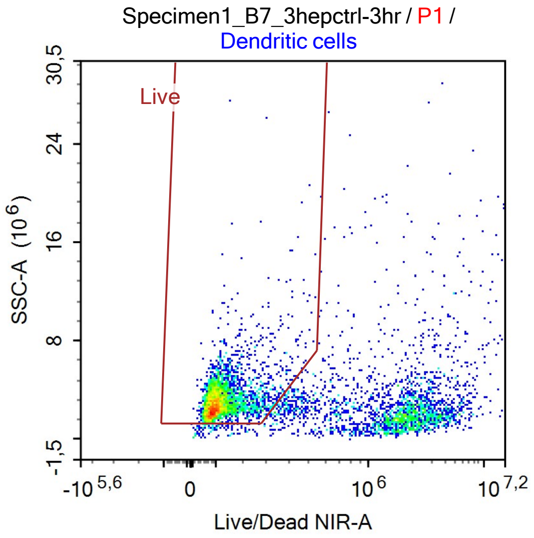

Gate	Count	% Dendritic cells
Dendritic cells	6.380	100,00%
Live	3.940	61,76%

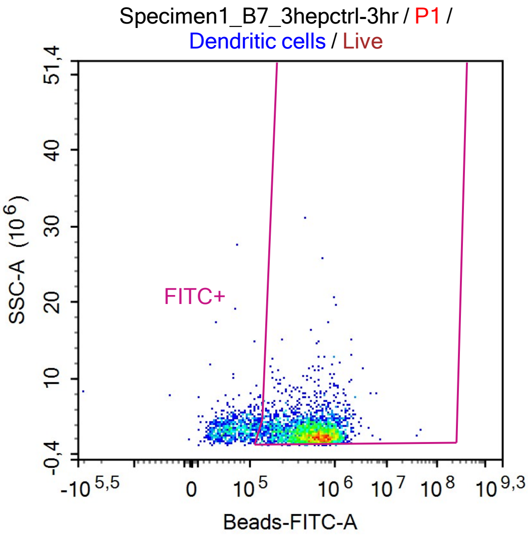

Gate	Count	% Live
Live	3.940	100,00%
FITC+	3.214	81,57%

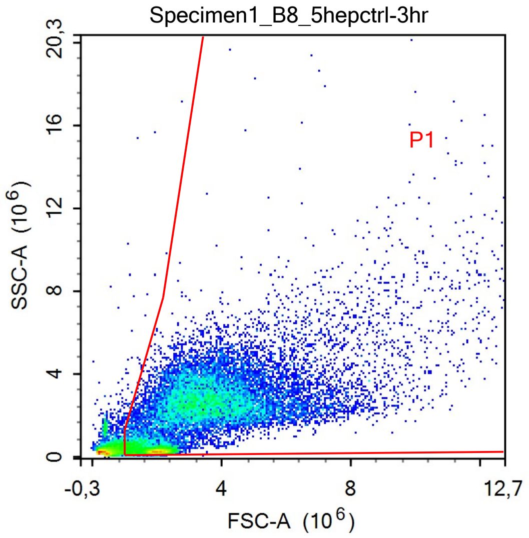

Gate	Count	% All
All	30.738	100,00%
P1	20.319	66,10%

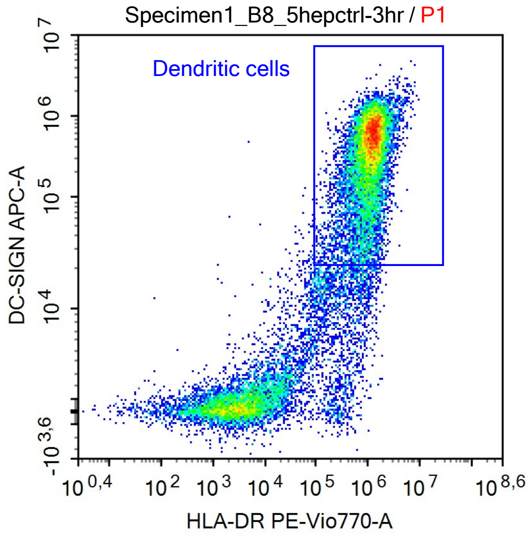

Gate	Count	% P1
P1	20.319	100,00%
Dendritic cells	11.878	58,46%

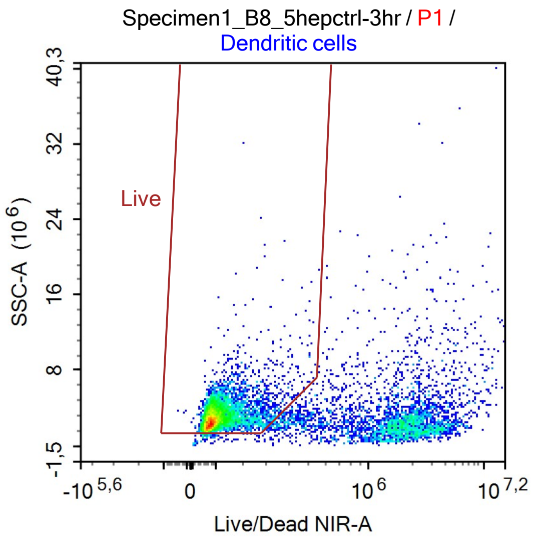

Gate	Count	% Dendritic cells
Dendritic cells	11.878	100,00%
Live	8.515	71,69%

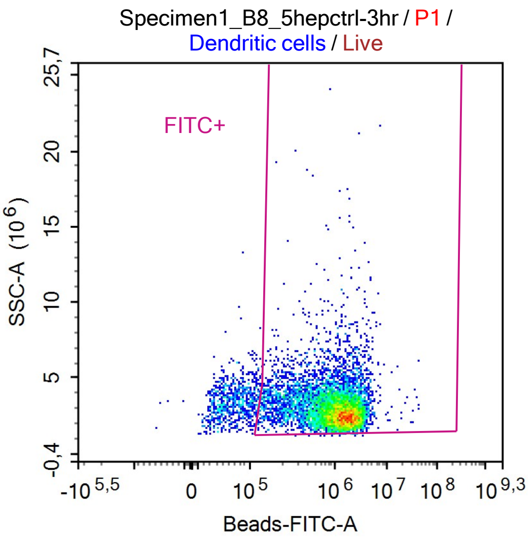

Gate	Count	% Live
Live	8.515	100,00%
FITC+	7.671	90,09%

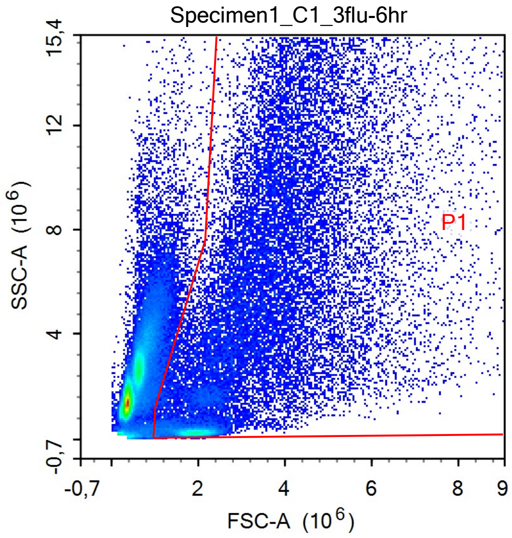

Gate	Count	% All
All	257.344	100,00%
P1	43.851	17,04%

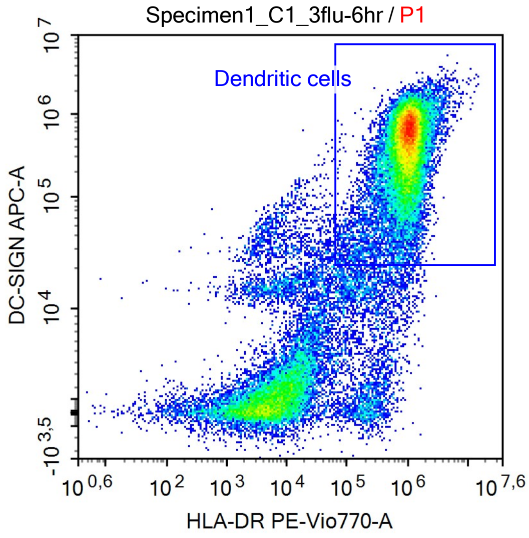

Gate	Count	% P1
P1	43.851	100,00%
Dendritic cells	25.792	58,82%

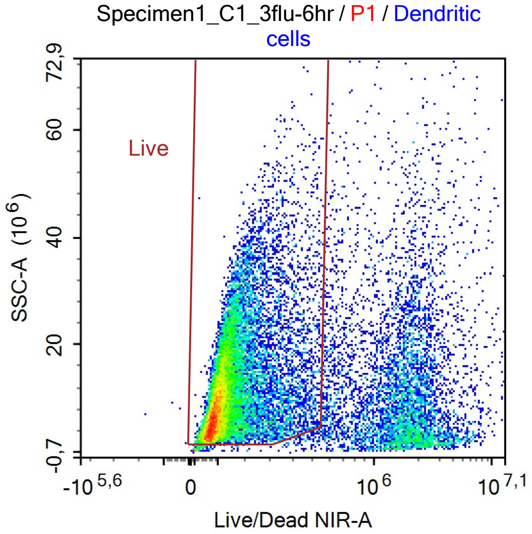

Gate	Count	% Dendritic cells
Dendritic cells	25.792	100,00%
Live	19.496	75,59%

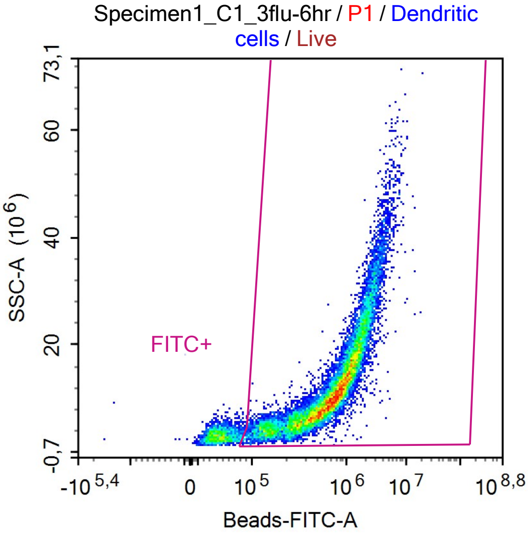

Gate	Count	% Live
Live	19.496	100,00%
FITC+	17.339	88,94%

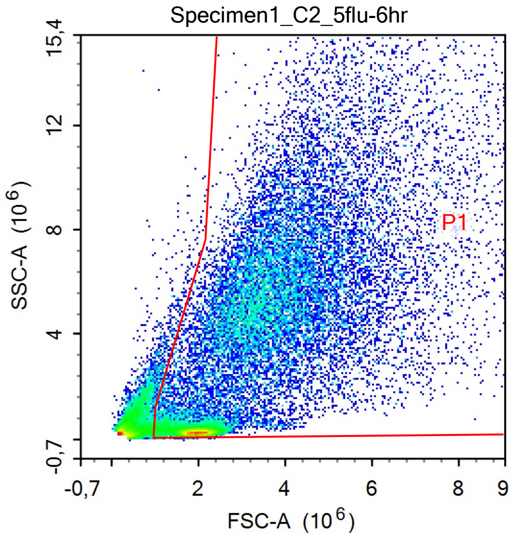

Gate	Count	% All
All	46.559	100,00%
P1	33.177	71,26%

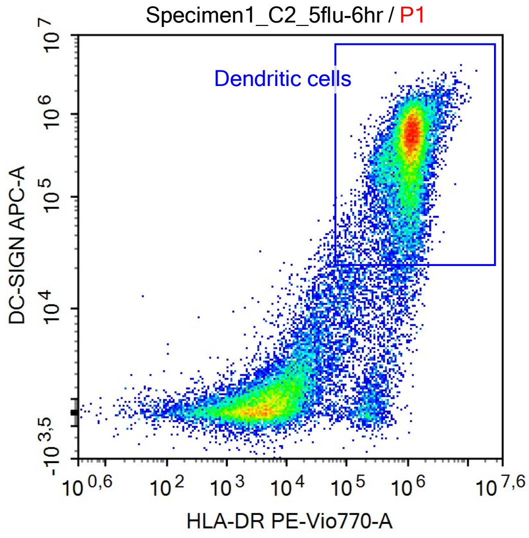

Gate	Count	% P1
P1	33.177	100,00%
Dendritic cells	16.337	49,24%

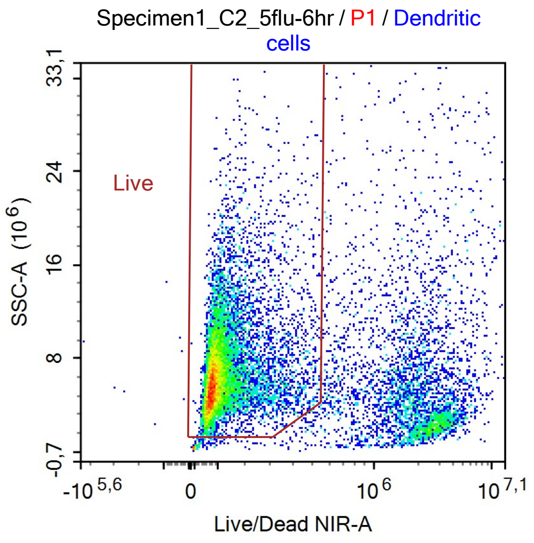

Gate	Count	% Dendritic cells
Dendritic cells	16.337	100,00%
Live	12.068	73,87%

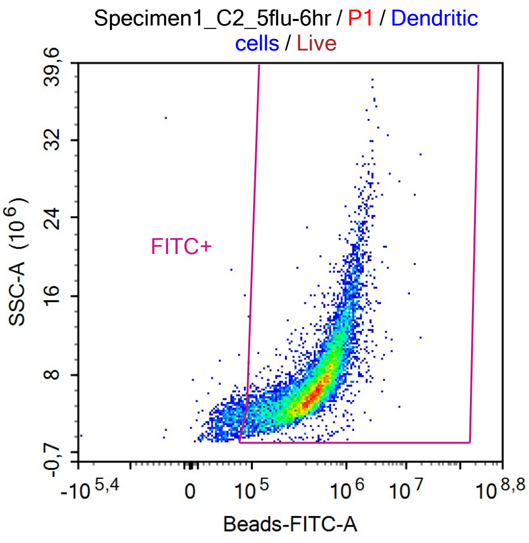

Gate	Count	% Live
Live	12.068	100,00%
FITC+	11.347	94,03%

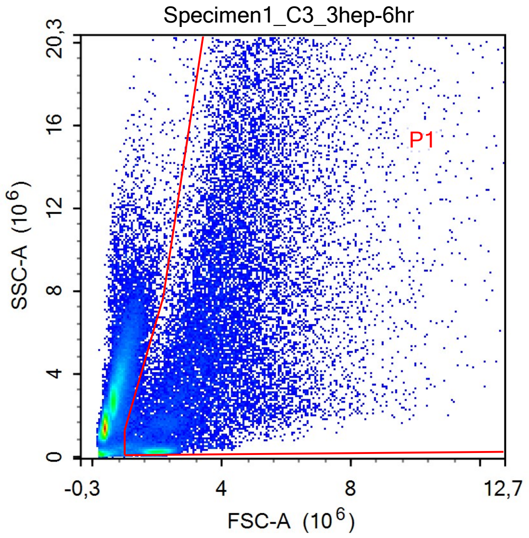

Gate	Count	% All
All	220.768	100,00%
P1	38.906	17,62%

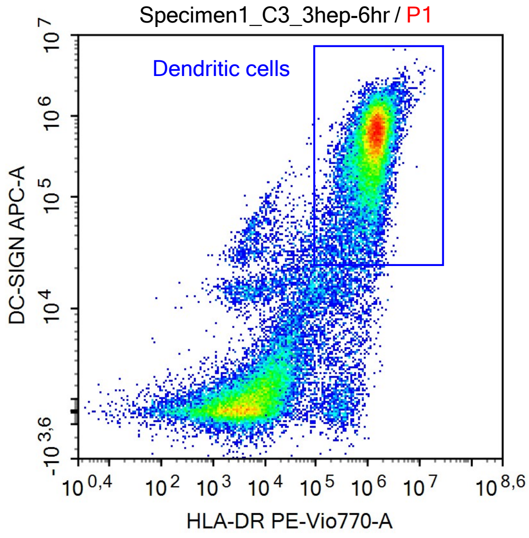

Gate	Count	% P1
P1	38.906	100,00%
Dendritic cells	18.726	48,13%

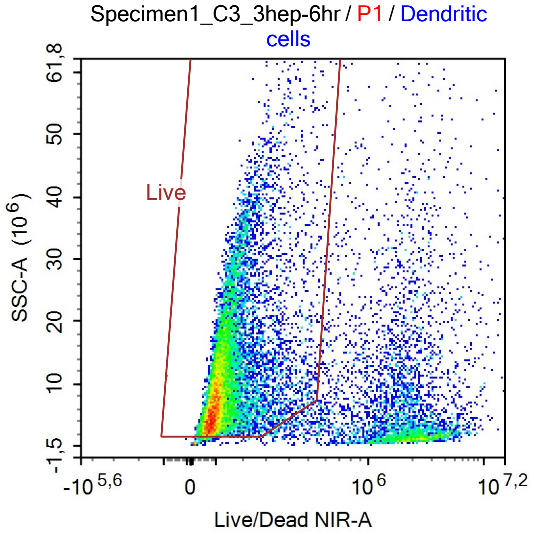

Gate	Count	% Dendritic cells
Dendritic cells	18.726	100,00%
Live	14.246	76,08%

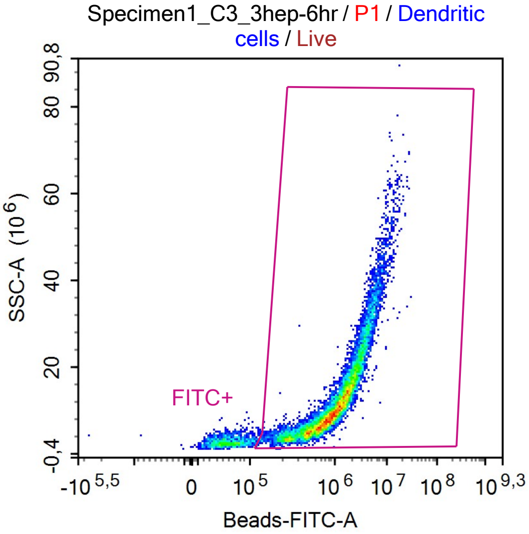

Gate	Count	% Live
Live	14.246	100,00%
FITC+	12.513	87,84%

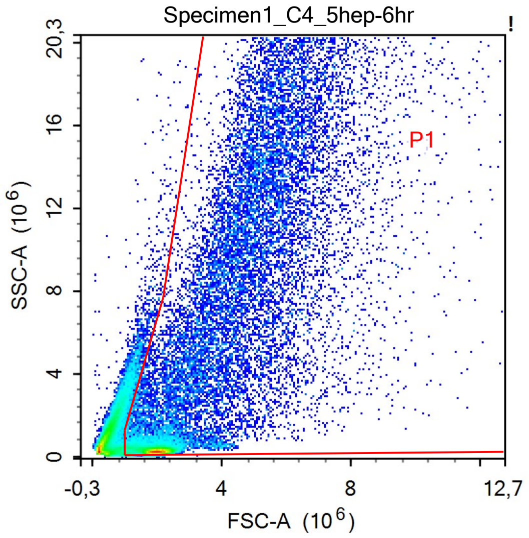

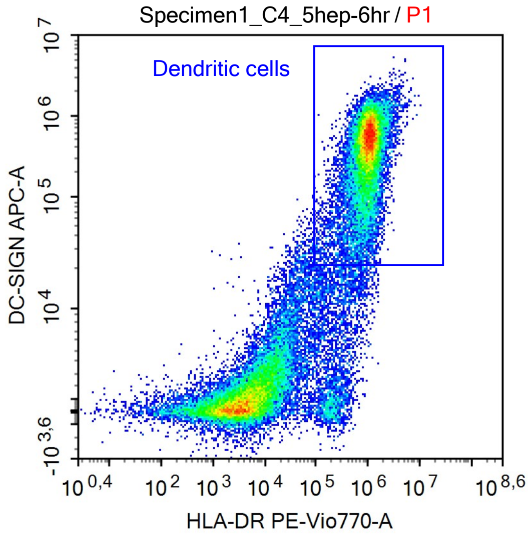

Gate	Count	% All
All	70.351	100,00%
P1	41.895	59,55%

Gate	Count	% P1
P1	41.895	100,00%
Dendritic cells	17.353	41,42%

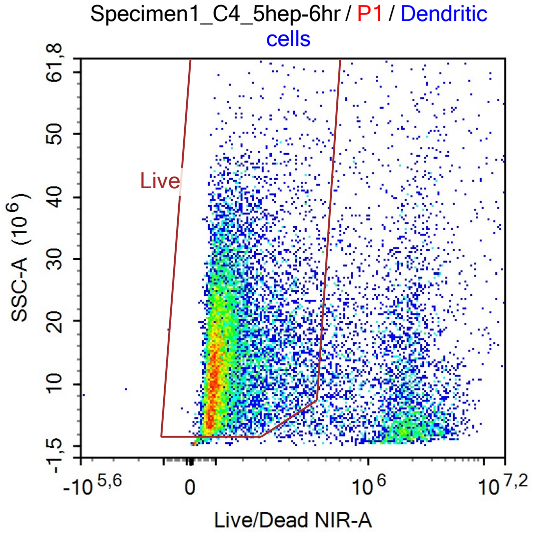

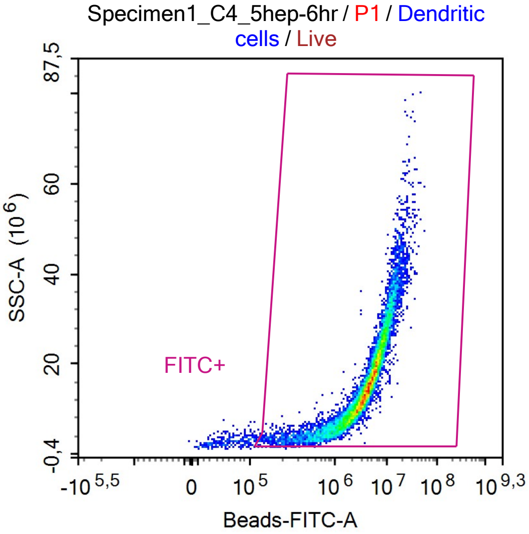

Gate	Count	% Dendritic cells
Dendritic cells	17.353	100,00%
Live	13.056	75,24%

Gate	Count	% Live
Live	13.056	100,00%
FITC+	12.742	97,59%

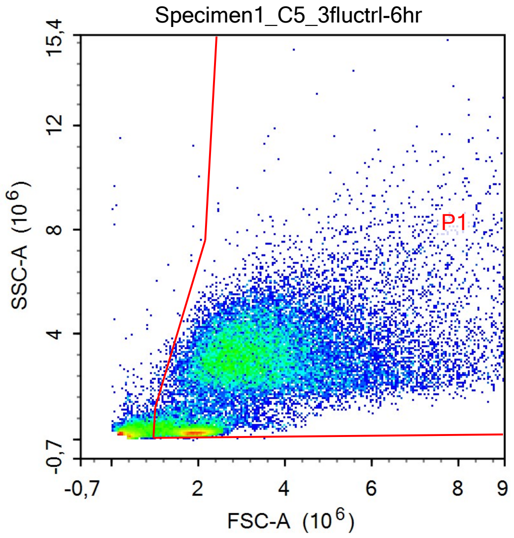

Gate	Count	% All
All	40.616	100,00%
P1	32.634	80,35%

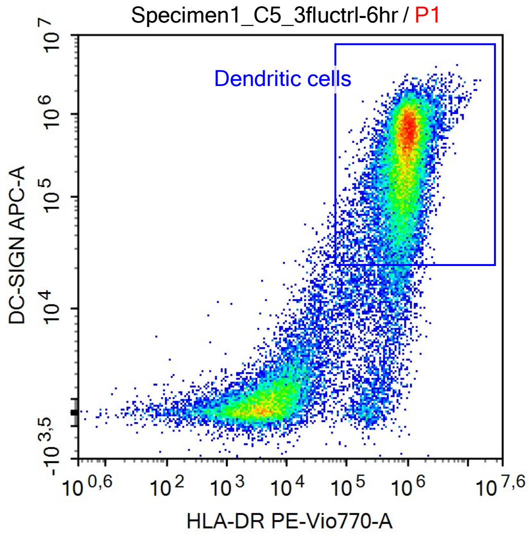

Gate	Count	% P1
P1	32.634	100,00%
Dendritic cells	18.975	58,14%

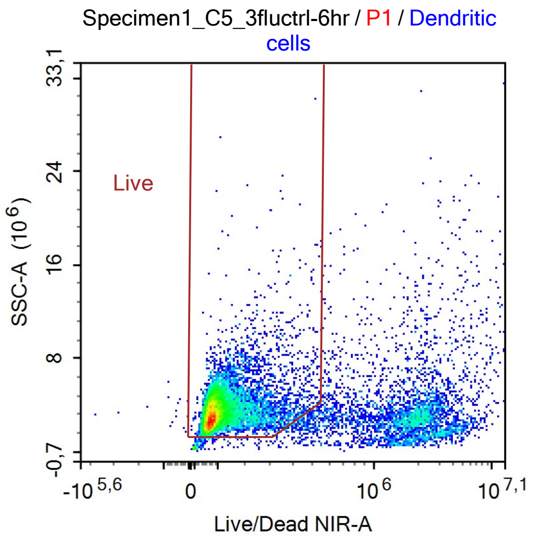

Gate	Count	% Dendritic cells
Dendritic cells	18.975	100,00%
Live	14.836	78,19%

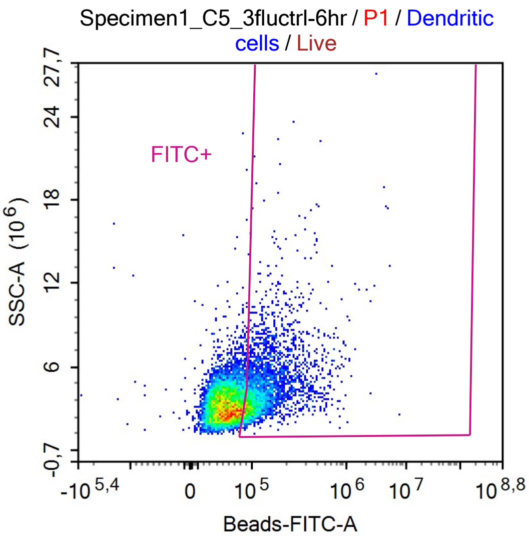

Gate	Count	% Live
Live	14.836	100,00%
FITC+	4.028	27,15%

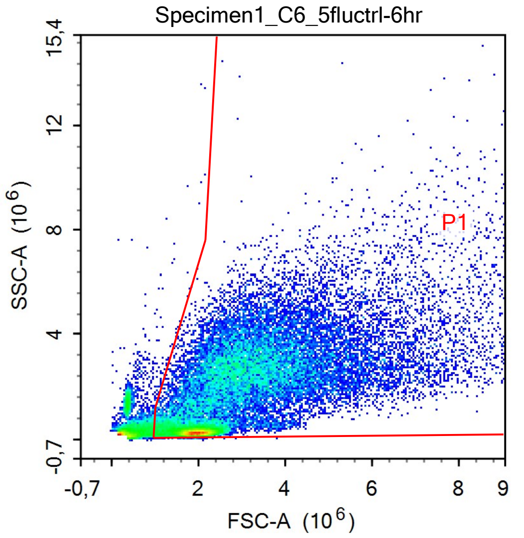

Gate	Count	% All
All	50.549	100,00%
P1	39.867	78,87%

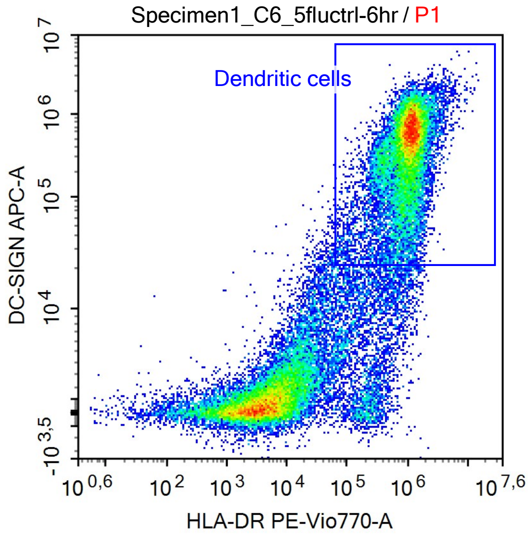

Gate	Count	% P1
P1	39.867	100,00%
Dendritic cells	17.251	43,27%

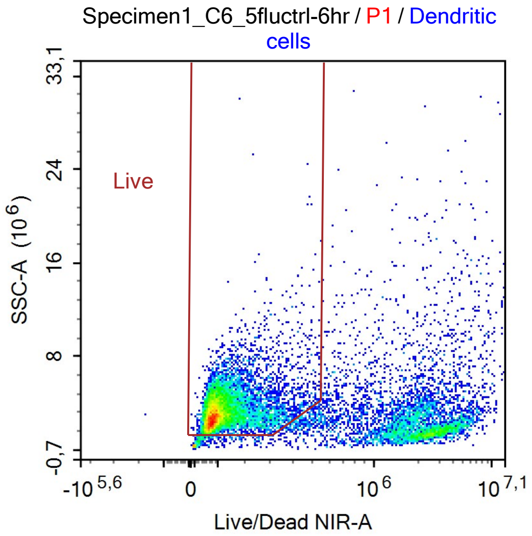

Gate	Count	% Dendritic cells
Dendritic cells	17.251	100,00%
Live	11.761	68,18%

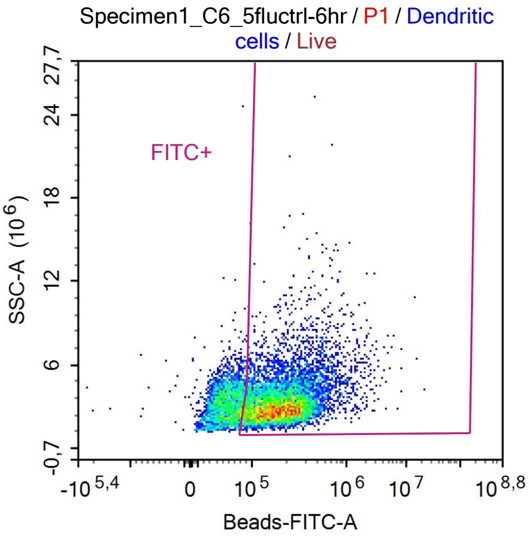

Gate	Count	% Live
Live	11.761	100,00%
FITC+	8.591	73,05%

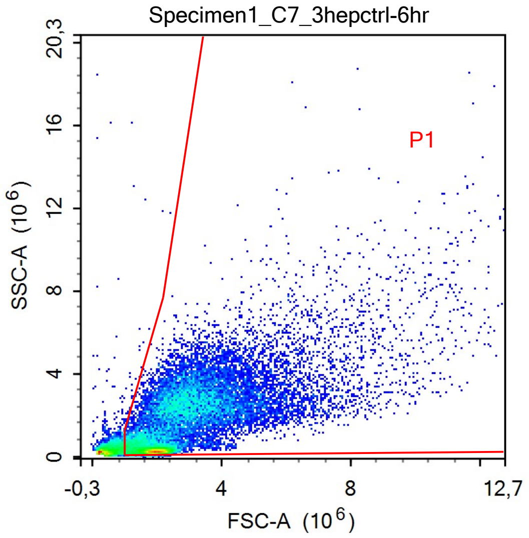

Gate	Count	% All
All	38.366	100,00%
P1	27.628	72,01%

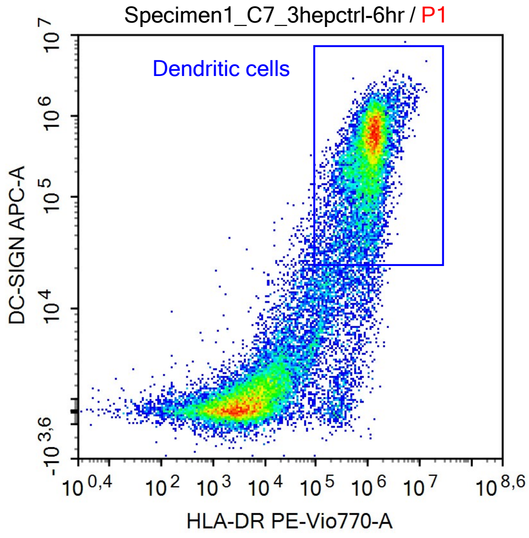

Gate	Count	% P1
P1	27.628	100,00%
Dendritic cells	11.228	40,64%

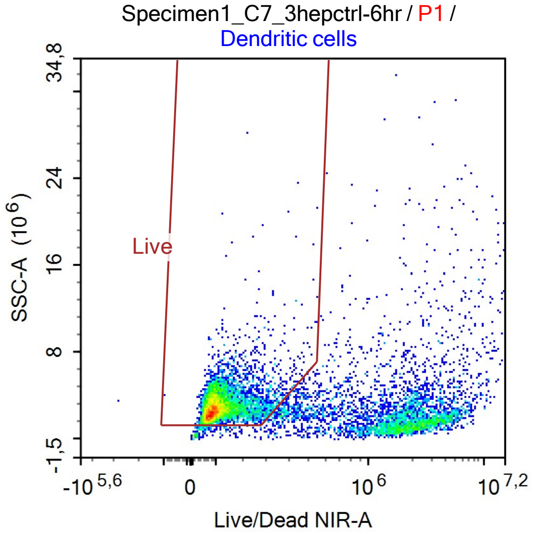

Gate	Count	% Dendritic cells
Dendritic cells	11.228	100,00%
Live	7.078	63,04%

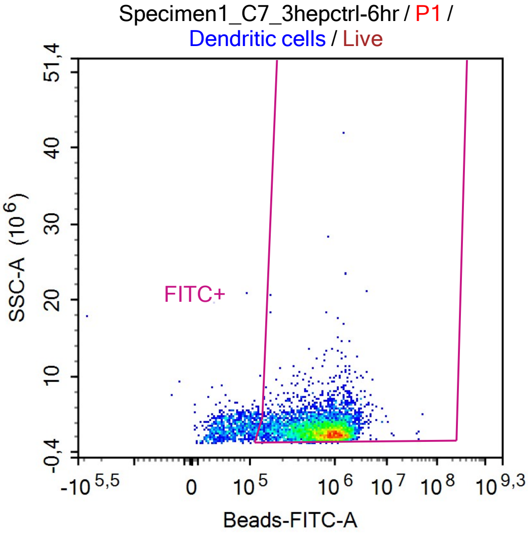

Gate	Count	% Live
Live	7.078	100,00%
FITC+	6.179	87,30%

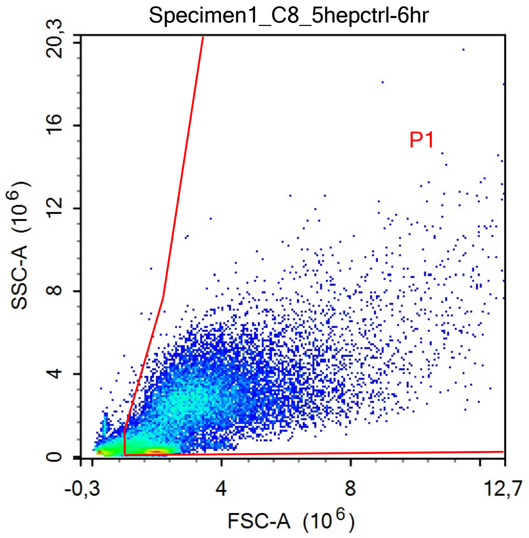

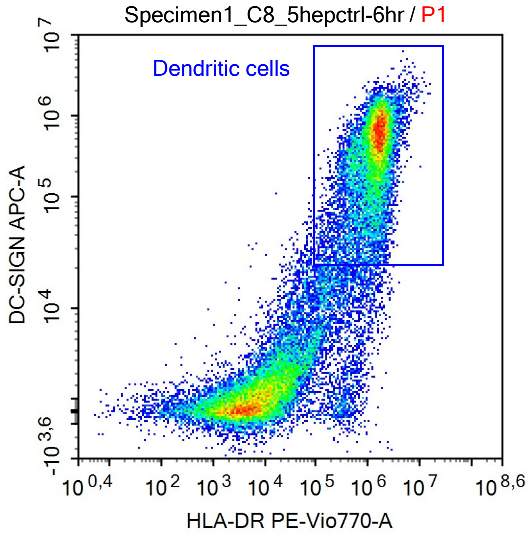

Gate	Count	% All
All	42.322	100,00%
P1	31.366	74,11%

Gate	Count	% P1
P1	31.366	100,00%
Dendritic cells	12.630	40,27%

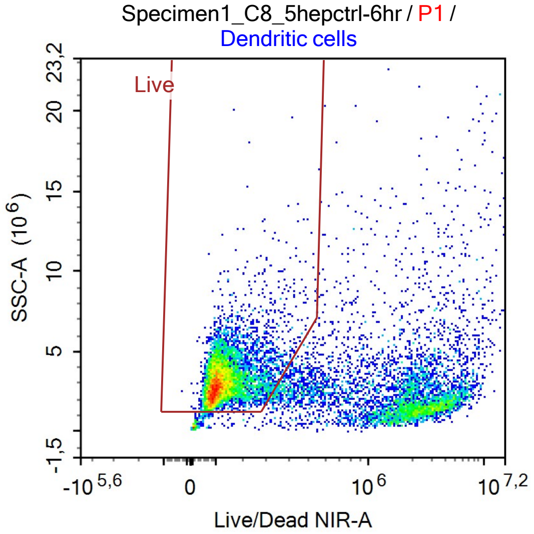

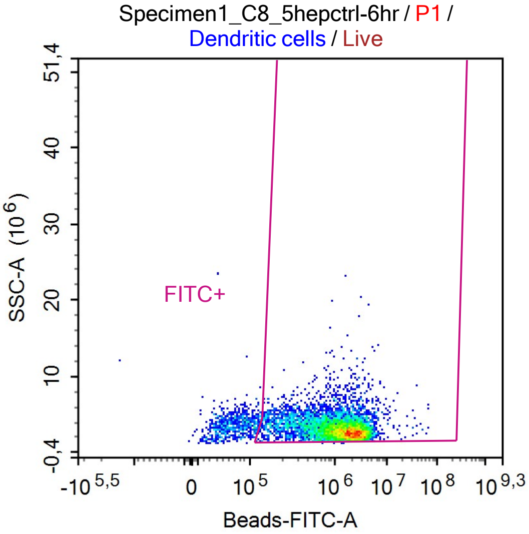

Gate	Count	% Dendritic cells
Dendritic cells	12.630	100,00%
Live	7.340	58,12%

Gate	Count	% Live
Live	7.340	100,00%
FITC+	6.605	89,99%

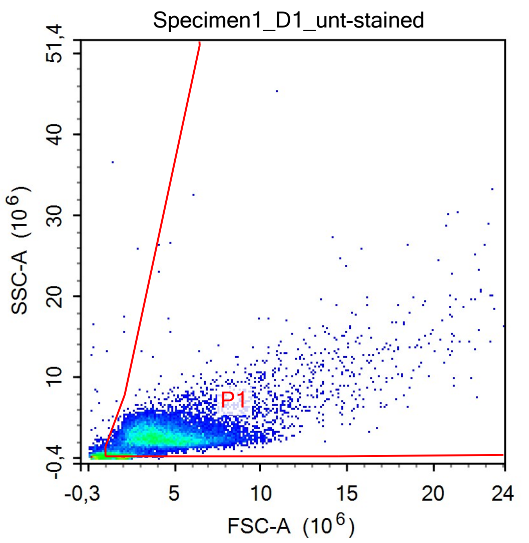

Gate	Count	% All
All	32.009	100,00%
P1	24.511	76,58%

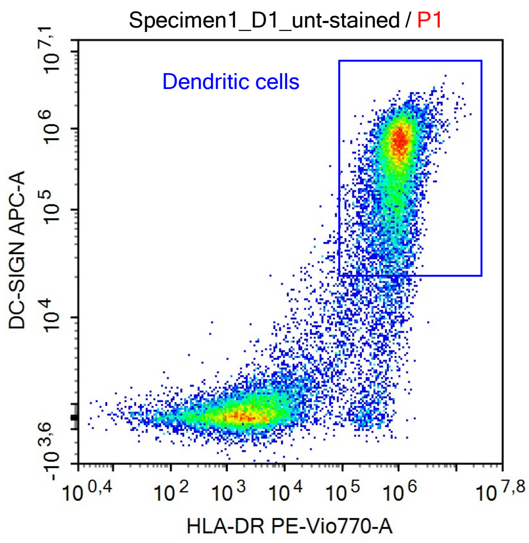

Gate	Count	% P1
P1	24.511	100,00%
Dendritic cells	12.236	49,92%

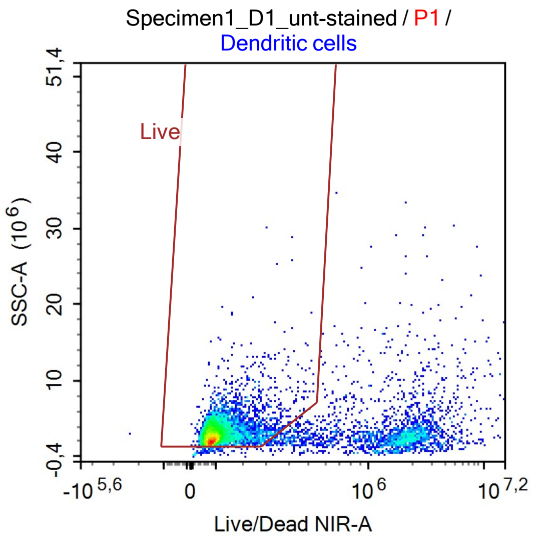

Gate	Count	% Dendritic cells
Dendritic cells	12.236	100,00%
Live	9.839	80,41%

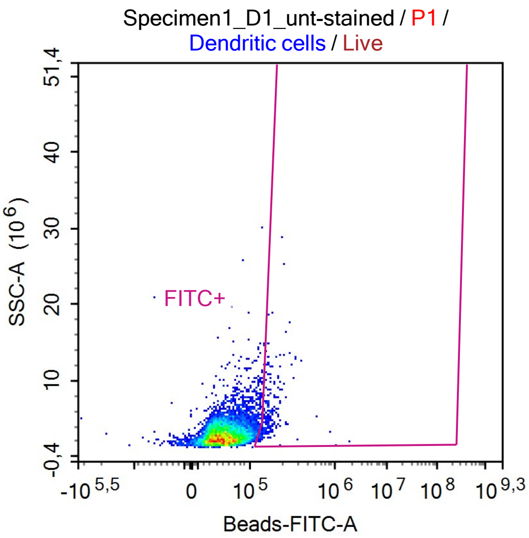

Gate	Count	% Live
Live	9.839	100,00%
FITC+	165	1,68%

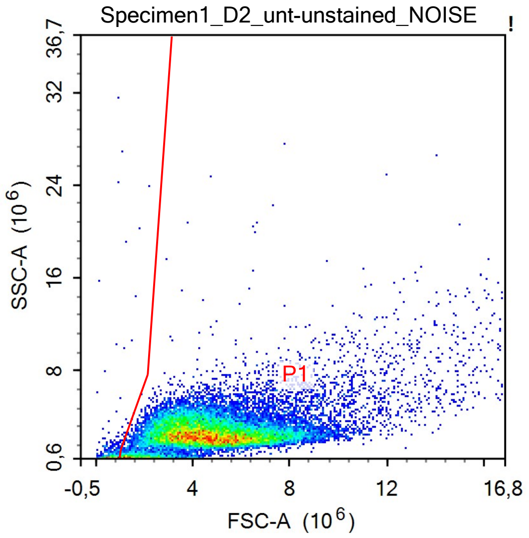

Gate	Count	% All
All	45.422	100,00%
P1	37.646	82,88%

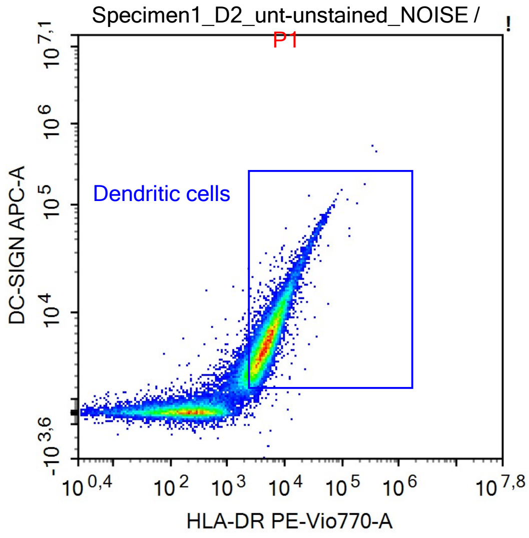

Gate	Count	% P1
P1	37.646	100,00%
Dendritic cells	17.638	46,85%

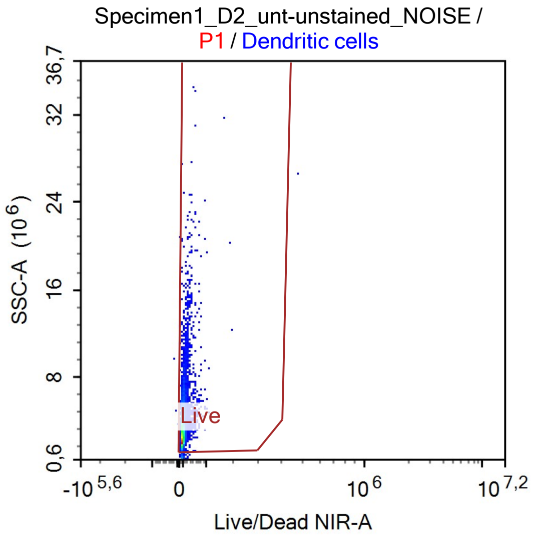

Gate	Count	% Dendritic cells
Dendritic cells	17.638	100,00%
Live	17.236	97,72%

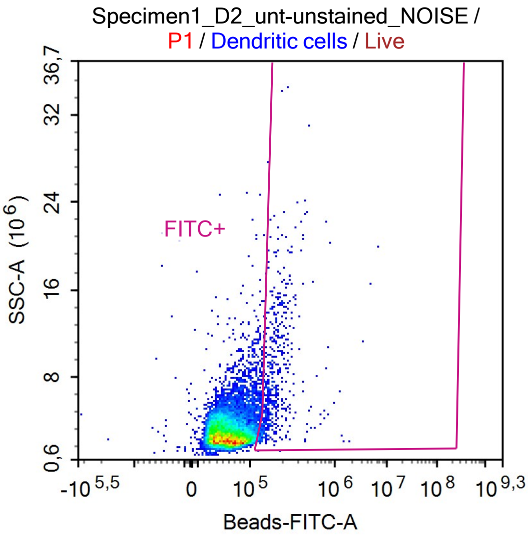

Gate	Count	% Live
Live	17.236	100,00%
FITC+	544	3,16%

Donor 48 FLU

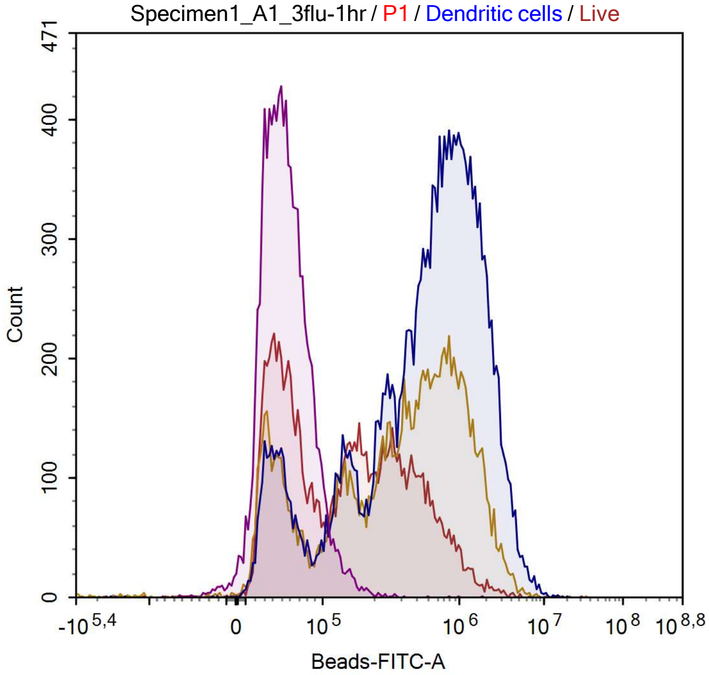

#	Sample	Gate	Count	Mean X	Median X
1	Specimen1_A1_3flu-1hr	Live	10.059	238.456	135.271
2	Specimen1_B1_3flu-3hr	Live*	12.288	581.865	336.735
3	Specimen1_C1_3flu-6hr	Live*	19.496	898.968	574.998
4	Specimen1_D1_unt-[illegible]	Live*	9.839	55.326	51.407

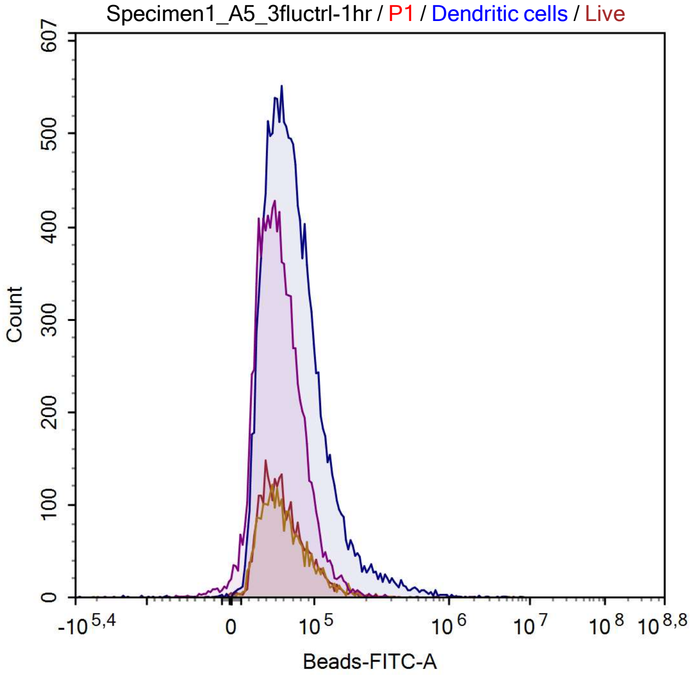

#	Sample	Gate	Count	Mean X	Median X
1	Specimen1_A5_3fluctrl-1hr	Live	2.855	67.465	55.033
2	Specimen1_B5_3fluctrl-3hr	Live*	2.564	64.231	55.131
3	Specimen1_C5_3fluctrl-6hr	Live*	14.836	80.145	65.771
4	Specimen1_D1_unt-[illegible]	Live*	9.839	55.326	51.407

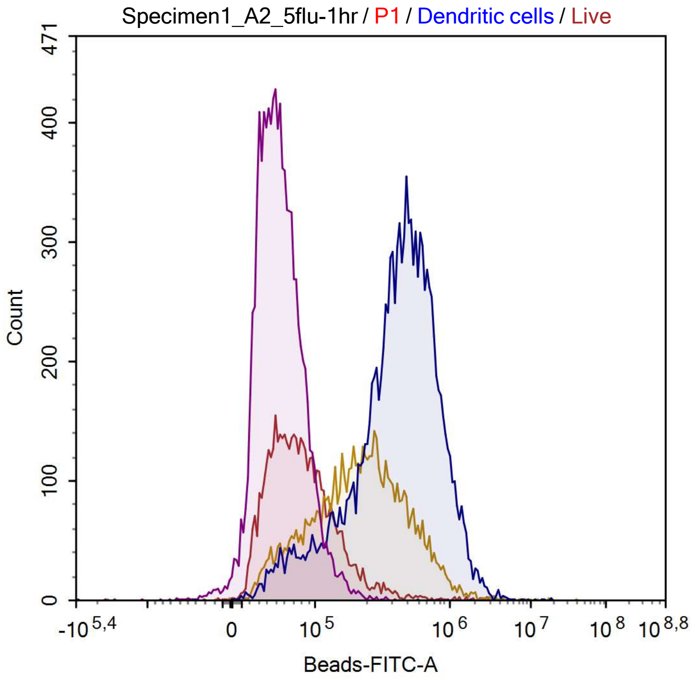

#	Sample	Gate	Count	Mean X	Median X
1	Specimen1_A2_5flu-1hr	Live	4.809	102.458	75.179
2	Specimen1_B2_5flu-3hr	Live*	6.231	276.649	184.316
3	Specimen1_C2_5flu-6hr	Live*	12.068	466.762	356.032
4	Specimen1_D1_unt-[illegible]	Live*	9.839	55.326	51.407

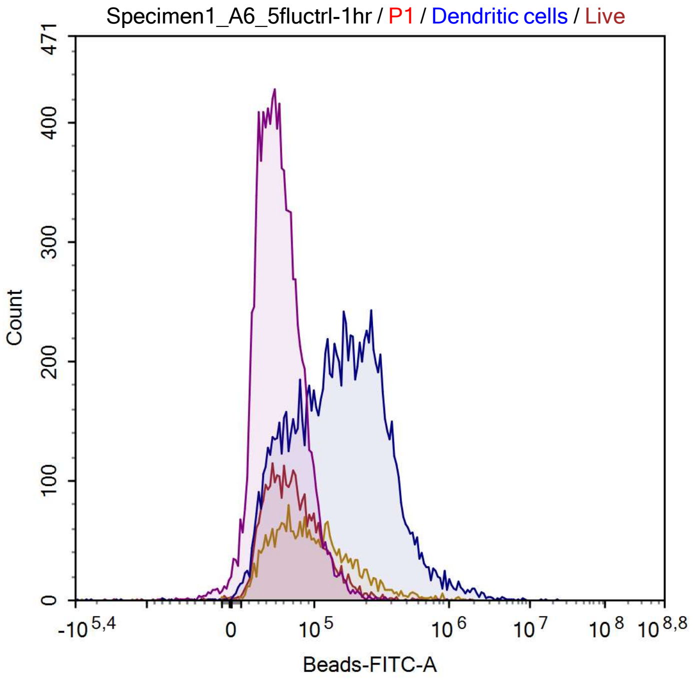

#	Sample	Gate	Count	Mean X	Median X
1	Specimen1_A6_5fluctrl-1hr	Live	3.129	74.690	67.320
2	Specimen1_B6_5fluctrl-3hr	Live*	2.670	117.200	89.041
3	Specimen1_C6_5fluctrl-6hr	Live*	11.761	197.478	140.344
4	Specimen1_D1_unt-[illegible]	Live*	9.839	55.326	51.407

Donor 48 HEP

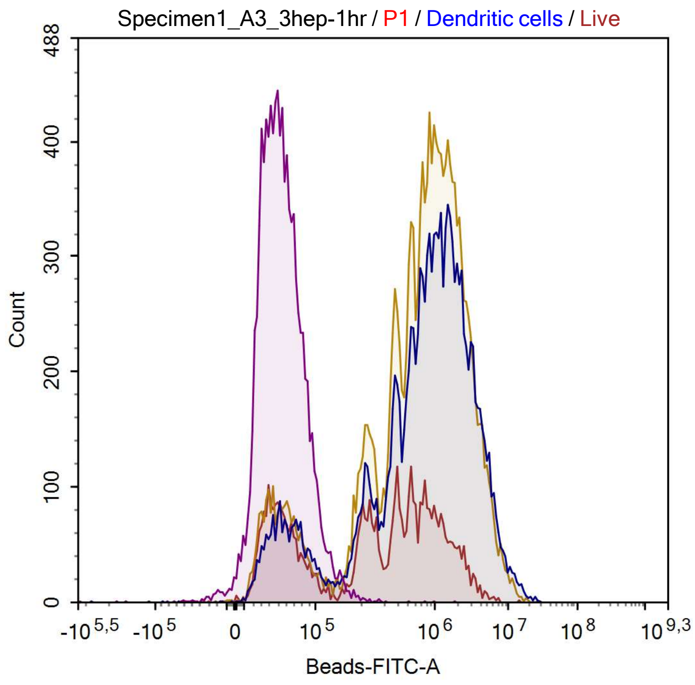

#	Sample	Gate	Count	Mean X	Median X
1	Specimen1_A3_3hep-1hr	Live	5.294	595.659	291.612
2	Specimen1_B3_3hep-3hr	Live*	16.998	1.315.019	814.667
3	Specimen1_C3_3hep-6hr	Live*	14.246	1.563.690	895.797
4	Specimen1_D1_unt-[illegible]	Live*	9.839	55.326	51.407

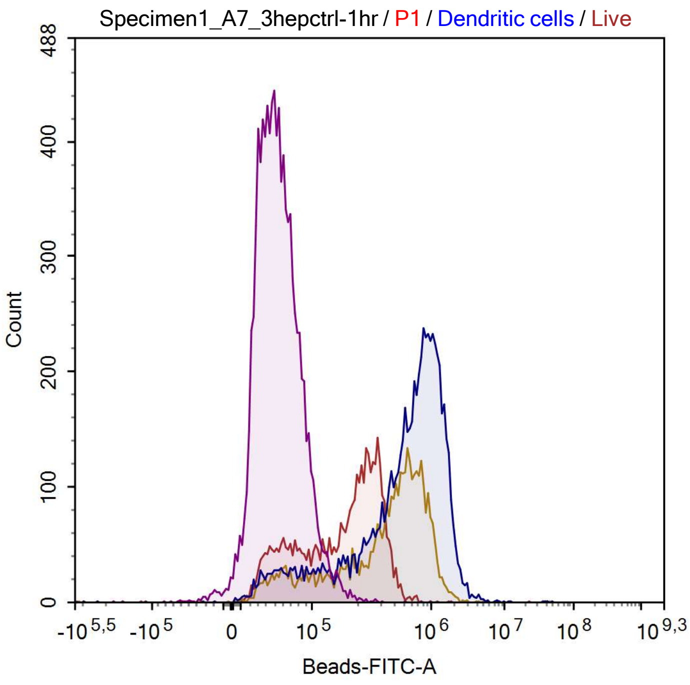

[illegible]	Sample	Gate	Count	Mean X	Median X
[illegible]	Specimen1_A7_3hepctrl-1hr	Live	3.975	196.757	175.293
[illegible]	Specimen1_B7_3hepctrl-3hr	Live*	3.940	490.572	405.389
[illegible]	Specimen1_C7_3hepctrl-6hr	Live*	7.078	761.769	627.019
[illegible]	Specimen1_D1_unt-[illegible]	Live*	9.839	55.326	51.407

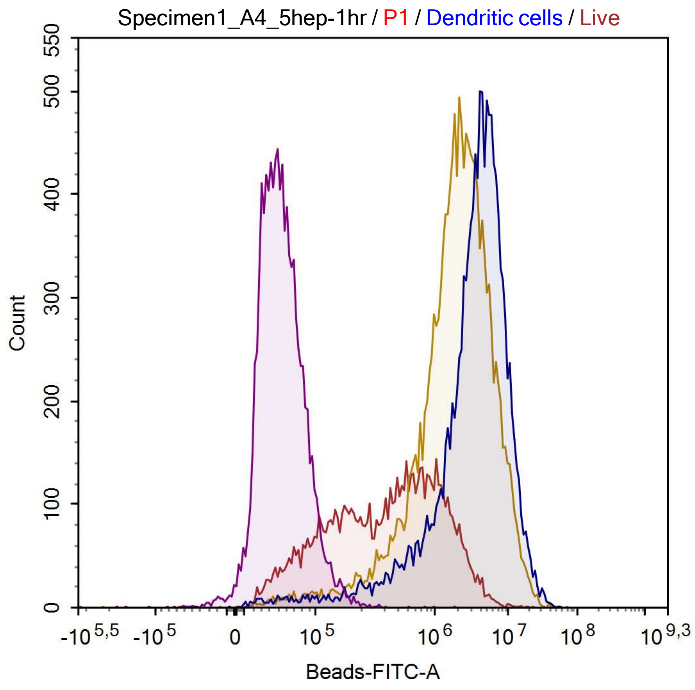

#	Sample	Gate	Count	Mean X	Median X
1	Specimen1_A4_5hep-1hr	Live	7.802	694.833	344.183
2	Specimen1_B4_5hep-3hr	Live*	15.583	3.124.614	2.068.289
3	Specimen1_C4_5hep-6hr	Live*	13.056	4.908.621	3.847.195
4	Specimen1_D1_unt-[illegible]	Live*	9.839	55.326	51.407

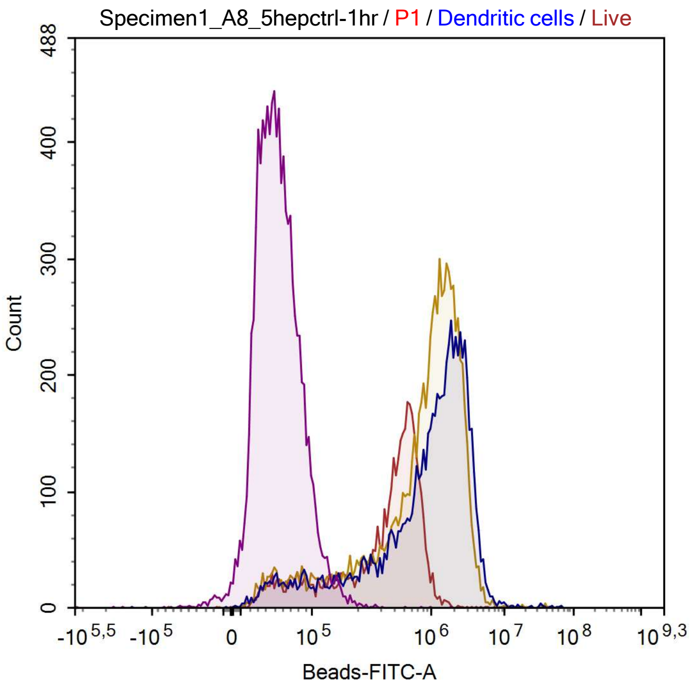

#	Sample	Gate	Count	Mean X	Median X
1	Specimen1_A8_5hepctrl-1hr	Live	3.927	474.666	407.560
2	Specimen1_B8_5hepctrl-3hr	Live*	8.515	1.259.363	1.041.578
3	Specimen1_C8_5hepctrl-6hr	Live*	7.340	1.561.640	1.183.339
4	Specimen1_D1_unt-[illegible]	Live*	9.839	55.326	51.407

All donors combined 6hr timepoint - FLU

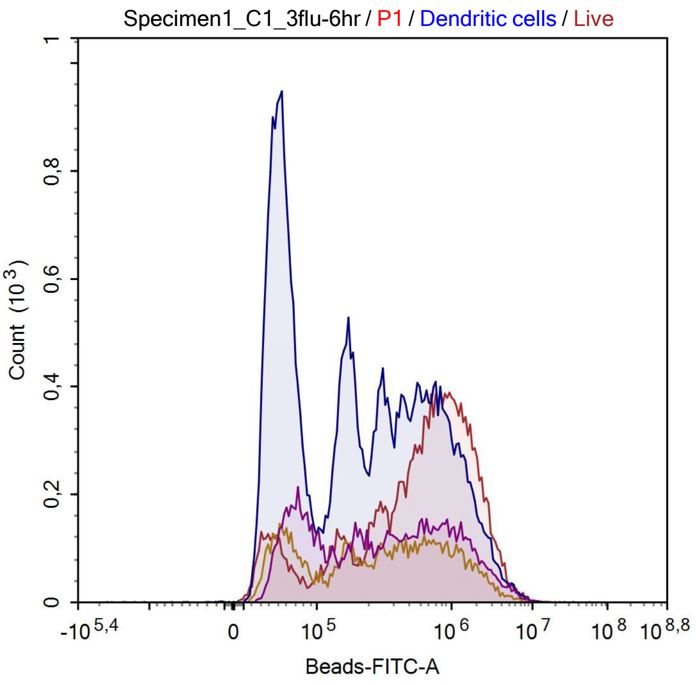

#	Sample	Gate	Count	Mean X	Median X
1	Specimen1_C1_3flu-6hr	Live	19.496	898.968	574.998
2	Donor 31_C1_3flu-6hr	Live*	9.057	574.420	249.097
3	Donor 41_C1_3flu-6hr	Live*	38.209	438.813	163.632
4	Donor 45 C1 3flu-6hr	Live*	12.152	647.736	250.656

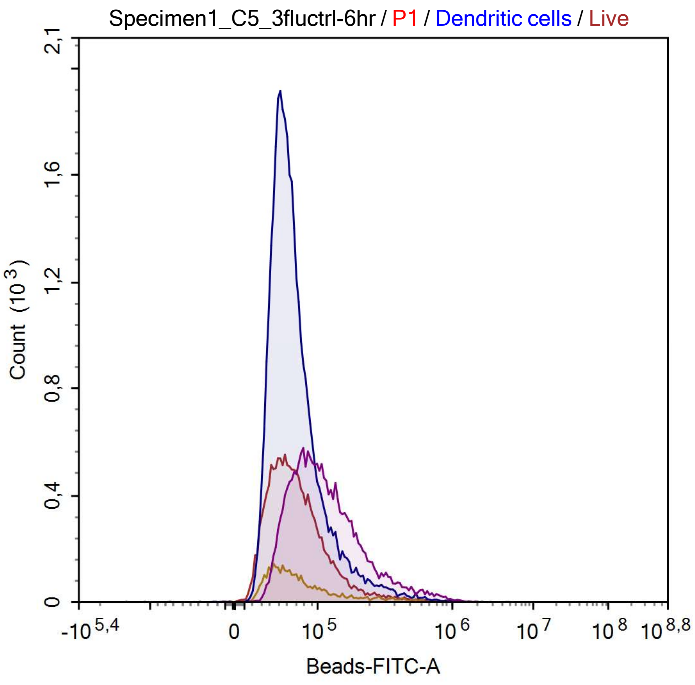

#	Sample	Gate	Count	Mean X	Median X
1	Specimen1_C5_3fluctrl-6hr	Live	14.836	80.145	65.771
2	Donor 31_C5_3fluctrl-6hr	Live*	3.508	102.194	66.283
3	Donor 41_C5_3fluctrl-6hr	Live*	35.635	76.946	61.943
4	Donor 45 C5 3fluctrl-6hr	Live*	18.815	132.241	101.177

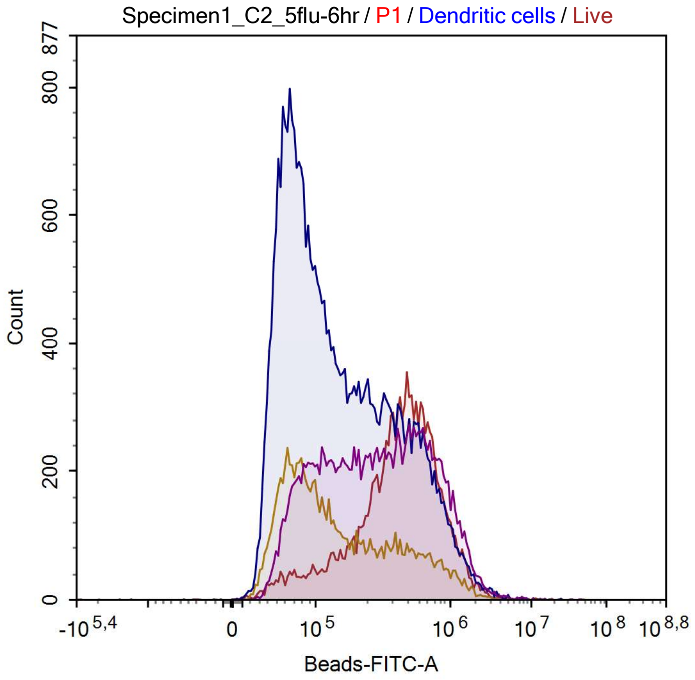

#	Sample	Gate	Count	Mean X	Median X
1	Specimen1_C2_5flu-6hr	Live	12.068	466.762	356.032
2	Donor 31_C2_5flu-6hr	Live*	9.603	261.009	114.100
3	Donor 41_C2_5flu-6hr	Live*	32.444	235.225	114.565
4	Donor 45 C2 5flu-6hr	Live*	17.672	414.182	230.279

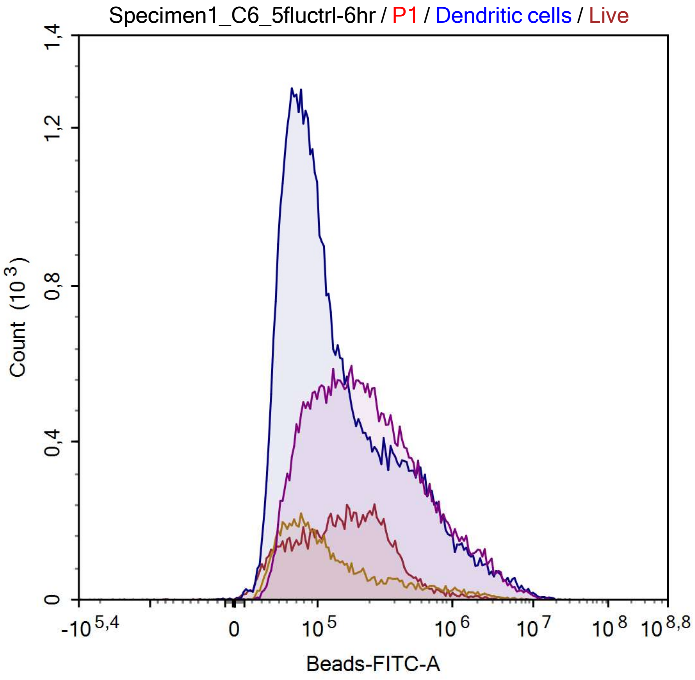

#	Sample	Gate	Count	Mean X	Median X
1	Specimen1_C6_5fluctrl-6hr	Live	11.761	197.478	140.344
2	Donor 31_C6_5fluctrl-6hr	Live*	8.031	247.583	98.258
3	Donor 41_C6_5fluctrl-6hr	Live*	52.039	317.900	105.272
4	Donor 45 C6 5fluctrl-6hr	Live*	35.744	428.219	176.819

All donors combined 6hr timepoint - HEP

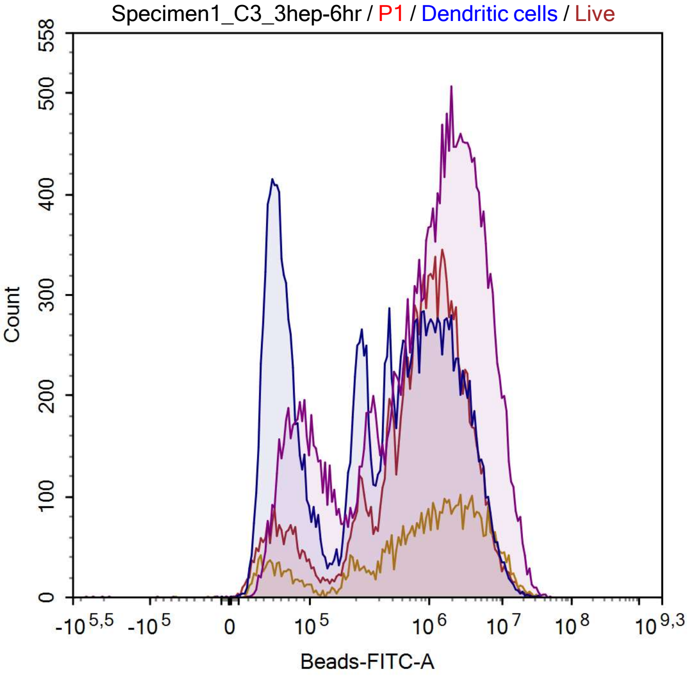

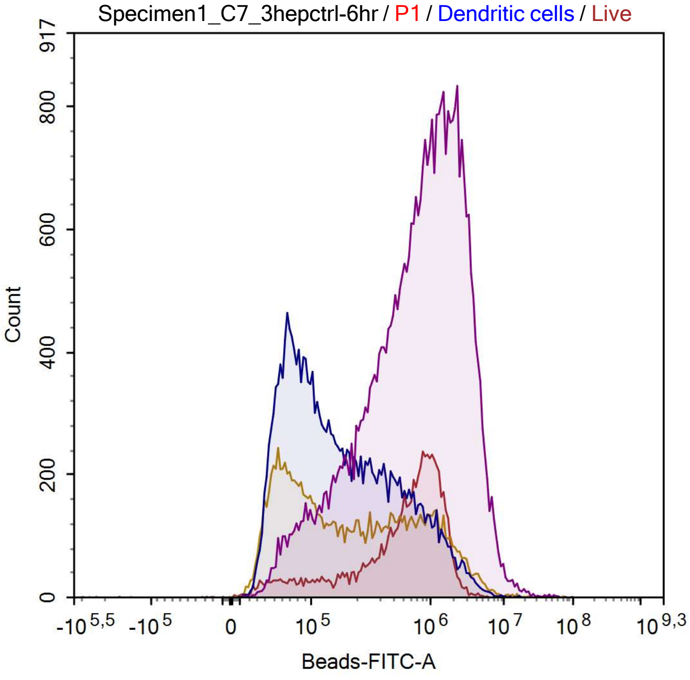

#	Sample	Gate	Count	Mean X	Median X
1	Specimen1_C3_3hep-6hr	Live	14.246	1.563.690	895.797
2	Donor 31_C3_3hep-6hr	Live*	5.300	2.507.104	1.141.961
3	Donor 41_C3_3hep-6hr	Live*	20.978	1.072.366	360.326
4	Donor 45_C3_3hep-6hr	Live*	25.633	2.508.999	1.172.151

#	Sample	Gate	Count	Mean X	Median X
1	Specimen1_C7_3hepctrl-6hr	Live	7.078	761.769	627.019
2	Donor 31_C7_3hepctrl-6hr	Live*	12.654	607.275	168.234
3	Donor 41_C7_3hepctrl-6hr	Live*	20.316	357.293	131.236
4	Donor 45_C7_3hepctrl-6hr	Live*	37.711	1.454.734	835.261

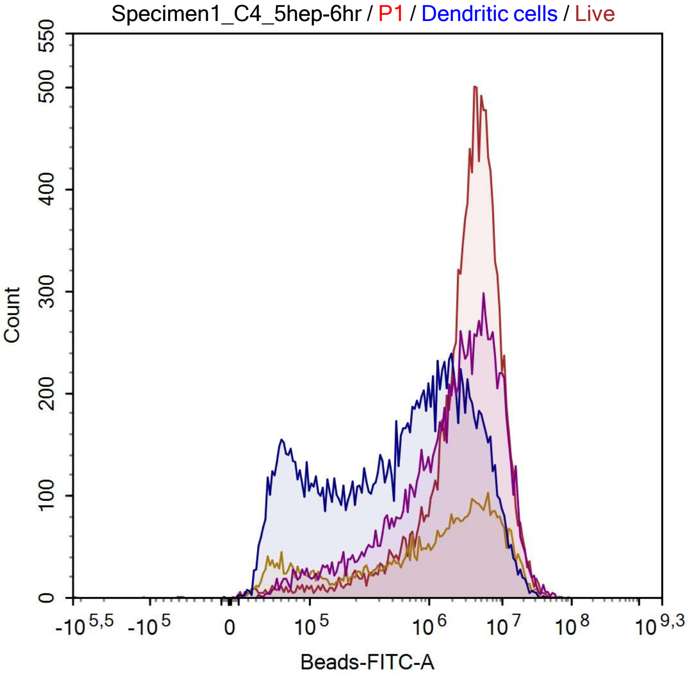

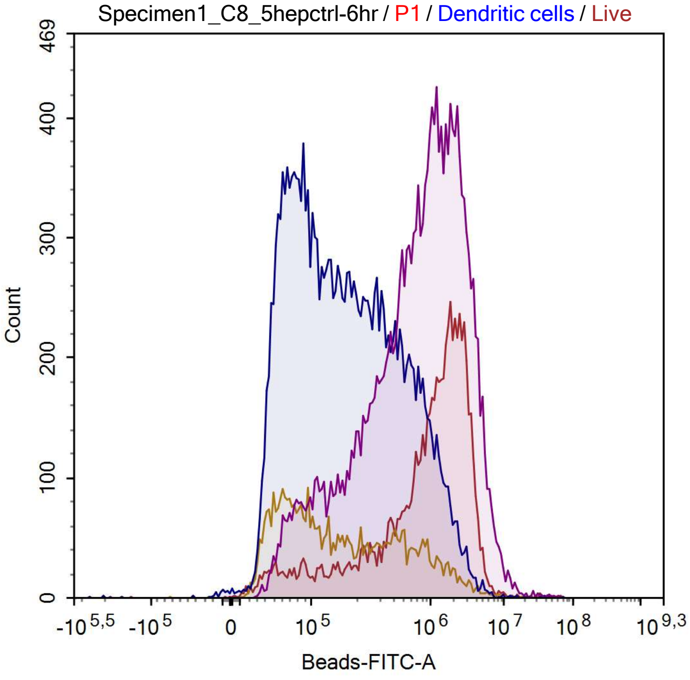

#	Sample	Gate	Count	Mean X	Median X
1	Specimen1_C4_5hep-6hr	Live	13.056	4.908.621	3.847.195
2	Donor 31_C4_5hep-6hr	Live*	5.234	3.710.281	1.614.936
3	Donor 41_C4_5hep-6hr	Live*	15.764	1.852.649	611.403
4	Donor 45_C4_5hep-6hr	Live*	11.807	4.327.917	2.545.932

#	Sample	Gate	Count	Mean X	Median X
1	Specimen1_C8_5hepctrl-6hr	Live	7.340	1.561.640	1.183.339
2	Donor 31_C8_5hepctrl-6hr	Live*	4.620	477.545	140.718
3	Donor 41_C8_5hepctrl-6hr	Live*	20.336	363.463	152.320
4	Donor 45_C8_5hepctrl-6hr	Live*	18.985	1.458.732	829.042
